# Discovery of an effective anti-inflammatory agent for inhibiting the activation of NF-κB

**DOI:** 10.1080/14756366.2023.2225135

**Published:** 2023-06-16

**Authors:** Yaoyao Yan, Qi Lv, Feilong Zhou, Yujie Jian, Liu Xinhua, Xing Chen, Yong Hu

**Affiliations:** a School of Pharmacy, Anhui Province Key Laboratory of Major Autoimmune Diseases Anhui Medical University, Hefei, PR China; b School of Public Health, Key Laboratory of Population Health Across Life Cycle, Anhui Medical University, Hefei, PR China; c Anhui Academy of Agricultural Sciences, Agricultural Products Processing Institute, Hefei, P. R. China

**Keywords:** LPS-induced inflammation, NO, NF-κB pathway, anti-inflammation small molecule

## Abstract

In this study, based on the effect of compounds on the activation of NF-κB and NO release, compound **51** was discovered as the best one with NO release inhibition IC_50_ value was 3.1 ± 1.1 μM and NF-κB activity inhibition IC_50_ value was 172.2 ± 11.4 nM. Compound **51** could inhibit the activation of NF-κB through suppressing phosphorylation and nuclear translocation of NF-κB, and suppress LPS-induced inflammatory response in RAW264.7 cells, such as the over-expression of TNF-α and IL-6, which were target genes of NF-κB. This compound also showed preferable anti-inflammatory activity in *vivo*, including alleviating significantly gastric distention and splenomegaly caused by LPS stimulation, reducing the level of oxidative stress induced by LPS, and inhibiting the expression of IL-6 and TNF-α in serum. Thus, it’s reasonable to consider that this compound is a promising small molecule with anti-inflammatory effect for inhibiting the NF-κB signalling pathway.

## Introduction

Inflammation is a series of complex defense-related response caused by various injury factors, with red, swelling, heat, pain as the main characteristics^1^. The body’s inflammatory response causes cellular changes and immune responses that result in repair of the damaged tissue and cellular proliferation at the injured sites^2^. When inflammation becomes chronic or lasts too long, it may be harmful and lead to disease, even creates an environment that is suitable for the development of various of cancers^3^. Various signalling pathways are key contributors in causing epigenetic changes, and switching on these internal mutations^4^. Therefore, treating the inflammatory causes is always important.

**Figure 1.**
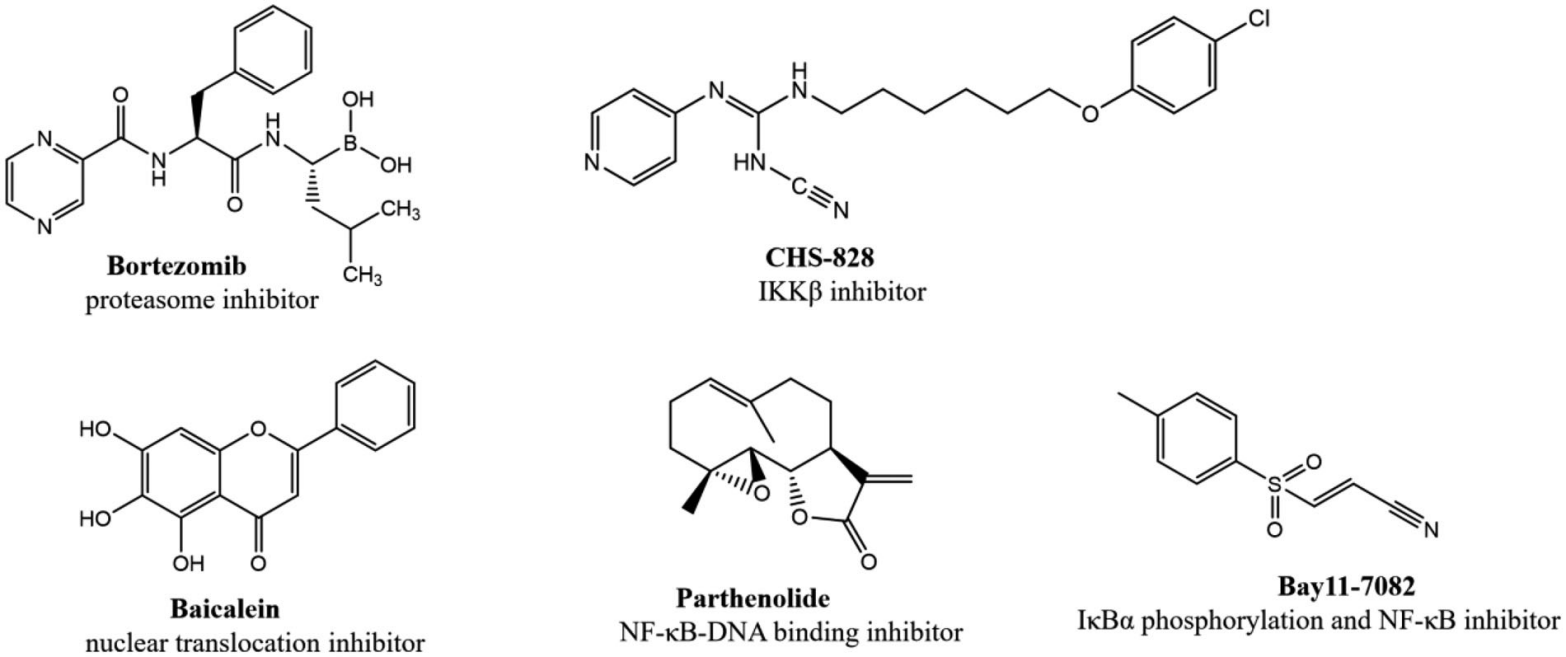
Some reported NF-κB inhibitors.

Nuclear factor-κB (NF-κB) is a nuclear transcription factor that plays an important role in cell growth, proliferation, differentiation, apoptosis and carcinogenesis^5–8^. The NF-κB pathway has long been considered as a typical inflammatory signalling pathway, it regulates proinflammatory cytokine production, leukocyte recruitment, or cell survival, which are important contributors to the inflammatory response^9^^,^^10^. It’s reported that the activation of NF-κB will induce expression of pro-inflammatory cytokines such as tumour necrosis factor-α (TNF-α), and interleukin-6 (IL-6), which lead to inflammatory diseases, including arthritis^11^, psoriasis^12^, chronic obstructive pulmonary disease (COPD)^13^ and inflammatory bowel disease (IBD)^14^. Thus, inhibiting the activation of NF-κB is a promising approach to alleviate inflammation. Currently, several compounds have been identified as NF-κB inhibitors^15–18^ (Figure 1).

It has been reported that LPS induced the activation of NF-κB, promoting the release of proinflammatory cytokines, such as NO, IL-6 and TNF-α^19^. Excessive NO can induce the development of inflammatory diseases^20^^,^^21^. Therefore, inhibiting the excessive secretion of NO effectively is one of the important measures to control the inflammatory response. In this study, we used LPS-stimulated RAW264.7 cell model to measure the effect of compounds on NO release, and a dual-luciferase reporter assay to detect the effect of compounds on NF-κB activity in HEK293T cells, which is a cell line with high efficiency of transfection. Through preliminary evaluation of anti-inflammatory activity, we found that compound **6** with the structural characteristics of polysubstituted pyridine in a library of more than 3000 compounds with structural diversity showed good activity and has the potential for modification. Through a series of structural optimisation and biological evaluation, compound **51** (NO release inhibition activity IC_50_=3.1 ± 1.1 *μ*M, NF-κB transcriptional inhibition activity IC_50_=172.2 ± 11.4 nM) was selected as our title compound. Further studies showed that compound **51** could inhibit LPS-induced phosphorylation of NF-κB p65 and IκB in RAW264.7 cells. In HEK293T cells, compound **51** could block the nuclear translocation of p65 and p50, which induced by TNF-α. Above results indicated that compound **51** could inhibit the activation of NF-κB signal. We also verified the effect of compound **51** on target genes of NF-κB, such as TNF-α and IL-6, the results showed that compound **51** inhibited the expression of TNF-α and IL-6.The results of the in *vitro* and in *vivo* experiments showed that the compound is a promising anti-inflammatory compound.

## Result and discussion

### Synthesis of aryl amides (6–52)

**Scheme 1.**
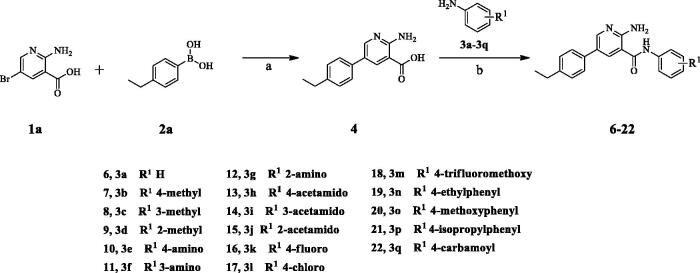
**Synthesis of *aryl amide (6–22)***. **Reagents and conditions**: (a) **1a** (1.0 eq.), **2a** (1.0 eq.), K_3_PO_4_ (1.5 eq.), Pd(PPh_3_)_4_ (0.05 eq.), 80 °C, 1,4-dioxane/H_2_O (3:1), Ar_2_ atmosphere, 12 h; (b) **5** (1.0 eq.), aniline (1.1 eq.), DIPEA (4.0 eq.), HATU (1.2 eq.), room temperature, DCM, 18 h.

**Scheme 2.**
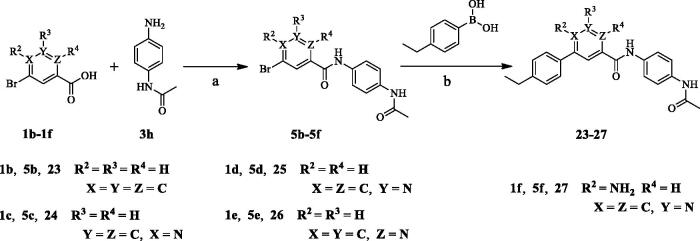
**Synthesis of *aryl amide (23–37)***. **Reagents and conditions**: (a) Acid (1.0 eq.), **3h** (1.0 eq.), HATU (1.2 eq.), DIPEA (4.0 eq.), RT, DCM, 18 h; (b) Bromide (1.0 eq.), **2a** (1.0 eq.), K_3_PO_4_ (1.5 equiv), Pd(PPh_3_)_4_ (0.05 eq.), 80 °C, Ar_2_ atmosphere, 1,4-dioxane/H_2_O (3:1), 12 h.

**Scheme 3.**
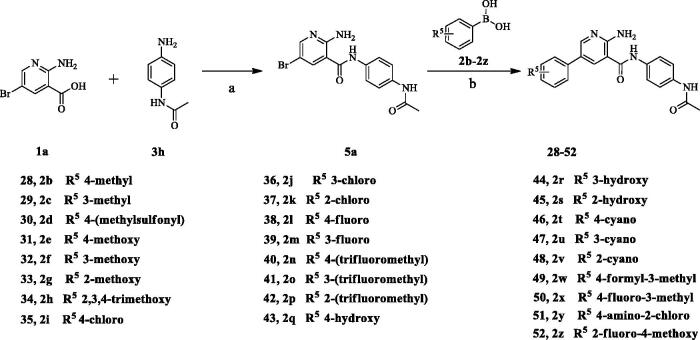
**Synthesis of *aryl amide (28–52)***. **Reagents and conditions**: (a) **1a** (1.0 eq.), **3H** (1.0 eq.), HATU (1.2 eq.), DIPEA (4.0 eq.), RT, DCM, 18 h; (b) **4a** (1.0 eq.), boric acid (1.0 eq.), K_3_PO_4_ (1.5 equiv), Pd(PPh_3_)_4_ (0.05 eq.), 1,4-dioxane/H_2_O, Ar_2_ atmosphere, 80 °C, 12 h.

The target compounds (**6**–**52**) are synthesised according to the route shown in Schemes 1–3. The intermediate **4** can be obtained by Suzuki reaction^22^^,^^23^ between the key raw materials 2-amino-5-bromonetinic acid (**1a**) and (4-ethylphenyl)boronic acid(**2a)**. The title compound **6**–**22** are prepared by amide condensation reaction between different substituted aniline (**3a**–**3q**) and carboxylic acid intermediate **4**. The preparation of intermediates (**5a**–**5f**) and target compounds (**23–27**, **28**–**52**) followed the same reaction conditions.

### Anti-inflammatory activity and SAR study

Through screening the in-house compound library, compound **6** was found to have effective anti-inflammatory activity, moderate inhibition on LPS-induced NO release, but with undesirable inhibition effect on NF-κB transcriptional activity(NO release inhibition IC_50_=19.7 ± 2.6 *μ*M, NF-κB activity inhibition IC_50_=1619.7 ± 13.2 nM). Therefore, we identified compound **6** as the HIT compound in this study. In order to obtain more excellent lead compound with anti-inflammatory activity, three rounds of structural modification of compound **6** were successfully implemented.

As listed in Table 1, we first considered to replace the group on the benzene ring at C3 position of pyridine core to observe the change of anti-inflammatory activity. The introduction of methyl (**7**–**9**) did not change the activity (NO IC_50_ = 15.2–30.2 *μ*M), but the introduction of amino (**10**–**12**) significantly improved the inhibitions of NO release (NO IC_50_ = 0.9–2.3 *μ*M) and NF-κB reporter activity (NF-κB reporter IC_50_ = 95.8–570.9 nM), in which the inhibition of NO release was increased by 10–20 folds, and the inhibition of NF-κB reporter was increased by 3–16 folds. Unfortunately, these three compounds showed strong cytotoxicity, which may not be suitable as lead compounds for the development of anti-inflammatory drugs. Further, the amino groups were replaced with acetylamino groups to obtain compound **13**–**15**. Similar to compounds **10**–**12**, compounds **14** (NO IC_50_ = 3.2 *μ*M, NF-κB reporter IC_50_ = 167.4 nM) and **15** (NO IC_50_ = 1.1 *μ*M, NF-κB reporter IC_50_ = 532.5 nM) also showed strong anti-inflammatory activity and strong cytotoxicity. It is gratifying to note that the anti-inflammatory activity of compound **13** (NO IC_50_ = 10.2 *μ*M, NF-κB IC_50_ = 713.9 nM) was not significantly improved as that of other compounds, but the cytotoxicity is reduced. Next, halogens, trifluoromethoxy, ethyl, methoxy, isopropyl and carbamoyl were introduced to the *para* position of benzene ring to obtain compounds **16**–**22**. The introduction of halogens (**16**–**17**), trifluoromethoxy (**18**) and carbamoyl (**22**) led to the loss of anti-inflammatory activity of the compounds. The introduction of ethyl (**19**), methoxy (**20**) and isopropyl (**21**) did not cause the fluctuation of anti-inflammatory activity, which was consistent with the introduction of methyl (**7**). Considering the changes in activity and cytotoxicity, compound **13** was identified as the node compound for the next round of transformation, although its activity was not the best.

**Table 1.** Structure-activity relationship study at position 3 of the pyridine core.

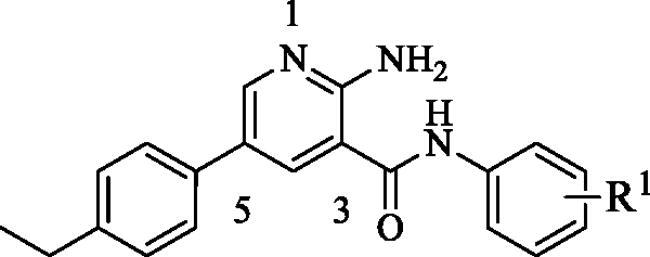				
NO.	R^1^	NO inhibition IC_50_ (*μ*M)^a^	NF-κB reporter IC_50_(nM)^b^	Cytotoxicity IC_50_(*μ*M)^c^
**6**	H	19.7 ± 2.6	1619.7 ± 13.2	>25
**7**	4Me	26.7 ± 3.2	>2000	>25
**8**	3Me	15.2 ± 2.8	1328.6 ± 11.5	>25
**9**	2Me	30.2 ± 4.0	>2000	>25
**10**	4NH_2_	2.3 ± 0.9	419.7 ± 10.4	7.3 ± 1.2
**11**	3NH_2_	1.7 ± 0.9	95.8 ± 6.2	>25
**12**	2NH_2_	0.9 ± 0.5	570.9 ± 9.3	4.7 ± 1.0
**13**	4NHCOMe	10.2 ± 5.4	713.9 ± 4.2	>25
**14**	3NHCOMe	3.2 ± 1.1	167.4 ± 8.4	>25
**15**	2NHCOMe	1.1 ± 0.4	532.5 ± 13.5	3.2 ± 0.8
**16**	4F	>50	>2000	>25
**17**	4Cl	>50	>2000	>25
**18**	4OCF_3_	>50	>2000	>25
**19**	4Et	28.5 ± 2.1	>2000	>25
**20**	4OMe	22.0 ± 0.8	>2000	>25
**21**	4iPr	>50	>2000	>25
**22**	4CONH_2_	>50	>2000	>25
**Bay11-7082**	–	2.1 ± 0.7	91.5 ± 10.3	22.7 ± 3.4

^a^NO inhibition IC_50_ was detected by Griess Reagent; ^b^NF-κB reporter assay: HEK293T cells were transfected with NF-κB reporter plasmid and stimulated by TNF-α. ^c^The toxicity of the compounds was detected by MTT assay.

Next, the 2-aminopyridine core was replaced to give compounds **23**–**27** to explore the importance of pyridine core. As listed in Table 2, any changes about the 2-aminopyridine led to decreased of inhibitions of NO release and NF-κB reporter activity (Table 2). Therefore, in the third round of transformation, we continued to keep the core of compound **13** unchanged.

**Table 2.** Structure-activity relationship study of the core.

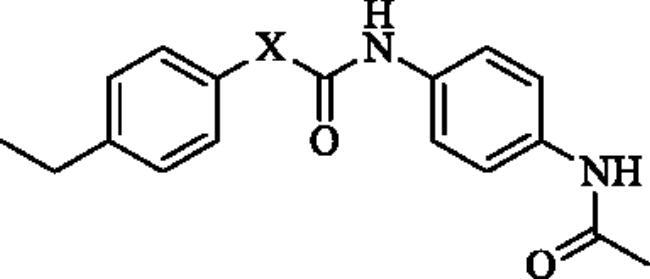				
NO.	X	NO inhibition IC_50_ (*μ*M)	NF-κB reporter IC_50_(nM)	Cytotoxicity IC_50_(*μ*M)
**23**	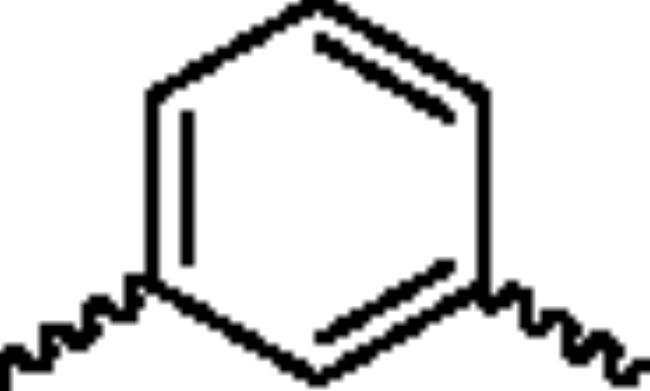	32.7 ± 3.4	>2000	>25
**24**	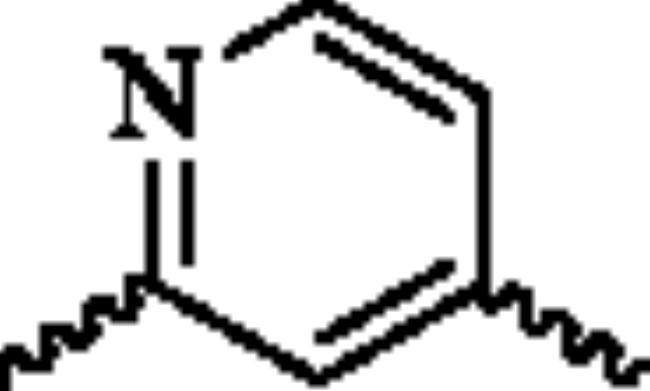	14.1 ± 1.7	763.2 ± 15.1	>25
**25**	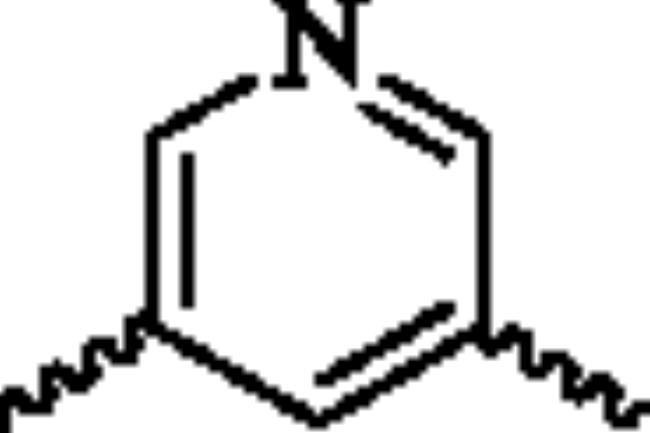	25.4 ± 3.1	1329.5 ± 16.3	>25
**26**	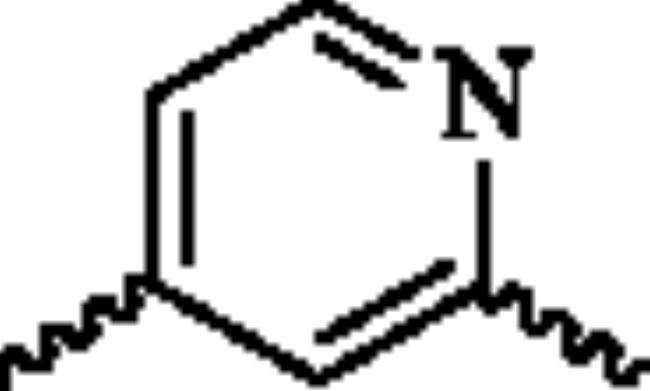	19.5 ± 3.2	1962.3 ± 53.5	>25
**27**	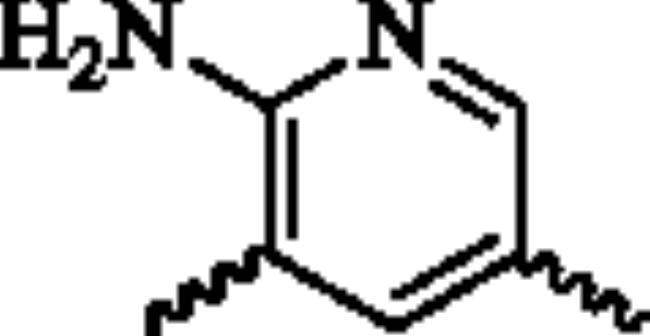	21.5 ± 2.6	1109.7 ± 12.4	>25
**Bay11-7082**	–	2.1 ± 0.7	91.5 ± 10.3	22.7 ± 3.4

^a^NO inhibition IC_50_ was detected by Griess Reagent; ^b^NF-κB reporter assay: HEK293T cells were transfected with NF-κB reporter plasmid and stimulated by TNF-α. ^c^The toxicity of the compounds was detected by MTT assay.

Finally, we began to discuss the effect of substituents on the benzene ring at C5 position of pyridine core on the anti-inflammatory activity of compounds. As listed in Table 3, after replacing ethyl with methyl (**28**, **29**) and methoxy (**31**–**34**), the anti-inflammatory activity decreased significantly. The introduction of chlorine atom on the *para* position of benzene ring significantly improved the anti-inflammatory activity of the compound (**35** NO IC_50_ = 4.2 *μ*M, NF-κB reporter IC_50_ = 206.5 nM), but the introduction of chlorine atoms on the *meta* and *ortho* positions led to the opposite result. Compounds **38** and **39** showed a similar trend. In addition, compounds **40**–**48** were obtained by introducing trifluoromethyl, hydroxyl and cyano groups at different positions of the benzene ring. Unfortunately, with the exception of compound **41** (NO IC_50_ = 8.4 *μ*M, NF-κB reporter IC_50_ = 306.6 nM), none of these compounds exhibited effective anti-inflammatory activity. Finally, in order to explore the possibility of activity change, we obtained compounds **49**–**52** by introducing poly-substituted benzene rings. Surprisingly, compounds **50** (NO IC_50_ = 8.6 *μ*M, NF-κB reporter IC_50_ = 577.1 nM) and **51** (NO IC_50_ = 3.1 *μ*M, NF-κB reporter IC_50_ = 172.7 nM) showed superior anti-inflammatory activity, of which compound **51** was the best. Compared with HIT compound **6**, its inhibition of NO release was increased by 6-folds, and the inhibition of NF-κB transcriptional activity was increased by nine-folds. In addition, the compound did not show obvious cytotoxicity. With comprehensive consideration, **51** was identified as the optimal one for further study.

**Table 3.** Structure-activity relationship study at position 5 of the pyridine core.

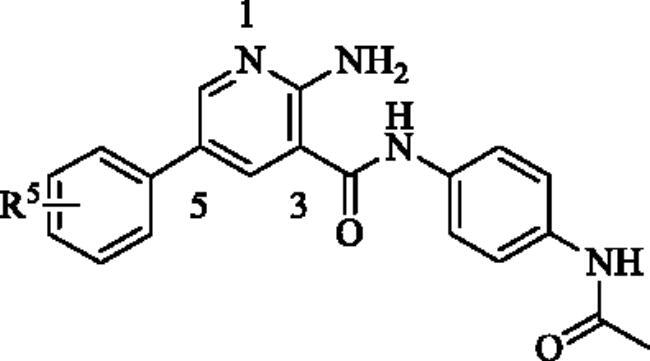				
NO.	R^5^	NO inhibition IC_50_ (*μ*M)^a^	NF-κB reporter IC_50_(nM)^b^	Cytotoxicity IC_50_(*μ*M)^c^
**28**	4Me	24.2 ± 4.1	>2000	>50
**29**	3Me	>50	>2000	>50
**30**	4SO_2_CH_3_	17.8 ± 2.7	1042.8 ± 17.5	>50
**31**	4OMe	>50	>2000	>50
**32**	3OMe	>50	>2000	>50
**33**	2OMe	>50	>2000	>50
**34**	2,3,4(OMe)_3_	>50	>2000	>50
**35**	4Cl	4.2 ± 0.8	206.5 ± 13.0	>50
**36**	3Cl	>50	>2000	>50
**37**	2Cl	>50	>2000	>50
**38**	4F	13.7 ± 1.8	827.3 ± 18.6	>50
**39**	3F	>50	>2000	>50
**40**	4CF_3_	26.5 ± 3.0	>2000	>50
**41**	3CF_3_	8.4 ± 1.7	306.6 ± 21.4	>50
**42**	2CF_3_	14.6 ± 2.3	1006.4 ± 18.4	>50
**43**	4OH	>50	>2000	>50
**44**	3OH	>50	>2000	>50
**45**	2OH	>50	>2000	>50
**46**	4CN	>50	>2000	>50
**47**	3CN	28.9 ± 2.1	>2000	>50
**48**	2CN	>50	>2000	>50
**49**	4-formyl-3-Me	>50	>2000	>50
**50**	4-F-3-Me	8.6 ± 1.9	577.1 ± 21.6	>50
**51**	4-NH_2_-2-Cl	3.1 ± 1.1	172.2 ± 11.4	>50
**52**	2-F-4-OMe	>50	1417.5 ± 24.7	>50
**Bay11-7082**	–	2.1 ± 0.7	91.5 ± 10.3	22.7 ± 3.4

^a^NO inhibition IC_50_ was detected by Griess Reagent; ^b^NF-κB reporter assay: HEK293T cells were transfected with NF-κB reporter plasmid and stimulated by TNF-α. ^c^The toxicity of the compounds was detected by MTT assay.

### Compound 51 suppressed NF-κB signalling activation

**Figure 2.**
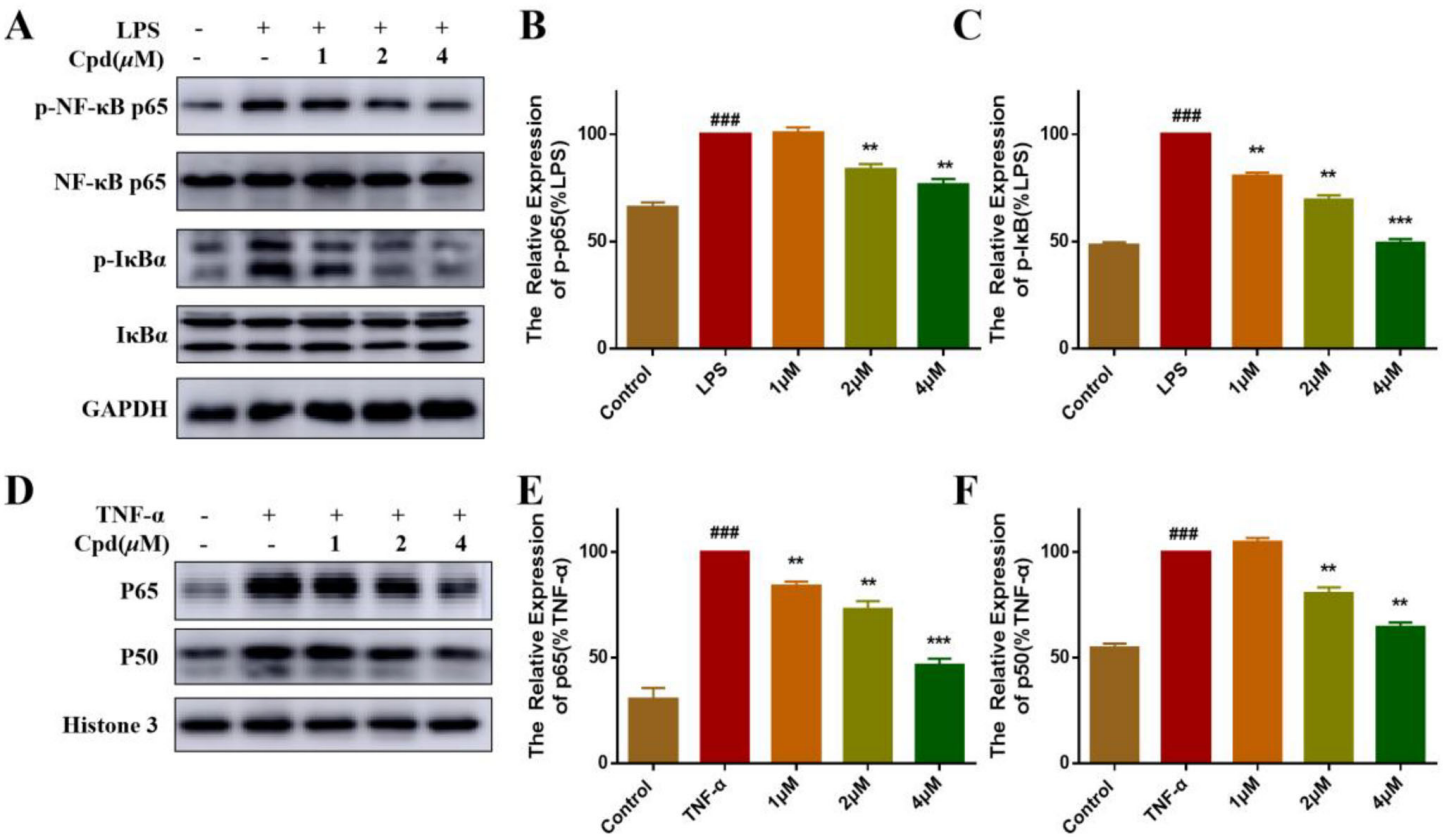
Compound **51** suppressed the activation of NF-κB. (A–C) Compound **51** inhibited the phosphorylation of P65 and IκBα. RAW264.7 cells were treated with compound **51** for different concentrations and then stimulated by LPS. The cell lysates were analysed using WB. ^###^*p < *0.001 compared with control group. **p < 0.01, ***p < 0.001 compared to LPS group. (D–F) Compound **51** inhibited TNFα-induced nuclear translocation of p65 and p50. HEK293T were treated with compound **51** for different concentrations and then stimulated by TNF-α. The nuclear protein was analysed using WB. ^###^*p* < 0.001 compared with control group. **p < *0*.01, ***p  <  0.001 compared to TNF-α group.

Under normal conditions, inactivated NF-κB is located in the cytoplasm and binds to IκB inhibitors^24^. Activation by inflammatory stimuli leads to the release and nuclear translocation of NF-κB, which shows as the breakdown and phosphorylation of the p65-IκBα complex^25^. As shown in Figure 2(A–C), LPS induced phosphorylation of p65 and IκBα protein, and after pre-treatment with different concentrations of compound **51**, the LPS-induced phosphorylation of p65 and IκBα was suppressed significantly in a dose dependent manner. Studies reported that TNFα-activated NF-κB transcriptional activity is accompanied by the nuclear translocation of p65 and p50. Therefore, we tested the effects of compound **51** on TNFα-induced nuclear translocation of p65 and p50 in HEK293T cells by isolating the nuclear fraction. As the result shown, TNF-α induced the appearance of RELA (p65) and NFKB1 (p50) subunits of NF-κB in the nucleus, and this effect of TNF-α was inhibited by compound in a dose dependent manner (Figure 2(D–E)).

### Compound 51 suppressed activation of MAPK signalling pathway

**Figure 3.**
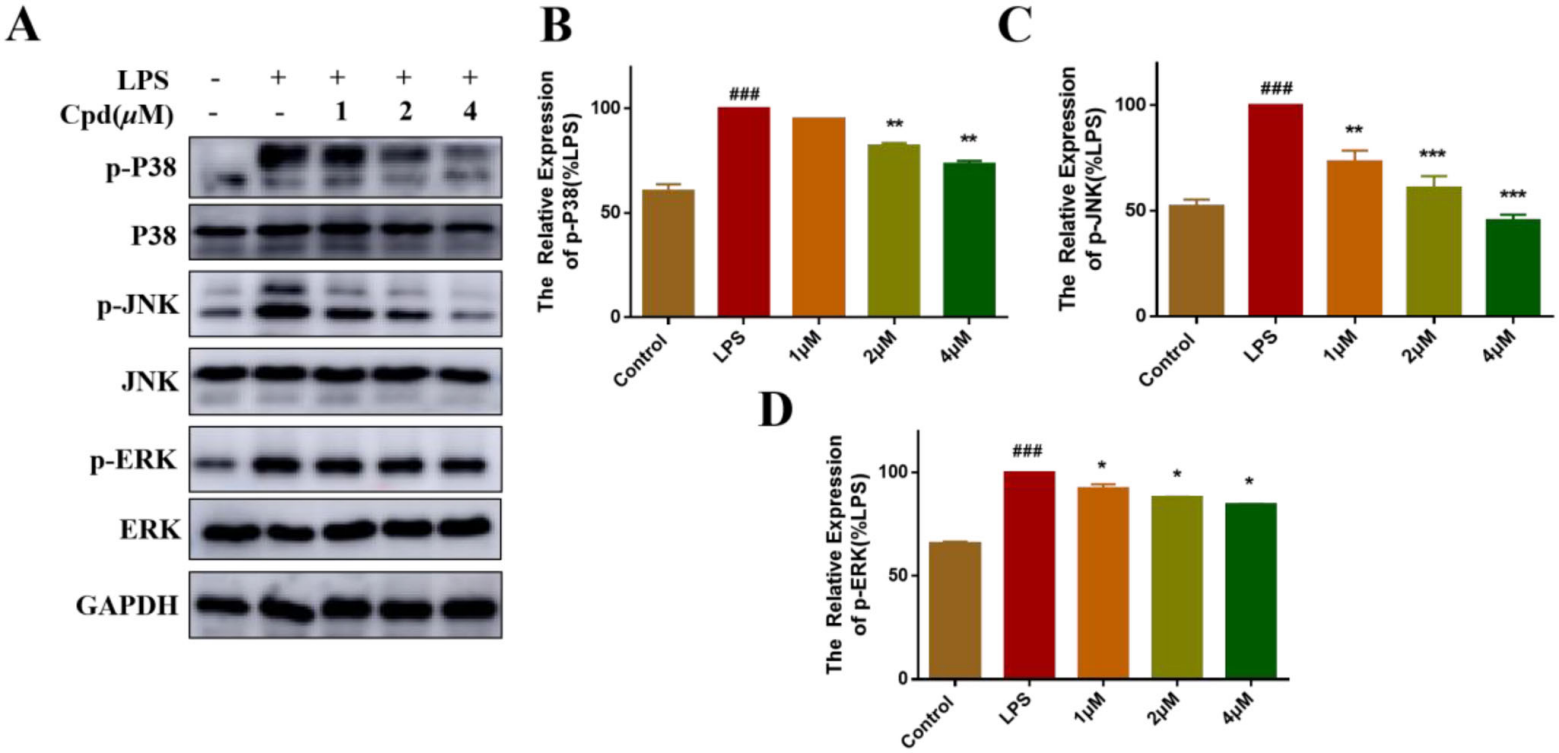
Compound **51** inhibited the activation of MAPKs signalling. (A–D) Compound **51** inhibited the phosphorylation of MAPKs. RAW264.7 cells were treated with compound **51** for different concentrations and then stimulated by LPS. The cell lysates were analysed using WB. ^###^*p* < 0.001 compared with control group. *p < 0.1, **p < 0.01, ***p < 0.001 compared to LPS group.

MAPKs signalling pathway also plays a key role in the regulation of inflammation^26^. LPS-stimulation activate NF-κB/MAPKs signalling pathway, allowing the transcription factors AP-1 translocate into the nucleus and promoting the production of inflammatory mediators^27^. Therefore, we measured the effects of compound **51** on LPS-regulated MAPK signalling activation by WB. As shown in Figure 3(A–D), LPS increased the expression of phosphorylation of P38, JNK and ERK, after treated with compound **51**, the phosphorylation level of MAPKs was significantly decreased.

### Compound 51 suppressed inflammatory response

**Figure 4.**
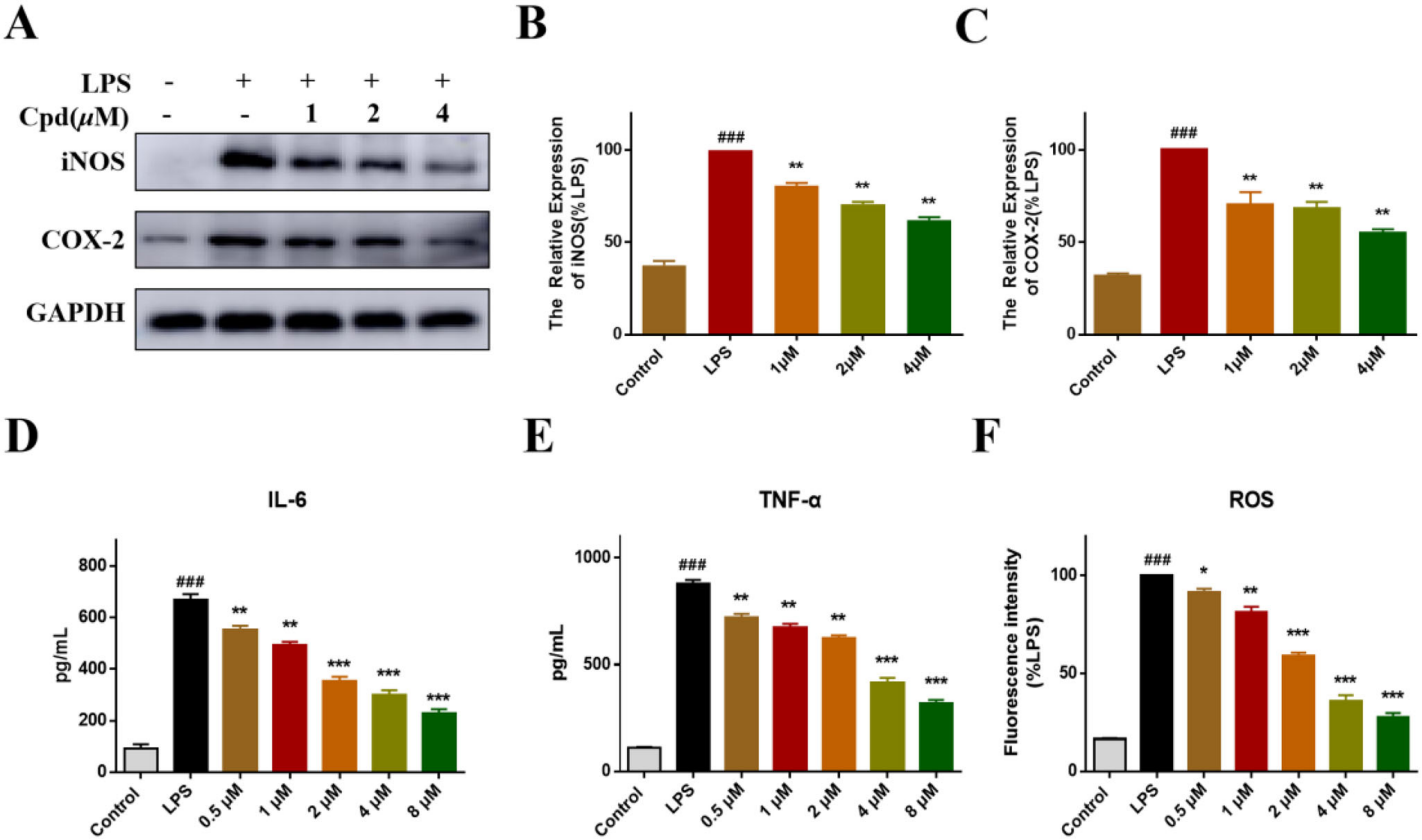
Compound **51** inhibited LPS-induced inflammatory response. (A–C) The expression of iNOS and COX-2. RAW264.7 cells were treated with compound **51** for 1 h, then stimulated by LPS for further 24 h. The samples were analysed by WB. (D). The level of IL-6. (E) The level of TNF-α. (F) The level of ROS. ^###^*p* < 0.001 compared with control group. *p < 0.1, **p < 0.01, ***p < 0.001 compared to LPS group.

iNOS is activated in the inflammatory response, which catalyses NO production^28^. COX-2 is also an inflammatory mediator, regulates the activity of NF-κB and other transcription factors, then participates in a series of physiological and pathological processes^29^. We found that the expression of iNOS and COX-2 was decreased significantly after treated with compound **51** (Figure 4(A–C)). Activation of NF-κB triggers transcription of various inflammatory factor genes, leading to the release of many inflammation factors such as TNF-α and IL-6, enhancing and amplifying the inflammatory response^30^^,^^31^. We detected effect of compound **51** on TNF-α and IL-6. As expected, compound **51** decreased the levels of TNF-α and IL-6 significantly in a dose dependent manner (Figure 4(D–E)). In addition, LPS stimulation will enhance the production of ROS, accumulation of ROS also triggered a series of inflammatory response^32^. We found that compound could decrease the level of ROS obviously (Figure 4(F)), which indicated that compound can alleviate the oxidative stress response induced by LPS. Above results suggested compound **51** could suppress the inflammatory response which induced by NF-κB activation.

### Compound 51 alleviated LPS-induced inflammation *in vivo*

**Figure 5.**
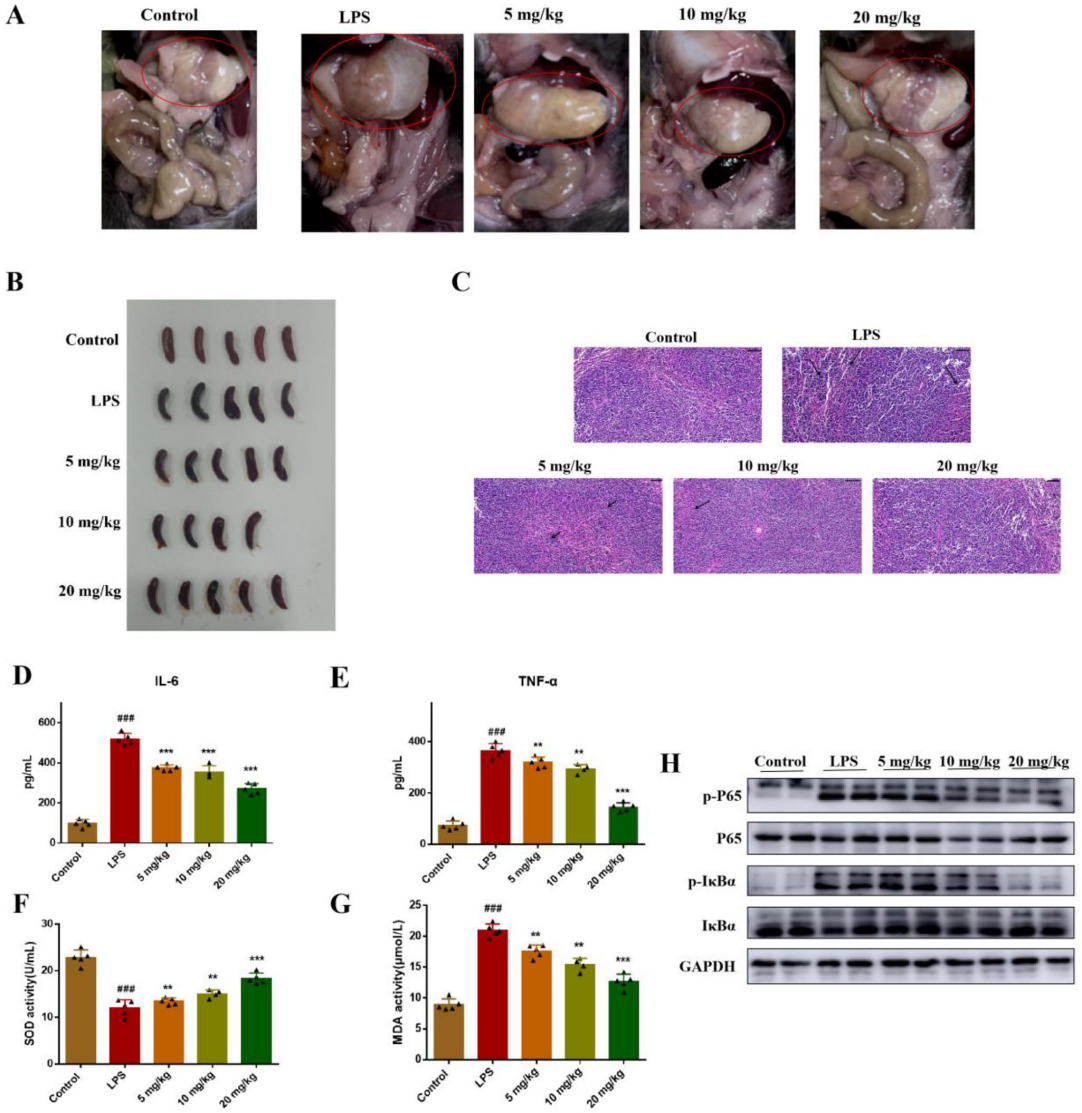
Compound **51** suppressed LPS-induced inflammatory response in *vivo*. (A-B) The picture of mice organs. (C) HE staining of spleen tissues. Scan bar:50 *μ*M. (D-E) The levels of IL-6 and TNF-α in blood. The samples were analysed by ELISA kits. (F–G) The SOD and MDA activity in blood. ^###^*p* < 0.001 compared with control group. *p < 0.1, **p < 0.01, ***p < 0.001 compared to LPS group. (H) The expression of NF-κB in splenic organ. The samples were analysed by WB.

*In vitro* activity showed that compound **51** significantly inhibited TNF-α induced NF-κB transcriptional activity and LPS-induced inflammatory response. To further evaluate *in vivo* anti-inflammatory activity of compound **51**, we established LPS-induced inflammation model of mice. We found that there were significant changes in organ tissues in mice treated with LPS, including gastric distention and splenomegaly (Figure 5(A,B)), and these symptoms were relieved after mice were treated with compound. The result of HE staining showed that LPS induced obvious injury of spleen, including broken of nuclear and numerous extramedullary haematopoietic cells. Compound **51** alleviated these injury in a dose-dependent manner (Figure 5(C)). In addition, after treated with 10 mg/kg LPS, the levels of IL-6 and TNF-α in blood of mice were increased, which suggested there is inflammatory response in mice. While IL-6 and TNF-α levels in mice treated with compound **51** were decreased obviously (Figure 5(D,E)). SOD and MDA were used as important indicators to evaluate the level of oxidative stress in antioxidant and oxidative capacity, respectively. We found that when compared with control group, SOD activity was decreased and MDA activity was increased in LPS group, compound **51** reversed their activity (Figure 5(F,G)). To confirm compound **51** exert anti-inflammatory effect *in vivo* through inhibiting NF-κB signalling pathway, we detected the expression of NF-κB in spleen. As shown in Figure 5(H), compound **51** suppressed the activation of NF-κB in splenic organ. The results indicated that compound **51** could alleviate LPS-induced inflammation through inhibiting NF-κB signalling pathway *in vivo*.

## Conclusion

The NF-κB pathway is implicated as a typical inflammatory signalling pathway, its activation leads to a series of inflammatory response. Thus, inhibiting NF-κB activation has long been considered as a promising approach to alleviate many inflammatory diseases. In this study, we synthesised a series of compounds, among them, compound **51** showed favourable NO release inhibition activity (IC_50_=3.1 *μ*M) and inhibited NF-κB transcriptional activity strongly. Further studies showed that this compound suppressed LPS and TNF-α induced NF-κB activation, including the effect on phosphorylation of and nuclear translocation of NF-κB. In addition, title compound also exert inhibition effect on MAPKs signal, the expression of phosphorylation of P38, JNK and ERK was significantly decreased. NF-κB activation triggered the downstream inflammatory response, we found that compound **51** decrease the levels of IL-6, TNF-α and ROS, which indicated this compound could alleviate LPS and TNF-α induced inflammatory response through inhibiting NF-κB activation. In *vivo* study, we evaluated the anti-inflammatory activity of compound **51** using animal model of acute inflammation induced by LPS. LPS stimulation caused changes in organ tissues in *vivo*, compound reduced inflammatory symptoms, including gastric distention and splenomegaly. The result also showed that title compound decreased the levels of IL-6 and TNF-α in blood. In addition, it was confirmed that this compound could decrease ROS level in *vitro*, which indicated it could alleviate oxidative stress. So we also measured the activity of SOD and MDA in *vivo*, two key indicators of evaluate the level of oxidative stress. We found that treatment of compound increased SOD activity and decreased MDA activity. What’s more, title compound suppressed the activation of NF-κB signalling in spleen, which indicated that this compound inhibited LPS-induced inflammation through inhibiting NF-κB activity. Overall, we obtained a novel compound with favourable anti-inflammatory activity in *vitro* and in *vivo*, which is meaningful for the development of NF-κB inhibitors and anti-inflammatory drugs.

## Experimental section (see supplementary data)

### Statistical analysis

The results were expressed as the means ± *SD*s (SPSS software), and the differences between groups were evaluated using Turkey’s method (GraphPad Software). Differences between data were considered significant when the *p* values was <0.05.

## Supplementary Material

Supplemental Material
