# Supplementary material for: Discovery of an effective anti-inflammatory agent for inhibiting the activation of NF-κB
Source: J Enzyme Inhib Med Chem. 2023 Jun 16;38(1):2225135. doi: 10.1080/14756366.2023.2225135 (PMC10281321; doi:10.1080/14756366.2023.2225135)

Supporting Information

4.1. Chemistry methods

Synthesis of 2-amino-5-(4-ethylphenyl)nicotinic acid ***(4)***. To a suspension of 2-amino-5-bromonicotinic acid (**1a**, 0.060 g, 0.26 mmol) in 1,4-dioxane (10 mL) was added **2a** (0.040, 0.26 mmol), $Pd(PPh_3)_4$ (0.015 g, 0.013 mmol) and K_3PO_4 (0.082 g, 0.39 mmol), and the flask was purged with Ar_2 and heated at 80 ℃ for 12 h. After the reaction was completed, the reaction solution was partitioned with ethyl acetate and water. The ethyl acetate phase was washed with saturated brine. The organic phase was treated with drying agent overnight and concentrated; the crude product was purified to afford 0.050 g of **4** as a light yellow solid (yield: 80%, purity > 80% was enough for subsequent reactions).

Synthesis of compounds ***5a-5f***. The synthesis of compounds **5a**-**5f** was exemplified by **5a**. *N*-(4-acetamidophenyl)-2-amino-5-bromonicotinamide (**5a**). To a solution of 2-amino-5-bromonicotinic acid (**1a**, 1.57 g, 6.03 mmol) in DCM (25 mL) was added HATU (2.75 g, 7.23 mmol). After reaction at RT for 1 h, then *N*-(4-aminophenyl)acetamide (**3h**, 0.91 g, 6.03 mmol) and DIPEA (4 mL, 12.05 mmol) were added. After the reaction was completed, the reaction solution was concentrated and the concentrate was re-dissolved in DMF (5 mL). The DMF solution was quenched with a large amount of water. The mixture was filtered to obtain **5a** as yellow solid, which was used in subsequent reactions without further purification.

Synthesis of compounds ***6-22***. The synthesis of compounds **6**-**22** was exemplified by **6**. *2-amino-5-(4-ethylphenyl)-N-phenylnicotinamide* (**6**). To a solution of **4** (0.145 g, 0.60

mmol) in DCM (15 mL) was added HATU (0.280 g, 0.72 mmol). After reaction at RT for 1 h, the aniline (**3a**, 0.062 g, 0.67 mmol) and DIPEA (0.4 mL, 1.21 mmol) were added thereto. After the reaction was completed, the reaction solution was concentrated and the concentrate was dissolved with ethyl acetate. The ethyl acetate phase was treated with drying agent overnight and concentrated; the crude product was purified to afford 2-amino-5-(4-ethylphenyl)-N-phenylnicotinamide as off-while solid (127 mg, yield: 60%, purity > 95%).

*Synthesis of compounds **23-52***. The synthesis of compounds **23-52** was exemplified by **51**. N-(4-acetamidophenyl)-2-amino-5-(4-amino-2-chlorophenyl)nicotinamide (**51**). To a suspension of **5a** (0.090 g, 0.26 mmol) in 1,4-dioxane (10 mL) was added phenylboronic acid (**2y**, 0.044 g, 0.26 mmol), $Pd(PPh_3)_4$ (0.015 g, 0.013 mmol) and K_3PO_4 (0.082 g, 0.39 mmol), and the flask was purged with Ar_2. The flask was sealed and heated at 80 ℃ for 12 h. After cooling to RT, the reaction solution was partitioned with ethyl acetate and water. The ethyl acetate phase was washed with saturated brine. The organic phase was treated with drying agent overnight and concentrated; the crude product was purified to afford 0.062 g of compound **51** as while solid. (yield: 60%, purity > 95%).

2-amino-5-(4-ethylphenyl)-N-phenylnicotinamide (**6**) white solid, MP: 214 ℃; ^{1}H NMR (600 MHz, DMSO-d_6) δ 10.28 (s, 1H), 8.47 (d, *J* = 2.3 Hz, 1H), 8.30 (d, *J* = 2.4 Hz, 1H), 7.71 (d, *J* = 7.3 Hz, 2H), 7.63 (d, *J* = 8.1 Hz, 2H), 7.41 – 7.34 (m, 2H), 7.30 (d, *J* = 7.9 Hz, 2H), 7.12 (t, *J* = 7.4 Hz, 1H), 7.07 (s, 2H), 2.64 (q, *J* = 7.6 Hz, 2H), 1.20 (t, *J* = 7.6 Hz, 3H). ^{13}C NMR (151 MHz, DMSO-d_6) δ 167.03, 158.39, 149.78, 142.87,

139.32, 135.57, 135.19, 129.06(2C), 128.79(2C), 126.24(2C), 124.31, 124.26, 121.33(2C), 110.34, 28.29, 16.21. HRMS (ESI): *m/z*$[M+H]^+$ calcd for C20H19N3O: 318.1601; found: 318.1609.

2-amino-5-(4-ethylphenyl)-N-(p-tolyl)nicotinamide (**7**) white solid, MP: 203 ℃; ^{1}H NMR (600 MHz, DMSO-d_6) δ 10.21 (s, 1H), 8.46 (s, 1H), 8.29 (s, 1H), 7.63 (d, *J* = 7.9 Hz, 2H), 7.59 (d, *J* = 8.1 Hz, 2H), 7.29 (d, *J* = 7.0 Hz, 2H), 7.17 (d, *J* = 7.9 Hz, 2H), 7.07 (s, 2H), 2.64 (q, *J* = 7.6, 7.2 Hz, 2H), 2.29 (s, 3H), 1.27 – 1.16 (m, 3H). ^{13}C NMR (151 MHz, DMSO-d_6) δ 166.84, 158.39, 149.67, 142.85, 136.76, 135.46, 135.21, 133.30, 129.45(2C), 128.77(2C), 126.24, 124.25(2C), 121.36, 110.38, 28.29, 20.98, 16.21. HRMS (ESI): *m/z*$[M+H]^+$ calcd for C21H21N3O: 332.1757; found: 332.1757.

2-amino-5-(4-ethylphenyl)-N-(m-tolyl)nicotinamide (**8**) white solid, MP: 278 ℃; ^{1}H NMR (600 MHz, DMSO-d_6) δ 10.20 (s, 1H), 8.46 (s, 1H), 8.29 (s, 1H), 7.63 (d, *J* = 8.2 Hz, 2H), 7.55 (s, 1H), 7.51 (d, *J* = 8.2 Hz, 1H), 7.30 (d, *J* = 7.5 Hz, 2H), 7.24 (t, *J* = 7.7 Hz, 1H), 7.07 (s, 1H), 6.94 (d, *J* = 7.8 Hz, 1H), 2.64 (q, *J* = 7.7 Hz, 2H), 2.32 (d, *J* = 2.4 Hz, 3H), 1.21 (t, *J* = 7.7 Hz, 2H). ^{13}C NMR (151 MHz, DMSO-d_6) δ 166.95, 158.39, 149.74, 142.86, 139.24, 138.21, 135.52, 135.19, 128.90, 128.78(2C), 126.24(2C), 124.99, 124.25, 121.83, 118.45, 110.34, 28.29, 21.65, 16.21. HRMS (ESI): *m/z*$[M+H]^+$ calcd for C21H21N3O: 332.1757; found: 332.1764.

2-amino-5-(4-ethylphenyl)-N-(o-tolyl)nicotinamide (**9**) white solid, MP: 183 ℃; ^{1}H NMR (600 MHz, DMSO-d_6) δ 10.01 (s, 1H), 8.48 (s, 1H), 8.41 (s, 1H), 7.64 (d, *J* = 8.2 Hz, 2H), 7.33 – 7.27 (m, 4H), 7.24 (t, *J* = 7.3 Hz, 1H), 7.19 (t, *J* = 7.5 Hz, 1H), 7.15 (s,

1H), 2.64 (q, J = 7.9 Hz, 2H), 2.24 (s, 3H), 1.20 (t, J = 7.6 Hz, 2H). ^{13}C NMR (151 MHz, DMSO-d_6) δ 167.00, 158.60, 149.80, 142.85, 136.63, 135.47, 135.19, 134.57, 130.81, 128.79(2C), 127.34, 126.65, 126.51, 126.17(2C), 124.26, 109.63, 28.29, 18.41, 16.20. HRMS (ESI): *m/z*[M+H]$^+$ calcd for C21H21N3O: 332.1757; found: 332.1761.

tert-butyl (4-(2-amino-5-(4-ethylphenyl)nicotinamido)phenyl)carbamate (**10**) white solid, MP: 269 ℃; ^{1}H NMR (600 MHz, DMSO-d_6) δ 10.19 (s, 1H), 9.32 (s, 1H), 8.46 (s, 1H), 8.29 (s, 1H), 7.63 (d, J = 7.8 Hz, 2H), 7.57 (d, J = 8.9 Hz, 2H), 7.44 (d, J = 8.4 Hz, 2H), 7.30 (d, J = 7.7 Hz, 2H), 7.08 (s, 2H), 2.64 (q, J = 7.7 Hz, 2H), 1.48 (s, 9H), 1.20 (t, J = 7.6 Hz, 3H). ^{13}C NMR (151 MHz, DMSO-d_6) δ 166.66, 158.36, 153.29, 149.61, 142.86, 136.07, 135.41, 135.19, 133.62, 128.77(2C), 126.23(2C), 124.25, 121.92(2C), 118.67, 110.36, 79.38, 28.63(3C), 28.29, 16.21. HRMS (ESI): *m/z*[M+H]$^+$ calcd for C25H28N4O3: 433.2234; found: 433.2243.

tert-butyl (3-(2-amino-5-(4-ethylphenyl)nicotinamido)phenyl)carbamate (**11**) white solid, MP: 225 ℃; ^{1}H NMR (600 MHz, DMSO-d_6) δ 10.26 (s, 1H), 9.39 (s, 1H), 8.46 (d, J = 2.4 Hz, 1H), 8.30 (d, J = 2.4 Hz, 1H), 7.95 (s, 1H), 7.64 (d, J = 8.2 Hz, 2H), 7.35 (d, J = 8.0 Hz, 1H), 7.30 (d, J = 8.2 Hz, 2H), 7.21 (t, J = 8.0 Hz, 1H), 7.10 (d, J = 8.2 Hz, 1H), 7.07 (s, 1H), 2.64 (q, J = 7.6 Hz, 2H), 1.48 (s, 9H), 1.21 (t, J = 7.6 Hz, 3H). ^{13}C NMR (151 MHz, DMSO-d_6) δ 166.98, 158.41, 153.22, 149.72, 142.84, 140.24, 139.58, 135.65, 135.21, 128.99, 128.77(2C), 126.23(2C), 124.22, 115.44, 114.49, 111.46, 110.29, 79.44, 28.61(3C), 28.30, 16.20. HRMS (ESI): *m/z*[M+H]$^+$ calcd for C25H28N4O3: 433.2234; found: 433.2237.

tert-butyl (2-(2-amino-5-(4-ethylphenyl)nicotinamido)phenyl)carbamate (**12**) white solid, MP: 269 ℃; ^{1}H NMR (600 MHz, DMSO-d_6) δ 9.88 (s, 1H), 8.59 (s, 1H), 8.49 (d, *J* = 2.3 Hz, 1H), 8.36 (d, *J* = 2.4 Hz, 1H), 7.62 (t, *J* = 9.1 Hz, 3H), 7.45 (d, *J* = 7.9 Hz, 1H), 7.29 (d, *J* = 7.9 Hz, 2H), 7.21 (t, *J* = 7.8 Hz, 1H), 7.16 – 7.11 (m, 3H), 2.64 (q, *J* = 7.6 Hz, 2H), 1.41 (s, 9H), 1.20 (t, *J* = 7.6 Hz, 3H). ^{13}C NMR (151 MHz, DMSO-d_6) δ 167.14, 158.49, 153.76, 149.94, 142.89, 135.69, 135.14, 133.16, 129.71, 128.77(2C), 127.17, 126.31, 126.16(2C), 124.24, 123.96, 109.76, 79.90, 28.49(3C), 28.29, 16.21. HRMS (ESI): *m/z*$[M+H]^+$ calcd for C25H28N4O3: 433.2234; found: 433.2241.

N-(4-acetamidophenyl)-2-amino-5-(4-ethylphenyl)nicotinamide (**13**) white solid, MP: 244 ℃; ^{1}H NMR (400 MHz, DMSO-d_6) δ 10.25 (s, 1H), 9.95 (s, 1H), 8.46 (d, *J* = 2.3 Hz, 1H), 8.29 (d, *J* = 2.4 Hz, 1H), 7.66 – 7.54 (m, 6H), 7.30 (d, *J* = 8.2 Hz, 2H), 7.09 (s, 2H), 2.64 (q, *J* = 7.6 Hz, 2H), 2.04 (s, 3H), 1.21 (t, *J* = 7.6 Hz, 3H). ^{13}C NMR (101 MHz, DMSO-d_6) δ 168.52, 166.74, 158.37, 149.66, 142.85, 135.89, 135.44, 135.18, 134.39, 128.79(2C), 126.22(2C), 124.22, 121.80(2C), 119.57(2C), 110.31, 28.29, 24.41, 16.24. HRMS (ESI): *m/z*$[M+H]^+$ calcd for C22H22N4O2: 375.1816; found: 375.1809.

N-(3-acetamidophenyl)-2-amino-5-(4-ethylphenyl)nicotinamide (**14**) white solid, MP: 263 ℃; ^{1}H NMR (600 MHz, DMSO-d_6) δ 10.30 (s, 1H), 9.98 (s, 1H), 8.46 (d, *J* = 2.3 Hz, 1H), 8.30 (d, *J* = 2.4 Hz, 1H), 8.04 (s, 1H), 7.64 (d, *J* = 8.1 Hz, 2H), 7.37 (d, *J* = 8.1 Hz, 1H), 7.34 (d, *J* = 8.7 Hz, 1H), 7.30 (d, *J* = 7.9 Hz, 2H), 7.26 (t, *J* = 8.0 Hz, 1H), 7.07 (s, 2H), 2.64 (q, *J* = 7.6 Hz, 2H), 2.05 (s, 3H), 1.21 (t, *J* = 7.6 Hz, 3H). ^{13}C NMR (151 MHz, DMSO-d_6) δ 167.69, 165.96, 157.32, 148.66, 141.79, 138.93, 138.49, 134.61, 134.12,

128.03, 127.71(2C), 125.16, 123.16(2C), 115.15, 114.10, 111.16, 109.25, 27.22, 23.42, 15.14. HRMS (ESI): *m/z*$[M+H]^+$ calcd for C22H28N4O2: 375.1816; found: 375.1816.

N-(2-acetamidophenyl)-2-amino-5-(4-ethylphenyl)nicotinamide (**15**) white solid, MP: 274 ℃; ^{1}H NMR (600 MHz, DMSO-d_6) δ 9.95 (s, 1H), 9.62 (s, 1H), 8.50 (s, 1H), 8.36 (s, 1H), 7.66 – 7.54 (m, 6H), 7.30 (d, *J* = 7.8 Hz, 2H), 7.23 – 7.19 (m, 2H), 7.17 (s, 2H), 2.64 (q, *J* = 7.6 Hz, 2H), 2.09 (s, 3H), 1.21 (t, *J* = 7.6 Hz, 3H). ^{13}C NMR (151 MHz, DMSO-d_6) δ 169.55, 166.88, 158.54, 149.89, 142.90, 135.50, 135.14, 132.52, 132.50, 132.45, 131.99, 130.46, 129.27, 128.82(2C), 126.79, 126.08(2C), 125.94, 125.14, 124.92, 124.22, 28.28, 24.12, 16.16. HRMS (ESI): *m/z*$[M+H]^+$ calcd for C22H28N4O2: 375.1816; found: 375.1810.

2-amino-5-(4-ethylphenyl)-N-(4-fluorophenyl)nicotinamide (**16**) white solid, MP: 227 ℃; ^{1}H NMR (600 MHz, DMSO-d_6) δ 10.33 (s, 1H), 8.47 (d, *J* = 2.4 Hz, 1H), 8.30 (d, *J* = 2.4 Hz, 1H), 7.72 (dd, *J* = 9.1, 5.0 Hz, 2H), 7.63 (d, *J* = 8.2 Hz, 2H), 7.30 (d, *J* = 8.1 Hz, 2H), 7.21 (t, *J* = 8.9 Hz, 2H), 7.08 (s, 2H), 2.64 (q, *J* = 7.6 Hz, 2H), 1.20 (t, *J* = 7.6 Hz, 3H). ^{13}C NMR (151 MHz, DMSO-d_6) δ 166.94, 159.70, 158.37, 158.11, 149.84, 142.88, 135.65, 135.64 (d, *J* = 2.6 Hz), 135.16, 128.79(2C), 126.22(2C), 124.26, 123.22 (2C, d, *J* = 7.9 Hz), 115.65 (2C, d, *J* = 22.3 Hz), 110.14, 28.29, 16.20. HRMS (ESI): *m/z*$[M+H]^+$ calcd for C20H18N3OF: 336.1507; found: 336.1512.

2-amino-N-(4-chlorophenyl)-5-(4-ethylphenyl)nicotinamide (**17**) white solid, MP: 275 ℃; ^{1}H NMR (600 MHz, DMSO-d_6) δ 10.38 (s, 1H), 8.48 (d, *J* = 2.3 Hz, 1H), 8.30 (d, *J* = 2.3 Hz, 1H), 7.76 (d, *J* = 8.9 Hz, 2H), 7.63 (d, *J* = 8.0 Hz, 2H), 7.43 (d, *J* = 8.8 Hz,

2H), 7.30 (d, *J* = 8.1 Hz, 2H), 7.08 (s, 2H), 2.64 (q, *J* = 7.6 Hz, 2H), 1.20 (t, *J* = 7.6 Hz, 3H). ^{13}C NMR (151 MHz, DMSO-d_6) δ 166.01, 157.30, 148.91, 141.83, 137.27, 134.55, 134.06, 127.90(2C), 127.72(2C), 126.84, 125.17(2C), 123.20, 121.71(2C), 108.97, 27.23, 15.14. HRMS (ESI): *m/z*[M+H]$^+$ calcd for C20H18N3OCl: 352.1211; found: 352.1205.

2-amino-5-(4-ethylphenyl)-N-(4-(trifluoromethoxy)phenyl)nicotinamide (**18**) white solid, MP: 249 ℃; ^{1}H NMR (600 MHz, DMSO-d_6) δ 10.44 (s, 1H), 8.48 (d, *J* = 2.3 Hz, 1H), 8.31 (d, *J* = 2.3 Hz, 1H), 7.83 (d, *J* = 9.0 Hz, 2H), 7.63 (d, *J* = 8.1 Hz, 2H), 7.38 (d, *J* = 8.6 Hz, 2H), 7.30 (d, *J* = 7.9 Hz, 2H), 7.09 (s, 2H), 2.64 (q, *J* = 7.6 Hz, 2H), 1.21 (t, *J* = 7.6 Hz, 3H). ^{13}C NMR (151 MHz, DMSO-d_6) δ 167.15, 158.37, 150.02, 142.91, 138.58, 135.66, 135.12, 134.72, 128.79(2C), 127.30, 126.23(2C), 124.27, 122.61(2C), 121.91(2C), 28.29, 16.20. HRMS (ESI): *m/z*[M+H]$^+$ calcd for C21H18N3O2F3: 402.1424; found: 402.1417.

2-amino-N,5-bis(4-ethylphenyl)nicotinamide (**19**) white solid, MP: 290 ℃; ^{1}H NMR (600 MHz, DMSO-d_6) δ 10.22 (s, 1H), 8.46 (d, *J* = 2.3 Hz, 1H), 8.29 (d, *J* = 2.4 Hz, 1H), 7.63 (d, *J* = 8.4 Hz, 2H), 7.61 (d, *J* = 8.2 Hz, 2H), 7.30 (d, *J* = 7.9 Hz, 2H), 7.20 (d, *J* = 8.2 Hz, 2H), 7.08 (s, 2H), 2.64 (q, *J* = 7.7 Hz, 2H), 2.59 (q, *J* = 7.6 Hz, 2H), 1.21 (t, *J* = 6.7 Hz, 3H), 1.18 (t, *J* = 6.4 Hz, 3H). ^{13}C NMR (151 MHz, DMSO-d_6) δ 166.86, 158.40, 149.68, 142.85, 139.77, 136.97, 135.46, 135.22, 134.72, 128.78(2C), 128.25(2C), 127.30, 126.24(2C), 124.25, 121.43(2C), 110.38, 28.29, 28.13, 16.21, 16.19. HRMS (ESI): *m/z*[M+H]$^+$ calcd for C22H23N3O: 346.1914; found: 346.1927.

2-amino-5-(4-ethylphenyl)-N-(4-methoxyphenyl)nicotinamide (**20**) white solid, MP:

210 ℃; ^{1}H NMR (600 MHz, DMSO-d_6) δ 10.18 (s, 1H), 8.46 (d, *J* = 2.3 Hz, 1H), 8.29 (d, *J* = 2.4 Hz, 1H), 7.63 (d, *J* = 7.9 Hz, 2H), 7.60 (d, *J* = 8.9 Hz, 2H), 7.30 (d, *J* = 7.9 Hz, 2H), 7.08 (s, 2H), 6.94 (d, *J* = 9.0 Hz, 2H), 3.75 (s, 3H), 2.64 (q, *J* = 7.6 Hz, 2H), 1.20 (t, *J* = 7.6 Hz, 3H). ^{13}C NMR (151 MHz, DMSO-d_6) δ 166.66, 158.38, 156.20, 149.59, 142.84, 135.36, 135.22, 132.27, 128.78(2C), 126.22(2C), 124.25, 123.02(2C), 114.20(2C), 110.39, 55.66, 28.29, 16.21. HRMS (ESI): *m/z*[M+H]$^+$ calcd for C21H21N3O2: 348.1707; found: 348.1712.

2-amino-5-(4-ethylphenyl)-N-(4-isopropylphenyl)nicotinamide (**21**) white solid, MP: 257 ℃; ^{1}H NMR (600 MHz, DMSO-d_6) δ 10.22 (s, 1H), 8.46 (d, *J* = 2.3 Hz, 1H), 8.29 (d, *J* = 2.4 Hz, 1H), 7.64 – 7.60 (m, 4H), 7.30 (d, *J* = 7.8 Hz, 2H), 7.23 (d, *J* = 8.4 Hz, 2H), 7.08 (s, 2H), 2.87 (p, *J* = 6.9 Hz, 1H), 2.64 (q, *J* = 7.6 Hz, 2H), 1.21 (m, 9H). ^{13}C NMR (151 MHz, DMSO-d_6) δ 165.80, 157.33, 148.62, 143.37, 141.79, 135.97, 134.40, 134.15, 127.71(2C), 125.69(2C), 125.17(2C), 123.19, 120.35(2C), 109.32, 32.33, 27.23, 23.37(2C), 15.14. HRMS (ESI): *m/z*[M+H]$^+$ calcd for C23H25N3O: 360.2070; found: 360.2073.

2-amino-N-(4-carbamoylphenyl)-5-(4-ethylphenyl)nicotinamide (**22**) white solid, MP: 224 ℃; ^{1}H NMR (600 MHz, DMSO-d_6) δ 10.45 (s, 1H), 8.49 (d, *J* = 2.3 Hz, 1H), 8.32 (d, *J* = 2.3 Hz, 1H), 7.90 (d, *J* = 8.7 Hz, 3H), 7.80 (d, *J* = 8.4 Hz, 2H), 7.64 (d, *J* = 7.7 Hz, 2H), 7.30 (d, *J* = 7.9 Hz, 3H), 7.10 (s, 2H), 2.64 (q, *J* = 7.6 Hz, 2H), 1.21 (t, *J* = 7.6 Hz, 3H). ^{13}C NMR (151 MHz, DMSO-d_6) δ 167.86, 167.23, 158.40, 150.05, 142.91, 142.07, 135.72, 135.13, 129.73, 128.80(2C), 128.66(2C), 126.25(2C), 124.27, 120.19(2C),

110.08, 60.22, 28.30, 16.21. HRMS (ESI): *m/z*[M+H]$^+$ calcd for C21H20N4O2: 361.1659; found: 361.1665.

N-(4-acetamidophenyl)-4'-ethyl-[1,1'-biphenyl]-3-carboxamide (**23**) white solid, MP: 219 ℃; ^{1}H NMR (600 MHz, DMSO-d_6) δ 10.28 (s, 1H), 9.93 (s, 1H), 8.20 (s, 1H), 7.91 (d, *J* = 7.6 Hz, 1H), 7.85 (d, *J* = 7.8 Hz, 1H), 7.70 (t, *J* = 9.1 Hz, 4H), 7.63 (m, 3H), 7.35 (d, *J* = 7.9 Hz, 2H), 2.67 (q, *J* = 7.6 Hz, 2H), 2.04 (s, 3H), 1.22 (t, *J* = 7.6 Hz, 3H). ^{13}C NMR (151 MHz, DMSO-d_6) δ 168.48, 165.62, 144.00, 140.71, 137.41, 136.09, 135.78, 134.76, 133.55, 132.87, 132.51, 132.49, 131.99, 131.92, 129.89, 129.51, 129.26, 129.19, 128.90(2C), 127.31(2C), 126.94, 126.00, 121.37(2C), 119.65(2C), 28.30, 24.40, 16.08. HRMS (ESI): *m/z*[M+H]$^+$ calcd for C23H22N2O2: 359.1754; found: 359.1760.

N-(4-acetamidophenyl)-2-(4-ethylphenyl)isonicotinamide (**24**) white solid, MP: 182 ℃; ^{1}H NMR (600 MHz, DMSO-d_6) δ 10.51 (s, 1H), 9.96 (s, 1H), 8.83 (d, *J* = 5.0 Hz, 1H), 8.36 (d, *J* = 1.6 Hz, 1H), 8.11 (d, *J* = 8.1 Hz, 2H), 7.77 (d, *J* = 3.4 Hz, 1H), 7.71 (d, *J* = 8.8 Hz, 2H), 7.60 (d, *J* = 8.9 Hz, 2H), 7.38 (d, *J* = 8.0 Hz, 2H), 2.69 (q, *J* = 7.6 Hz, 2H), 2.05 (s, 3H), 1.23 (t, *J* = 7.6 Hz, 3H). ^{13}C NMR (151 MHz, DMSO-d_6) δ 168.56, 164.18, 157.27, 150.64, 145.81, 143.59, 136.22, 134.18, 131.99, 131.93, 129.27, 129.19, 128.74(2C), 127.23(2C), 121.52(2C), 120.57, 119.68(2C), 117.99, 28.41, 24.41, 15.91. HRMS (ESI): *m/z*[M+H]$^+$ calcd for C22H21N3O2: 360.1707; found: 360.1715.

N-(4-acetamidophenyl)-5-(4-ethylphenyl)nicotinamide (**25**) white solid, MP: 194 ℃; ^{1}H NMR (600 MHz, DMSO-d_6) δ 10.44 (s, 1H), 9.94 (s, 1H), 9.05 (d, *J* = 5.0 Hz, 2H), 8.52 (s, 1H), 7.76 (d, *J* = 7.8 Hz, 2H), 7.70 (d, *J* = 8.6 Hz, 2H), 7.58 (d, *J* = 8.5 Hz, 2H),

7.39 (d, J = 7.8 Hz, 2H), 2.68 (q, J = 7.6 Hz, 2H), 2.04 (s, 3H), 1.23 (t, J = 7.6 Hz, 3H). ^{13}C NMR (151 MHz, DMSO-d_6) δ 168.53, 164.11, 150.21, 147.81, 144.83, 136.03, 135.53, 134.41, 134.21, 133.24, 131.06, 129.13(2C), 127.53(2C), 121.35(2C), 119.68(2C), 28.33, 24.40, 16.04. HRMS (ESI): *m/z*[M+H]$^+$ calcd for C22H21N3O2: 360.1707; found: 360.1698.

N-(4-acetamidophenyl)-4-(4-ethylphenyl)picolinamide (**26**) white solid, MP: 237 ℃; ^{1}H NMR (600 MHz, DMSO-d_6) δ 10.62 (s, 1H), 9.95 (s, 1H), 8.76 (d, J = 5.1 Hz, 1H), 8.39 (d, J = 1.9 Hz, 1H), 7.97 (dd, J = 5.1, 1.9 Hz, 1H), 7.86 (d, J = 8.8 Hz, 2H), 7.82 (d, J = 8.2 Hz, 2H), 7.58 (d, J = 8.9 Hz, 2H), 7.41 (d, J = 7.9 Hz, 2H), 2.69 (q, J = 7.6 Hz, 2H), 2.05 (s, 3H), 1.23 (t, J = 7.6 Hz, 3H). ^{13}C NMR (151 MHz, DMSO-d_6) δ 168.51, 162.62, 151.26, 149.56, 149.29, 146.21, 135.96, 134.35, 134.02, 132.51, 132.49, 131.99, 131.92, 129.28, 129.18(2C), 127.42(2C), 124.18, 121.10(2C), 119.72(2C), 119.55, 28.37, 24.40, 15.87. HRMS (ESI): *m/z*[M+H]$^+$ calcd for C22H21N3O2: 360.1707; found: 360.1713.

N-(4-acetamidophenyl)-6-amino-5-(4-ethylphenyl)nicotinamide (**27**) white solid, MP: 185 ℃; ^{1}H NMR (600 MHz, DMSO-d_6) δ 9.90 (s, 1H), 9.88 (s, 1H), 8.60 (d, J = 2.3 Hz, 1H), 7.91 (d, J = 2.4 Hz, 1H), 7.64 (d, J = 9.0 Hz, 2H), 7.52 (d, J = 9.0 Hz, 2H), 7.42 (d, J = 8.1 Hz, 2H), 7.35 (d, J = 7.9 Hz, 2H), 6.26 (s, 2H), 2.67 (q, J = 7.6 Hz, 2H), 2.03 (s, 3H). ^{13}C NMR (151 MHz, DMSO-d_6) δ 168.42, 164.25, 159.03, 148.35, 143.71, 136.92, 135.40, 135.27, 135.01, 128.96(2C), 128.89(2C), 121.13(2C), 119.73, 119.63(2C), 119.60, 28.40, 24.37, 16.02. HRMS (ESI): *m/z*[M+H]$^+$ calcd for C22H22N4O2:

375.1816; found: 375.1820.

N-(4-acetamidophenyl)-2-amino-5-(*p*-tolyl)nicotinamide (**28**) A white solid, MP: 267 ℃; ^{1}H NMR (400 MHz, DMSO-d_6) δ 10.26 (s, 1H), 9.96 (s, 1H), 8.46 (d, *J* = 2.3 Hz, 1H), 8.29 (d, *J* = 2.4 Hz, 1H), 7.66 – 7.54 (m, 6H), 7.27 (d, *J* = 7.9 Hz, 2H), 7.09 (s, 2H), 2.34 (s, 3H), 2.04 (s, 3H). ^{13}C NMR (101 MHz, DMSO-d_6) δ 168.53, 166.74, 158.36, 149.62, 136.43, 135.89, 135.33, 134.85, 134.38, 129.96(2C), 126.07(2C), 124.12, 121.82(2C), 119.56(2C), 110.33, 24.41, 21.14. HRMS (ESI): *m/z*[M+H]$^+$ calcd for C21H20N4O2: 361.1659; found: 361.1646.

N-(4-acetamidophenyl)-2-amino-5-(*m*-tolyl)nicotinamide (**29**) A white solid, MP: 256 ℃; ^{1}H NMR (400 MHz, DMSO-d_6) δ 10.26 (s, 1H), 9.96 (s, 1H), 8.47 (d, *J* = 2.3 Hz, 1H), 8.31 (d, *J* = 2.4 Hz, 1H), 7.62 (d, *J* = 9.2 Hz, 2H), 7.59 – 7.49 (m, 4H), 7.35 (t, *J* = 7.6 Hz, 1H), 7.14 (m, 3H), 2.38 (s, 3H), 2.04 (s, 3H). ^{13}C NMR (101 MHz, DMSO-d_6) δ 168.52, 166.73, 158.50, 149.83, 138.53, 137.68, 135.90, 135.59, 134.37, 132.01, 129.28, 127.88, 126.82, 124.23, 123.38, 121.85(2C), 119.56(2C), 110.33, 24.41, 21.64. HRMS (ESI): *m/z*[M+H]$^+$ calcd for C21H20N4O2: 361.1659; found: 361.1646.

N-(4-acetamidophenyl)-2-amino-5-(4-(methylsulfonyl)phenyl)nicotinamide (**30**) A white solid, MP: 256 ℃; ^{1}H NMR (400 MHz, DMSO-d_6) δ 10.30 (s, 1H), 9.96 (s, 1H), 8.61 (d, *J* = 2.4 Hz, 1H), 8.43 (d, *J* = 2.4 Hz, 1H), 8.03 (d, *J* = 8.9 Hz, 2H), 7.99 (d, *J* = 8.8 Hz, 2H), 7.61 (d, *J* = 9.5 Hz, 2H), 7.58 (d, *J* = 9.4 Hz, 2H), 7.32 (s, 2H), 3.26 (s, 3H), 2.04 (s, 3H). ^{13}C NMR (101 MHz, DMSO-d_6) δ 168.54, 166.48, 159.18, 150.49, 142.86, 139.08, 136.12, 135.99, 134.26, 128.09(2C), 126.67(2C), 122.09, 121.86(2C),

119.59(2C), 110.39, 44.12, 24.42. HRMS (ESI): *m/z*$[M+H]^+$ calcd for C21H20N4O4S: 425.1278 ; found: 425.1274.

N-(4-acetamidophenyl)-2-amino-5-(4-methoxyphenyl)nicotinamide (**31**) A white solid, MP: 260 ℃; ^{1}H NMR (400 MHz, DMSO-d_6) δ 10.24 (s, 1H), 9.95 (s, 1H), 8.43 (d, *J* = 2.4 Hz, 1H), 8.26 (d, *J* = 2.4 Hz, 1H), 7.65 (d, *J* = 8.8 Hz, 2H), 7.61 (d, *J* = 9.1 Hz, 2H), 7.56 (d, *J* = 9.1 Hz, 2H), 7.11 – 7.01 (m, 4H), 3.79 (s, 3H), 2.04 (s, 3H). ^{13}C NMR (101 MHz, DMSO-d_6) δ 168.54, 166.77, 158.88, 158.09, 149.43, 135.87, 135.16, 134.40, 130.20, 127.43(2C), 124.08, 121.79(2C), 119.57(2C), 114.82(2C), 110.32, 55.64, 24.40. HRMS (ESI): *m/z*$[M+H]^+$ calcd for C21H20N4O3: 377.1608; found: 377.1634.

N-(4-acetamidophenyl)-2-amino-5-(3-methoxyphenyl)nicotinamide (**32**) A white solid, MP: 269 ℃; ^{1}H NMR (400 MHz, DMSO-d_6) δ 10.26 (s, 1H), 9.96 (s, 1H), 8.50 (d, *J* = 2.4 Hz, 1H), 8.32 (d, *J* = 2.4 Hz, 1H), 7.62 (d, *J* = 9.1 Hz, 2H), 7.57 (d, *J* = 9.1 Hz, 2H), 7.38 (t, *J* = 8.0 Hz, 1H), 7.29 (dd, *J* = 7.4, 1.5 Hz, 2H), 7.14 (s, 2H), 6.93 – 6.88 (m, 1H), 3.83 (s, 3H), 2.04 (s, 3H). ^{13}C NMR (101 MHz, Chloroform-*d*) δ 173.30, 171.45, 165.07, 163.38, 154.73, 143.98, 140.65, 140.47, 139.13, 135.20, 128.75, 126.60(2C), 124.33(2C), 123.34, 117.38, 116.68, 115.01, 60.40, 29.16. HRMS (ESI): *m/z*$[M+H]^+$ calcd for C21H20N4O3: 377.1608; found: 377.1625.

N-(4-acetamidophenyl)-2-amino-5-(2-methoxyphenyl)nicotinamide (**33**) A white solid, MP: 235 ℃; ^{1}H NMR (400 MHz, DMSO-d_6) δ 10.15 (s, 1H), 9.94 (s, 1H), 8.29 (d, *J* = 2.2 Hz, 1H), 8.14 (d, *J* = 2.3 Hz, 1H), 7.61 (d, *J* = 9.0 Hz, 2H), 7.55 (d, *J* = 9.1 Hz, 2H), 7.41 (dd, *J* = 7.5, 1.7 Hz, 1H), 7.34 (ddd, *J* = 8.3, 7.4, 1.8 Hz, 1H), 7.12 (dd, *J* = 8.4,

1.1 Hz, 1H), 7.05 (s, 1H), 3.79 (s, 3H), 2.04 (s, 3H). ^{13}C NMR (101 MHz, DMSO-d_6) δ 168.52, 166.80, 158.00, 156.65, 152.11, 137.90, 135.78, 134.49, 130.38, 129.09, 127.00, 121.99, 121.59(2C), 121.33, 119.58(2C), 112.09, 109.93, 56.02, 24.39. HRMS (ESI): *m/z*[M+H]$^+$ calcd for C21H20N4O3: 377.1608; found: 377.1617.

N-(4-acetamidophenyl)-2-amino-5-(2,3,4-trimethoxyphenyl)nicotinamide (**34**) A white solid, MP: 130 ℃; ^{1}H NMR (400 MHz, DMSO-d_6) δ 10.17 (s, 1H), 9.94 (s, 1H), 8.26 (d, *J* = 2.2 Hz, 1H), 8.11 (d, *J* = 2.3 Hz, 1H), 7.60 (d, *J* = 9.1 Hz, 2H), 7.54 (d, *J* = 9.1 Hz, 2H), 7.16 (d, *J* = 8.6 Hz, 1H), 7.04 (s, 2H), 6.91 (d, *J* = 8.8 Hz, 1H), 3.83 (s, 3H), 3.79 (s, 3H), 3.65 (s, 3H), 2.03 (s, 3H). ^{13}C NMR (101 MHz, DMSO-d_6) δ 168.53, 166.78, 157.95, 153.27, 151.61, 151.11, 142.61, 137.55, 135.79, 134.47, 124.92, 124.57, 121.70, 121.62, 119.58(2C), 110.00, 108.822C(), 61.13, 60.96, 56.42, 24.39. HRMS (ESI): *m/z*[M+H]$^+$ calcd for C23H24N4O5: 437.1819; found: 437.1826.

N-(4-acetamidophenyl)-2-amino-5-(4-chlorophenyl)nicotinamide (**35**) A white solid, MP: 279 ℃; ^{1}H NMR (400 MHz, DMSO-d_6) δ 10.26 (s, 1H), 9.96 (s, 1H), 8.50 (d, *J* = 2.4 Hz, 1H), 8.33 (d, *J* = 2.4 Hz, 1H), 7.77 (d, *J* = 8.7 Hz, 2H), 7.61 (d, *J* = 9.2 Hz, 2H), 7.57 (d, *J* = 9.2 Hz, 2H), 7.52 (d, *J* = 8.6 Hz, 2H), 7.18 (s, 2H), 2.04 (s, 3H). ^{13}C NMR (101 MHz, DMSO-d_6) δ 168.55, 166.60, 158.68, 149.87, 136.64, 135.92, 135.57, 134.32, 131.93, 129.31(2C), 127.90(2C), 122.75, 121.82(2C), 119.58(2C), 110.30, 24.41. HRMS (ESI): *m/z*[M+H]$^+$ calcd for C20H17ClN4O2: 381.1130; found: 381.1109.

N-(4-acetamidophenyl)-2-amino-5-(3-chlorophenyl)nicotinamide (**36**) A white solid, MP: 281 ℃; ^{1}H NMR (400 MHz, DMSO-d_6) δ 10.25 (s, 1H), 9.96 (s, 1H), 8.53 (d, *J* =

2.3 Hz, 1H), 8.35 (d, *J* = 2.4 Hz, 1H), 7.84 (t, *J* = 1.9 Hz, 1H), 7.71 (d, *J* = 7.8 Hz, 1H), 7.61 (d, *J* = 9.2 Hz, 2H), 7.57 (d, *J* = 9.2 Hz, 2H), 7.48 (t, *J* = 7.9 Hz, 1H), 7.38 (d, *J* = 9.1 Hz, 1H), 7.22 (s, 2H), 2.04 (s, 3H). ^{13}C NMR (101 MHz, DMSO-d_6) δ 168.55, 166.55, 158.89, 150.11, 140.00, 135.96, 135.74, 134.27, 131.18, 126.95, 125.81, 124.75, 122.49, 121.94(2C), 119.57(2C), 110.20, 24.41. HRMS (ESI): *m/z*[M+H]$^+$ calcd for C20H17ClN4O2: 381.1130; found: 381.1107.

N-(4-acetamidophenyl)-2-amino-5-(2-chlorophenyl)nicotinamide (**37**) A white solid, MP: 231 ℃; ^{1}H NMR (400 MHz, DMSO-d_6) δ 10.15 (s, 1H), 9.94 (s, 1H), 8.26 (d, *J* = 2.3 Hz, 1H), 8.17 (d, *J* = 2.3 Hz, 1H), 7.62 – 7.58 (m, 3H), 7.55 (td, *J* = 4.6, 3.8, 2.3 Hz, 3H), 7.46 (td, *J* = 7.5, 1.5 Hz, 1H), 7.40 (td, *J* = 7.6, 1.8 Hz, 1H), 7.23 (s, 1H), 2.03 (s, 3H). ^{13}C NMR (101 MHz, DMSO-d_6) δ 168.50, 166.46, 158.54, 151.96, 138.18, 137.03, 135.85, 134.36, 132.09, 131.99, 130.36, 129.53, 128.14, 122.49, 121.64(2C), 119.56(2C), 109.60, 24.40. HRMS (ESI): *m/z*[M+H]$^+$ calcd for C20H17ClN4O2: 381.1130; found: 381.1105.

N-(4-acetamidophenyl)-2-amino-5-(4-fluorophenyl)nicotinamide (**38**) A white solid, MP: 271 ℃; ^{1}H NMR (400 MHz, DMSO-d_6) δ 10.25 (s, 1H), 9.96 (s, 1H), 8.47 (d, *J* = 2.4 Hz, 1H), 8.30 (d, *J* = 2.5 Hz, 1H), 7.78 (d, *J* = 5.4 Hz, 1H), 7.76 (d, *J* = 5.6 Hz, 1H), 7.61 (d, *J* = 9.1 Hz, 2H), 7.57 (d, *J* = 9.1 Hz, 2H), 7.30 (t, *J* = 8.9 Hz, 2H), 7.13 (s, 2H), 2.04 (s, 3H). ^{13}C NMR (101 MHz, DMSO-d_6) δ 168.55, 166.66, 158.47, 149.81, 135.91, 135.63, 134.35, 128.20 (2C, d, *J* = 8.0 Hz), 123.22, 121.80(2C), 119.58(2C), 116.16 (2C,

d, J = 21.3 Hz), 110.28, 24.40. HRMS (ESI): m/z[M+H]$^+$ calcd for C20H17FN4O2: 365.1408; found: 365.1418.

N-(4-acetamidophenyl)-2-amino-5-(3-fluorophenyl)nicotinamide (**39**) A white solid, MP: 283 ℃; ^{1}H NMR (400 MHz, DMSO-d_6) δ 10.21 (s, 1H), 9.95 (s, 1H), 8.36 (t, J = 2.3 Hz, 1H), 8.23 (d, J = 2.4 Hz, 1H), 7.65 (td, J = 7.8, 1.7 Hz, 1H), 7.60 (d, J = 9.1 Hz, 2H), 7.55 (d, J = 9.1 Hz, 2H), 7.44 – 7.30 (m, 3H), 7.21 (s, 2H), 2.04 (s, 3H). ^{13}C NMR (101 MHz, DMSO-d_6) δ 168.53, 166.50, 158.55, 151.60 (d, J = 5.0 Hz), 137.55, 135.88, 134.34, 130.70 (d, J = 3.5 Hz), 129.53 (d, J = 8.4 Hz), 125.71 (d, J = 13.4 Hz), 125.47 (d, J = 3.7 Hz), 121.69(2C), 119.58(2C), 118.97, 116.63, 116.41, 110.28, 24.40. HRMS (ESI): m/z[M+H]$^+$ calcd for C20H17FN4O2: 365.1408; found: 365.1538.

N-(4-acetamidophenyl)-2-amino-5-(4-(trifluoromethyl)phenyl)nicotinamide (**40**) A white solid, MP: 298 ℃; ^{1}H NMR (400 MHz, DMSO-d_6) δ 10.29 (s, 1H), 9.97 (s, 1H), 8.58 (d, J = 2.4 Hz, 1H), 8.41 (d, J = 2.4 Hz, 1H), 7.98 (d, J = 8.2 Hz, 2H), 7.81 (d, J = 7.9 Hz, 2H), 7.61 (d, J = 9.2 Hz, 2H), 7.57 (d, J = 9.3 Hz, 2H), 7.27 (s, 2H), 2.04 (s, 3H). ^{13}C NMR (101 MHz, DMSO-d_6) δ 168.56, 166.52, 159.06, 150.31, 141.87, 135.98 (d, J = 3.4 Hz), 134.29, 126.69(2C), 126.21 (d, J = 4.0 Hz), 122.34, 121.84(2C), 119.59(2C), 110.36, 24.40. HRMS (ESI): m/z[M+H]$^+$ calcd for C21H17F3N4O2: 415.1376; found: 415.1377.

N-(4-acetamidophenyl)-2-amino-5-(3-(trifluoromethyl)phenyl)nicotinamide (**41**) A white solid, MP: 301 ℃; ^{1}H NMR (400 MHz, DMSO-d_6) δ 10.27 (s, 1H), 9.97 (s, 1H), 8.58 (d, J = 2.3 Hz, 1H), 8.39 (d, J = 2.4 Hz, 1H), 8.08 (s, 1H), 8.05 (d, J = 6.4 Hz, 1H),

7.74 – 7.66 (m, 2H), 7.61 (d, *J* = 9.2 Hz, 2H), 7.58 (d, *J* = 9.2 Hz, 2H), 7.23 (s, 2H), 2.04 (s, 3H). ^{13}C NMR (101 MHz, DMSO-d_6) δ 168.56, 166.57, 158.93, 150.24, 138.94, 135.97, 134.27, 130.43, 130.19, 126.14, 123.68 (d, *J* = 4.0 Hz), 123.43, 122.60 (d, *J* = 3.8 Hz), 121.93(2C), 119.59(2C), 110.33, 24.40. HRMS (ESI): *m/z*[M+H]$^+$ calcd for C21H17F3N4O2: 415.1376; found: 415.1393.

N-(4-acetamidophenyl)-2-amino-5-(2-(trifluoromethyl)phenyl)nicotinamide (**42**) A white solid, MP: 232 ℃; ^{1}H NMR (400 MHz, DMSO-d_6) δ 10.12 (s, 1H), 9.94 (s, 1H), 8.10 (s, 1H), 8.09 (s, 1H), 7.87 (d, *J* = 6.7 Hz, 1H), 7.77 (t, *J* = 7.0 Hz, 1H), 7.63 (t, *J* = 7.8 Hz, 1H), 7.56 (q, *J* = 9.2 Hz, 5H), 7.26 (s, 2H), 2.03 (s, 3H). ^{13}C NMR (101 MHz, DMSO-d_6) δ 168.53, 166.38, 158.76, 151.01, 137.83, 137.58, 135.88, 134.27, 133.35, 132.99, 128.58, 127.76 (d, *J* = 28.8 Hz), 126.74 (d, *J* = 5.4 Hz), 124.74 (d, *J* = 273.9 Hz), 123.03, 121.77(2C), 119.56(2C), 109.15, 24.39. HRMS (ESI): *m/z*[M+H]$^+$ calcd for C21H17F3N4O2: 415.1376; found: 415.1358.

N-(4-acetamidophenyl)-2-amino-5-(4-hydroxyphenyl)nicotinamide (**43**) A white solid, MP: 270 ℃; ^{1}H NMR (400 MHz, DMSO-d_6) δ 10.23 (s, 1H), 9.95 (s, 1H), 9.52 (s, 1H), 8.39 (d, *J* = 2.3 Hz, 1H), 8.22 (d, *J* = 2.4 Hz, 1H), 7.61 (d, *J* = 9.1 Hz, 2H), 7.56 (d, *J* = 9.2 Hz, 2H), 7.53 (d, *J* = 8.7 Hz, 2H), 6.99 (s, 2H), 6.85 (d, *J* = 8.6 Hz, 2H), 2.04 (s, 3H). ^{13}C NMR (101 MHz, DMSO-d_6) δ 168.54, 166.82, 157.91, 157.04, 149.24, 135.84, 134.97, 134.43, 128.58, 127.45(2C), 124.53, 121.77(2C), 119.57(2C), 116.16(2C), 110.31, 24.40. HRMS (ESI): *m/z*[M+H]$^+$ calcd for C20H18N4O3: 363.1452; found: 363.1471.

N-(4-acetamidophenyl)-2-amino-5-(3-hydroxyphenyl)nicotinamide (**44**) A white solid, MP: 271 ℃; ^{1}H NMR (400 MHz, DMSO-d_6) δ 10.27 (s, 1H), 9.96 (s, 1H), 9.54 (s, 1H), 8.42 (d, *J* = 2.2 Hz, 1H), 8.27 (d, *J* = 2.3 Hz, 1H), 7.61 (d, *J* = 8.8 Hz, 2H), 7.56 (d, *J* = 8.9 Hz, 2H), 7.25 (t, *J* = 7.8 Hz, 1H), 7.19 – 7.04 (m, 4H), 6.74 (dd, *J* = 8.0, 2.2 Hz, 1H), 2.04 (s, 3H). ^{13}C NMR (101 MHz, DMSO-d_6) δ 168.56, 166.72, 158.54, 158.33, 149.73, 139.11, 135.87, 135.54, 134.39, 130.38, 124.28, 121.81(2C), 119.58(2C), 117.05, 114.24, 113.05, 110.23, 24.40. HRMS (ESI): *m/z*[M+H]$^+$ calcd for C20H18N4O3: 363.1452; found: 363.1450.

N-(4-acetamidophenyl)-2-amino-5-(2-hydroxyphenyl)nicotinamide (**45**) A white solid, MP: 265 ℃; ^{1}H NMR (400 MHz, DMSO-d_6) δ 10.17 (s, 1H), 9.94 (s, 1H), 9.62 (s, 1H), 8.37 (d, *J* = 2.2 Hz, 1H), 8.20 (d, *J* = 2.3 Hz, 1H), 7.61 (d, *J* = 9.1 Hz, 2H), 7.54 (d, *J* = 9.1 Hz, 2H), 7.37 (dd, *J* = 7.6, 1.7 Hz, 1H), 7.15 (ddd, *J* = 8.2, 7.3, 1.7 Hz, 1H), 7.01 (s, 2H), 6.97 – 6.93 (m, 1H), 6.92 – 6.86 (m, 1H), 2.04 (s, 3H). ^{13}C NMR (101 MHz, DMSO-d_6) δ 168.52, 166.90, 157.82, 154.75, 151.98, 137.74, 135.75, 134.52, 130.21, 128.68, 125.03, 122.47, 121.57(2C), 120.00, 119.58(2C), 116.34, 109.91, 24.39. HRMS (ESI): *m/z*[M+H]$^+$ calcd for C20H18N4O3: 363.1452; found: 363.1449.

N-(4-acetamidophenyl)-2-amino-5-(4-cyanophenyl)nicotinamide (**46**) A white solid, MP: 270 ℃; ^{1}H NMR (400 MHz, DMSO-d_6) δ 10.28 (s, 1H), 9.96 (s, 1H), 8.61 (d, *J* = 2.4 Hz, 1H), 8.42 (d, *J* = 2.5 Hz, 1H), 7.97 (d, *J* = 8.2 Hz, 2H), 7.92 (d, *J* = 8.3 Hz, 2H), 7.64 – 7.51 (m, 4H), 7.33 (s, 2H), 2.04 (s, 3H). ^{13}C NMR (101 MHz, DMSO-d_6) δ 168.56, 166.44, 159.20, 150.48, 142.41, 135.98, 134.26, 133.29(2C), 126.65(2C), 121.93,

121.86(2C), 119.59(2C), 110.33, 109.39, 24.41. HRMS (ESI): *m/z*[M+H]$^+$ calcd for C21H17N5O2: 372.1455; found: 372.1489.

N-(4-acetamidophenyl)-2-amino-5-(3-cyanophenyl)nicotinamide (**47**) A white solid, MP: 260 ℃; ^{1}H NMR (400 MHz, DMSO-d_6) δ 10.24 (s, 1H), 9.97 (s, 1H), 8.58 (d, *J* = 2.4 Hz, 1H), 8.41 (s, 1H), 8.26 (s, 1H), 8.14 – 8.07 (m, 1H), 7.78 (dt, *J* = 7.7, 1.3 Hz, 1H), 7.66 (t, *J* = 7.8 Hz, 1H), 7.61 (d, *J* = 9.2 Hz, 2H), 7.58 (d, *J* = 9.3 Hz, 2H), 7.26 (s, 2H), 2.04 (s, 3H). ^{13}C NMR (101 MHz, DMSO-d_6) δ 168.56, 166.49, 159.01, 150.22, 139.01, 135.98, 135.85, 134.24, 130.76, 130.63, 130.58, 129.53, 121.93(2C), 121.83, 119.58(2C), 119.42, 112.50, 110.25, 24.41. HRMS (ESI): *m/z*[M+H]$^+$ calcd for C21H17N5O2: 372.1455; found: 372.1483.

N-(4-acetamidophenyl)-2-amino-5-(2-cyanophenyl)nicotinamide (**48**) A white solid, MP: 219 ℃; ^{1}H NMR (400 MHz, DMSO-d_6) δ 10.21 (s, 1H), 9.96 (s, 1H), 8.41 (d, *J* = 2.4 Hz, 1H), 8.28 (d, *J* = 2.4 Hz, 1H), 7.96 (dd, *J* = 7.8, 1.3 Hz, 1H), 7.81 (td, *J* = 7.6, 1.4 Hz, 1H), 7.76 (d, *J* = 6.7 Hz, 1H), 7.62 – 7.53 (m, 5H), 7.34 (s, 2H), 2.04 (s, 3H). ^{13}C NMR (101 MHz, DMSO-d_6) δ 168.55, 166.29, 159.06, 151.37, 141.96, 137.83, 135.92, 134.29, 134.11, 130.26, 128.29, 121.66, 121.62(2C), 119.60(2C), 119.24, 110.21, 110.06, 49.07, 24.40. HRMS (ESI): *m/z*[M+H]$^+$ calcd for C21H17N5O2: 372.1455; found: 372.1436.

N-(4-acetamidophenyl)-2-amino-5-(3-formyl-5-methylphenyl)nicotinamide (**49**) A white solid, MP: 268 ℃; ^{1}H NMR (400 MHz, DMSO-d_6) δ 10.44 (s, 1H), 10.13 (s, 1H), 10.06 (s, 1H), 8.56 (d, *J* = 2.4 Hz, 1H), 8.46 (s, 1H), 8.13 (s, 1H), 7.98 (s, 1H), 7.69 –

7.57 (m, 5H), 7.21 (s, 2H), 2.47 (s, 3H), 2.05 (s, 3H). ^{13}C NMR (101 MHz, DMSO-d_6) δ 193.82, 168.57, 166.65, 158.86, 149.99, 139.83, 138.66, 137.42, 136.01, 135.86, 134.32, 132.86, 128.07, 125.11, 122.82, 121.94(2C), 119.50(2C), 110.37, 24.40, 21.32. HRMS (ESI): *m/z*[M+H]$^+$ calcd for C22H20N4O3: 389.1608; found: 389.1582.

N-(4-acetamidophenyl)-2-amino-5-(4-fluoro-3-methylphenyl)nicotinamide (**50**) A white solid, MP: 270 ℃; ^{1}H NMR (400 MHz, DMSO-d_6) δ 10.24 (s, 1H), 9.95 (s, 1H), 8.45 (d, *J* = 2.4 Hz, 1H), 8.28 (d, *J* = 2.4 Hz, 1H), 7.65 (dd, *J* = 7.2, 2.2 Hz, 1H), 7.61 (d, *J* = 9.1 Hz, 2H), 7.57 (d, *J* = 9.2 Hz, 3H), 7.27 – 7.18 (m, 1H), 7.11 (s, 2H), 2.31 (d, *J* = 1.9 Hz, 3H), 2.04 (s, 3H). ^{13}C NMR (101 MHz, DMSO-d_6) δ 168.52, 166.69, 158.42, 149.76, 135.92, 135.53, 134.35, 129.43 (d, *J* = 5.1 Hz), 125.46 (d, *J* = 8.0 Hz), 125.10 (d, *J* = 17.3 Hz), 123.35, 121.84(2C), 119.56(2C), 115.78 (d, *J* = 22.2 Hz), 110.29, 24.41, 14.79 (d, *J* = 3.1 Hz). HRMS (ESI): *m/z*[M+H]$^+$ calcd for C21H19FN4O2:479.1565; found: 479.1560.

N-(4-acetamidophenyl)-2-amino-5-(4-amino-2-chlorophenyl)nicotinamide (**51**) A white solid, MP: 178 ℃; ^{1}H NMR (400 MHz, DMSO-d_6) δ 10.14 (s, 1H), 9.94 (s, 1H), 8.15 (d, *J* = 2.3 Hz, 1H), 8.05 (d, *J* = 2.4 Hz, 1H), 7.60 (d, *J* = 9.1 Hz, 2H), 7.54 (d, *J* = 9.1 Hz, 2H), 7.16 (d, *J* = 8.2 Hz, 1H), 7.06 (s, 2H), 6.73 (d, *J* = 2.3 Hz, 1H), 6.61 (dd, *J* = 8.3, 2.3 Hz, 1H), 5.51 (s, 2H), 2.03 (s, 3H). ^{13}C NMR (101 MHz, DMSO-d_6) δ 168.49, 166.66, 157.85, 151.96, 149.94, 137.99, 135.79, 134.45, 132.24, 132.22, 123.62, 123.38, 121.62(2C), 119.55(2C), 114.36, 113.56, 109.63, 24.40. HRMS (ESI): *m/z*[M+H]$^+$ calcd for C20H18ClN5O2: 396.1222; found: 396.1217.

N-(4-acetamidophenyl)-2-amino-5-(2-fluoro-4-methoxyphenyl)nicotinamide (**52**) A white solid, MP: 248 ℃; ^{1}H NMR (400 MHz, DMSO-d_6) δ 10.20 (s, 1H), 9.95 (s, 1H), 8.29 (t, J = 2.2 Hz, 1H), 8.16 (d, J = 2.3 Hz, 1H), 7.63 – 7.51 (m, 5H), 7.13 (s, 2H), 6.97 (dd, J = 12.8, 2.5 Hz, 1H), 6.91 (dd, J = 8.6, 2.6 Hz, 1H), 3.81 (s, 3H), 2.04 (s, 3H). ^{13}C NMR (101 MHz, DMSO-d_6) δ 168.52, 166.58, 161.31, 160.28, 160.17, 158.87, 158.20, 151.31, 137.23, 135.87, 134.37, 131.09 (d, J = 5.4 Hz), 121.69(2C), 119.57(2C), 119.08 (d, J = 2.0 Hz), 117.92, 117.78, 111.43 (d, J = 2.9 Hz), 110.31, 102.59, 102.33, 56.20, 24.40. HRMS (ESI): *m/z*$[M+H]^+$ calcd for C21H19FN4O3: 395.1514; found: 395.1561.

4.2. Cell culture

RAW264.7 and HEK293T cells were obtained from the Shanghai Cell Resource Bank. Cells were cultured in DMEM with 10% FBS (*Shanghai Shuangru Biology Science* & *Technology Co.,Ltd*, LONSERA) and 1% penicillin-streptomycin(Beyotime). Cells were cultured within a humidified incubator at 37 ℃ in the presence of 5% CO_2.

4.3. Cell viability assay

RAW 264.7 and HEK293T were seeded into 96-well plates at a density of 1×10^4 cells per well at 37 ℃, 5% CO_2 for 24 h.20 μM compound was added into each well, and then cells were cultured for 24 h. 20 μL MTT (5 mg/mL) was added and incubated for 4 h. After that, all the culture medium was discarded, and DMSO (150 μL) was added to each well and detected using the microplate reader (492 nm, MQX200, Bio-Tek, USA).

4.4. NO assay

RAW264.7 cells were seeded into 48 well plate at a density of 6.5×10^4 cells per well.

After incubated for 24 hours, cells were treated with tested compounds for different concentrations(3.125,6.25,12.5,25 and 50μM) for 1 h, and then treated with 1 μg/mL LPS for further 24 h. The supernatants were collected and NO level was measured using Griess reagent (Beyotime, China).

4.5. NF-κB transcriptional activity assay

HEK293T cells were seeded into 96 well plate at a density of 1×10^5 cells per well. After incubated for 24 hours, cells were transfected with NF-κB reporter plasmid at 100 ng/well using lip2000.Six hours later, the medium was replaced by fresh medium containing tested compounds with different concentrations. Then incubated for another hour, TNF-α was added for a final concentration at 10 ng/mL.12 hours later, the cell lysate was collected and the RLU was measured by microplate reader.

4.6. TNF-α and IL-6 assay

RAW264.7 cells were seeded into 6 well plate at a density of 1.5×10^6 cells per well. After incubated for 24 hours, cells were treated with different concentrations of compound and stimulated by 1 μg/mL LPS after one hour.24 hours later, the supernatant was collected and measured by ELISA kits(MultiScience).

4.7. Reactive oxygen species(ROS) assay

RAW264.7 cells were seeded into 6 well plate at a density of 1.5×10^6 cells per well. After incubated for 24 hours, cells were treated with different concentrations of compound and stimulated by 1 μg/mL LPS after one hour.24 hours later, cells were collected and washed by PBS for three times. Then cells were resuspended by 10 μM DCFH-DA diluted by serum free medium. Cell samples were incubated for 20 min at 37°C,and then the fluorescence intensity measured by microplate reader using E_x λ 488 nm and E_m λ 525

nm.

4.8. Western blotting

RAW264.7 cells were seeded into 6 well plate at a density of 1.5×10^6 per well and incubated at 37 C in 5% CO_2 about 24 h. RAW264.7 cells were pretreated with compound (4, 2, 1 μM) for 1 h, then treated with LPS (1 μg/mL) for 30 min. The cells were lysed in 300 μL RIPA cell lysis buffer and the samples were analyzed by WB. HEK293T cells were seeded into 6 well plate at a density of 1.5×10^6 cells per well and incubated at 37°C in 5% CO_2 about 24 h. HEK293T cells were pretreated with compound (4, 2, 1 μM) for 1 h, then treated with TNF-α (1 μg/mL) for 12 h. The nuclear protein was extracted by Nuclear protein extraction kit(Beyotime).The samples were analyzed by WB. All antibodies were purchased from ZENBIO.

4.9 Establishment of LPS-induced inflammation models in vivo

The 30 male C57BL/6 mice weighing 18-22 g were purchased from Animal Department of Anhui Medical University. After adaptive feeding for a week, mice were randomly divided into five groups on average, including the control group, LPS group, compound 51 (5,10,20 mg/kg) +LPS group. Before received 20 mg/kg LPS by intraperitoneal injection, mice were gavaged for three days of compound **51**. 6 hours after received LPS, the blood and tissues were taken from mice. The levels of IL-6, TNF-α, SOD and MDA in blood were measured.

The ^{1}H NMR, ^{13}C NMR and ESI-HRMS spectra figure of target compounds

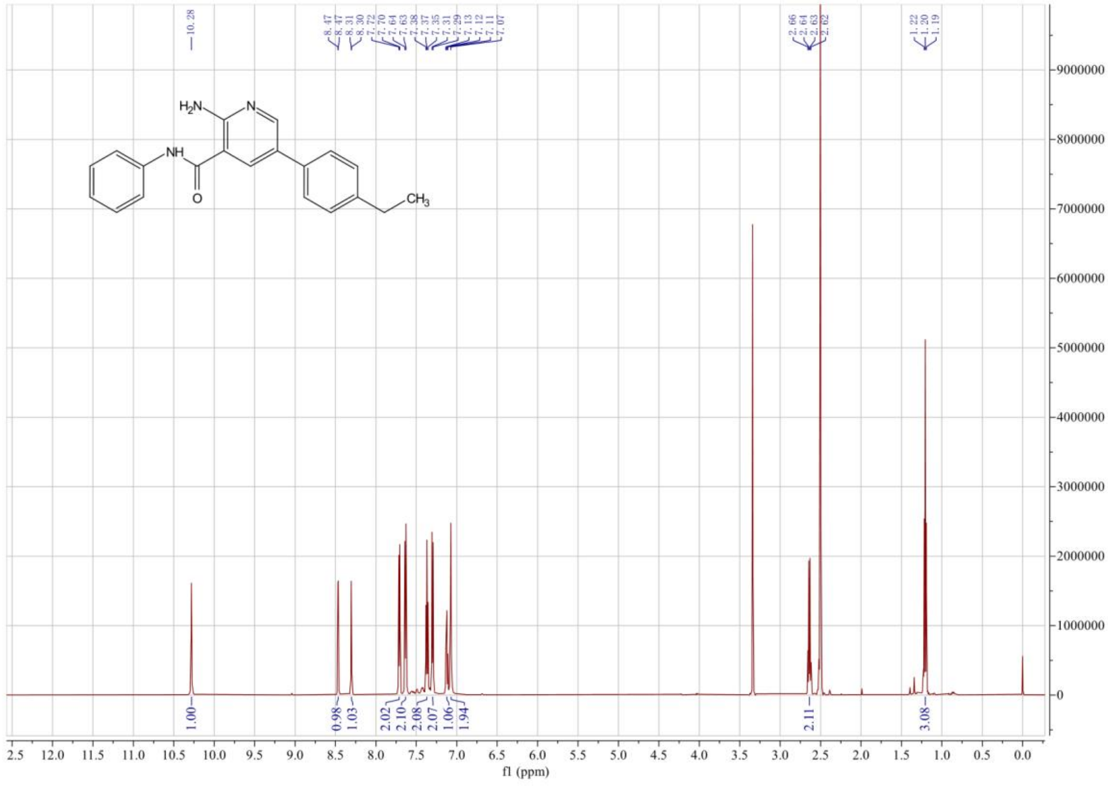
10.28
8.47
8.47
8.31
8.30
7.72
7.70
7.64
7.63
7.38
7.37
7.35
7.31
7.29
7.13
7.11
7.07
2.66
2.64
2.63
2.62
1.22
1.20
1.19
H2N
N
NH
O
CH3
1.00
0.98
1.03
2.02
2.10
2.08
2.07
1.06
1.94
2.11
3.08
f1 (ppm)

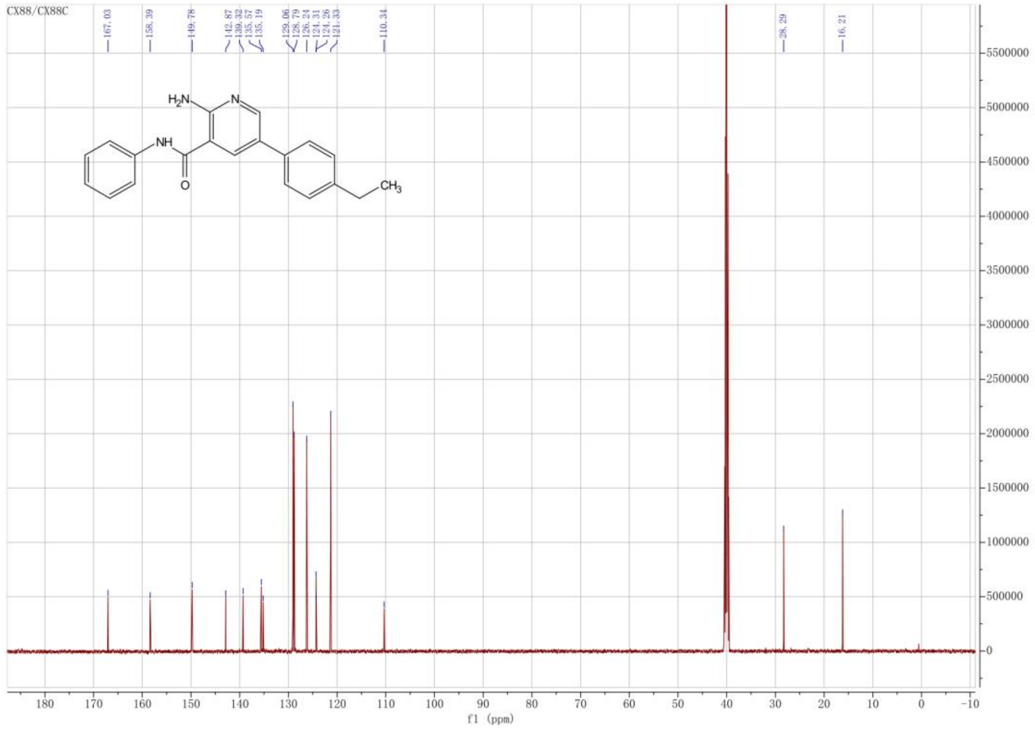
CX88/CX88C
167.03
158.39
149.78
142.87
139.52
135.57
135.19
129.06
128.79
126.24
124.31
121.26
121.33
110.34
28.29
16.21
H2N
N
NH
O
CH3
f1 (ppm)

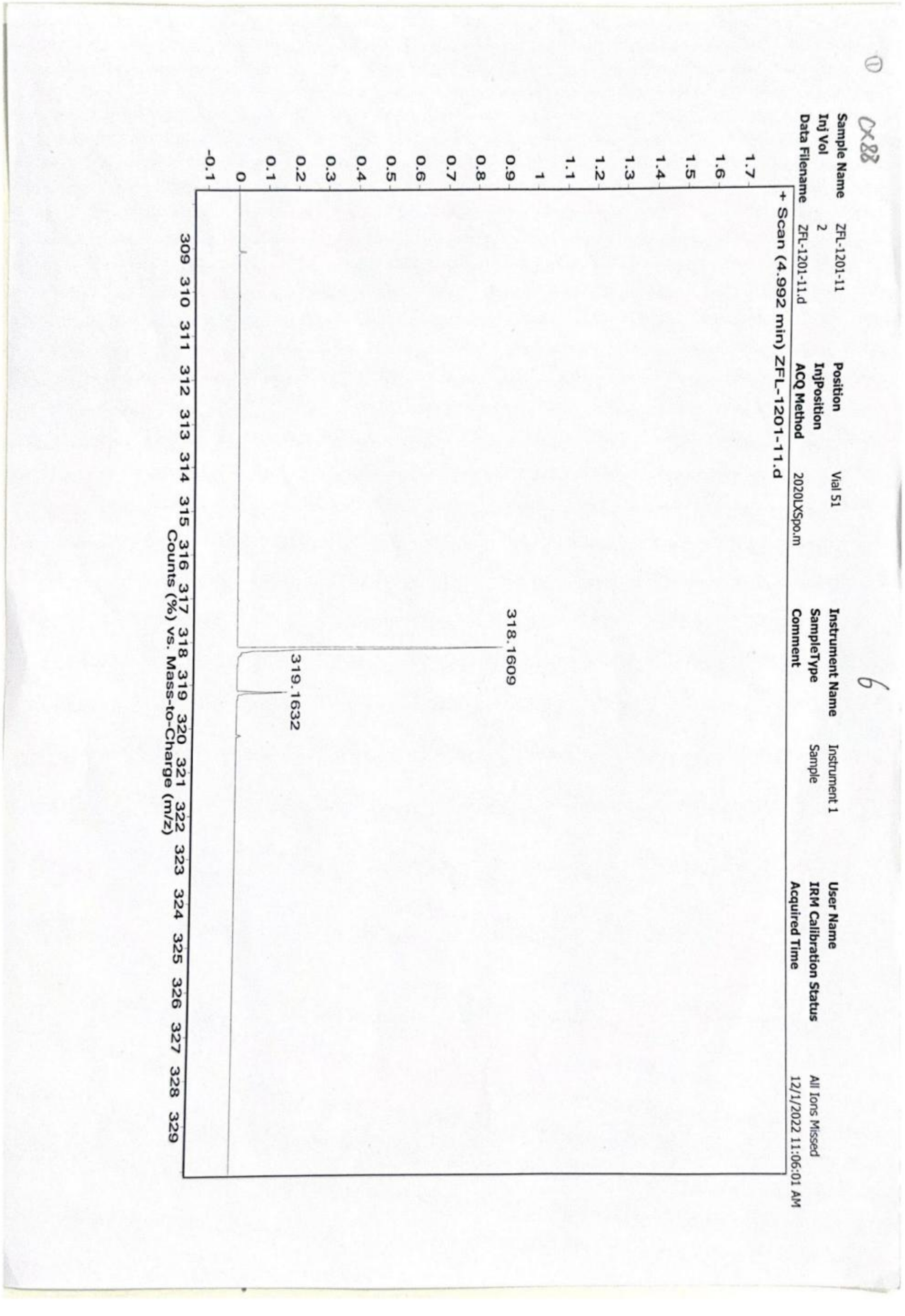

Figure S1. The ^{1}H NMR,^{13}C NMR and ESI-HRMS spectra of compound **6**

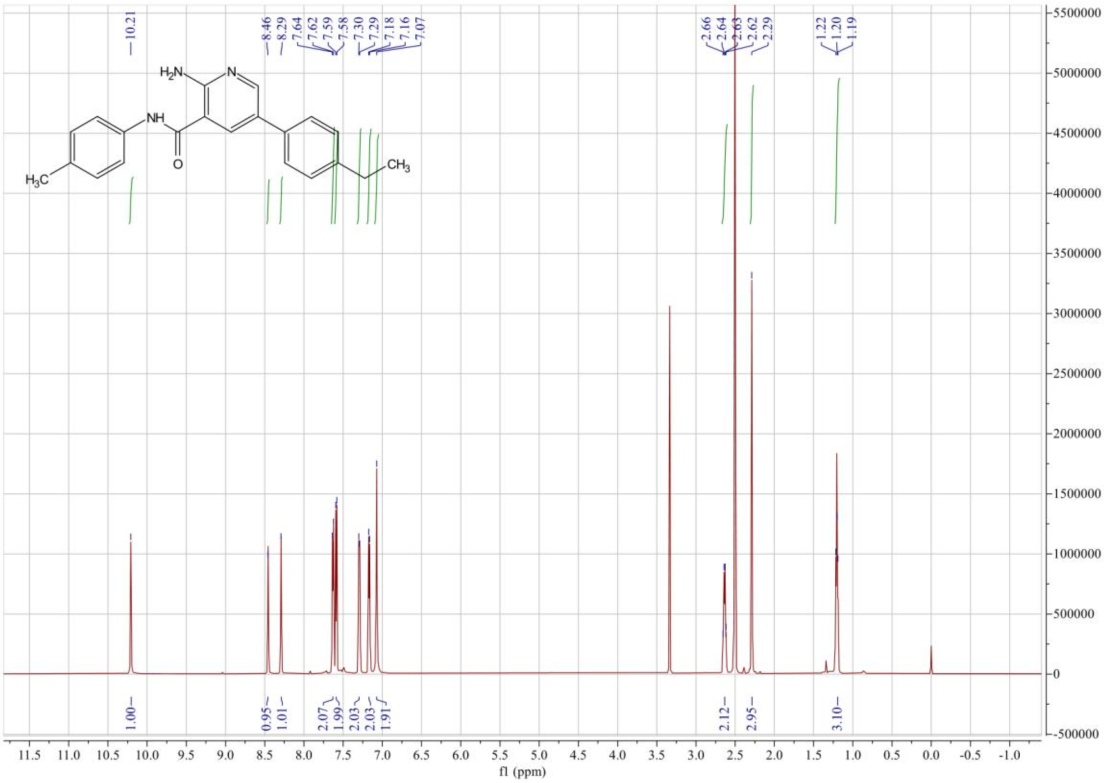

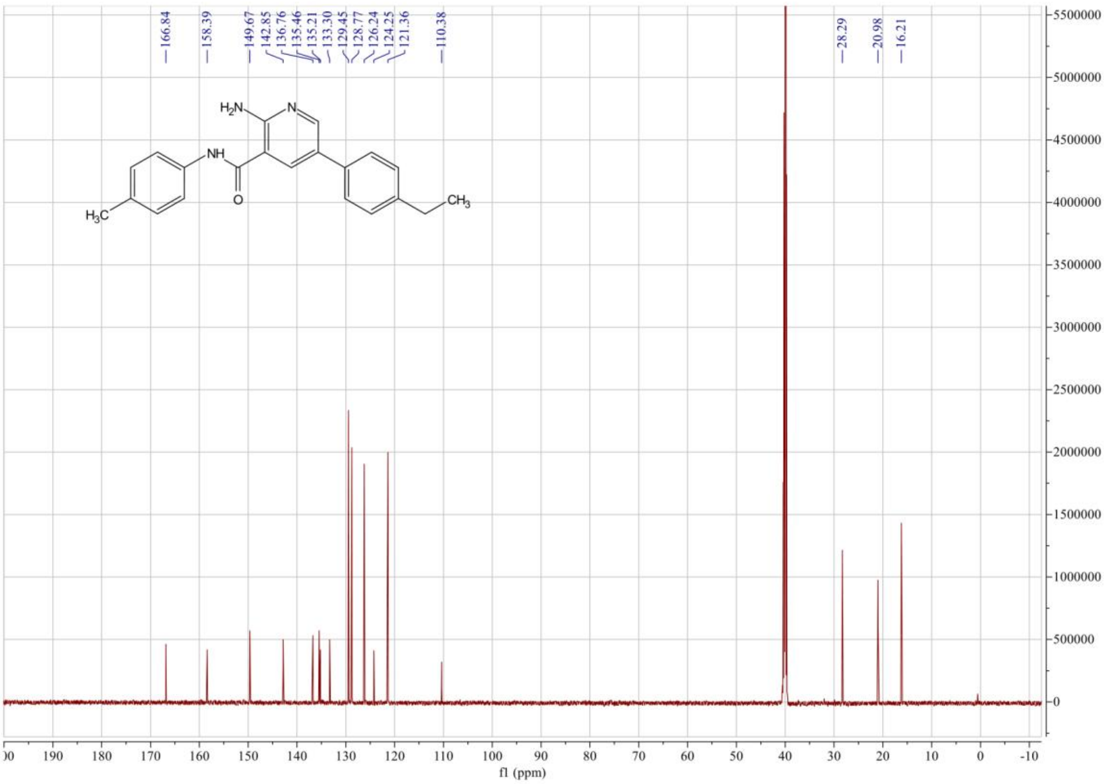

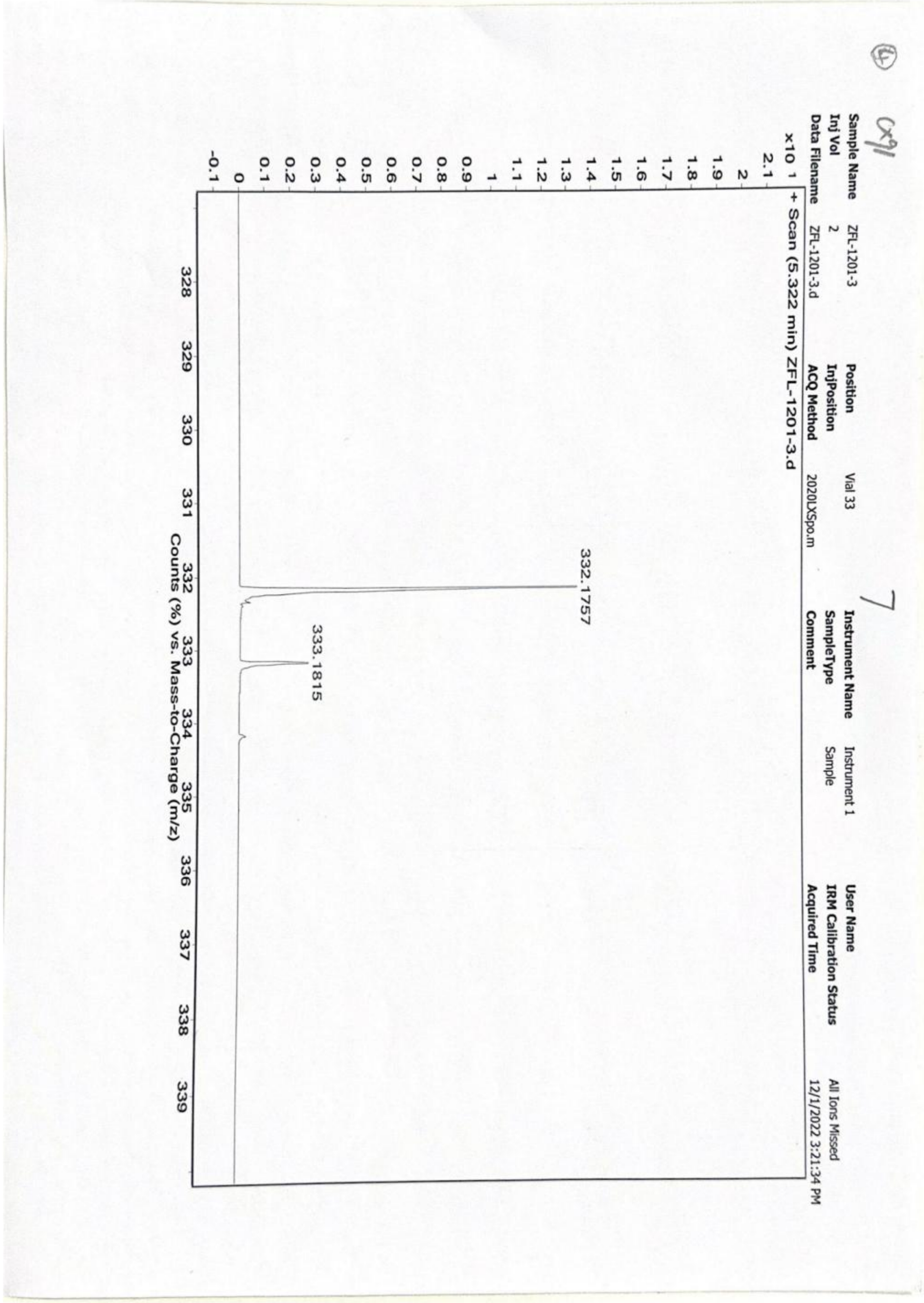

Figure S2. The ^{1}H NMR,^{13}C NMR and ESI-HRMS spectra of compound **7**

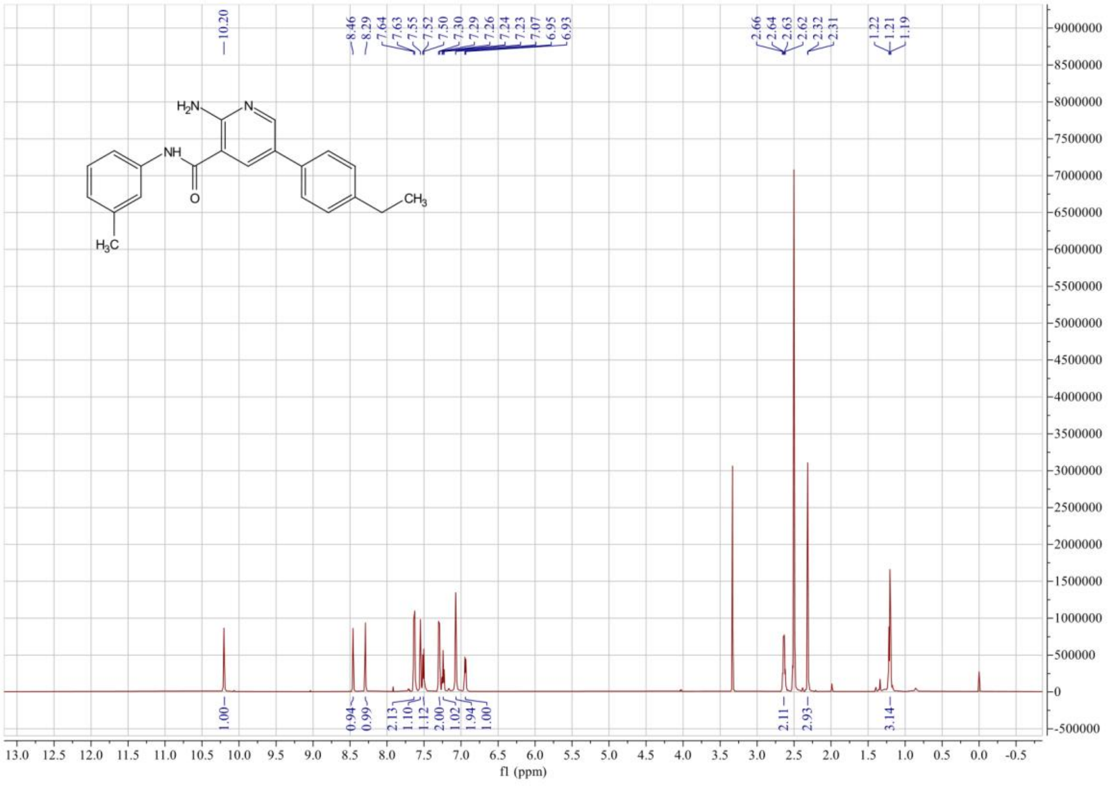
10.20
8.46
8.29
7.64
7.63
7.55
7.52
7.50
7.30
7.29
7.26
7.24
7.23
7.07
6.95
6.93
2.66
2.64
2.63
2.62
2.32
2.31
1.22
1.21
1.19
H2N
N
NH
O
CH3
H3C
1.00
0.94
0.99
2.13
1.10
1.12
2.00
1.02
1.94
1.00
2.11
2.93
3.14
f1 (ppm)

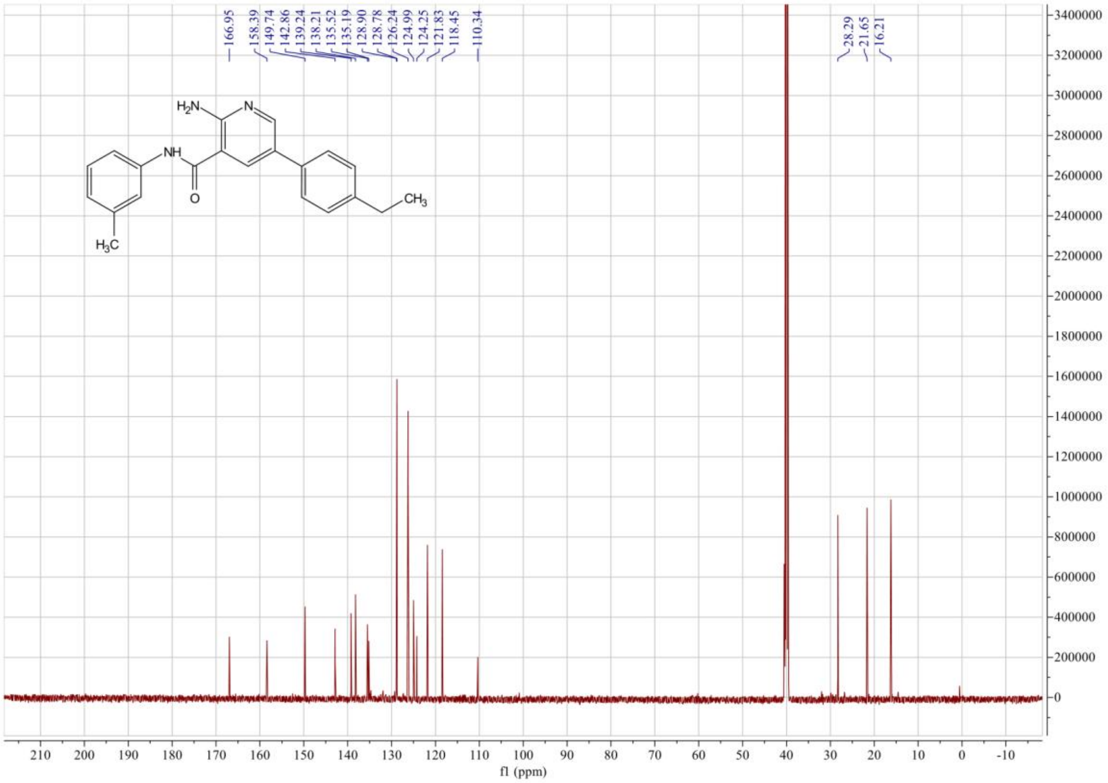
166.95
158.39
149.74
142.86
139.24
138.21
135.52
135.19
128.90
128.78
126.24
124.99
124.25
121.83
118.45
110.34
28.29
21.65
16.21
H2N
N
NH
O
CH3
H3C
f1 (ppm)

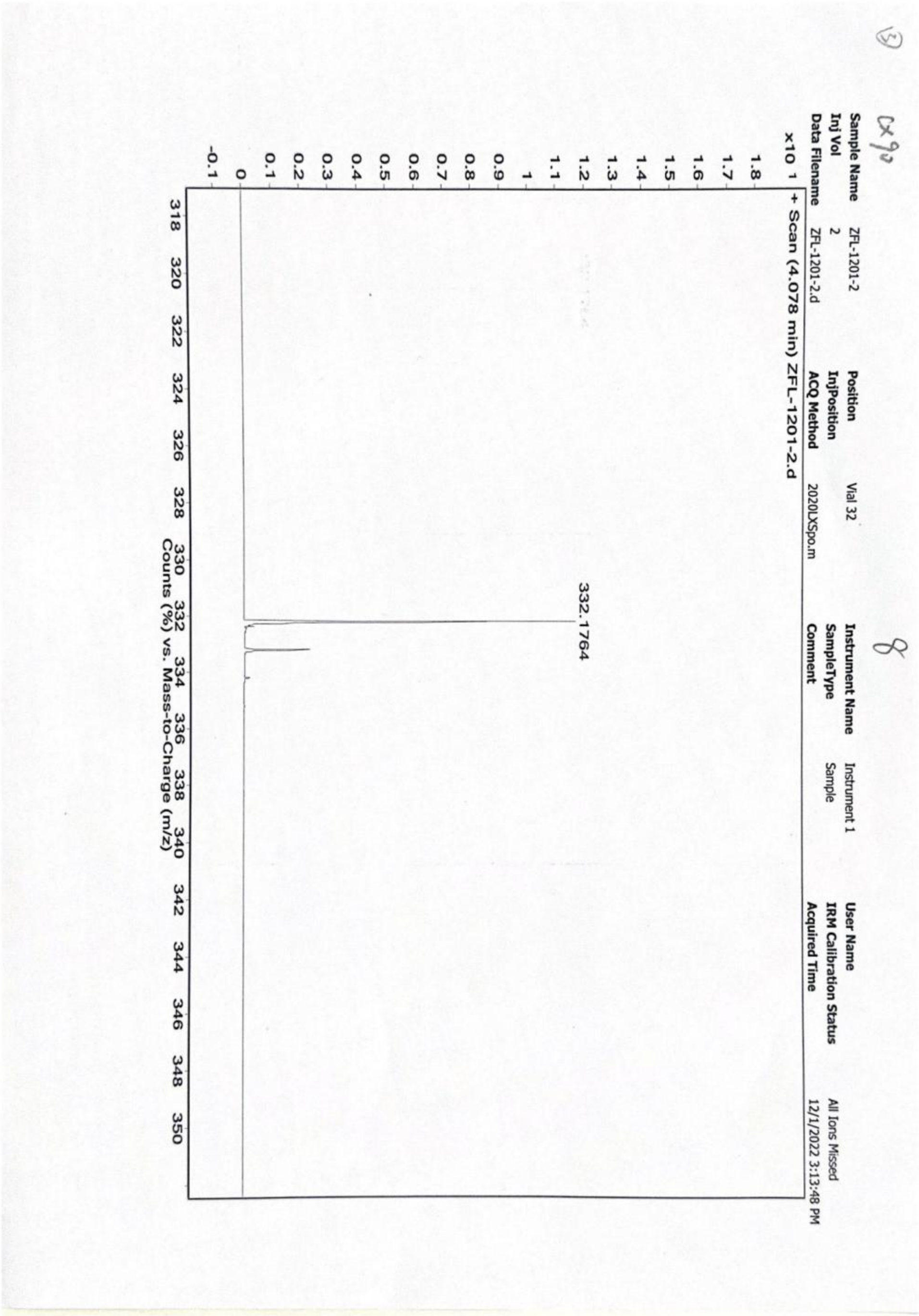

Figure S3. The ^{1}H NMR,^{13}C NMR and ESI-HRMS spectra of compound **8**

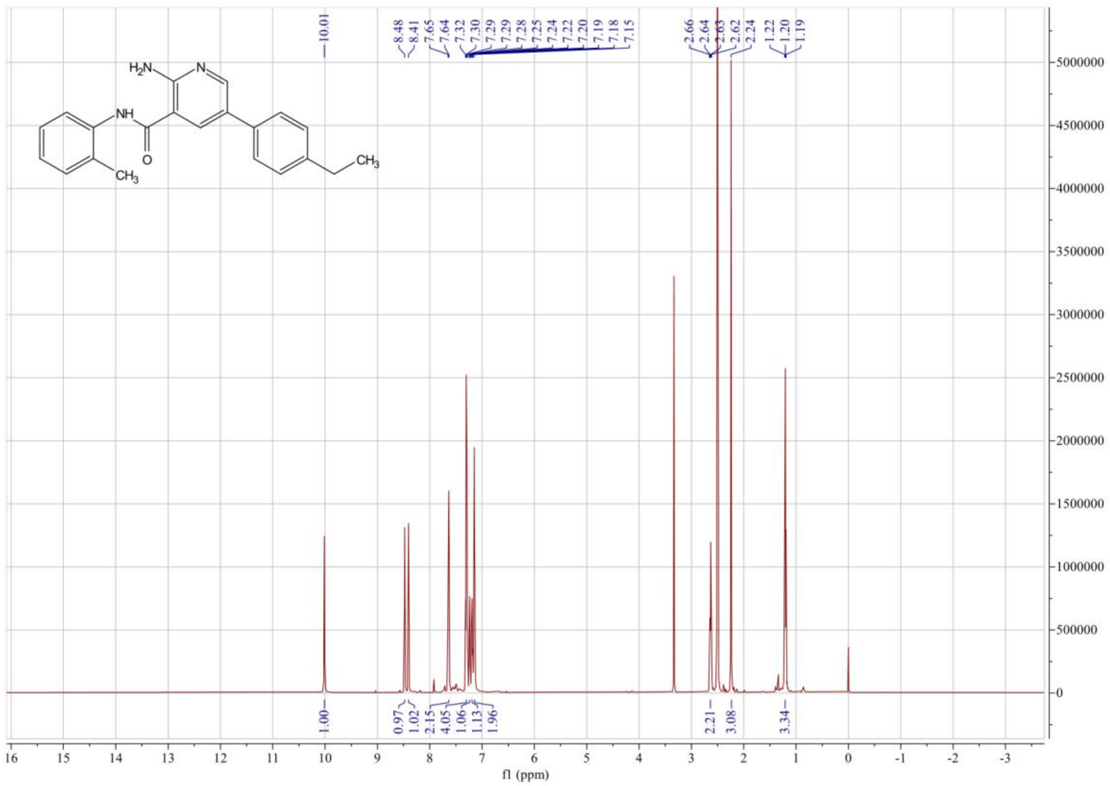

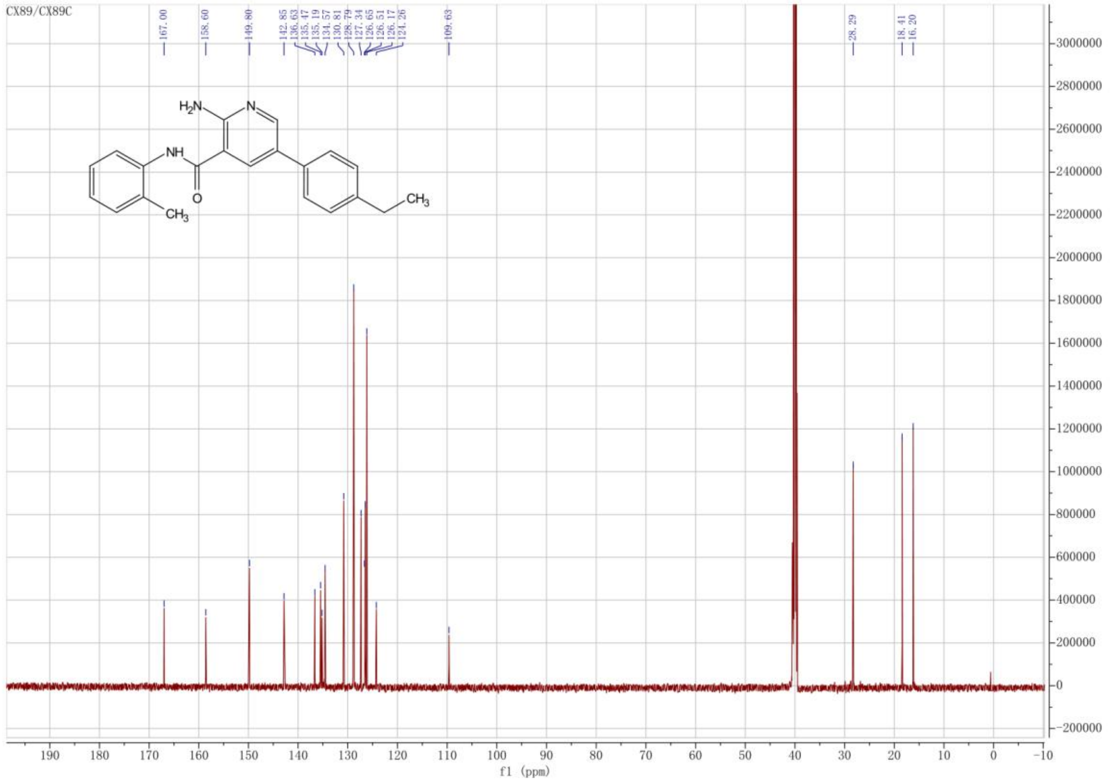

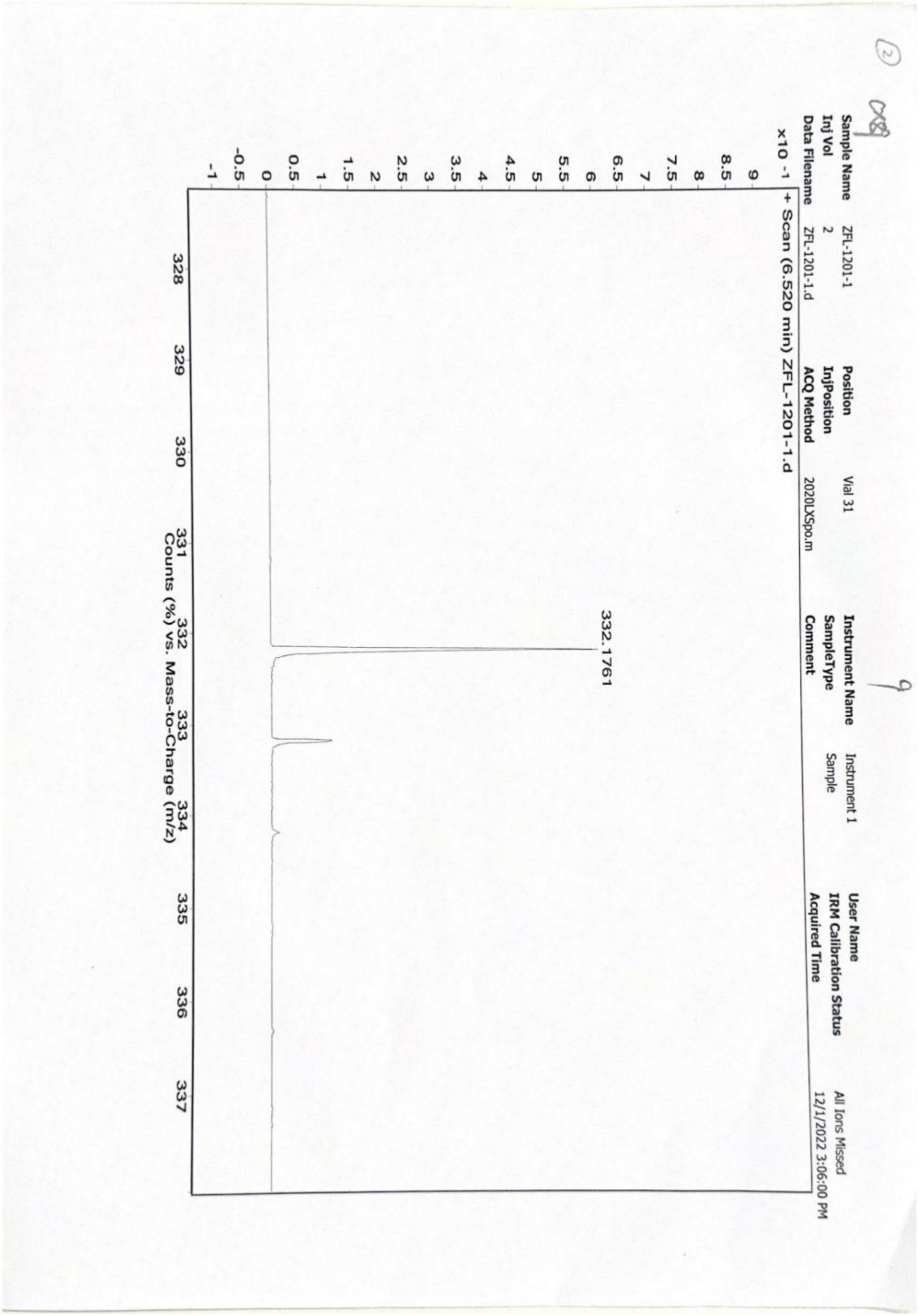

Figure S4. The ^{1}H NMR,^{13}C NMR and ESI-HRMS spectra of compound **9**

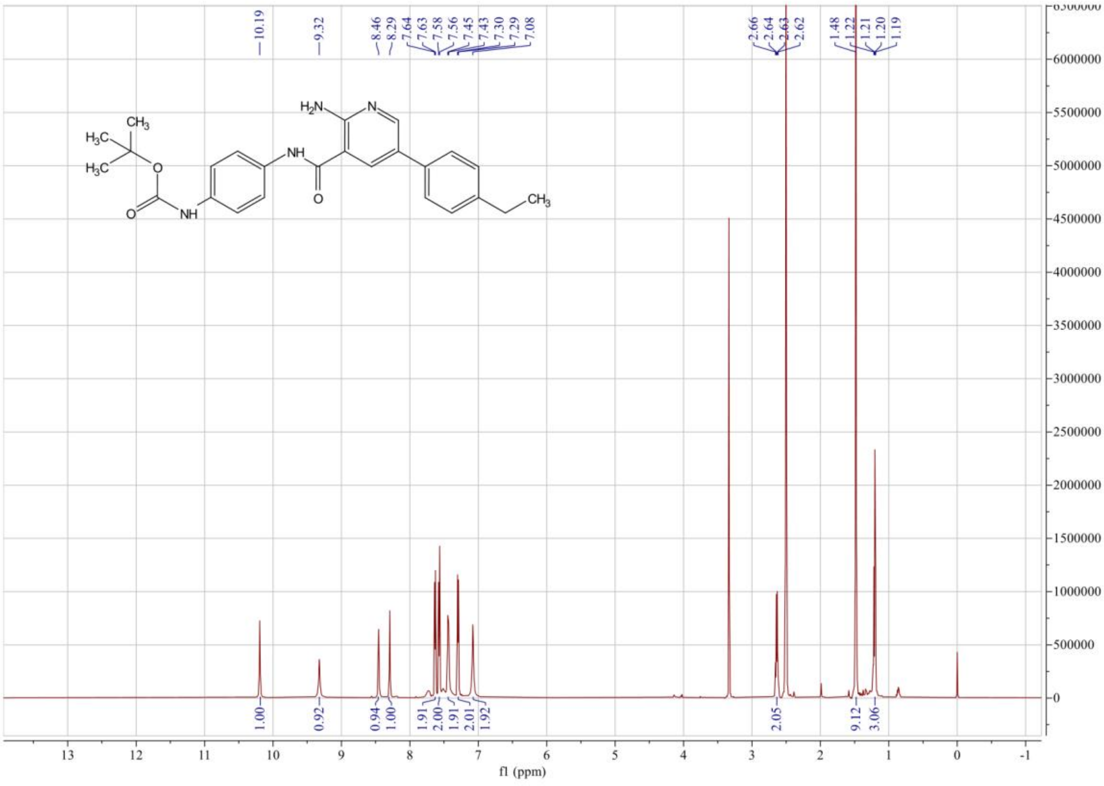

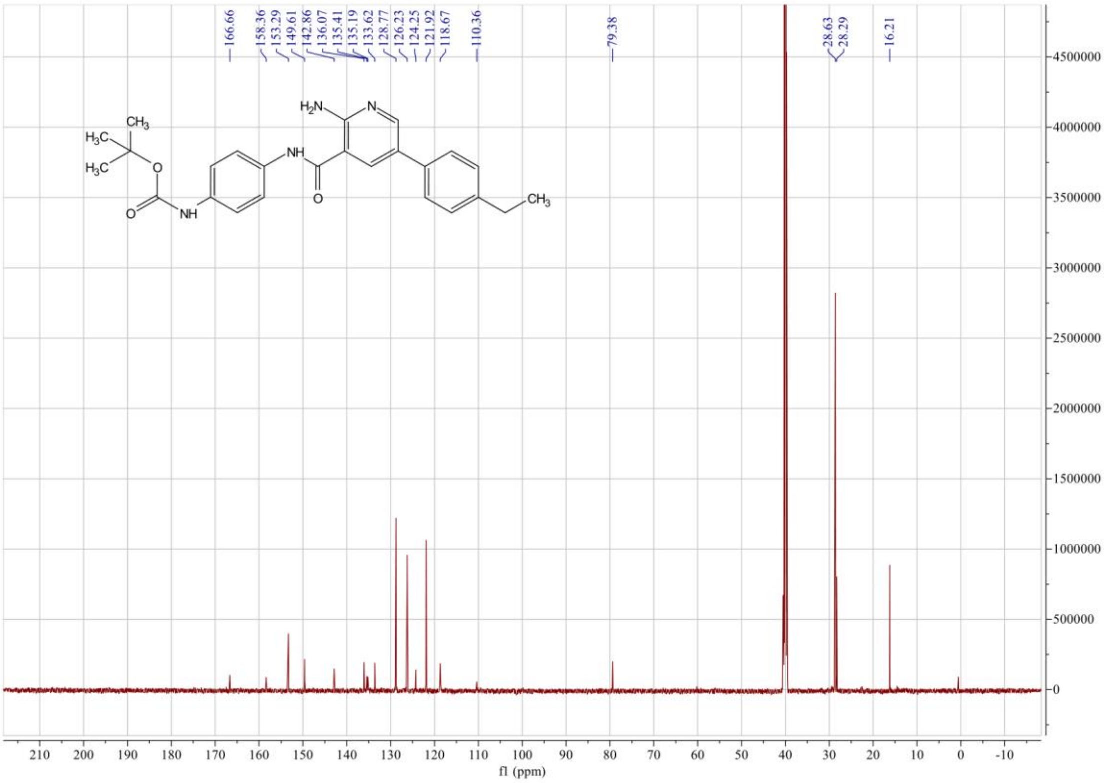

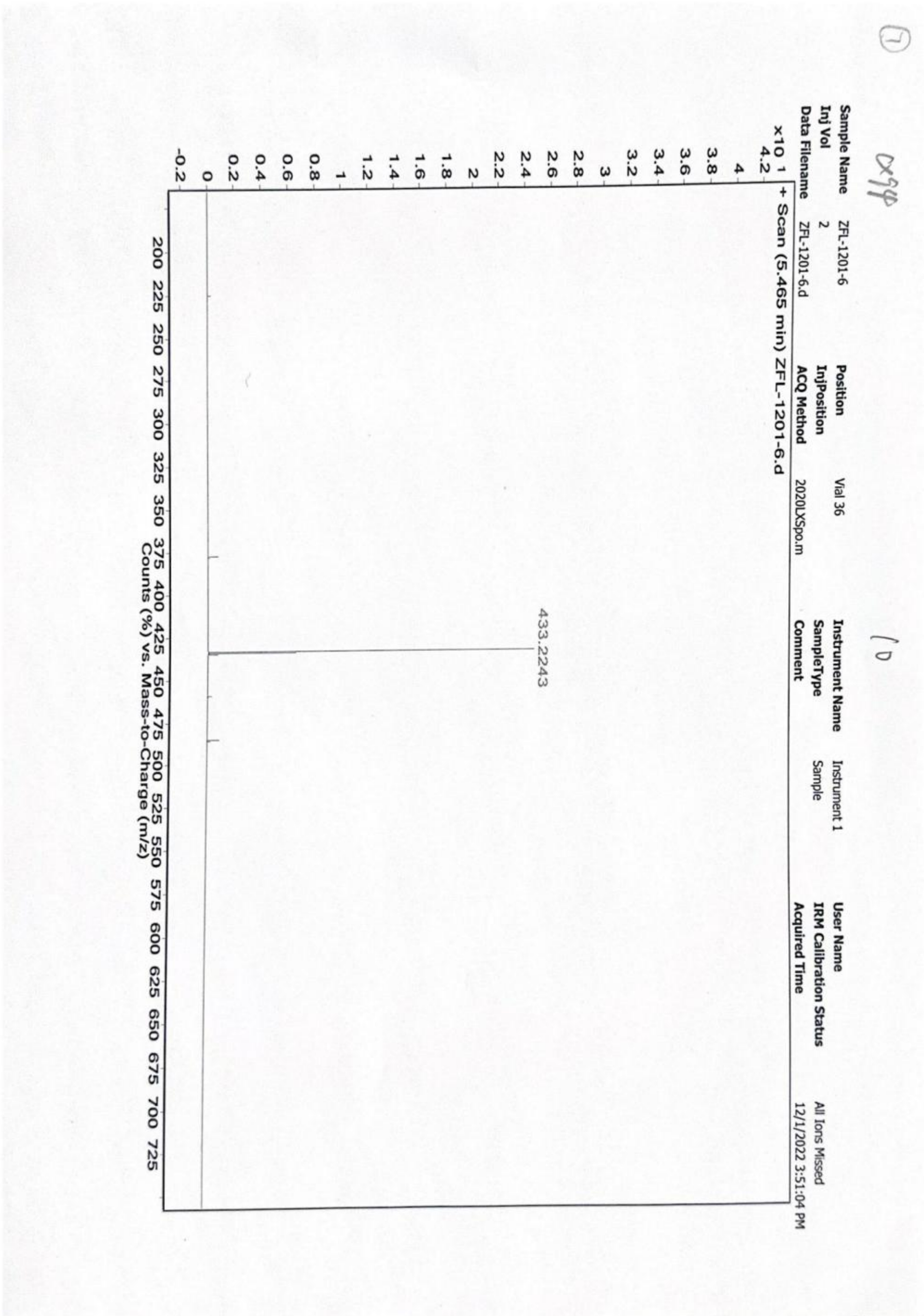

Figure S5. The ^{1}H NMR, ^{13}C NMR and ESI-HRMS spectra of compound **10**

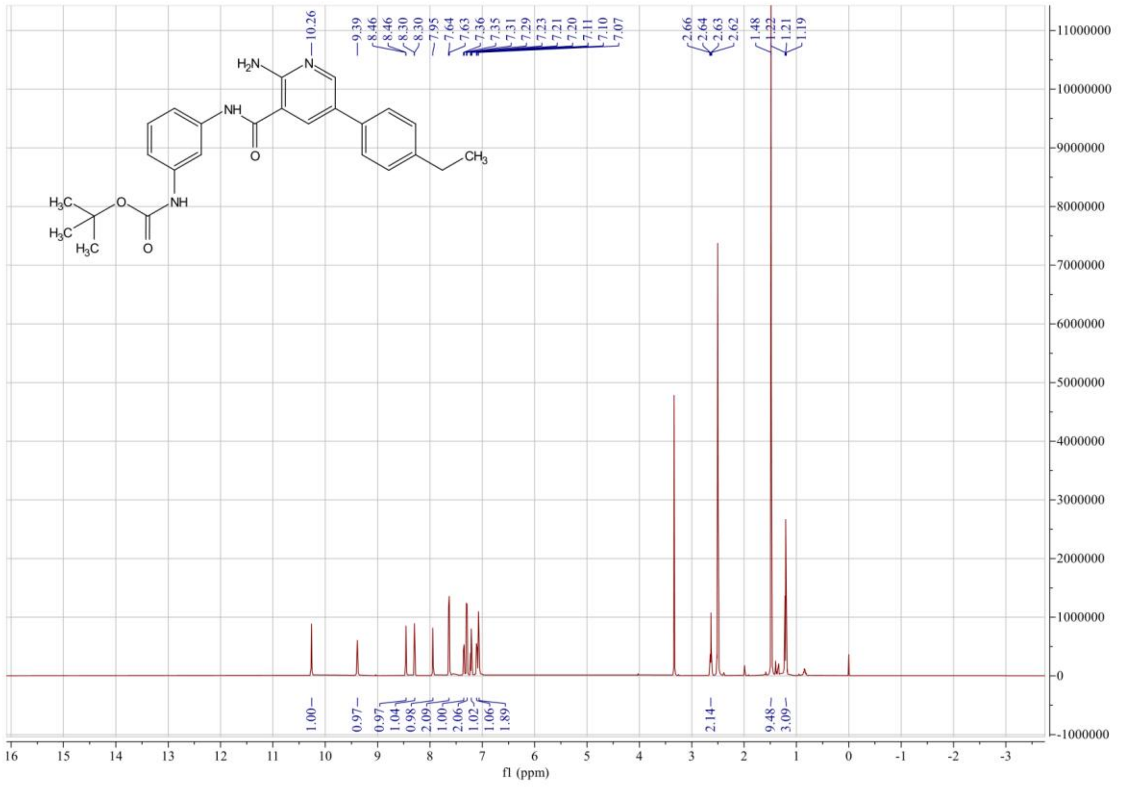
10.26
9.39
8.46
8.30
8.30
7.95
7.64
7.63
7.36
7.35
7.31
7.29
7.23
7.21
7.20
7.11
7.10
7.07
2.66
2.64
2.63
2.62
1.48
1.22
1.21
1.19
1.00
0.97
0.97
1.04
0.98
2.09
1.00
2.06
1.02
1.06
1.89
2.14
9.48
3.09
f1 (ppm)

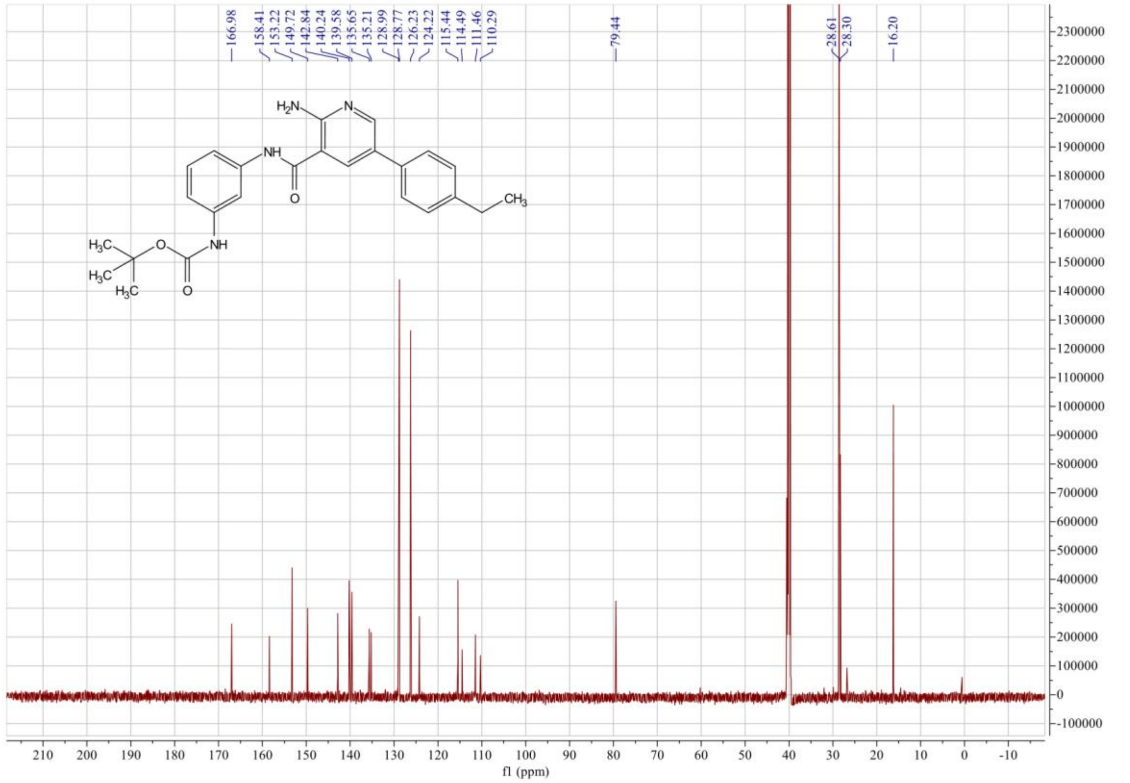
166.98
158.41
153.22
149.72
142.84
140.24
139.58
135.65
135.21
128.99
128.77
126.23
124.22
115.44
114.49
111.46
110.29
79.44
28.61
28.30
16.20
f1 (ppm)

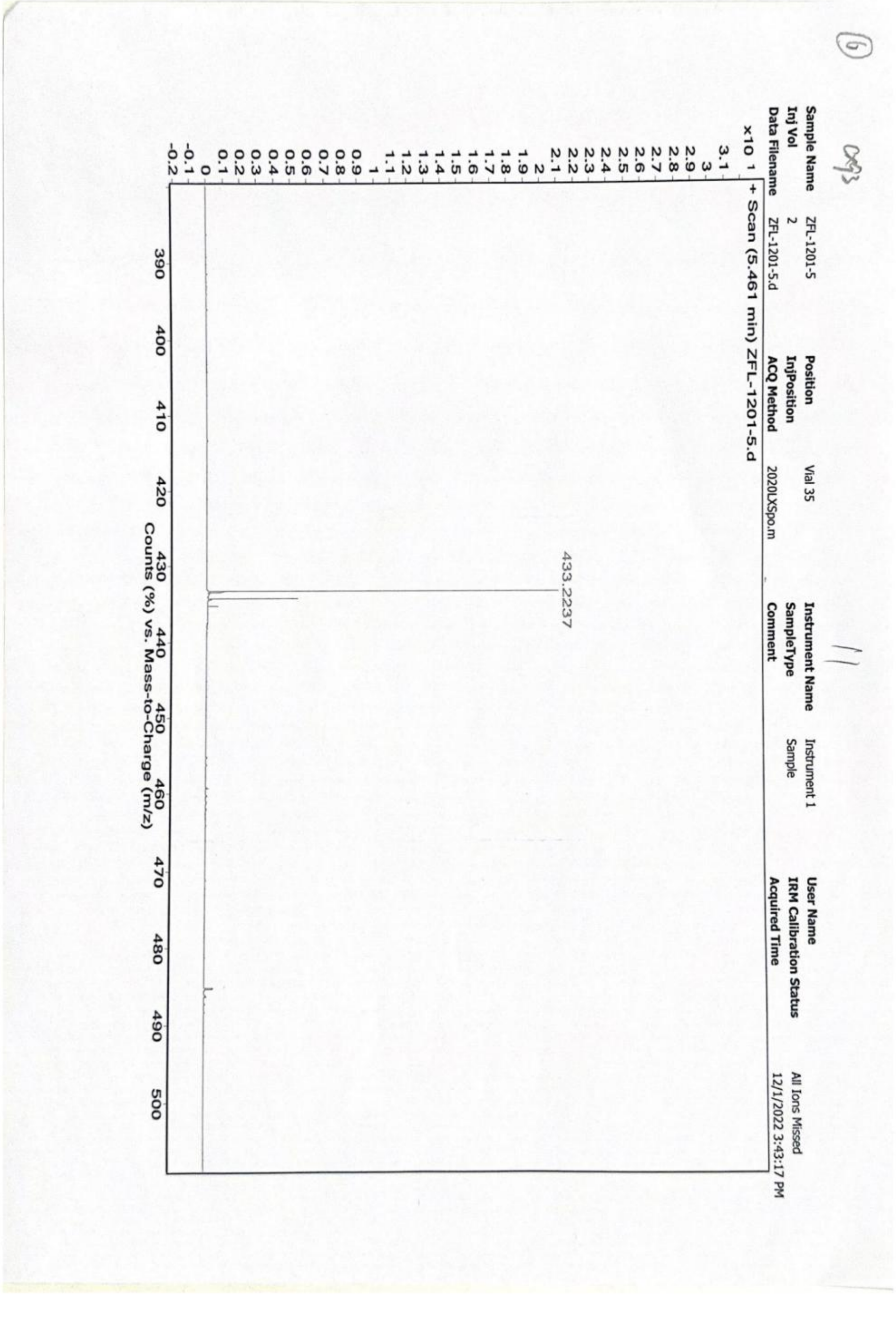

Figure S6. The ^{1}H NMR,^{13}C NMR and ESI-HRMS spectra of compound **11**

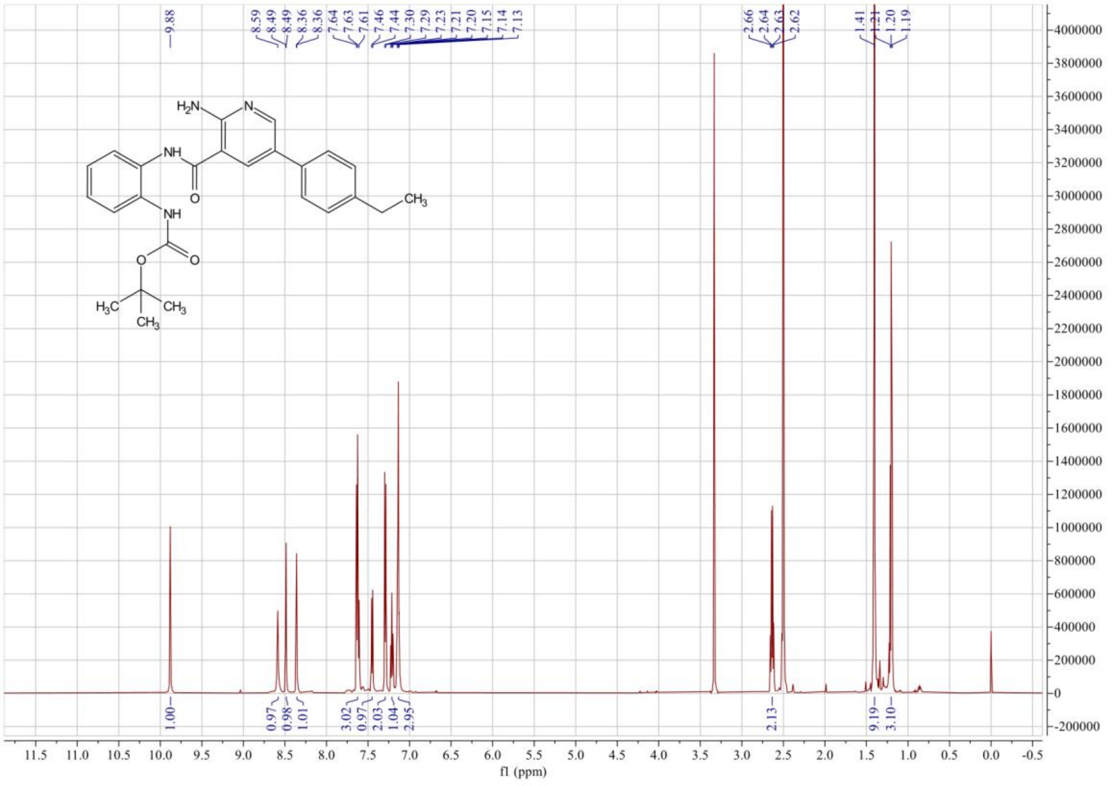

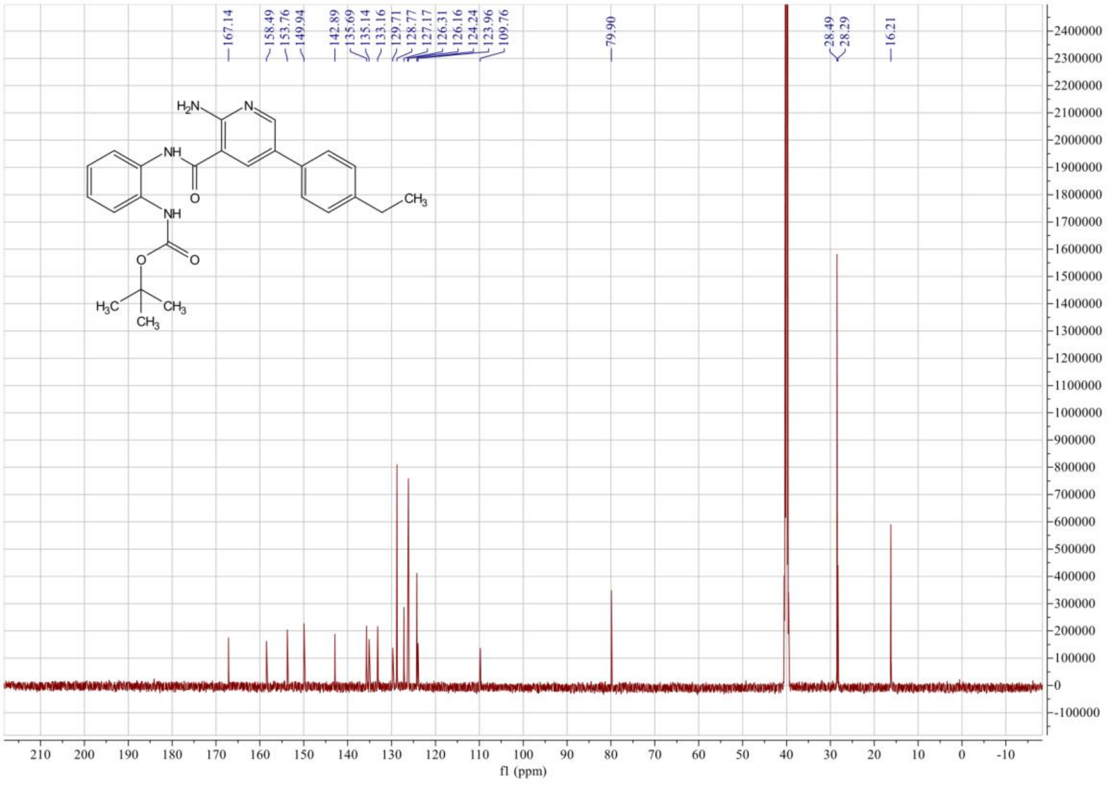

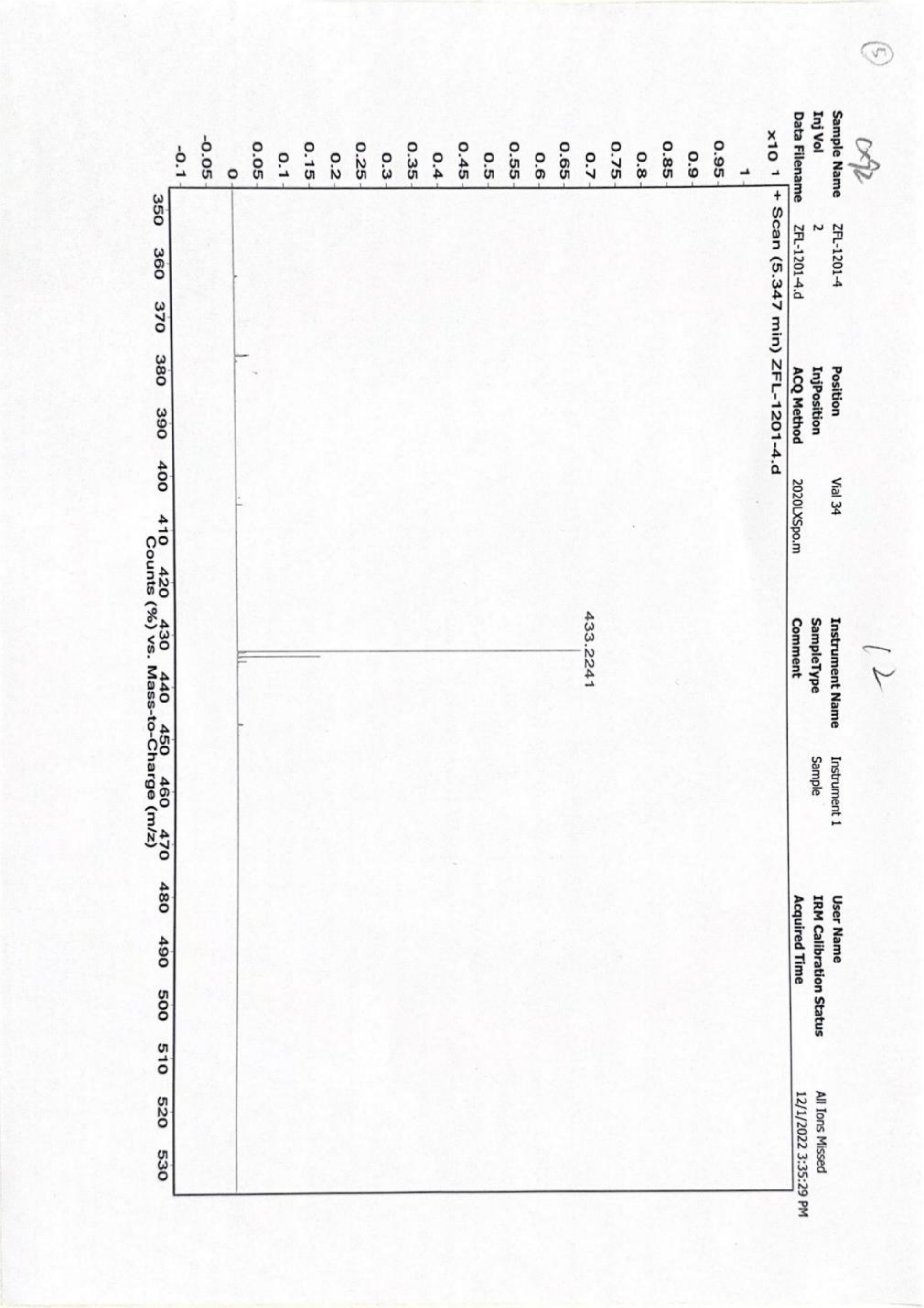

Figure S7. The ^{1}H NMR,^{13}C NMR and ESI-HRMS spectra of compound **12**

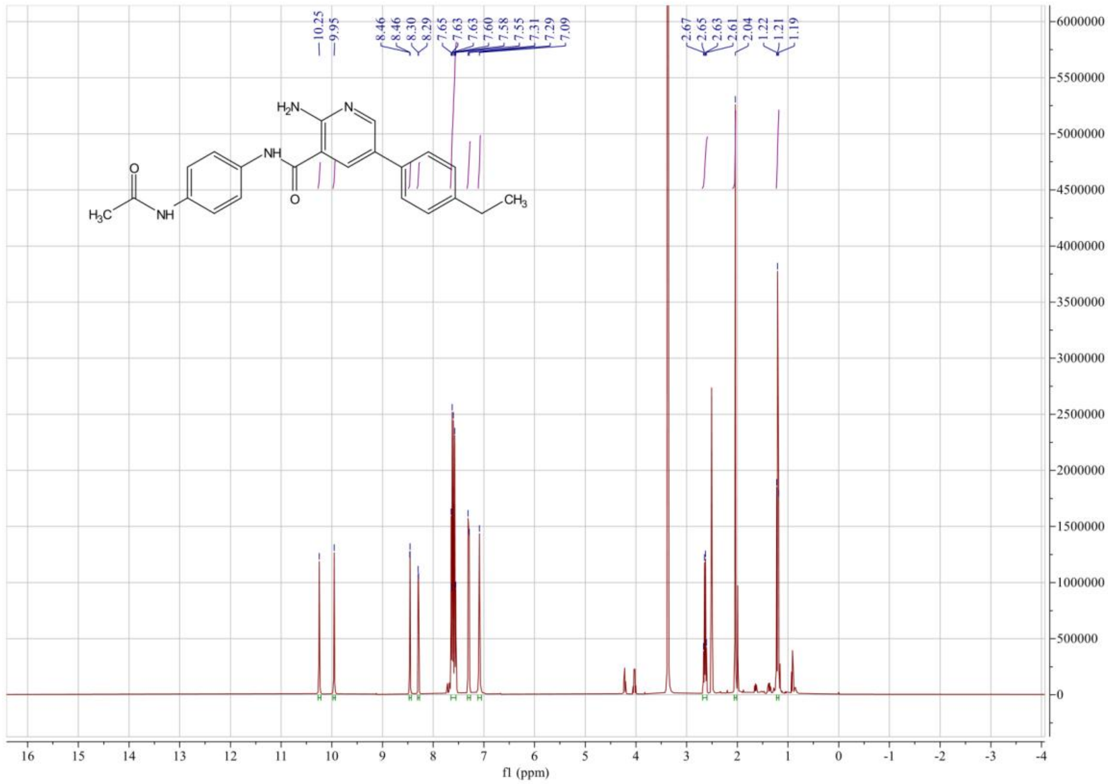
10.25
9.95
8.46
8.46
8.30
8.29
7.65
7.63
7.63
7.60
7.58
7.55
7.31
7.29
7.09
2.67
2.65
2.63
2.61
2.04
1.22
1.21
1.19
H2N
N
NH
O
H3C
NH
O
CH3
f1 (ppm)

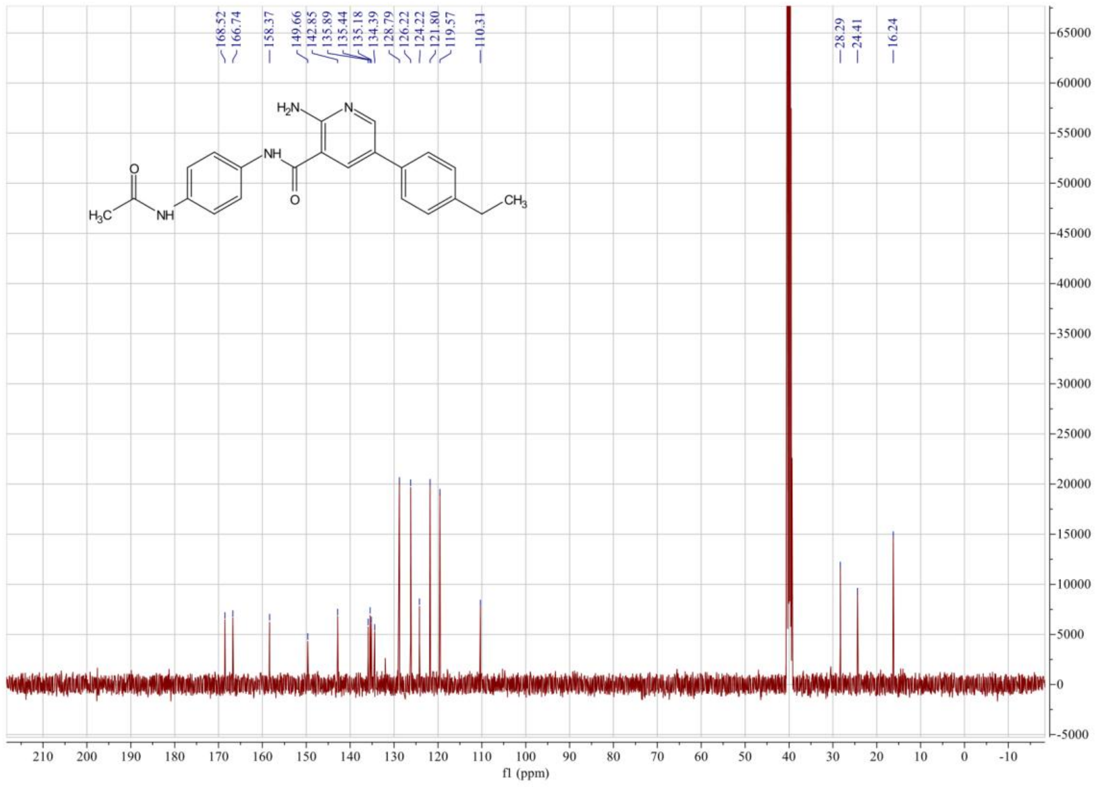
168.52
166.74
158.37
149.66
142.85
135.89
135.44
135.18
134.39
128.79
126.22
124.22
121.80
119.57
110.31
28.29
24.41
16.24
H2N
N
NH
O
H3C
NH
O
CH3
f1 (ppm)

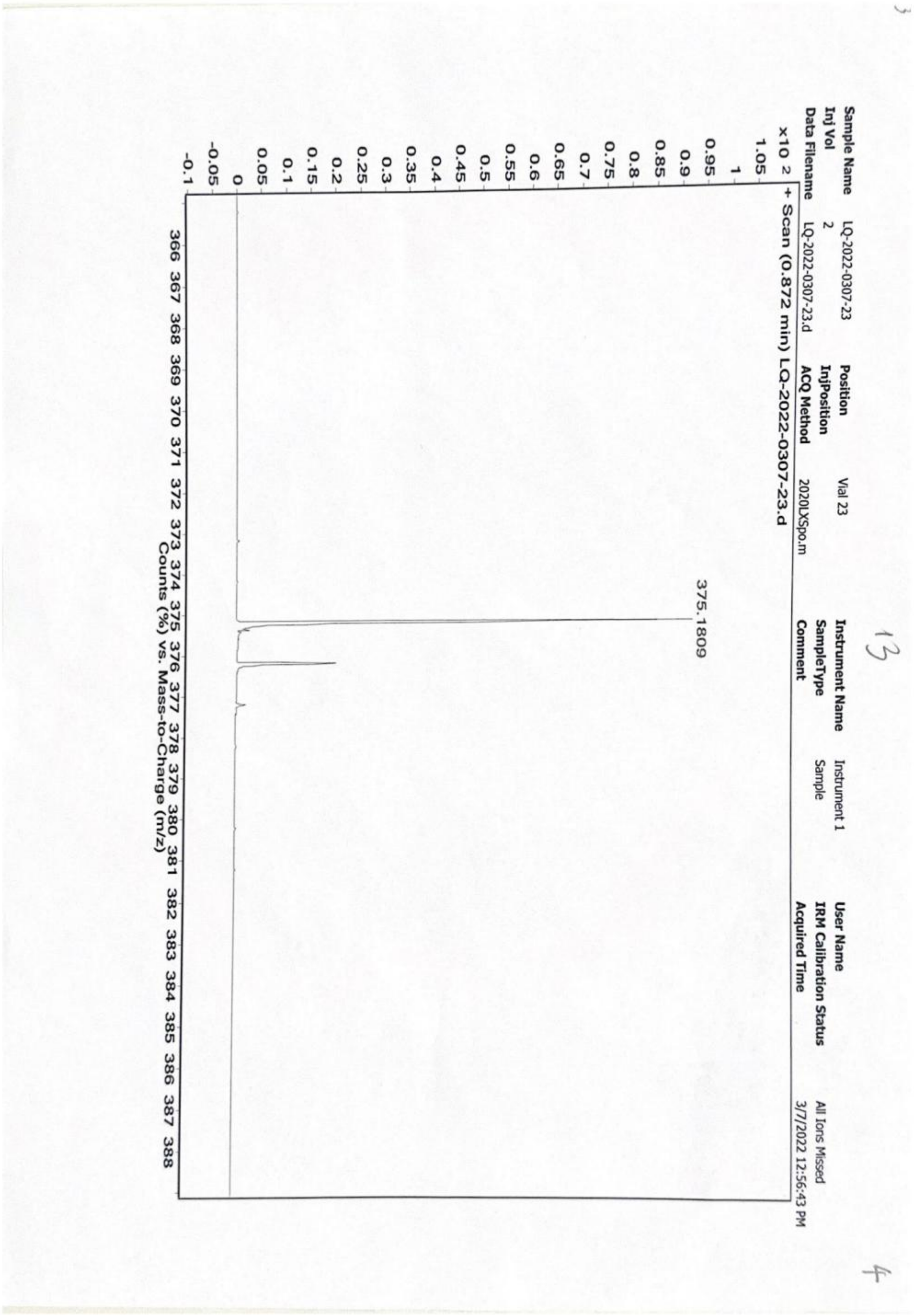

Figure S8. The ^{1}H NMR,^{13}C NMR and ESI-HRMS spectra of compound **13**

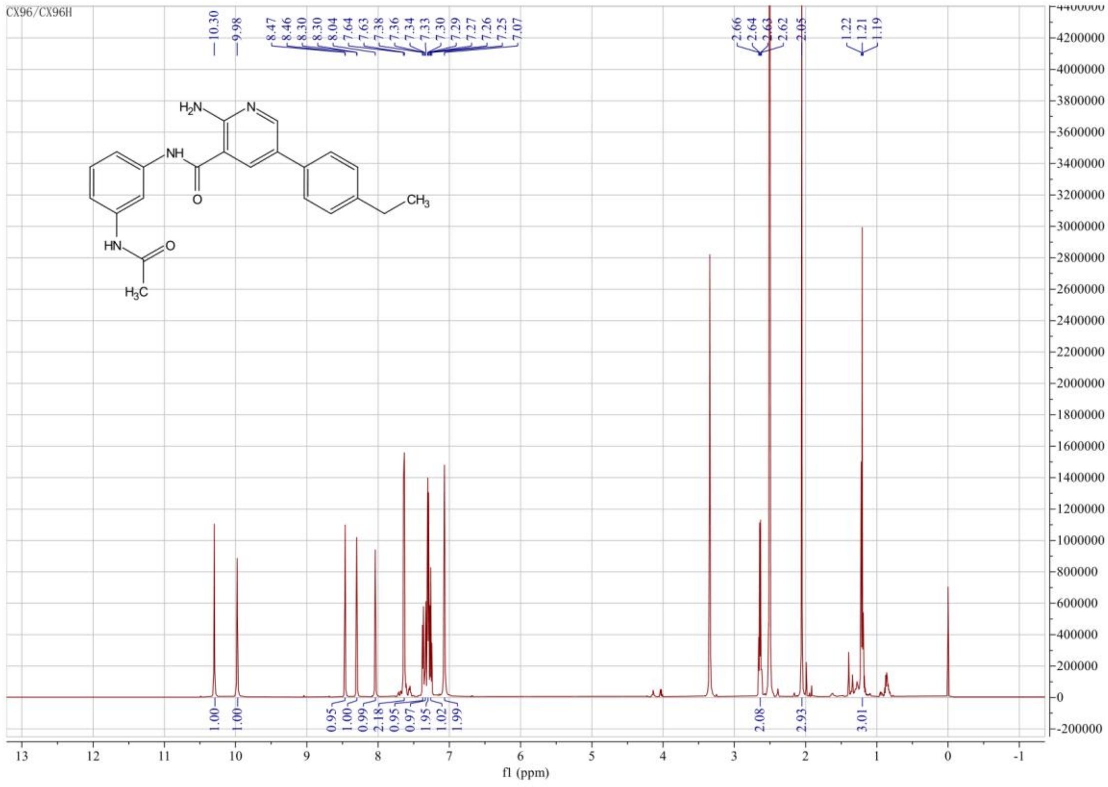
CX96/CX96H
fl (ppm)

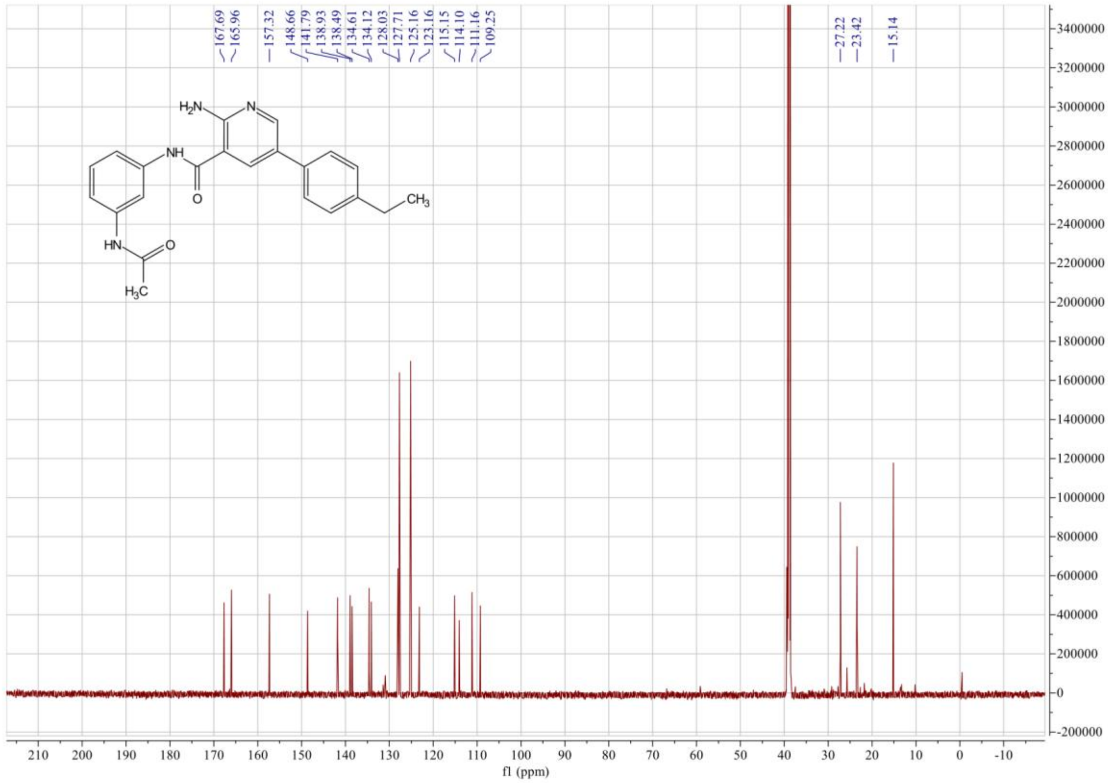
fl (ppm)

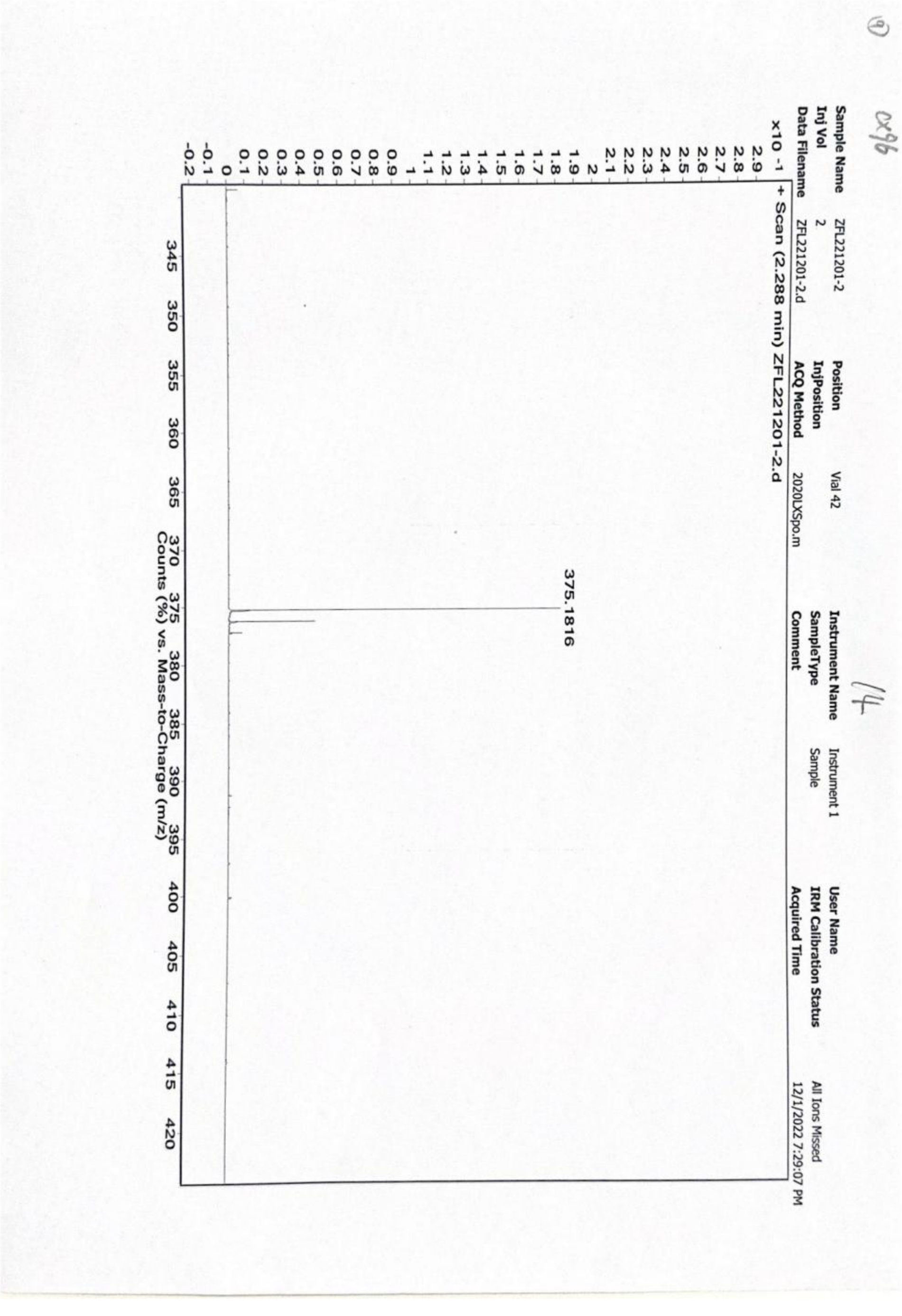

Figure S9. The ^{1}H NMR, ^{13}C NMR and ESI-HRMS spectra of compound **14**

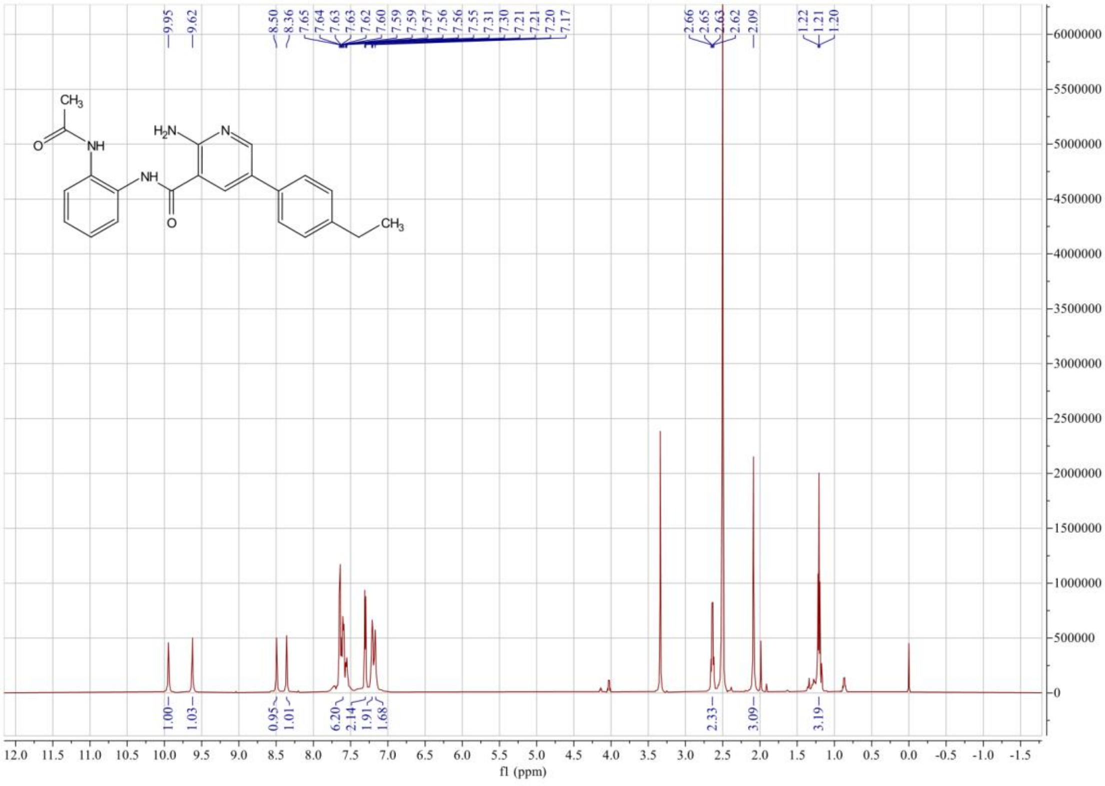

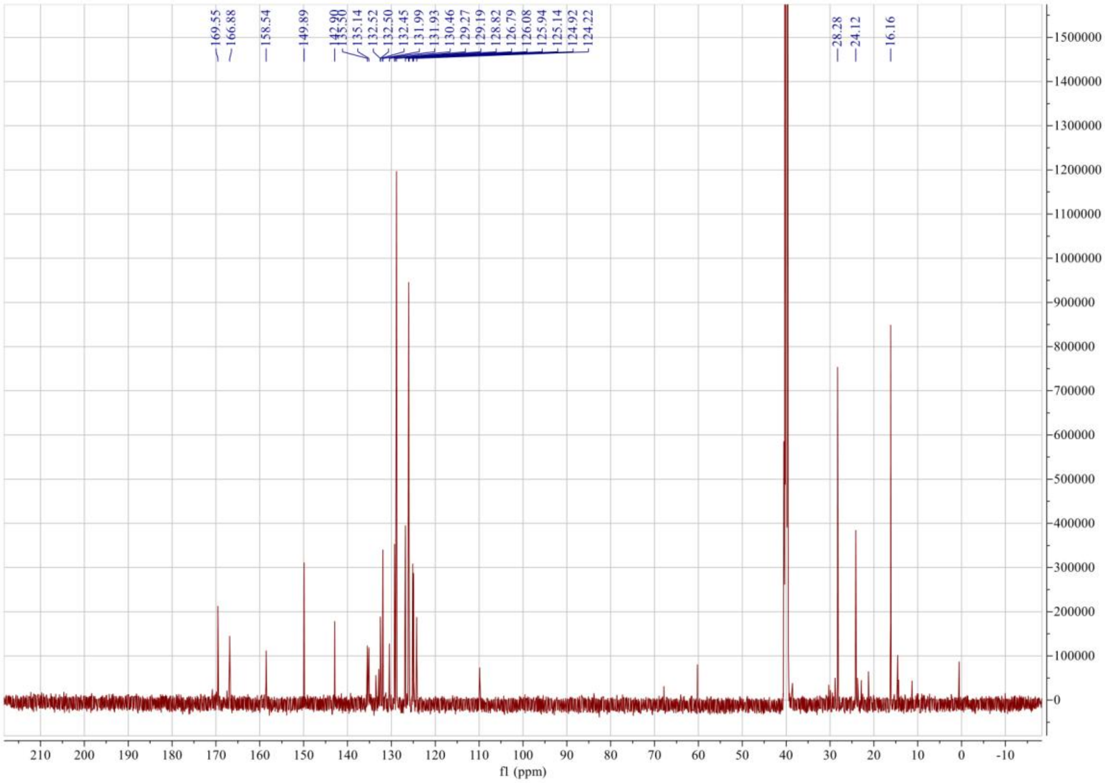

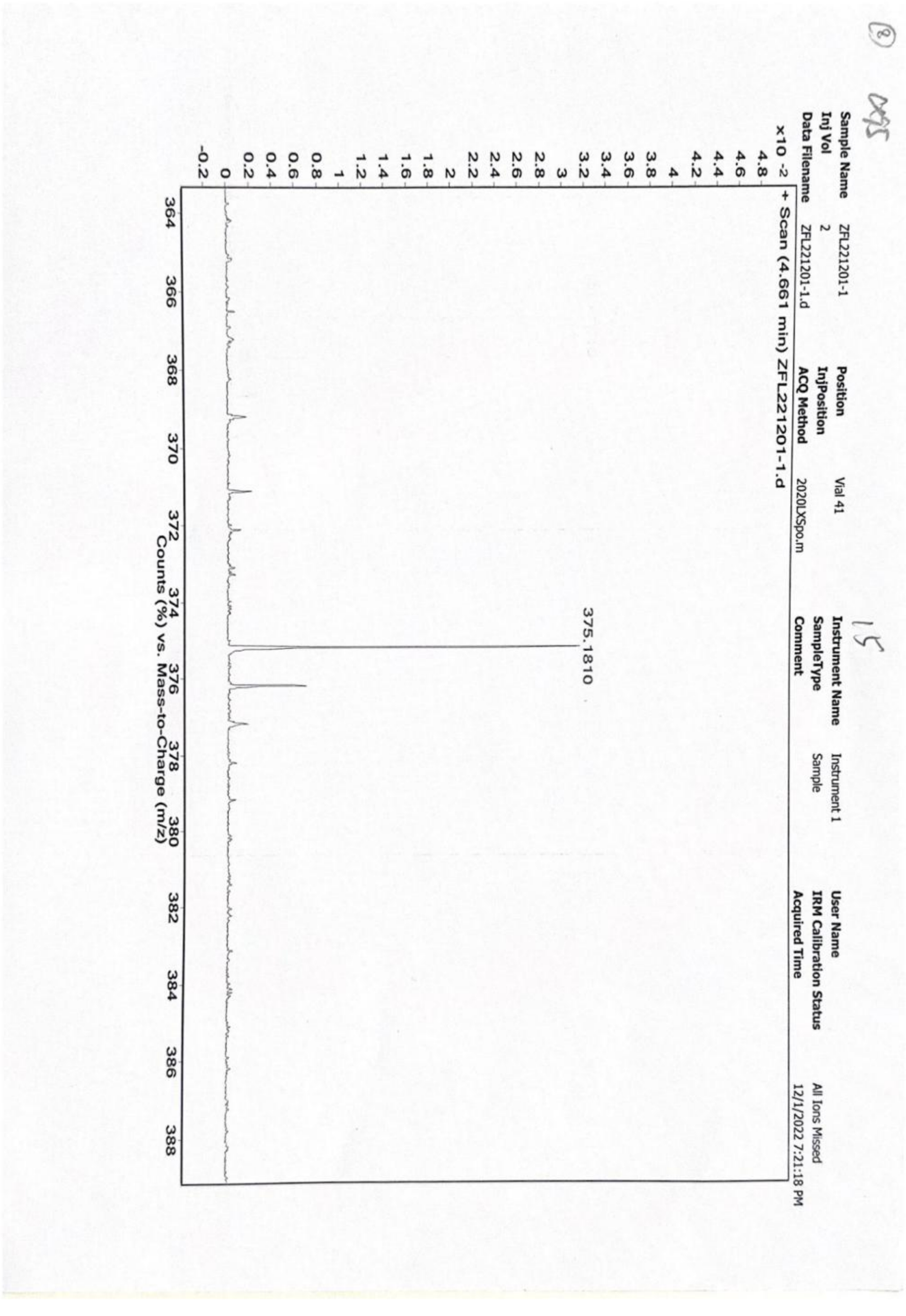

Figure S10. The ^{1}H NMR,^{13}C NMR and ESI-HRMS spectra of compound **15**

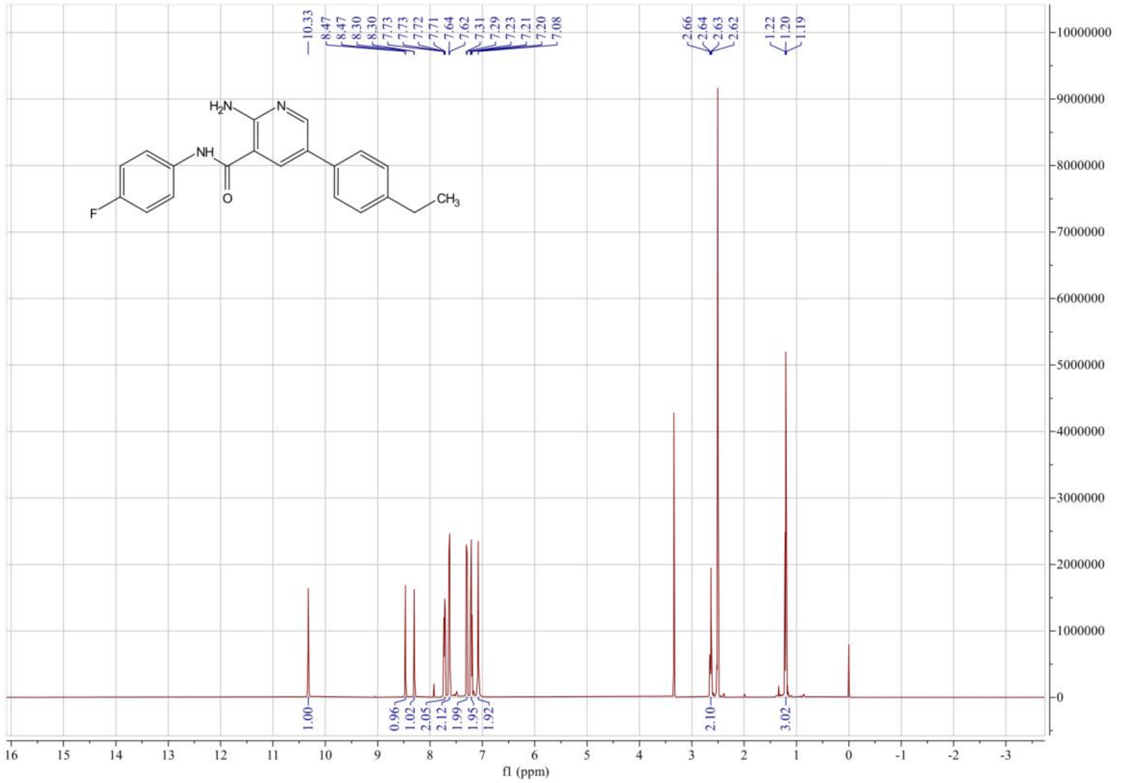
H2N
N
NH
O
F
CH3
f1 (ppm)

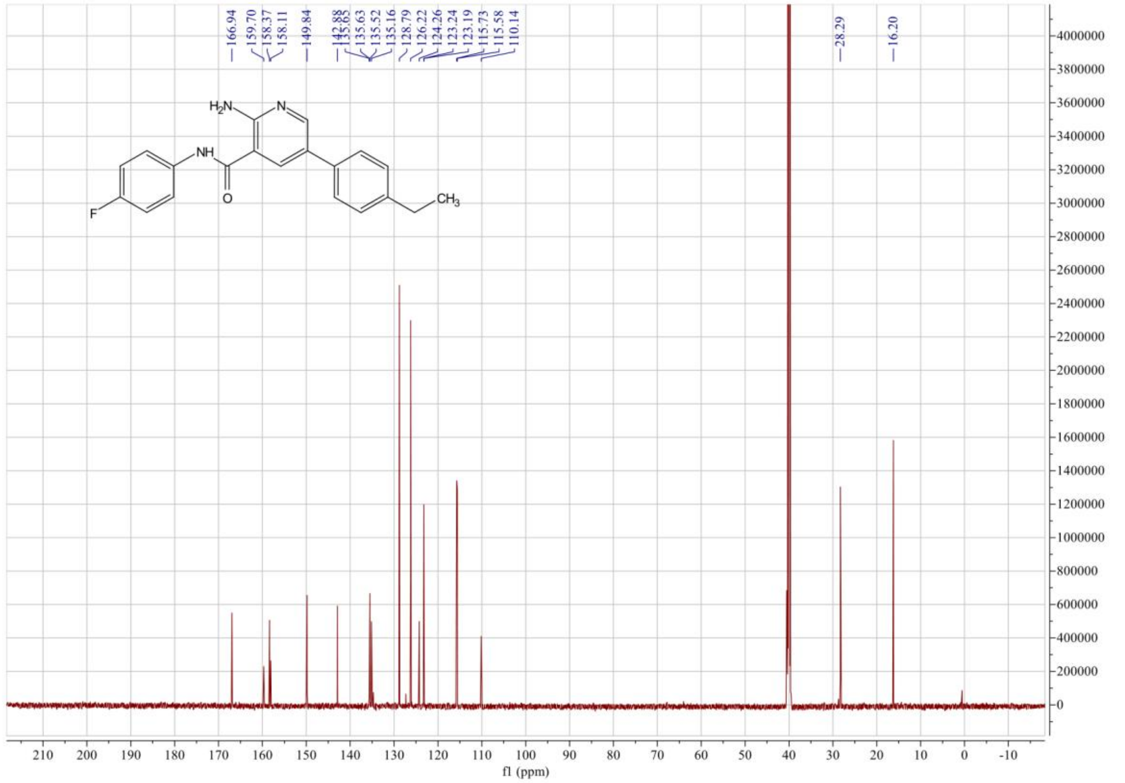
H2N
N
NH
O
F
CH3
f1 (ppm)

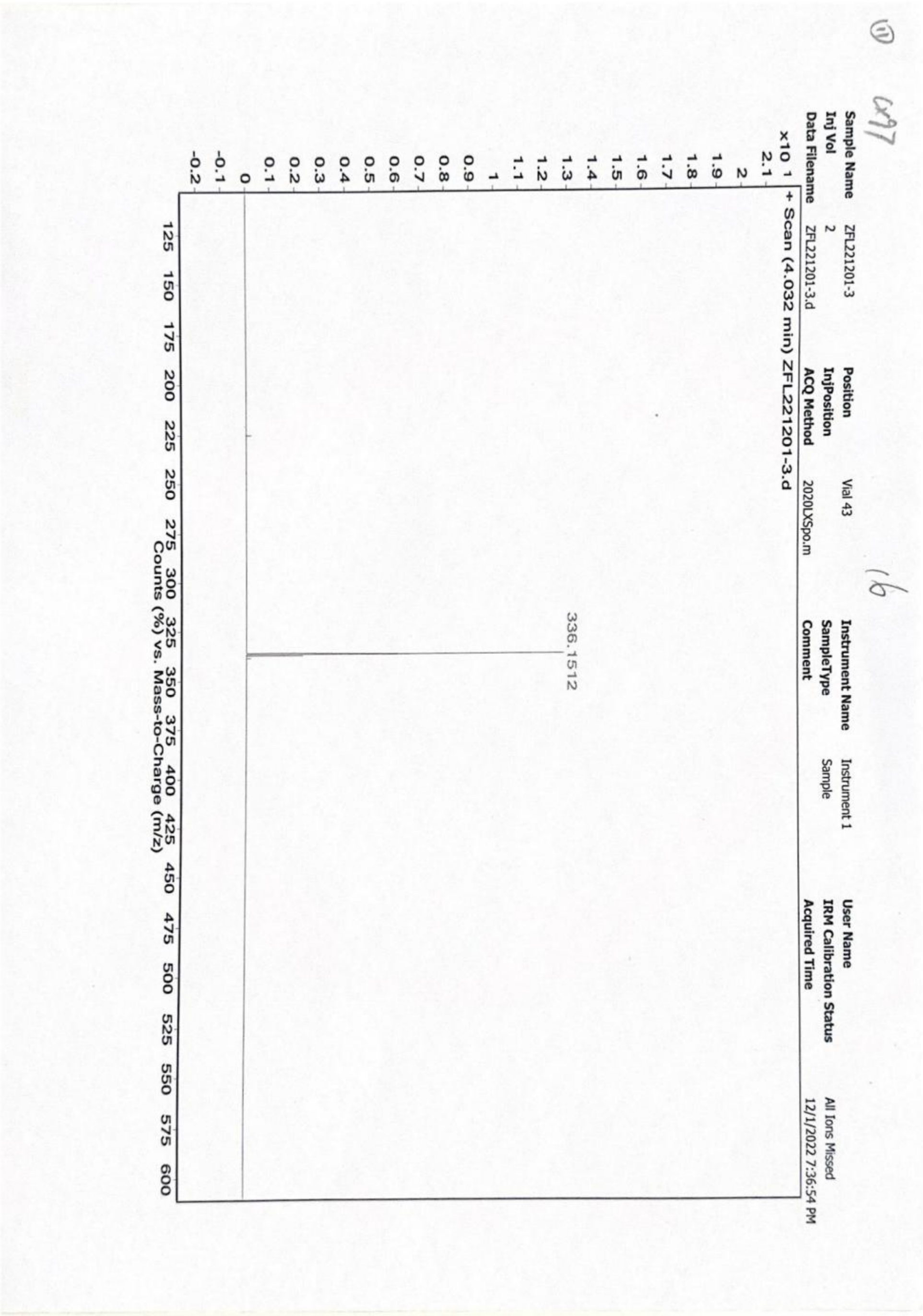

Figure S11. The ^{1}H NMR,^{13}C NMR and ESI-HRMS spectra of compound **16**

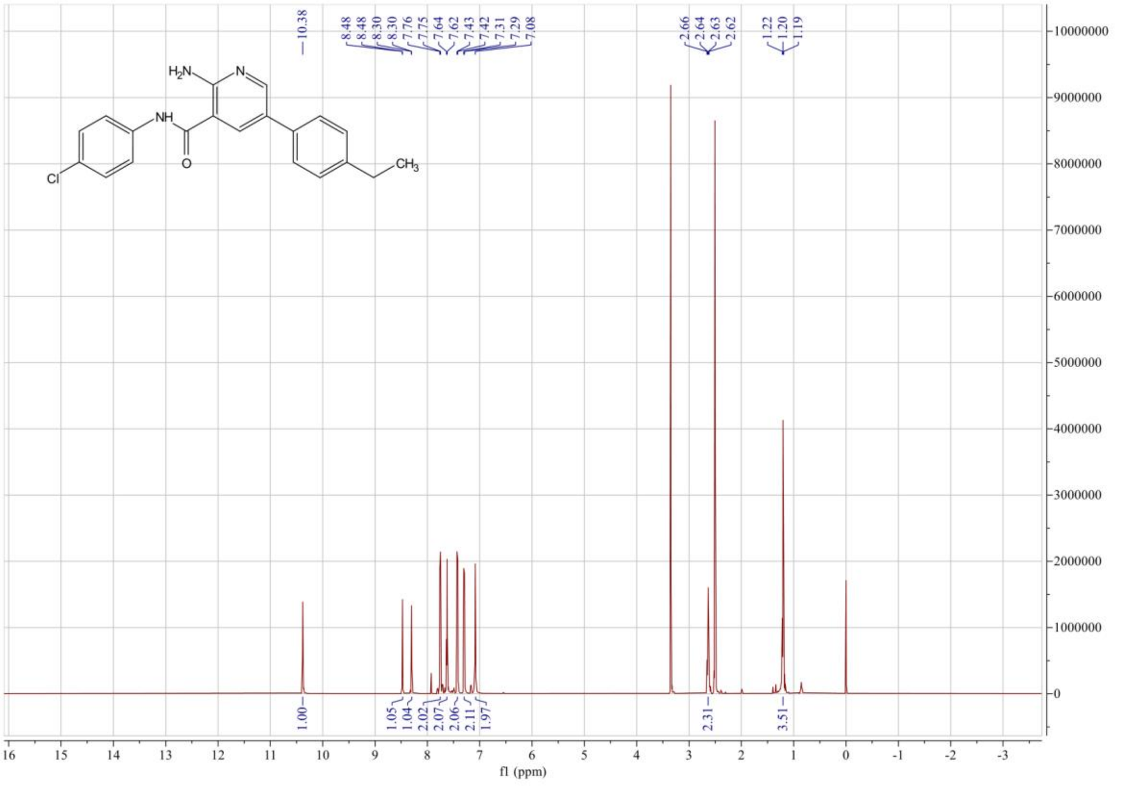
10.38
8.48
8.48
8.30
8.30
7.76
7.75
7.64
7.62
7.43
7.42
7.31
7.29
7.08
2.66
2.64
2.63
2.62
1.22
1.20
1.19
H_2N
N
NH
O
Cl
CH_3
1.00
1.05
1.04
2.02
2.07
2.06
2.11
1.97
2.31
3.51
f1 (ppm)

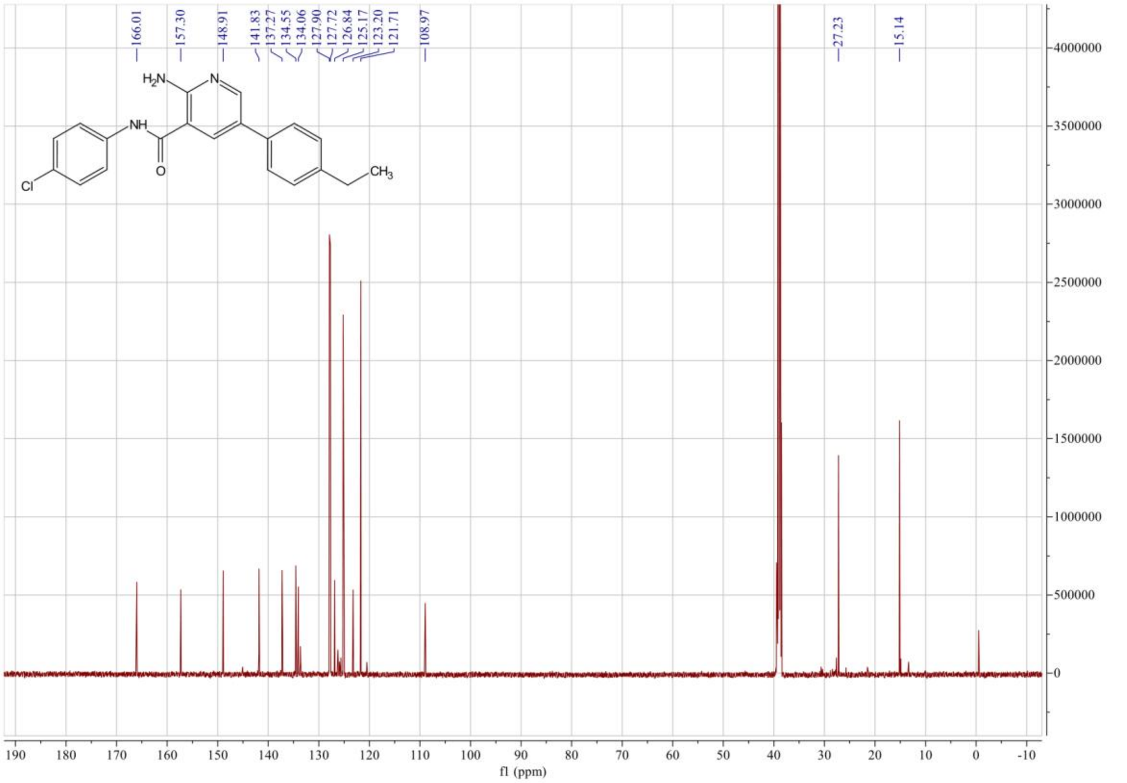
166.01
157.30
148.91
141.83
137.27
134.55
134.06
127.90
127.72
126.84
125.17
123.20
121.71
108.97
27.23
15.14
H_2N
N
NH
O
Cl
CH_3
f1 (ppm)

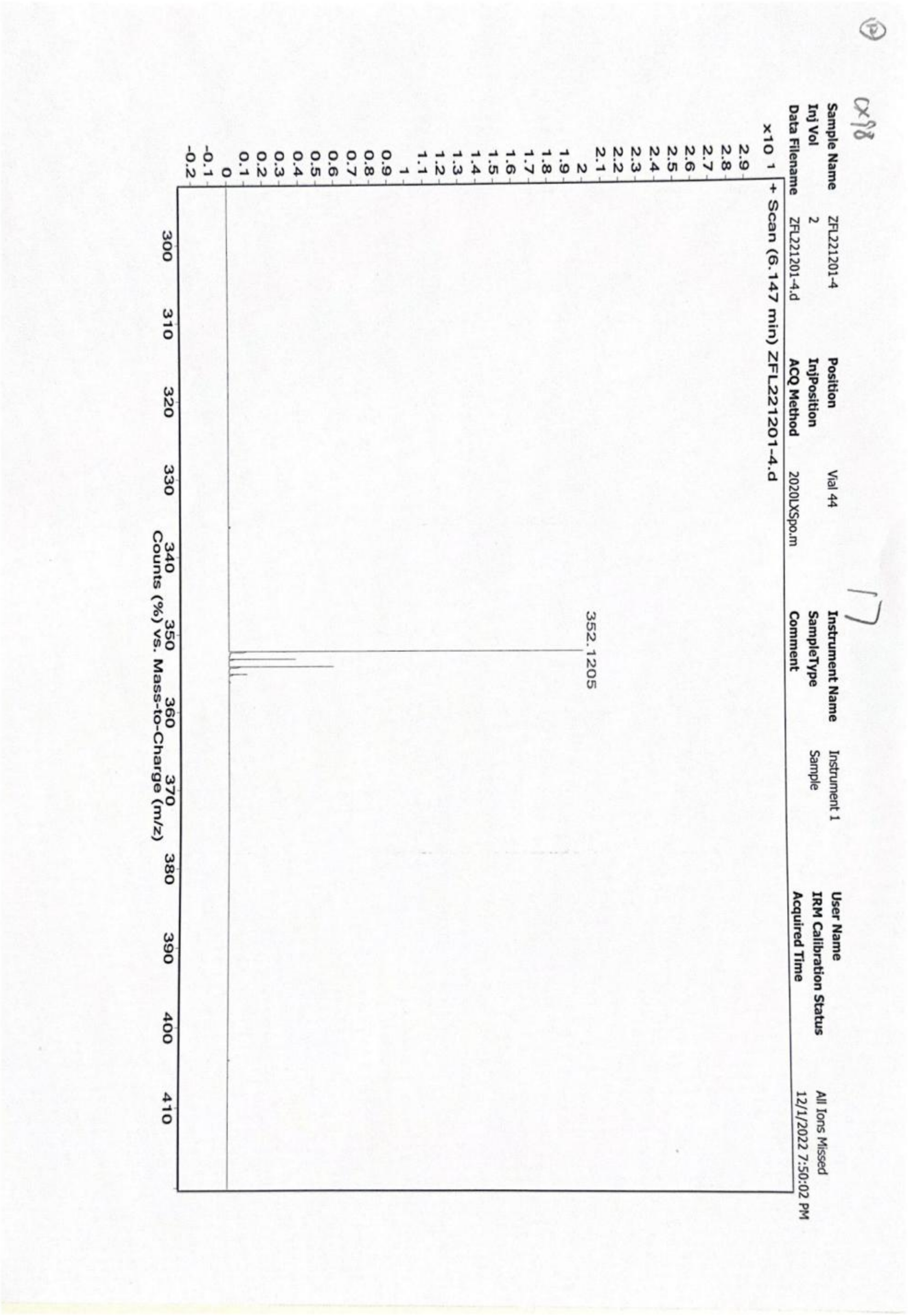

Figure S12. The ^{1}H NMR,^{13}C NMR and ESI-HRMS spectra of compound **17**

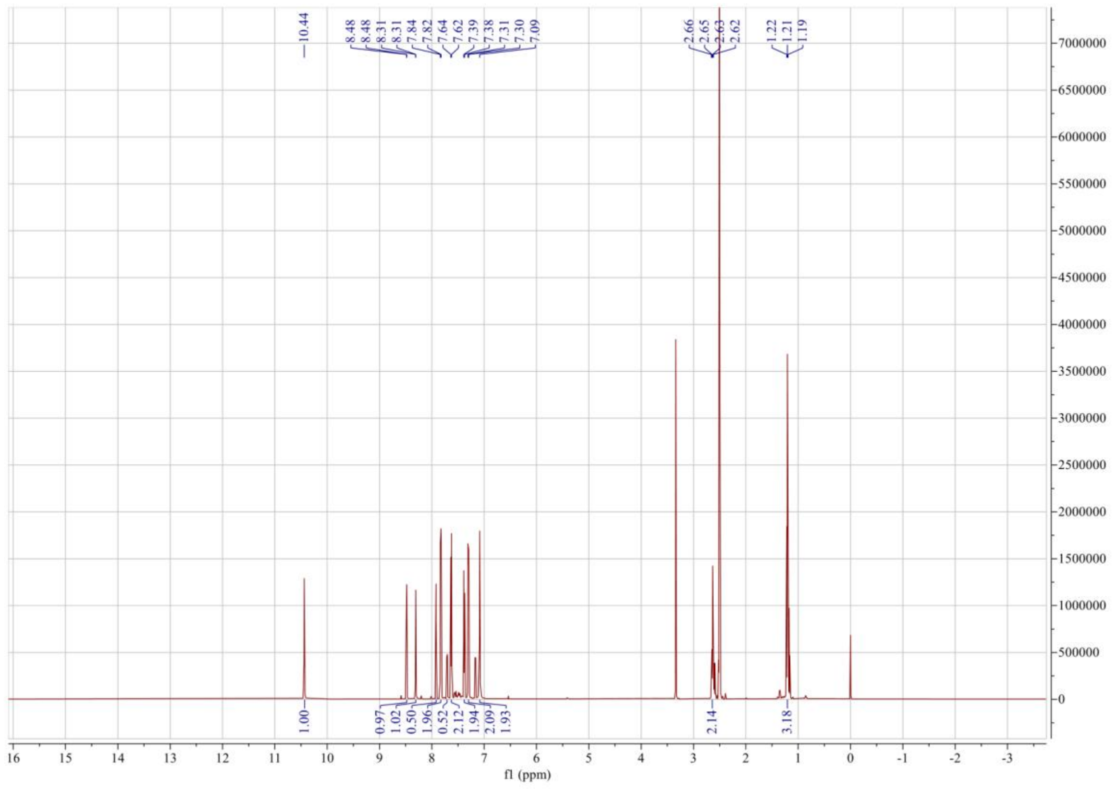

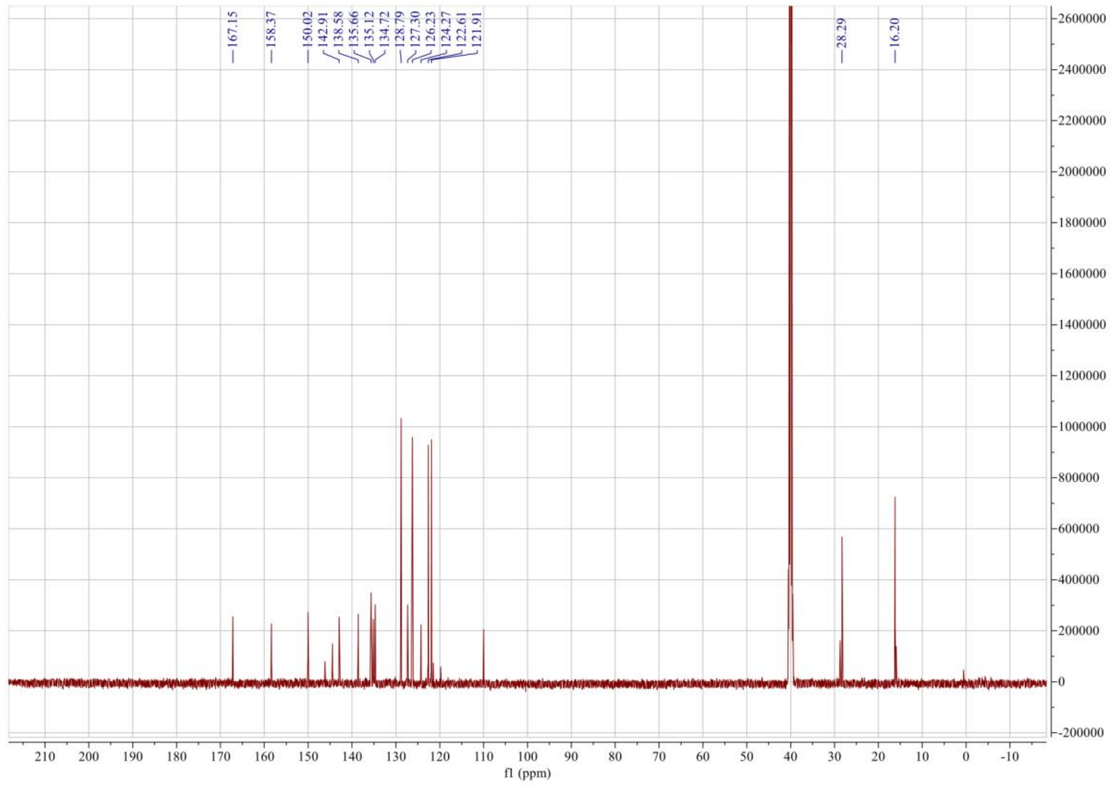

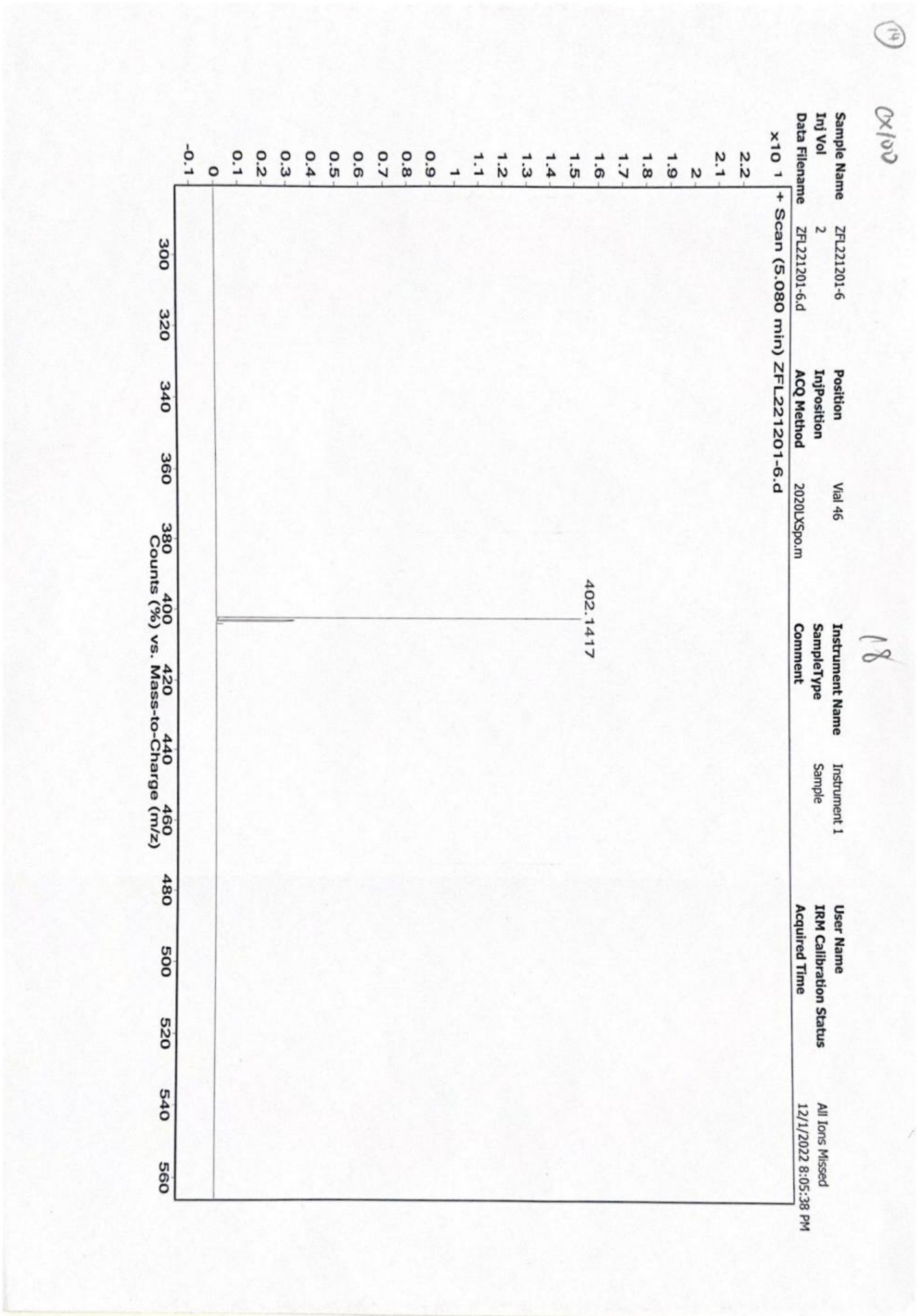

Figure S13. The ^{1}H NMR,^{13}C NMR and ESI-HRMS spectra of compound **18**

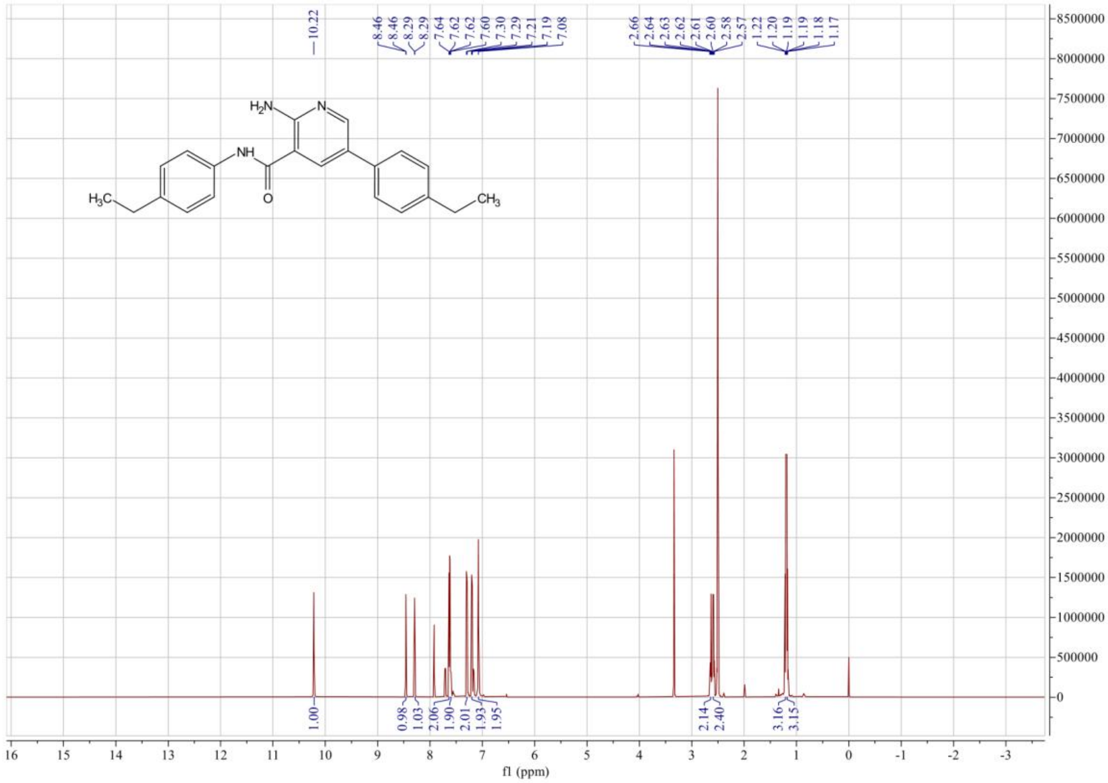
10.22
8.46
8.46
8.29
8.29
7.64
7.62
7.62
7.60
7.30
7.29
7.21
7.19
7.08
2.66
2.64
2.63
2.62
2.61
2.60
2.58
2.57
1.22
1.20
1.19
1.19
1.18
1.17
1.00
0.98
1.03
2.06
1.90
2.01
1.93
1.95
2.14
2.40
3.16
3.15
f1 (ppm)

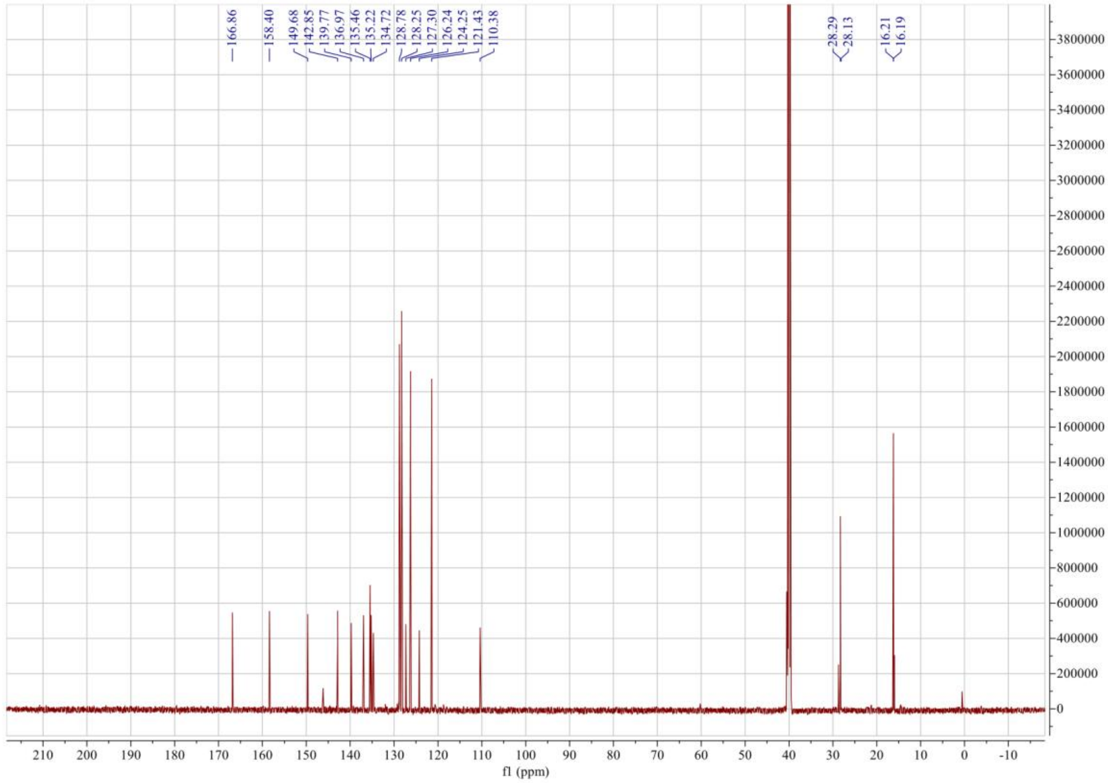
166.86
158.40
149.68
142.85
139.77
136.97
135.46
135.22
134.72
128.78
128.25
127.30
126.24
124.25
121.43
110.38
28.29
28.13
16.21
16.19
f1 (ppm)

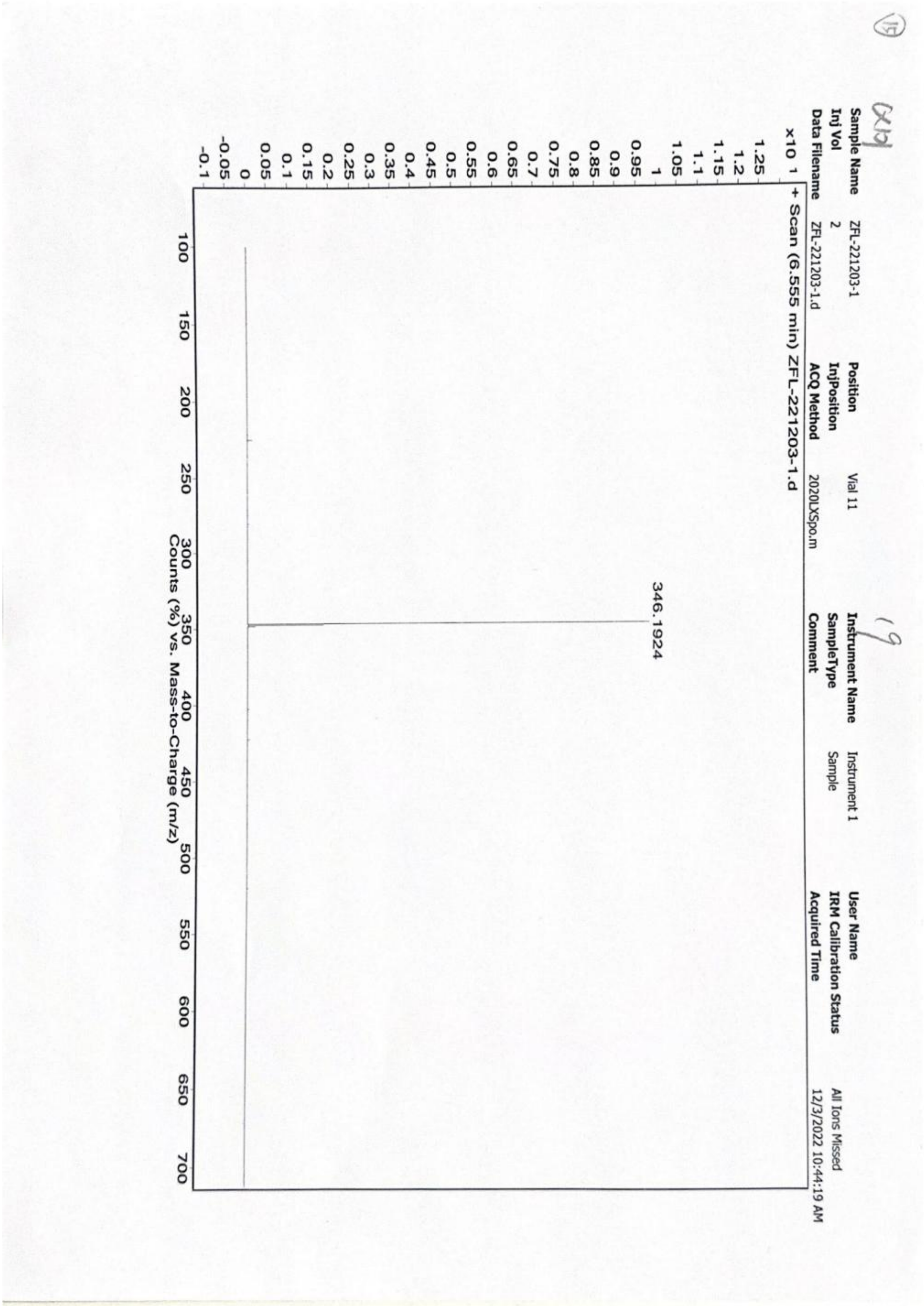

Figure S14. The ^{1}H NMR,^{13}C NMR and ESI-HRMS spectra of compound **19**

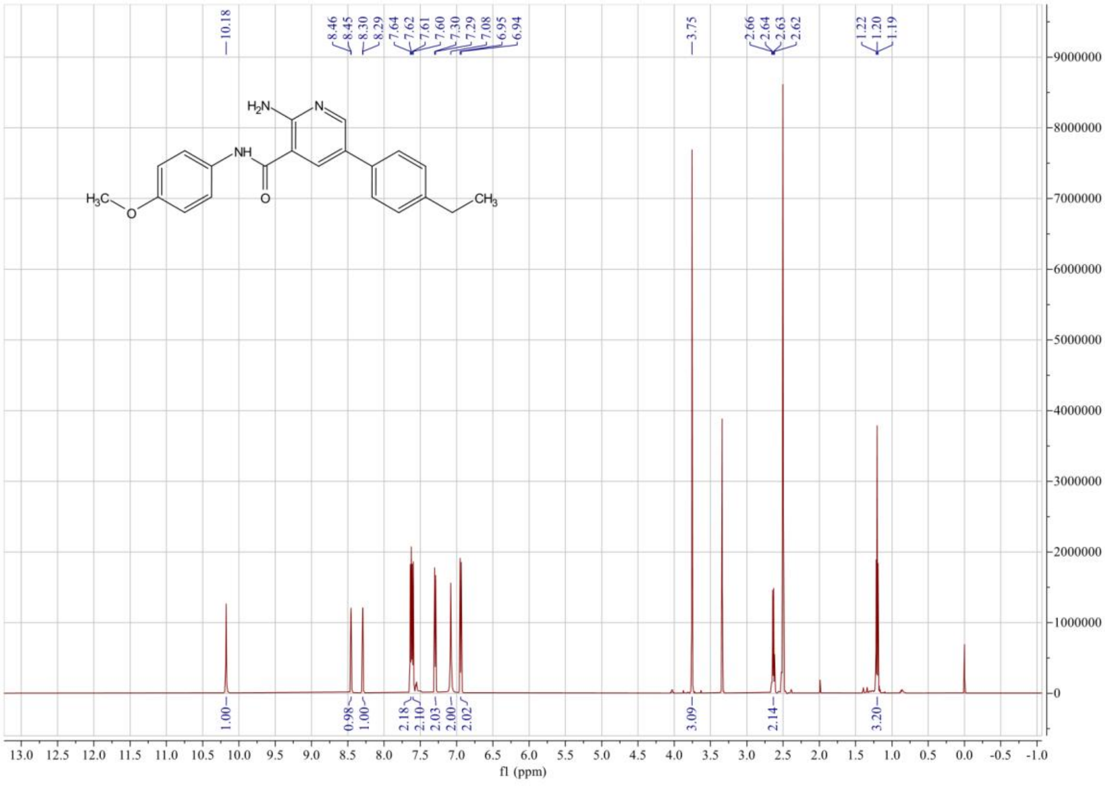

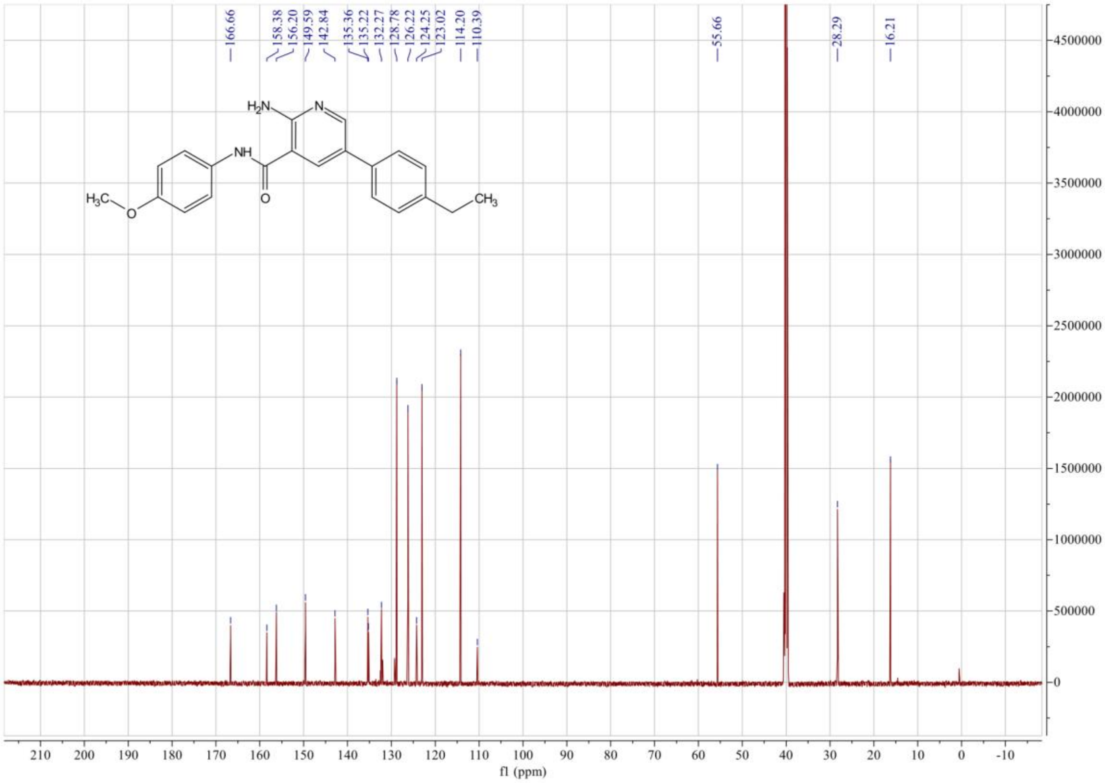

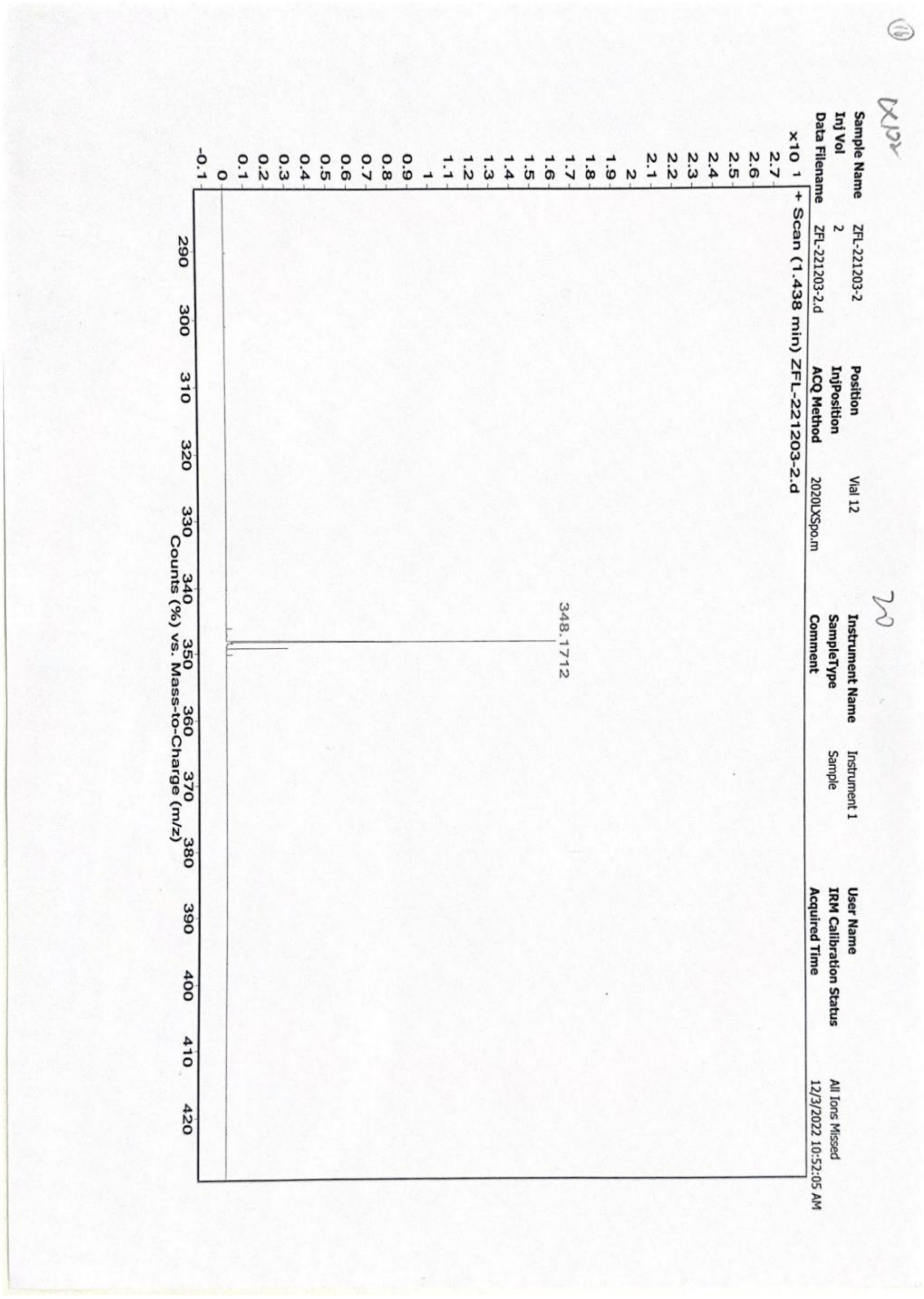

Figure S15. The ^{1}H NMR, ^{13}C NMR and ESI-HRMS spectra of compound **20**

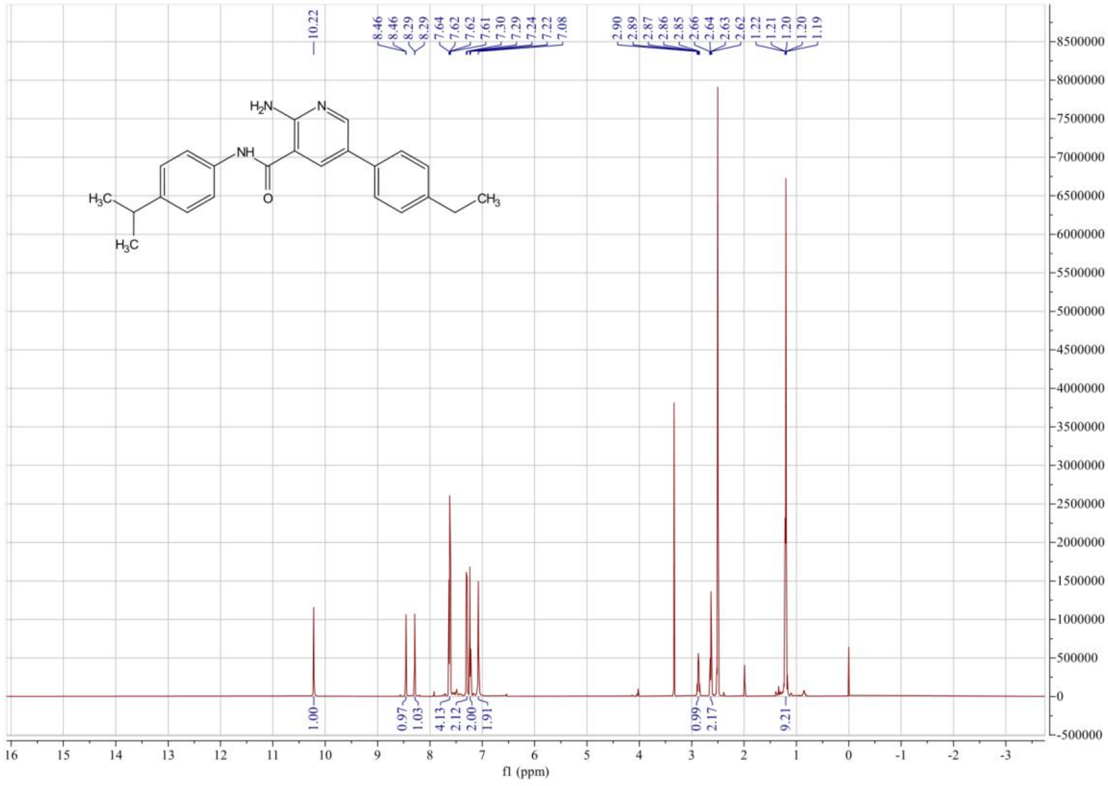
fl (ppm)

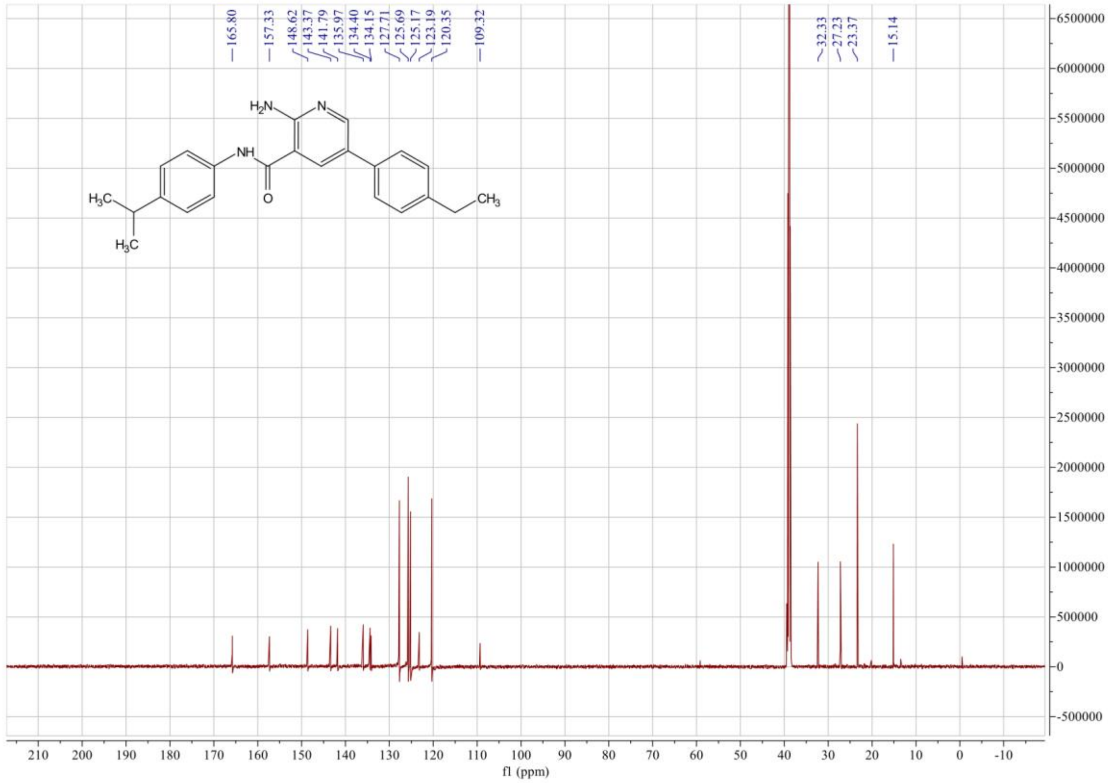
fl (ppm)

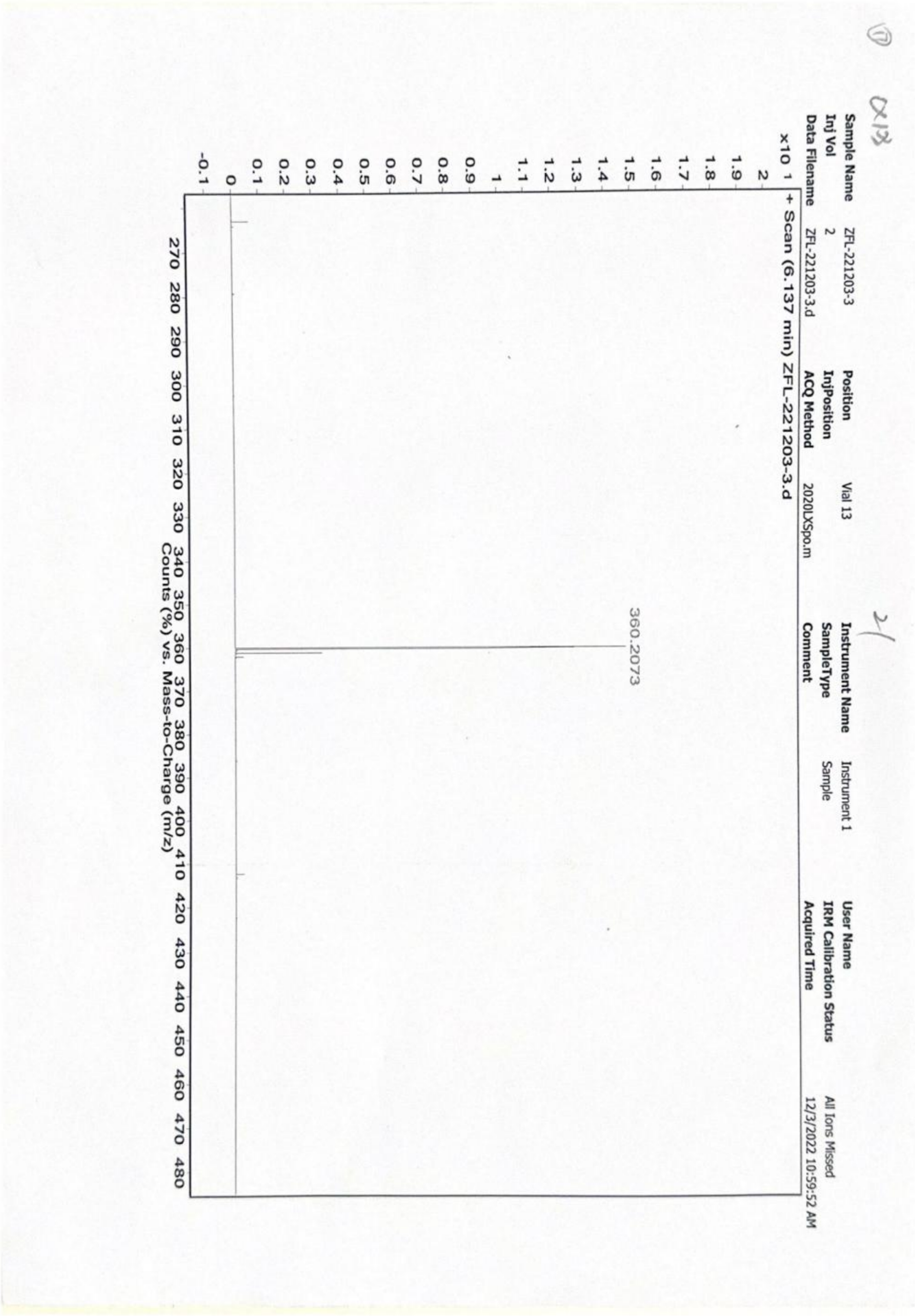

Figure S16. The ^{1}H NMR,^{13}C NMR and ESI-HRMS spectra of compound **21**

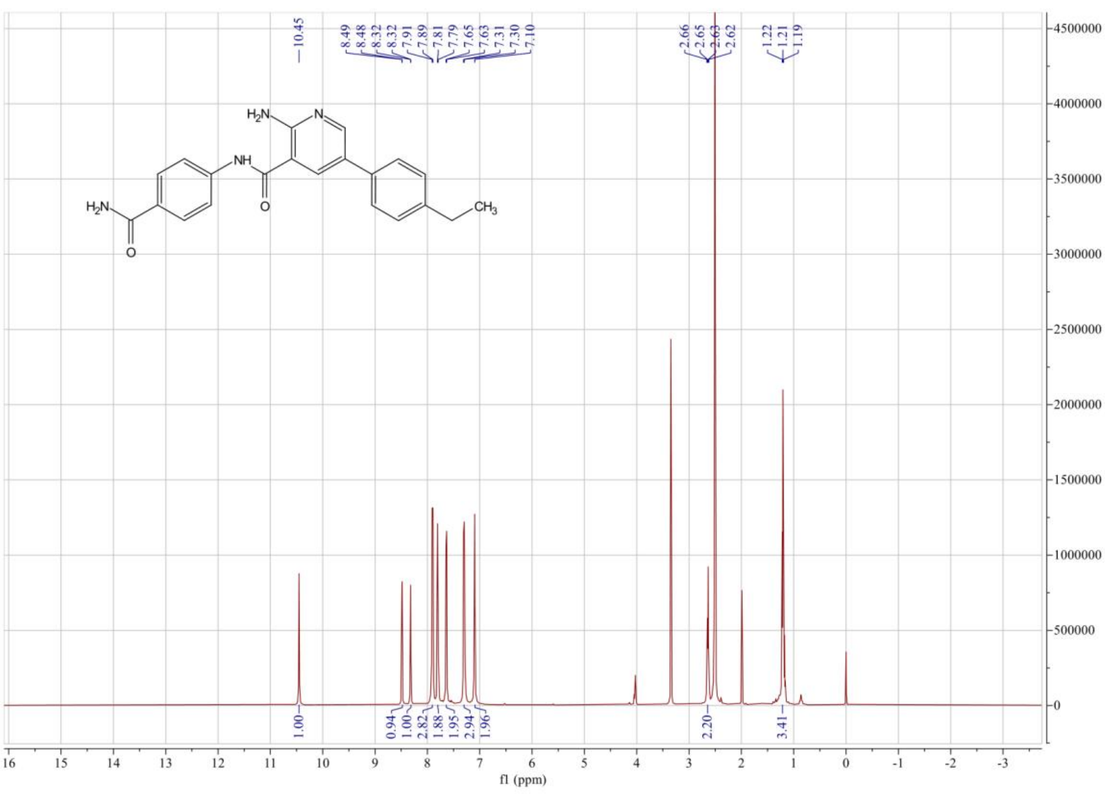

10.45
8.49
8.48
8.32
8.32
7.91
7.89
7.81
7.79
7.65
7.63
7.31
7.30
7.10
2.66
2.65
2.63
2.62
1.22
1.21
1.19
1.00
0.94
1.00
2.82
1.88
1.95
2.94
1.96
2.20
3.41
f1 (ppm)

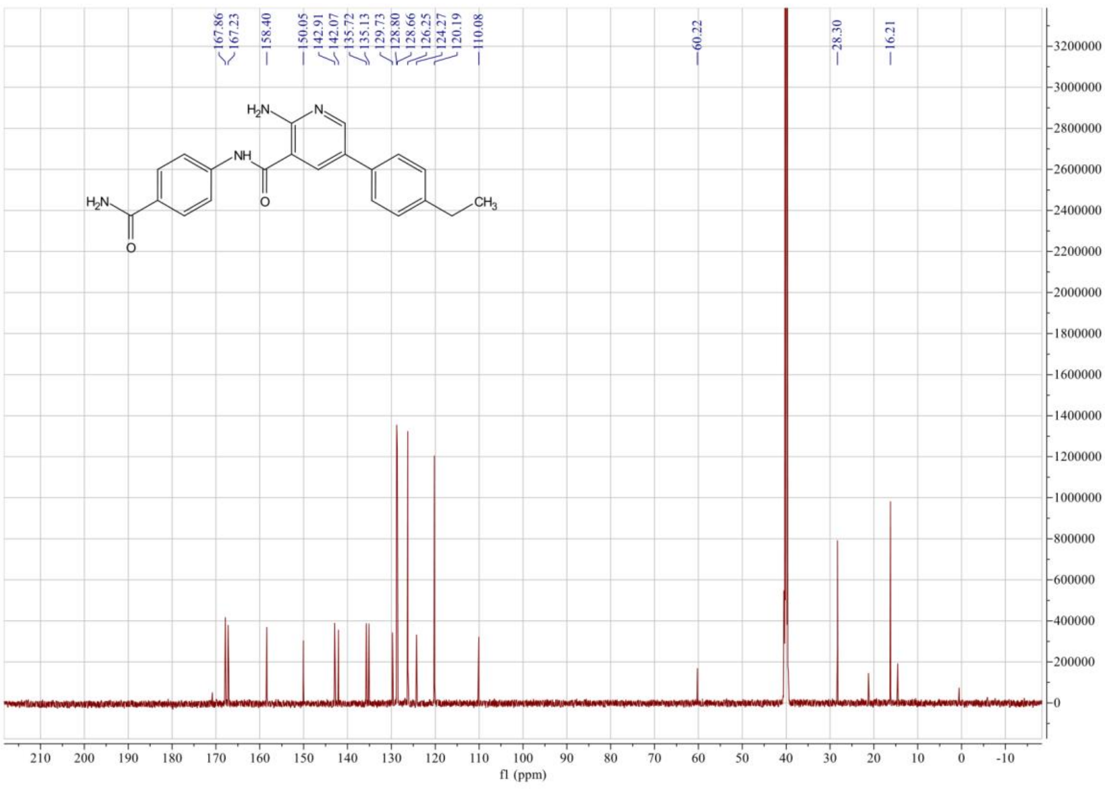

167.86
167.23
158.40
150.05
142.91
142.07
135.72
135.13
129.73
128.80
128.66
126.25
124.27
120.19
110.08
60.22
28.30
16.21
f1 (ppm)

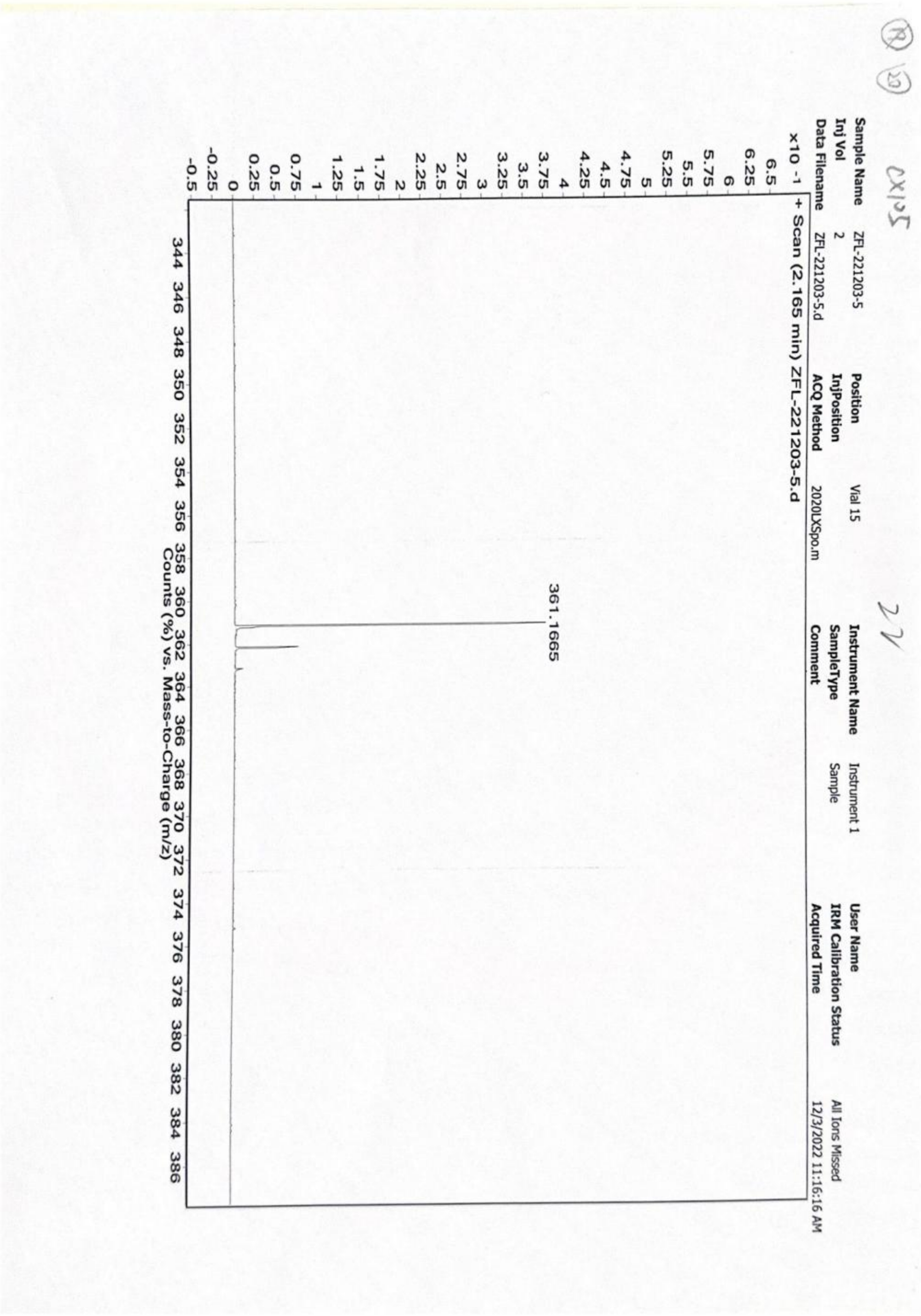

Figure S17. The ^{1}H NMR, ^{13}C NMR and ESI-HRMS spectra of compound **22**

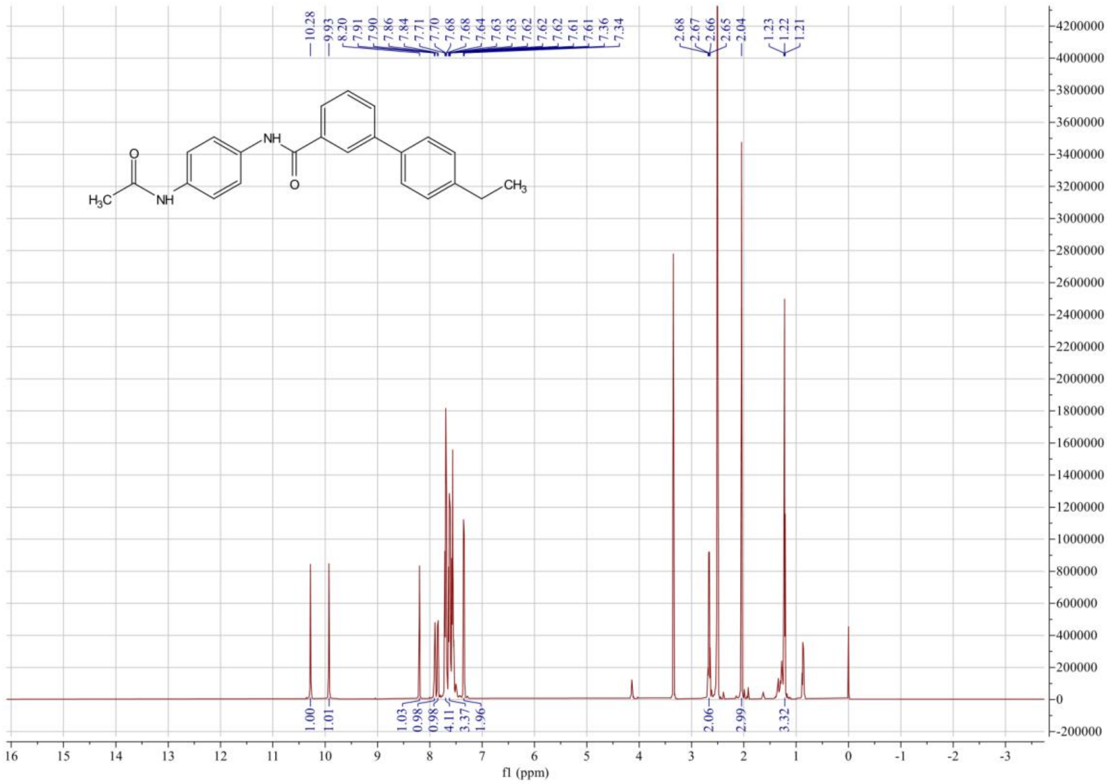

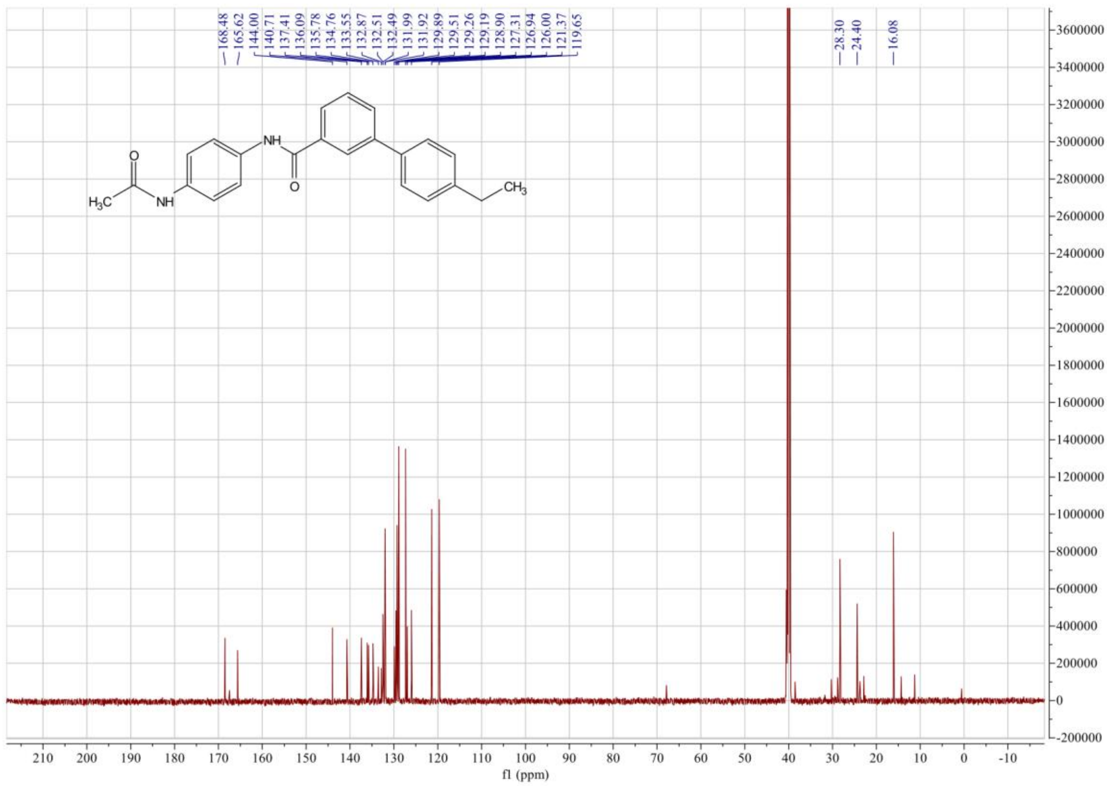

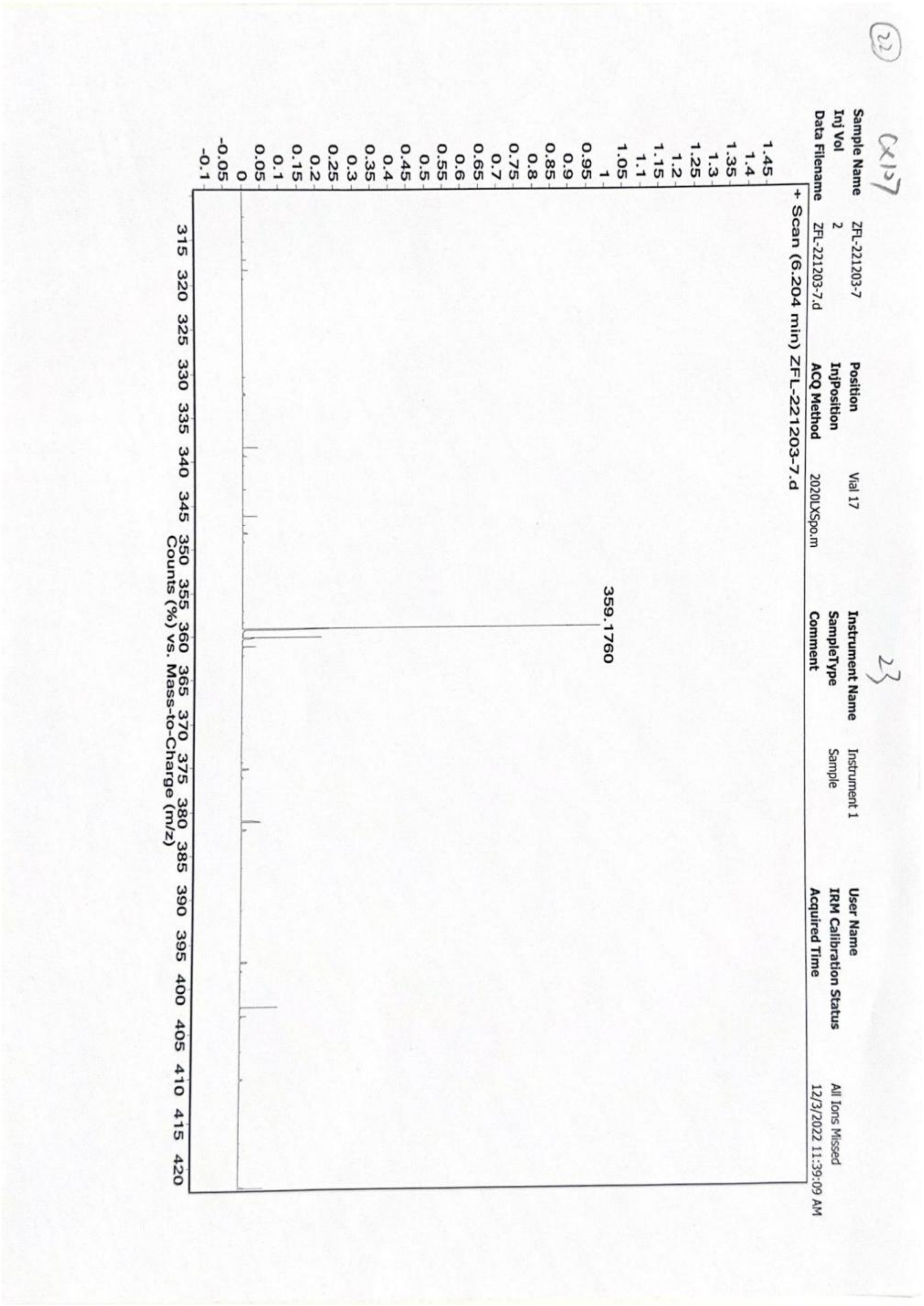

Figure S18. The ^{1}H NMR, ^{13}C NMR and ESI-HRMS spectra of compound **23**

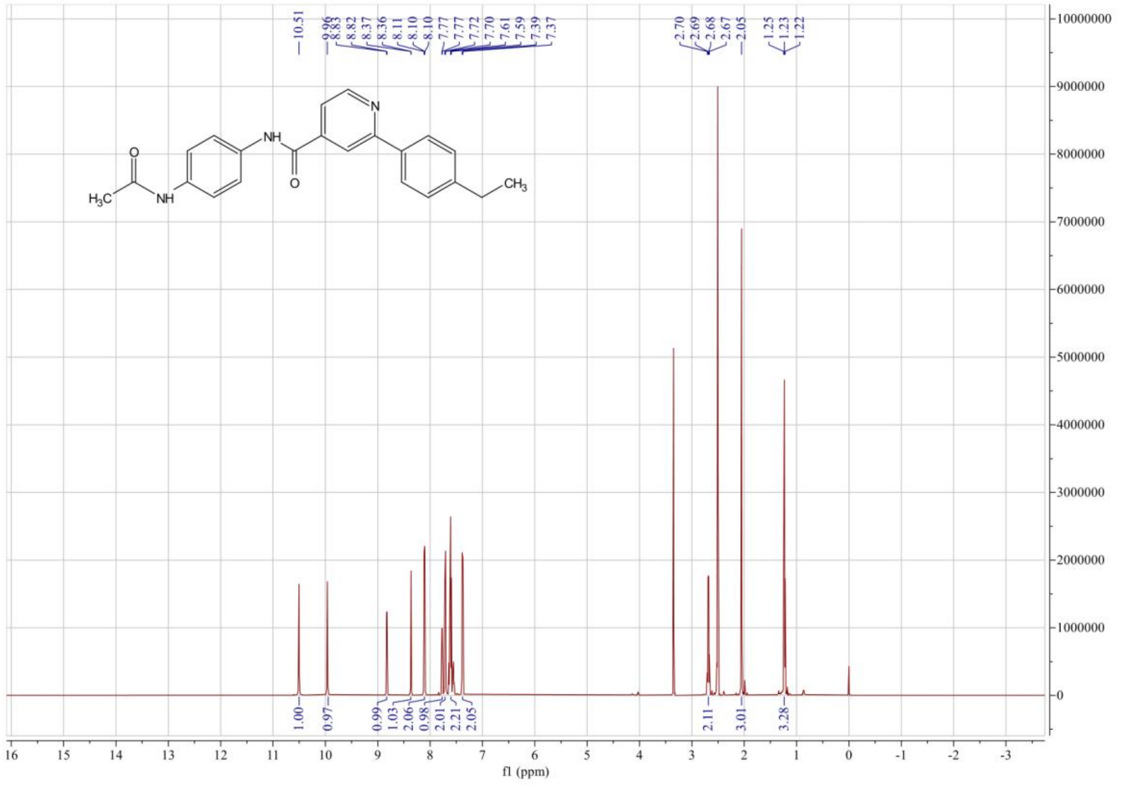
10.51
9.96
8.83
8.82
8.37
8.36
8.11
8.10
8.10
7.77
7.77
7.72
7.70
7.61
7.59
7.39
7.37
2.70
2.69
2.68
2.67
2.05
1.25
1.23
1.22
NH
N
O
H3C
NH
CH3
1.00
0.97
0.99
1.03
2.06
0.98
2.01
2.21
2.05
2.11
3.01
3.28
f1 (ppm)

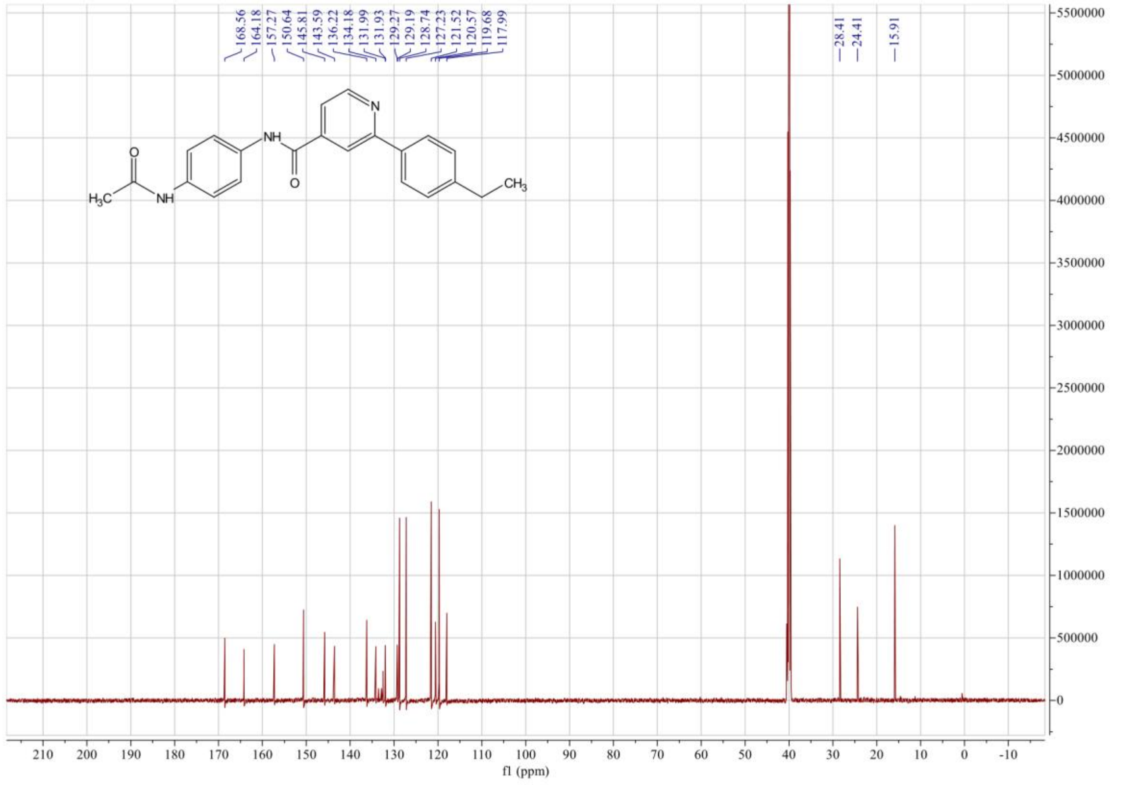
168.56
164.18
157.27
150.64
145.81
143.59
136.22
134.18
131.99
131.93
129.27
129.19
128.74
127.23
121.52
120.57
119.68
117.99
28.41
24.41
15.91
NH
N
O
H3C
NH
CH3
f1 (ppm)

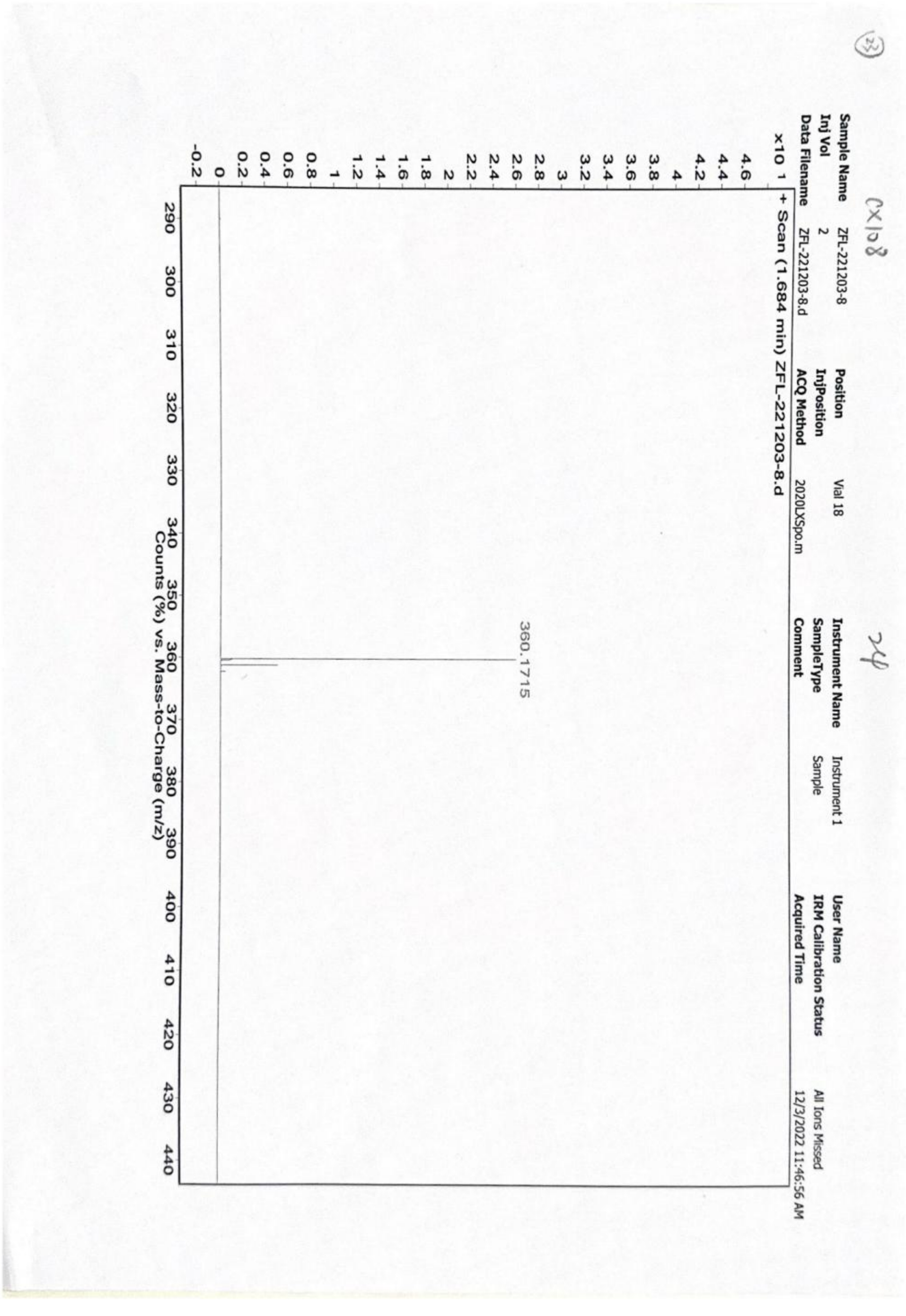

Figure S19. The ^{1}H NMR,^{13}C NMR and ESI-HRMS spectra of compound **24**

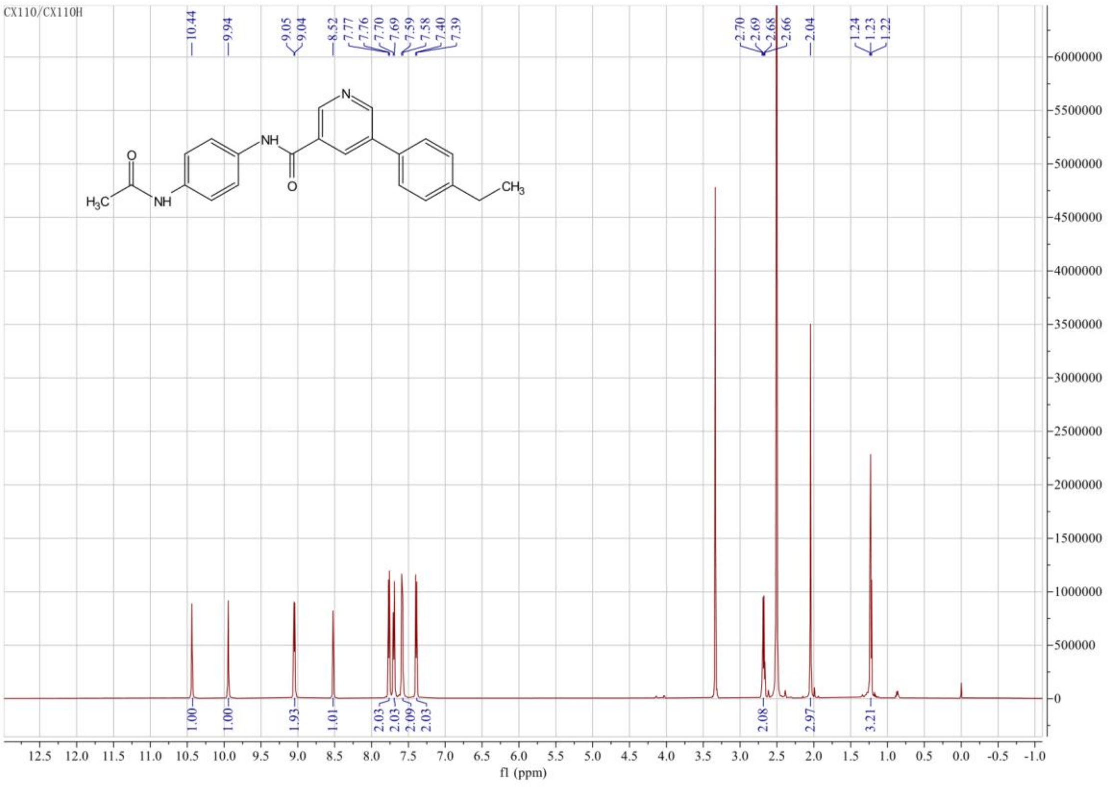
CX110/CX110H
10.44
9.94
9.05
9.04
8.52
7.77
7.76
7.70
7.69
7.59
7.58
7.40
7.39
2.70
2.69
2.68
2.66
2.04
1.24
1.23
1.22
1.00
1.00
1.93
1.01
2.03
2.03
2.09
2.03
2.08
2.97
3.21
f1 (ppm)

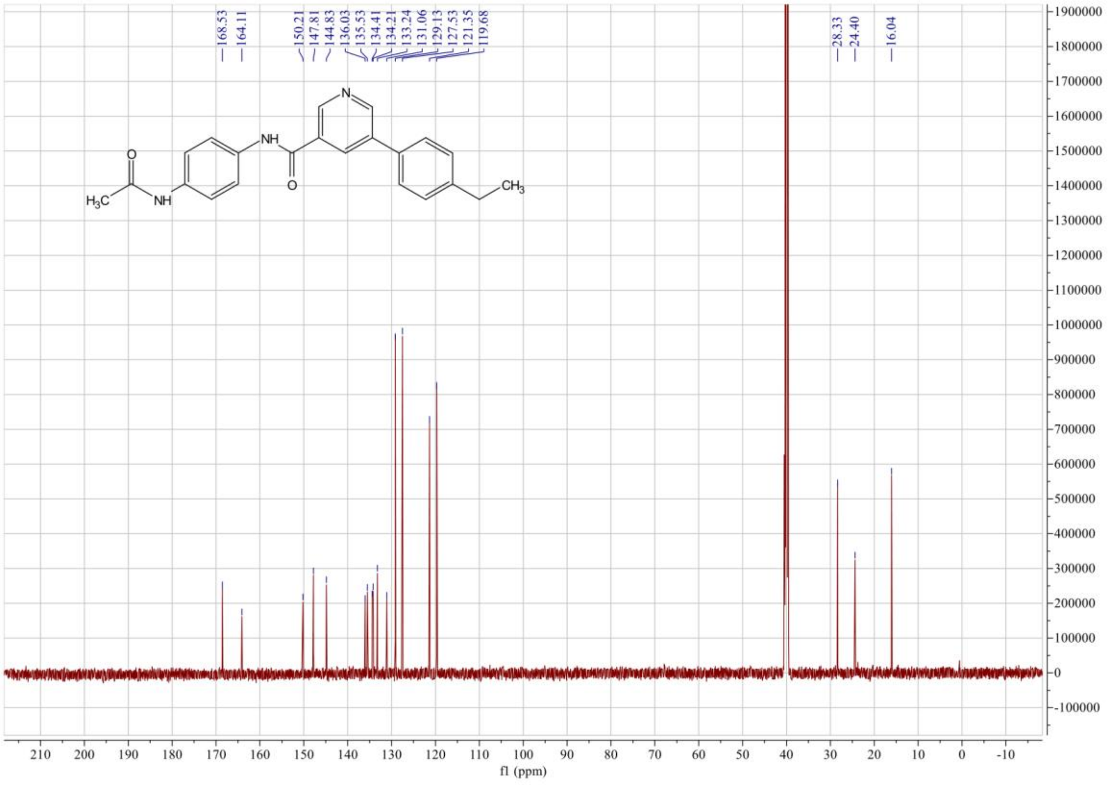
168.53
164.11
150.21
147.81
144.83
136.03
135.53
134.41
134.21
133.24
131.06
129.13
127.53
121.35
119.68
28.33
24.40
16.04
f1 (ppm)

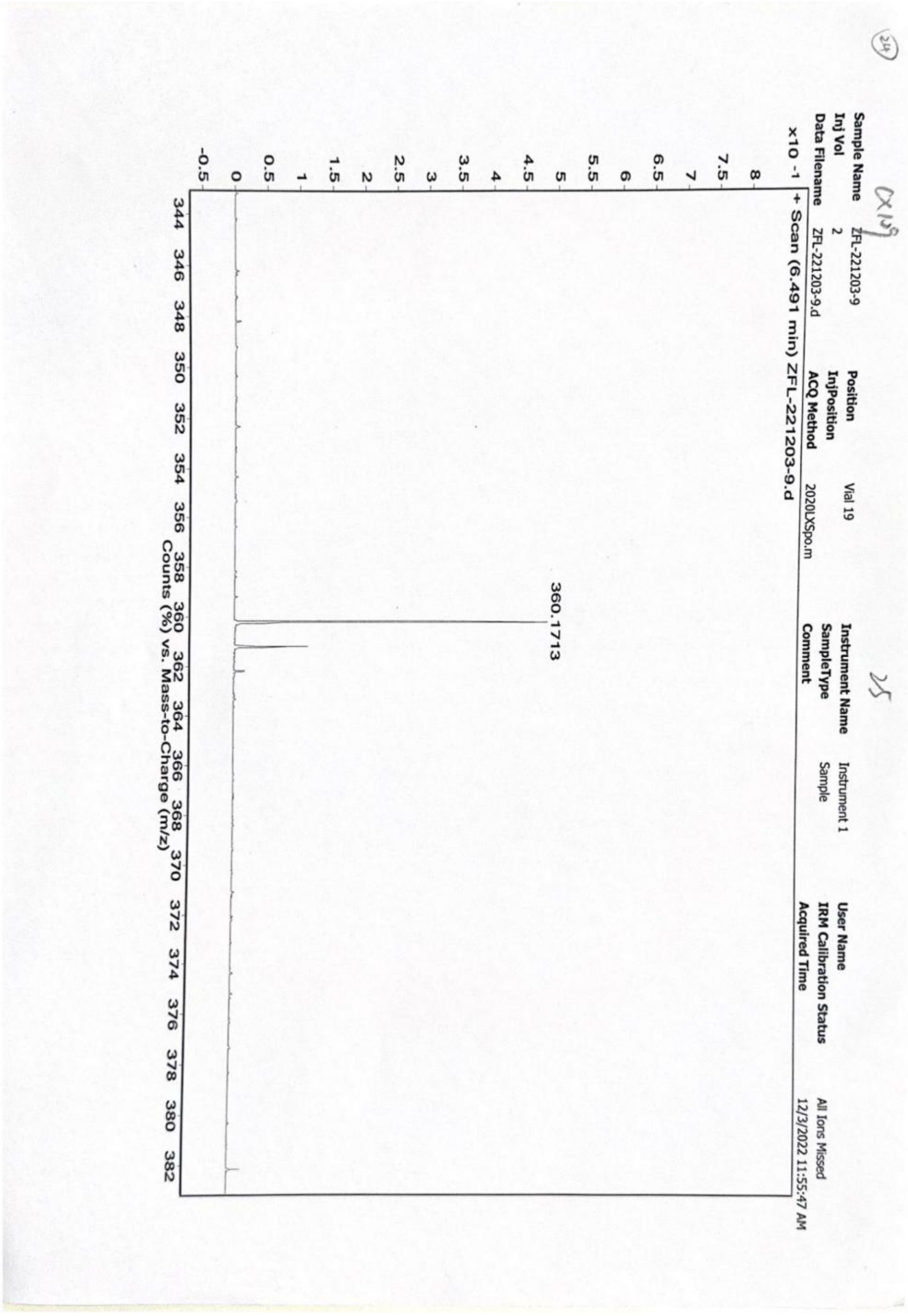

Figure S20. The ^{1}H NMR,^{13}C NMR and ESI-HRMS spectra of compound **25**

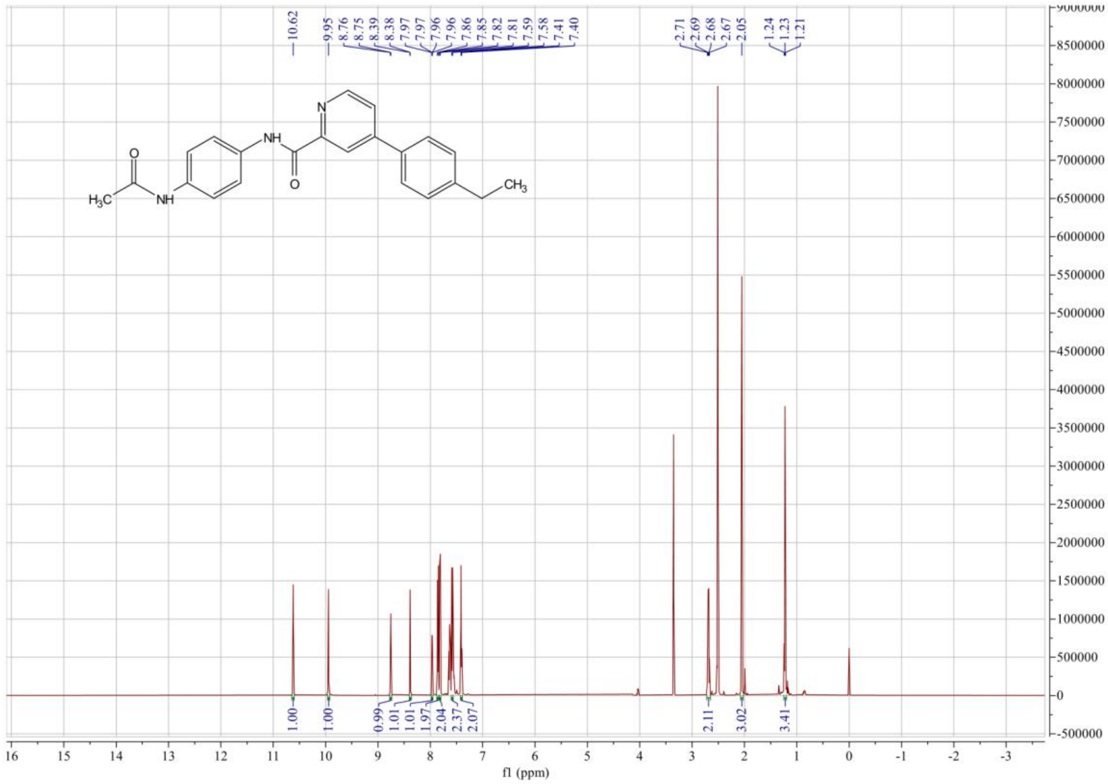
10.62
9.95
8.76
8.75
8.39
8.38
7.97
7.97
7.96
7.96
7.86
7.85
7.82
7.81
7.59
7.58
7.41
7.40
2.71
2.69
2.68
2.67
2.05
1.24
1.23
1.21
NH
N
O
H3C
NH
O
CH3
1.00
1.00
0.99
1.01
1.01
1.97
2.04
2.37
2.07
2.11
3.02
3.41
f1 (ppm)

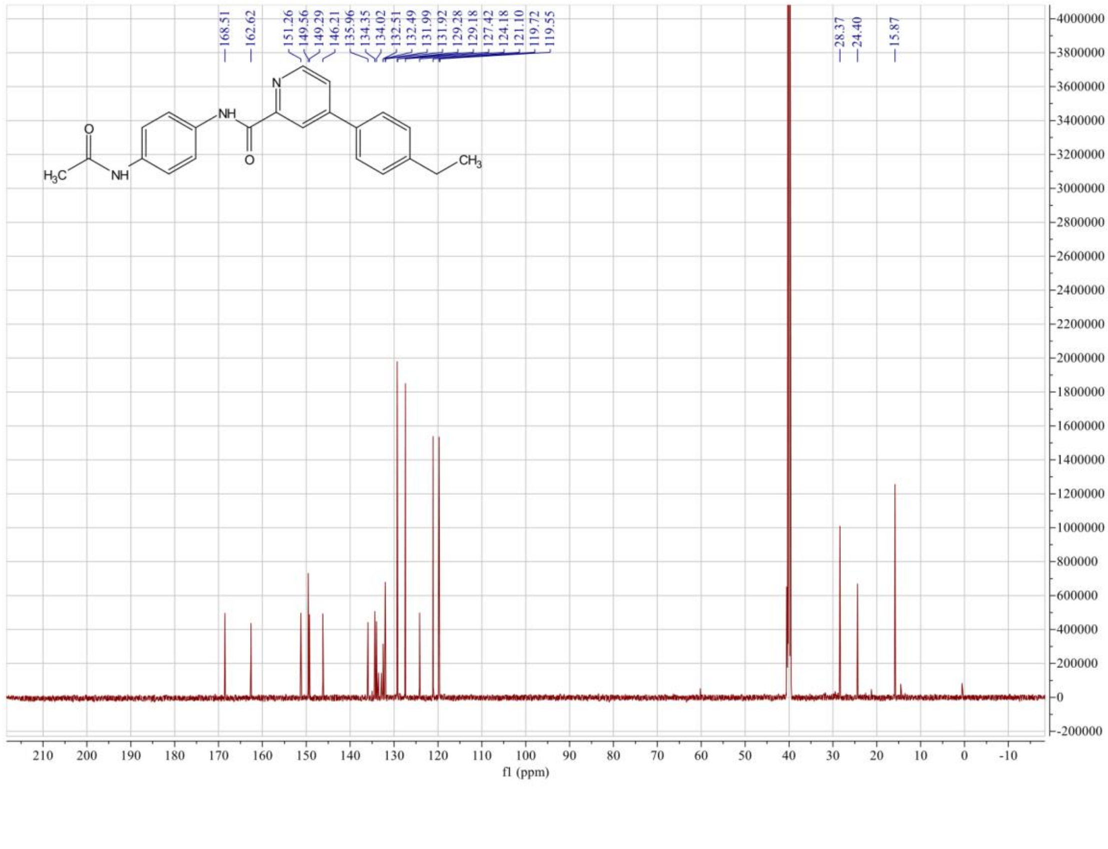
168.51
162.62
151.26
149.56
149.29
146.21
135.96
134.35
134.02
132.51
132.49
131.99
131.92
129.28
129.18
127.42
124.18
121.10
119.72
119.55
28.37
24.40
15.87
NH
N
O
H3C
NH
O
CH3
f1 (ppm)

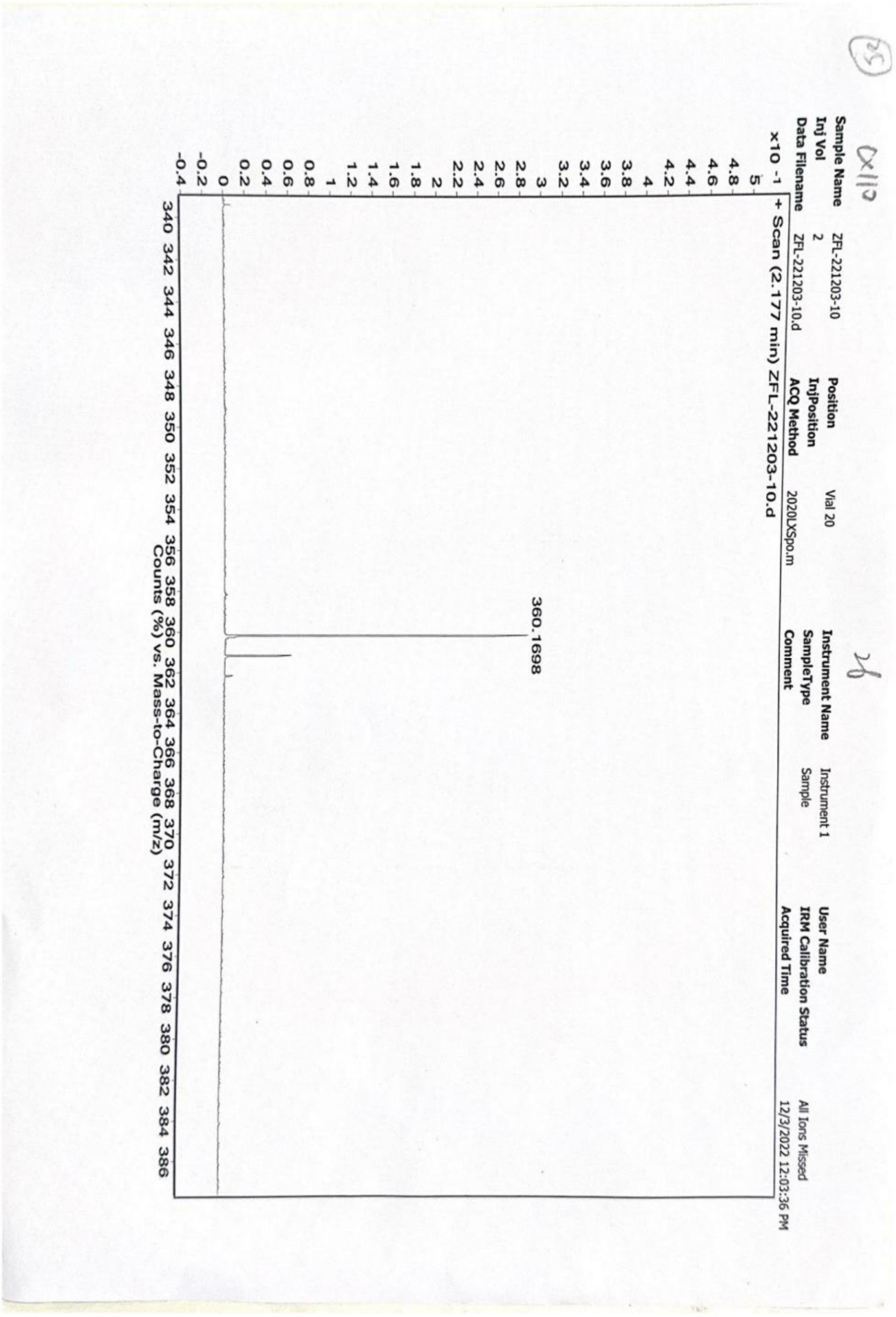

Figure S21. The ^{1}H NMR, ^{13}C NMR and ESI-HRMS spectra of compound **26**

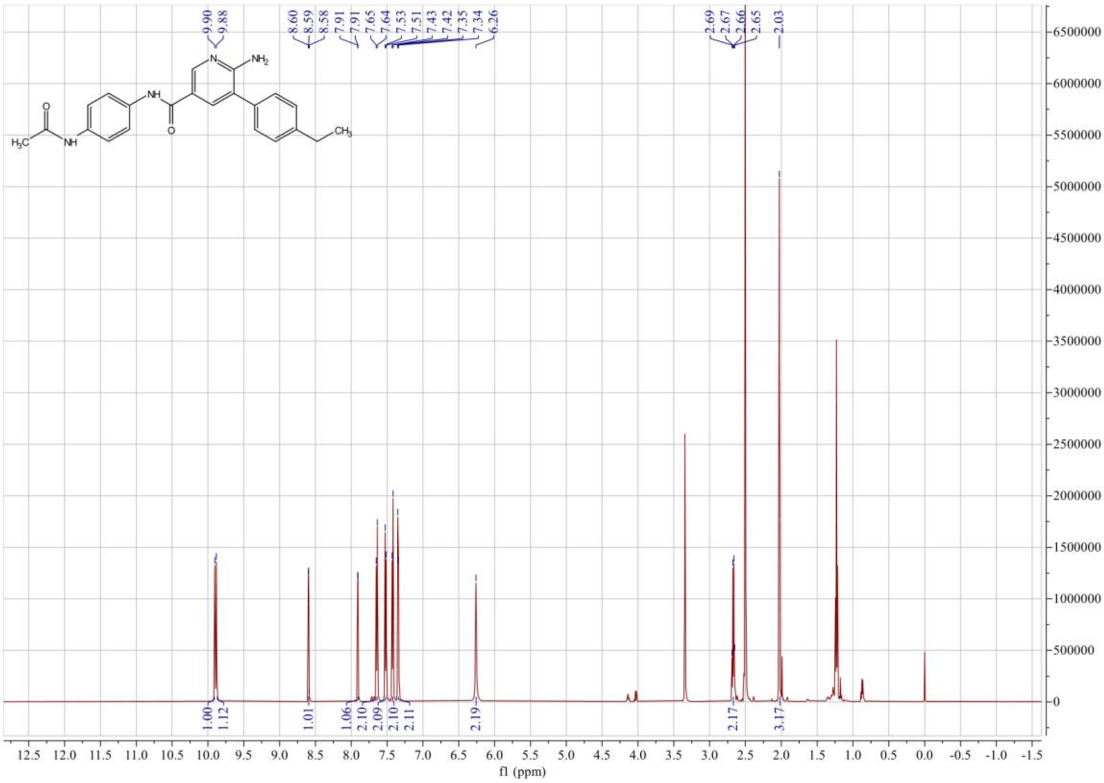
9.90
9.88
8.60
8.59
8.58
7.91
7.91
7.65
7.64
7.53
7.51
7.43
7.42
7.35
7.34
6.26
2.69
2.67
2.66
2.65
2.03
1.00
1.12
1.01
1.06
2.10
2.09
2.10
2.11
2.19
2.17
3.17
NH2
NH
O
H3C
NH
CH3
f1 (ppm)

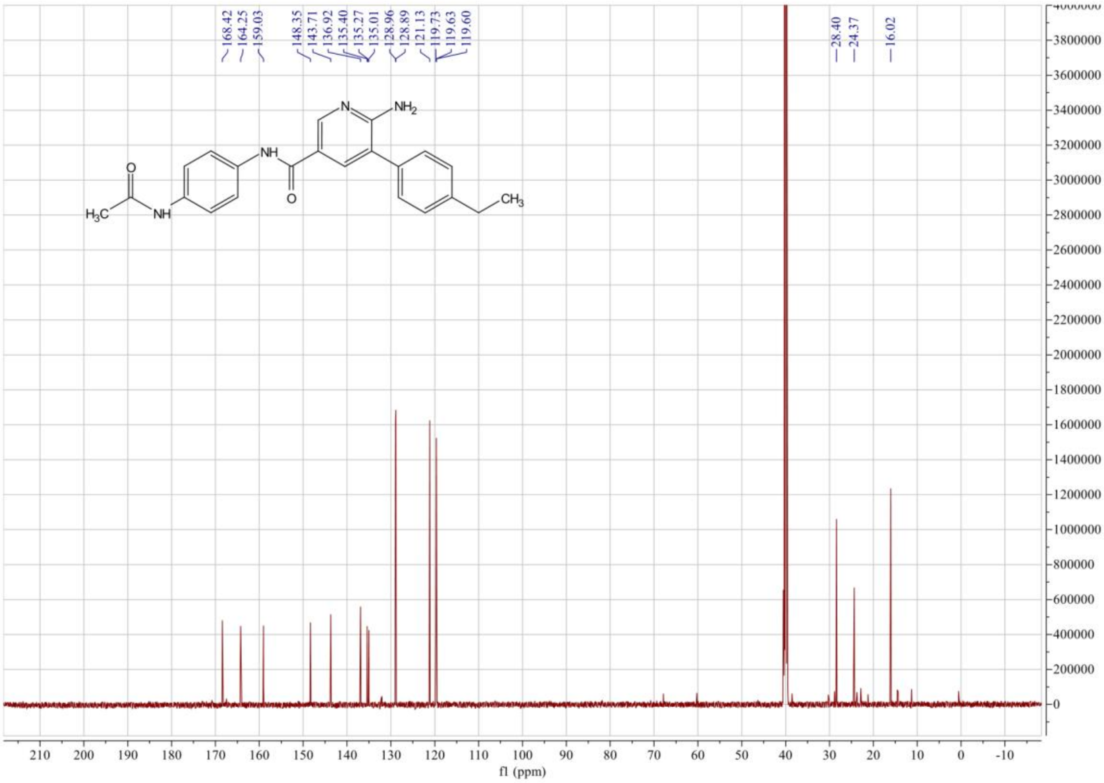
168.42
164.25
159.03
148.35
143.71
136.92
135.40
135.27
135.01
128.96
128.89
121.13
119.73
119.63
119.60
28.40
24.37
16.02
N
NH2
NH
O
H3C
NH
O
CH3
f1 (ppm)

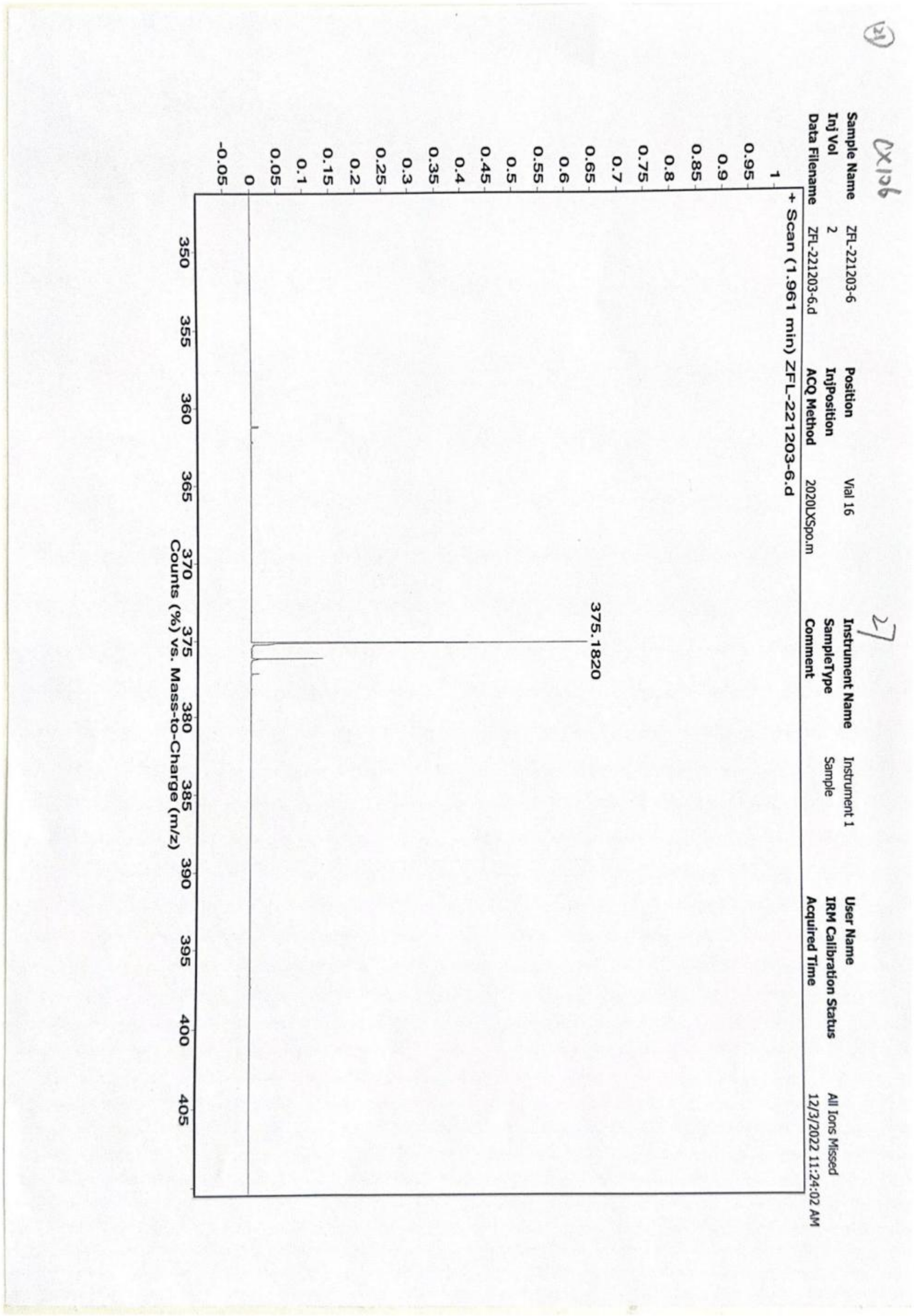

Figure S22. The ^{1}H NMR,^{13}C NMR and ESI-HRMS spectra of compound **27**

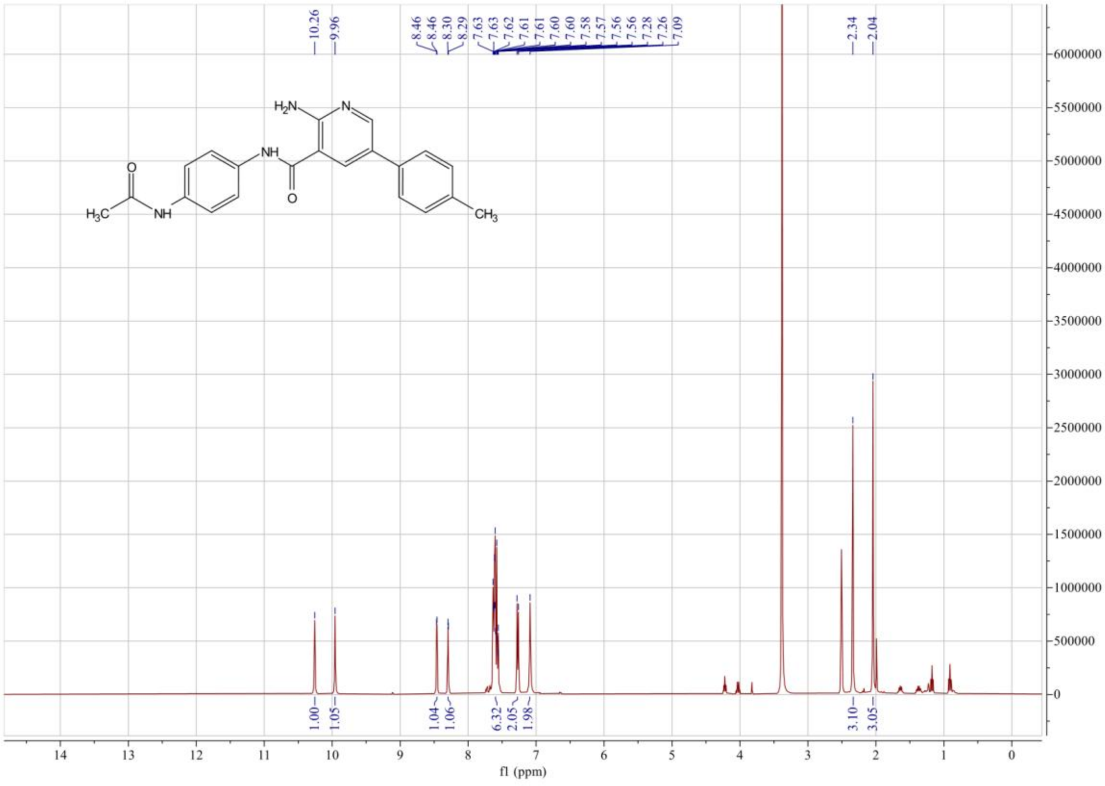
10.26
9.96
8.46
8.46
8.30
8.29
7.63
7.63
7.62
7.61
7.61
7.60
7.60
7.58
7.57
7.56
7.56
7.28
7.26
7.09
2.34
2.04
1.00
1.05
1.04
1.06
6.32
2.05
1.98
3.10
3.05
f1 (ppm)

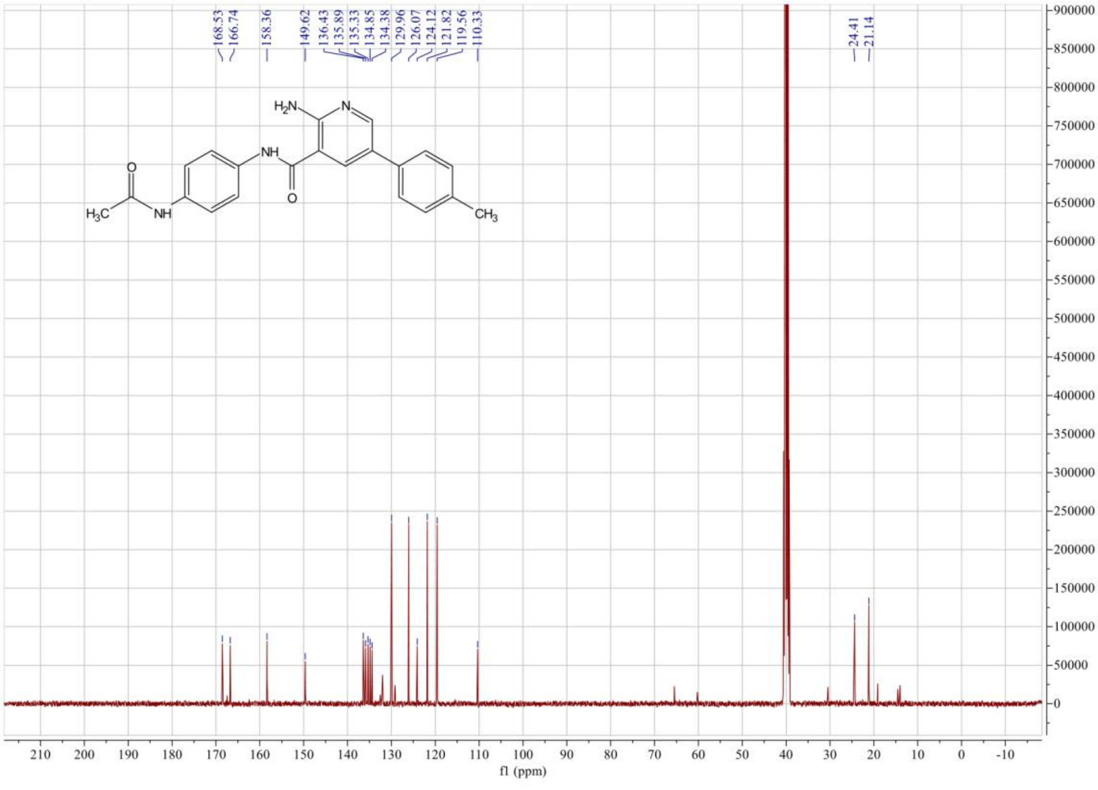
168.53
166.74
158.36
149.62
136.43
135.89
135.33
134.85
134.38
129.96
126.07
124.12
121.82
119.56
110.33
24.41
21.14
f1 (ppm)

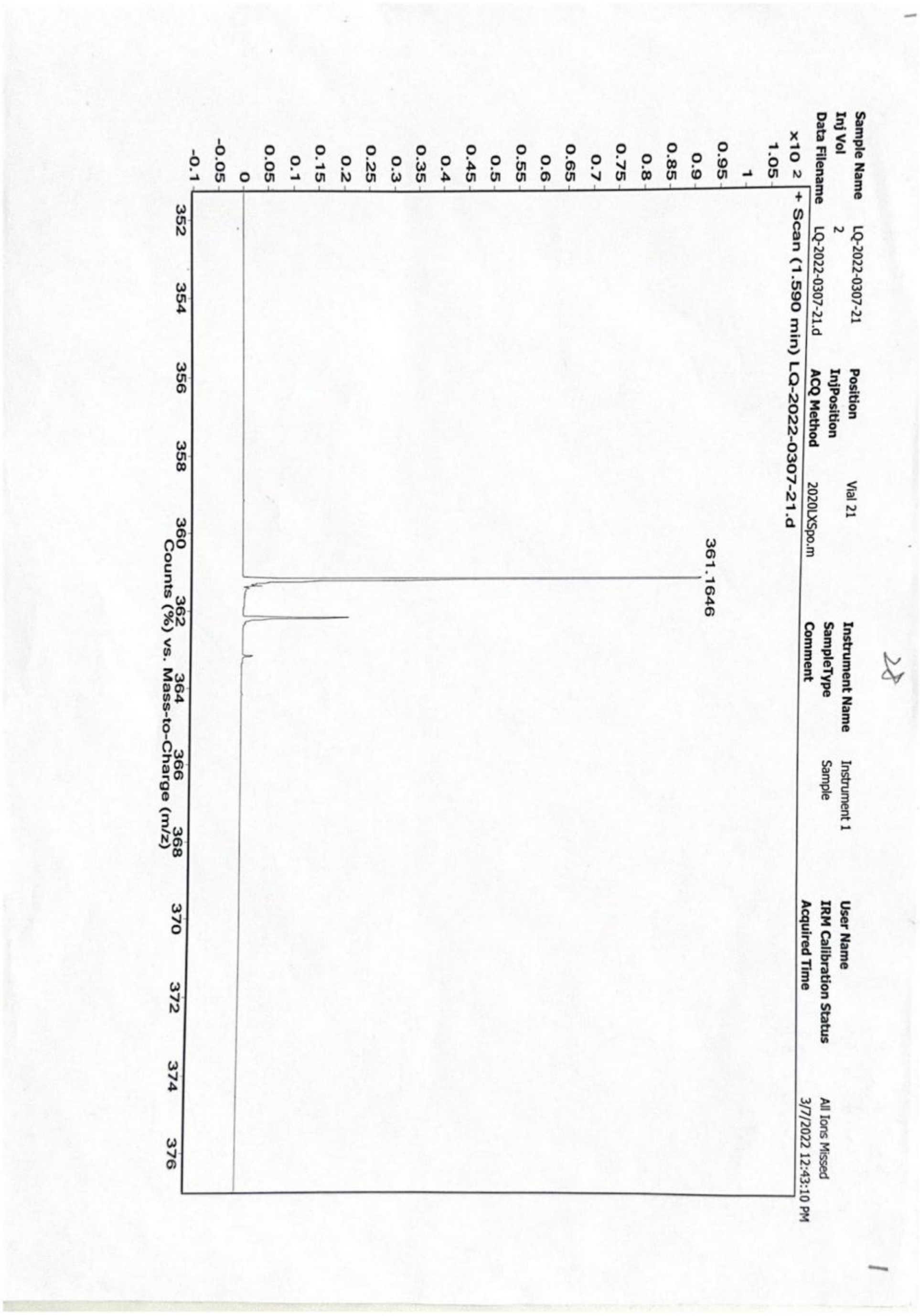

Figure S23. The ^{1}H NMR,^{13}C NMR and ESI-HRMS spectra of compound **28**

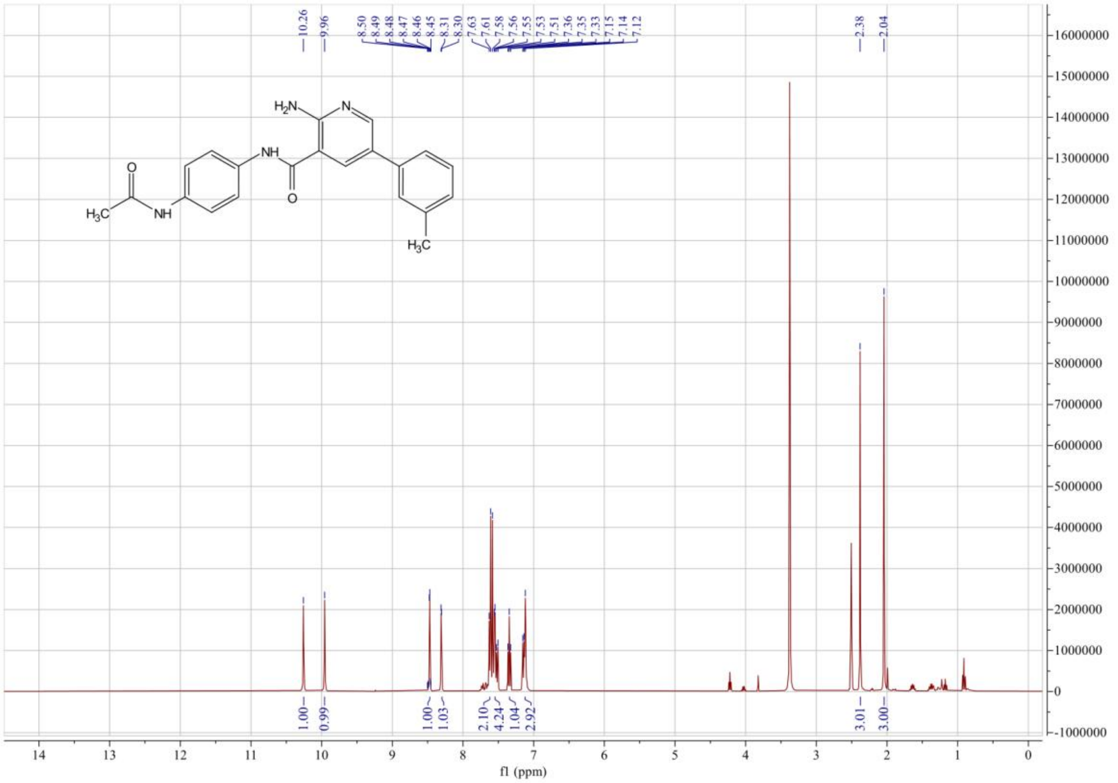
10.26
9.96
8.50
8.49
8.48
8.47
8.46
8.45
8.31
8.30
7.63
7.61
7.58
7.56
7.55
7.53
7.51
7.36
7.35
7.33
7.15
7.14
7.12
2.38
2.04
H2N
N
NH
O
O
H3C
NH
H3C
1.00
0.99
1.00
1.03
2.10
4.24
1.04
2.92
3.01
3.00
f1 (ppm)

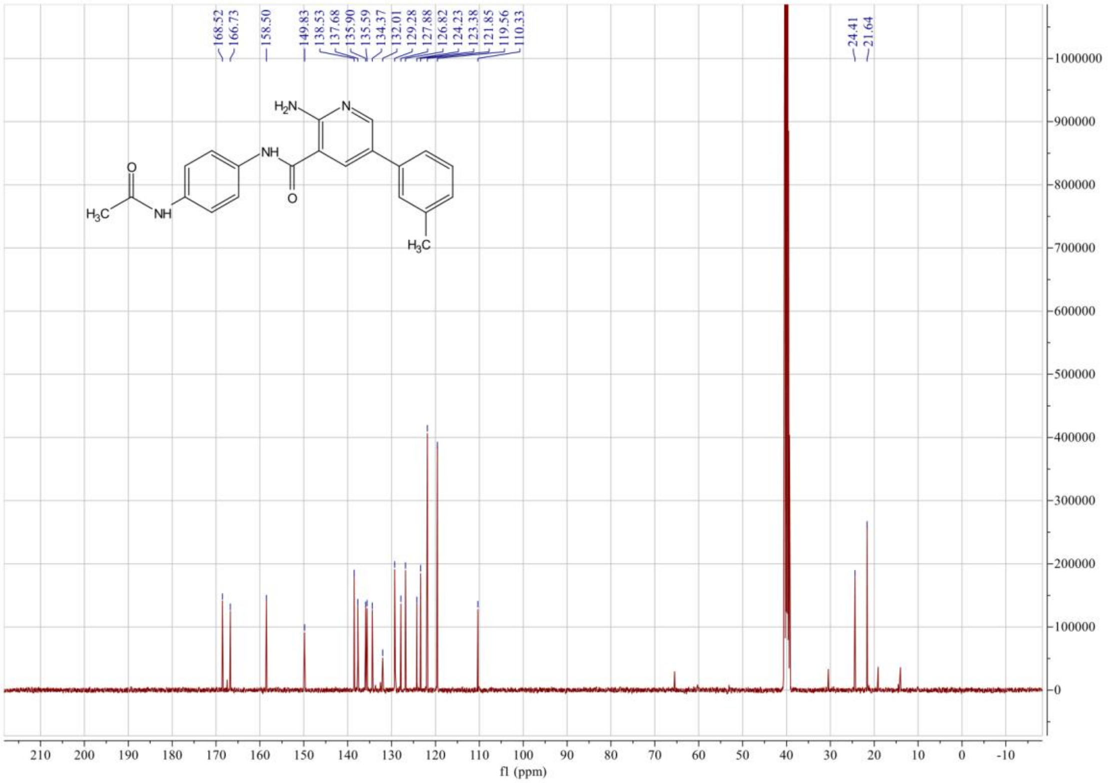
168.52
166.73
158.50
149.83
138.53
137.68
135.90
135.59
134.37
132.01
129.28
127.88
126.82
124.23
123.38
121.85
119.56
110.33
24.41
21.64
H2N
N
NH
O
O
H3C
NH
H3C
f1 (ppm)

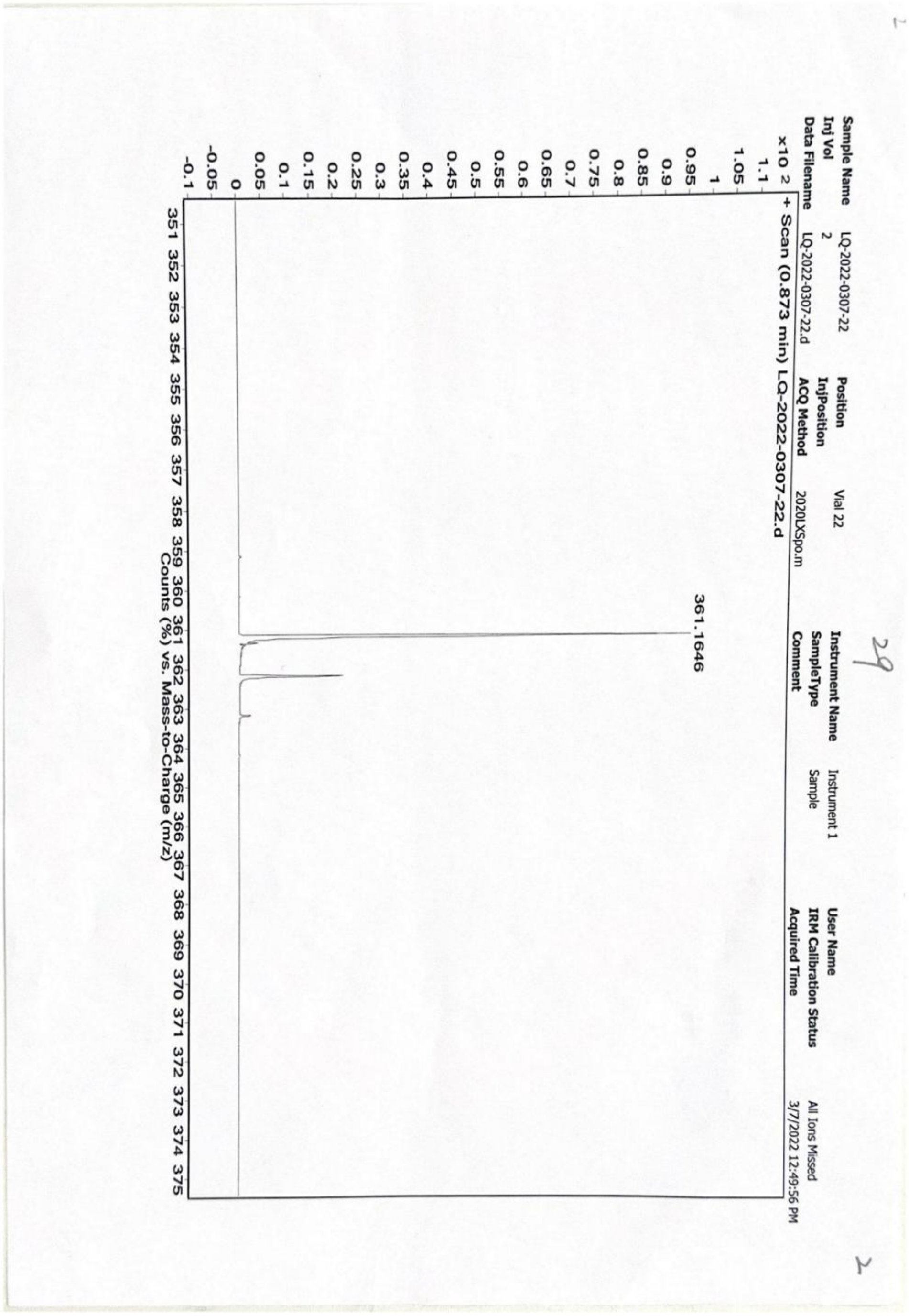

Figure S24. The ^{1}H NMR,^{13}C NMR and ESI-HRMS spectra of compound **29**

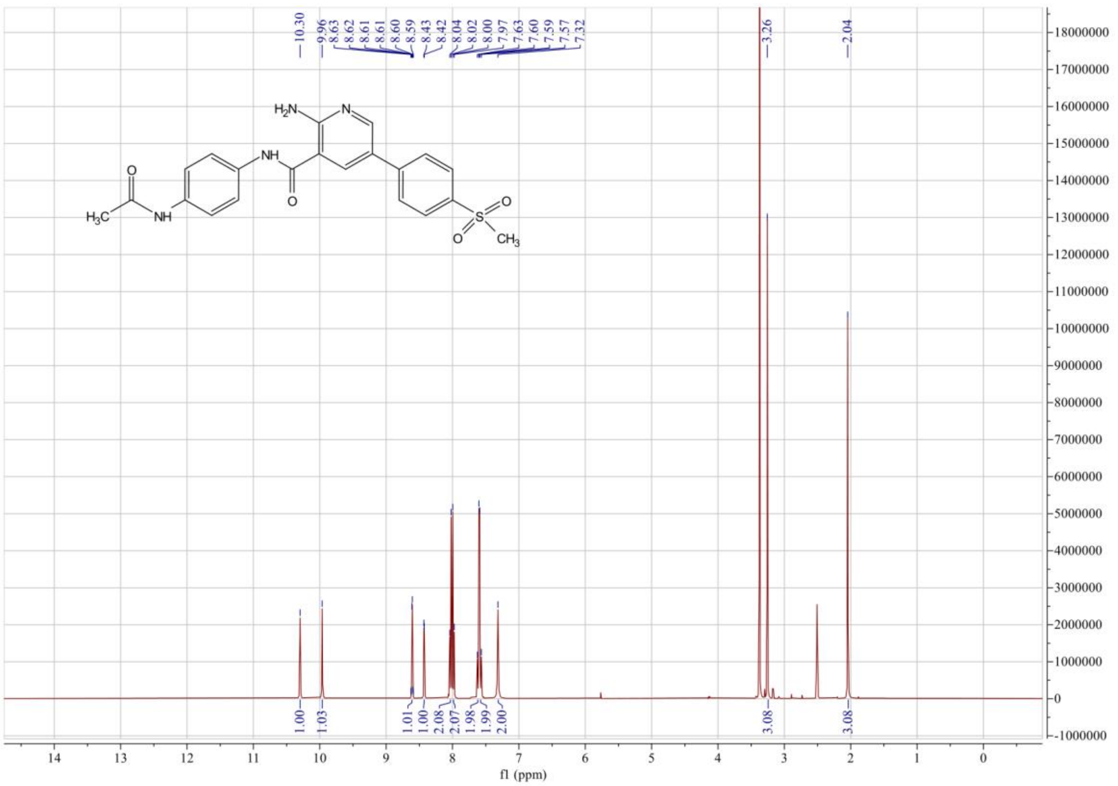

10.30
9.96
8.63
8.62
8.61
8.61
8.60
8.59
8.43
8.42
8.04
8.02
8.00
7.97
7.63
7.60
7.59
7.57
7.32
3.26
2.04
1.00
1.03
1.01
1.00
2.08
2.07
1.98
1.99
2.00
3.08
3.08
f1 (ppm)

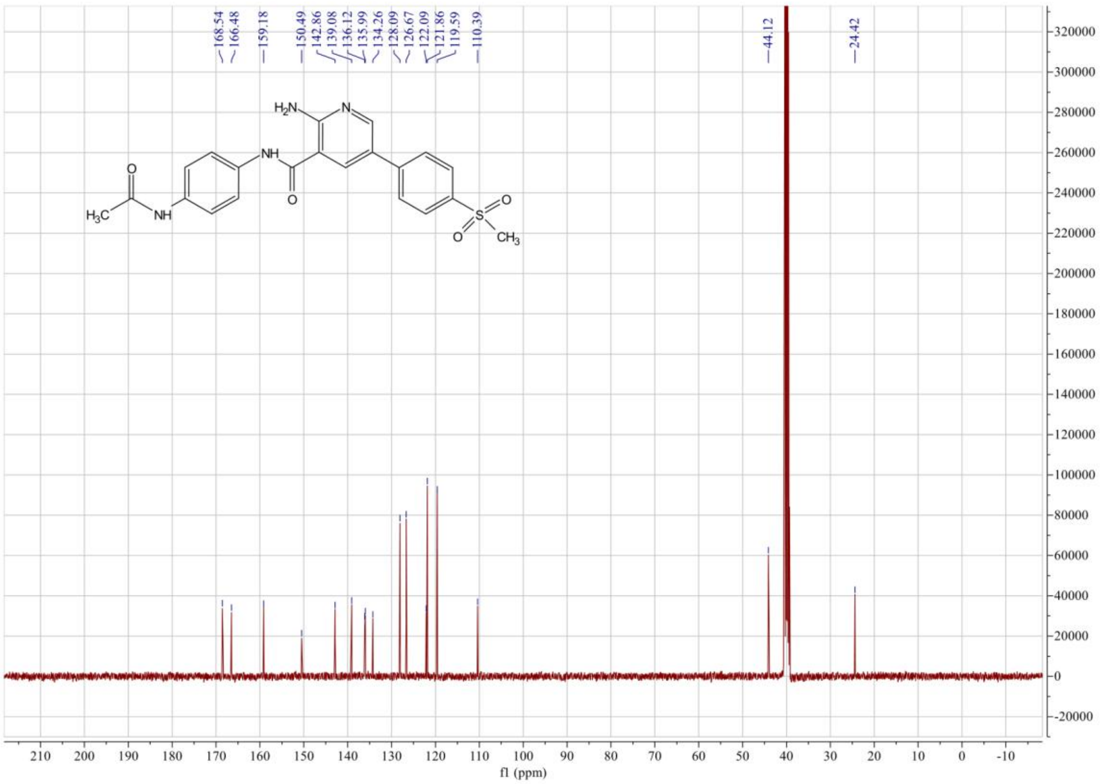

168.54
166.48
159.18
150.49
142.86
139.08
136.12
135.99
134.26
128.09
126.67
122.09
121.86
119.59
110.39
44.12
24.42
f1 (ppm)

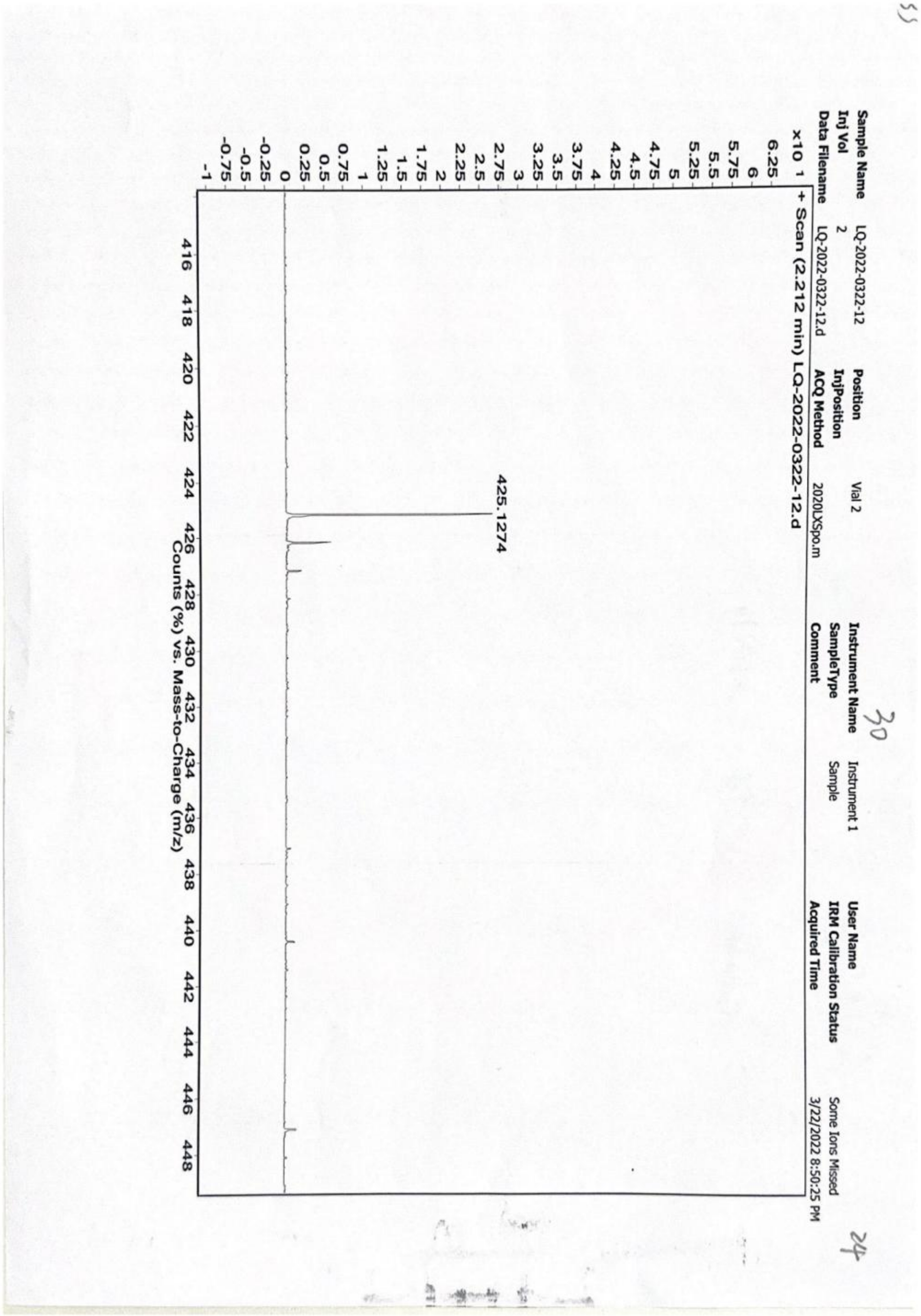

Figure S25. The ^{1}H NMR,^{13}C NMR and ESI-HRMS spectra of compound **30**

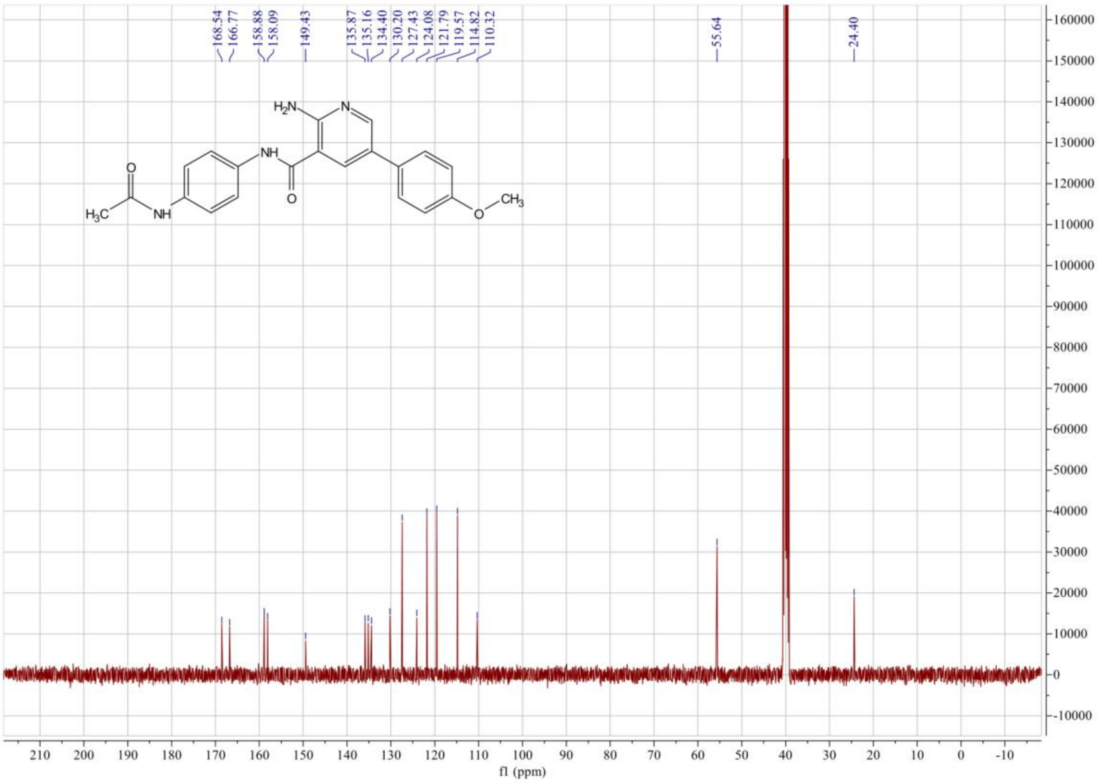
168.54
166.77
158.88
158.09
149.43
135.87
135.16
134.40
130.20
127.43
124.08
121.79
119.57
114.82
110.32
55.64
24.40
fl (ppm)

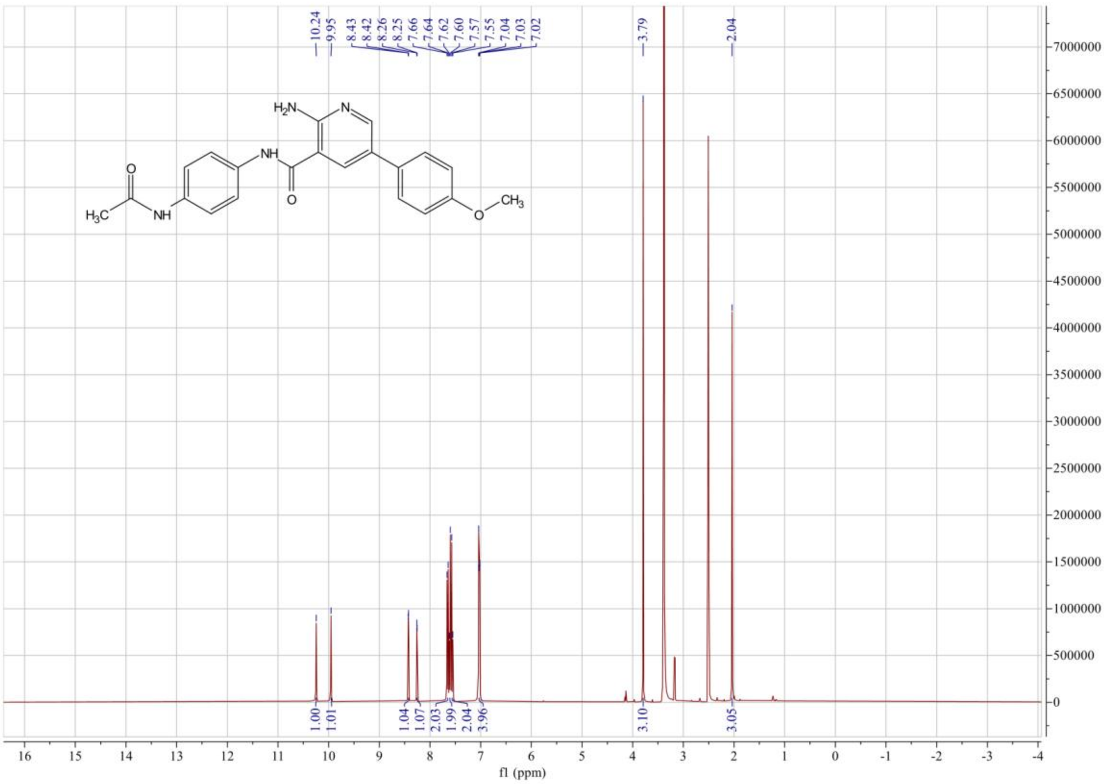
10.24
9.95
8.43
8.42
8.26
8.25
7.66
7.64
7.62
7.60
7.57
7.55
7.04
7.03
7.02
3.79
2.04
1.00
1.01
1.04
1.07
2.03
1.99
2.04
3.96
3.10
3.05
fl (ppm)

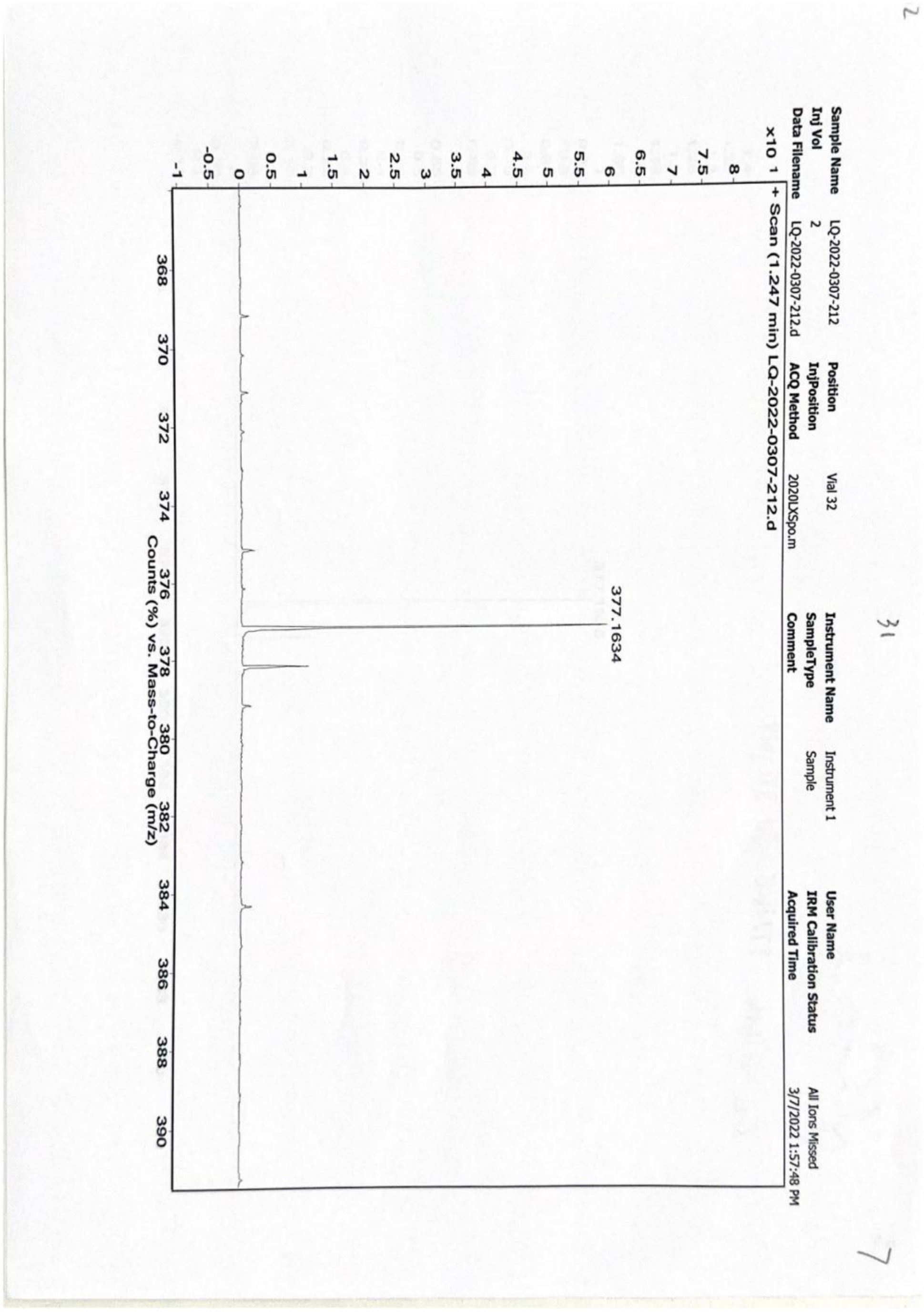

Figure S26. The ^{1}H NMR,^{13}C NMR and ESI-HRMS spectra of compound **31**

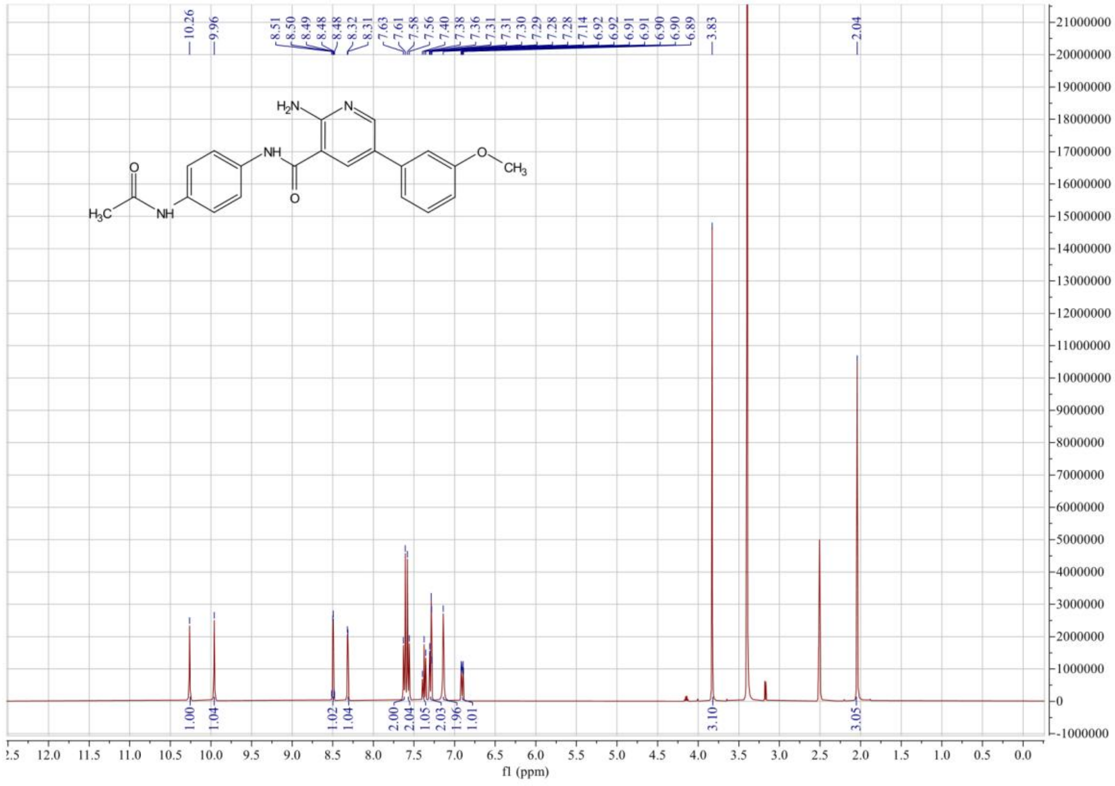
10.26
9.96
8.51
8.50
8.49
8.48
8.48
8.32
8.31
7.63
7.61
7.58
7.56
7.40
7.38
7.36
7.31
7.31
7.30
7.29
7.28
7.28
7.14
6.92
6.92
6.91
6.91
6.90
6.90
6.89
3.83
2.04
H2N
N
NH
O
O
CH3
H3C
NH
1.00
1.04
1.02
1.04
2.00
2.04
1.05
2.03
1.96
1.01
3.10
3.05
f1 (ppm)

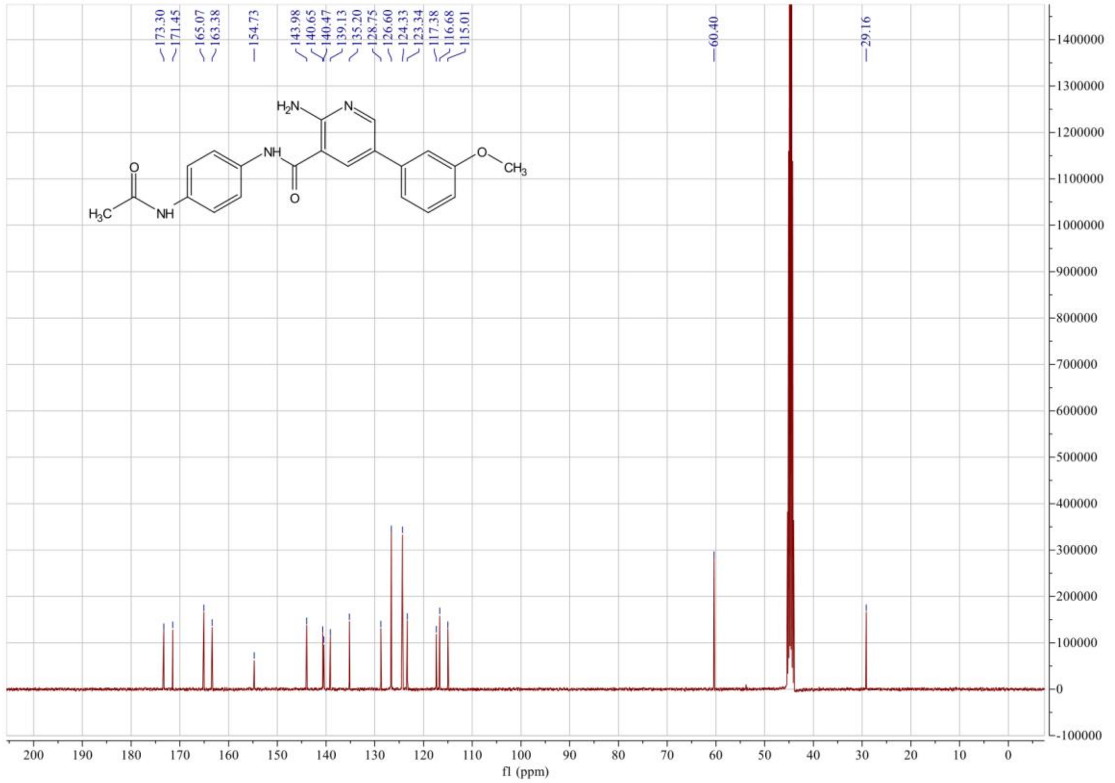
173.30
171.45
165.07
163.38
154.73
143.98
140.65
140.47
139.13
135.20
128.75
126.60
124.33
123.34
117.38
116.68
115.01
60.40
29.16
H2N
N
NH
O
O
CH3
H3C
NH
f1 (ppm)

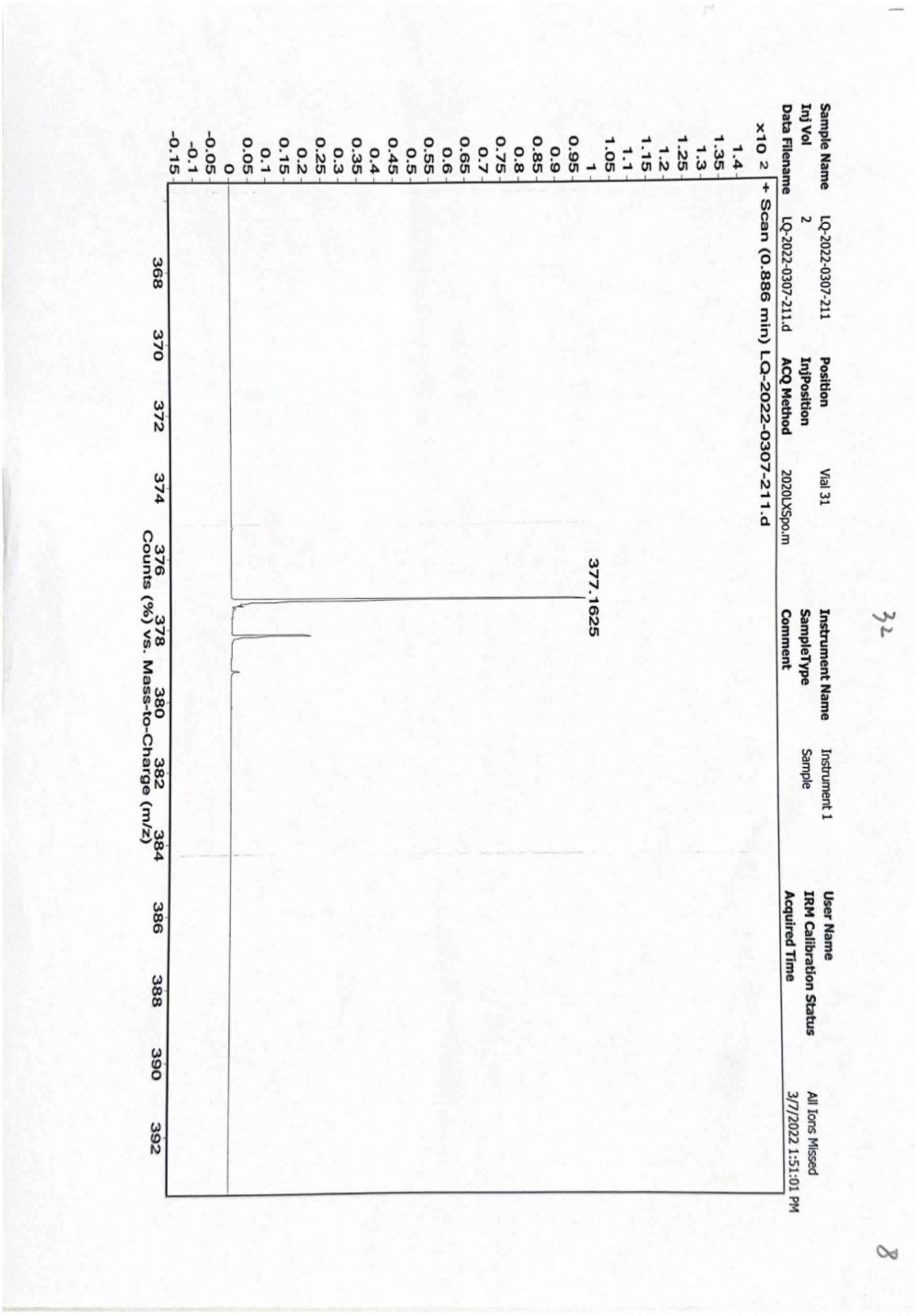

Figure S27. The ^{1}H NMR,^{13}C NMR and ESI-HRMS spectra of compound **32**

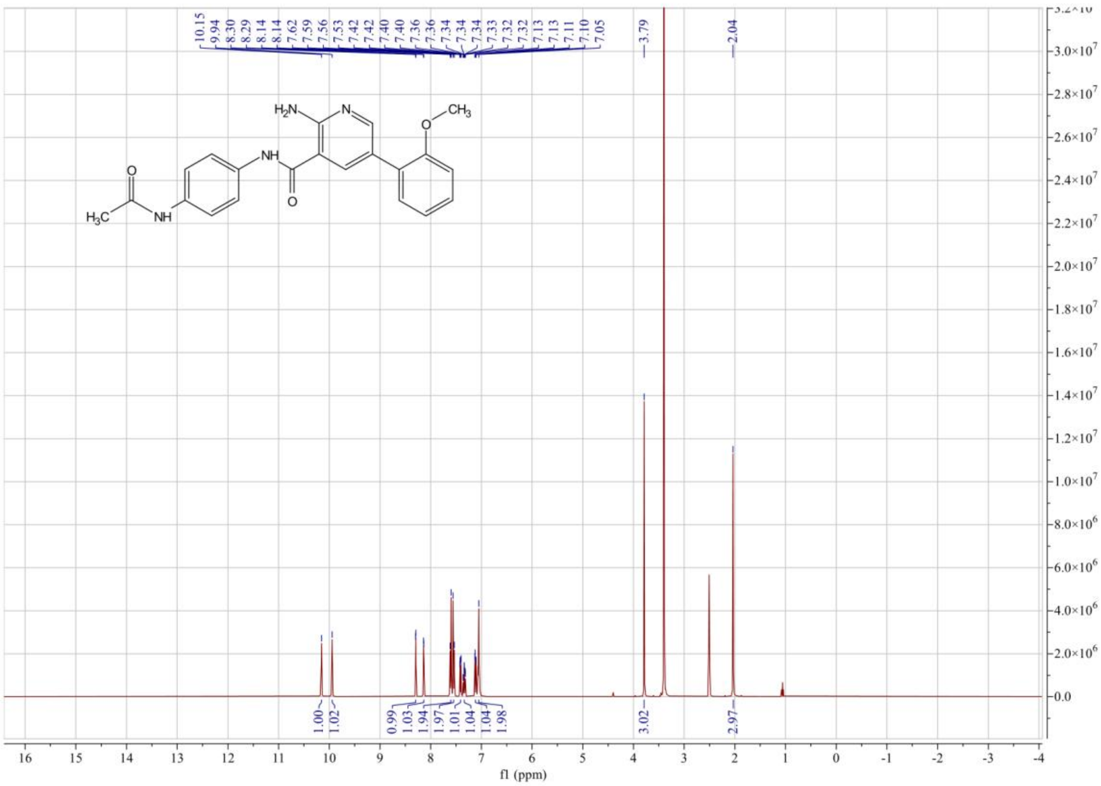

10.15 9.94 8.30 8.29 8.14 8.14 7.62 7.59 7.56 7.53 7.42 7.42 7.40 7.40 7.36 7.36 7.34 7.34 7.34 7.33 7.32 7.32 7.13 7.13 7.11 7.10 7.05
3.79
2.04
1.00 1.02 0.99 1.03 1.94 1.97 1.01 1.04 1.04 1.98 3.02 2.97
fl (ppm)

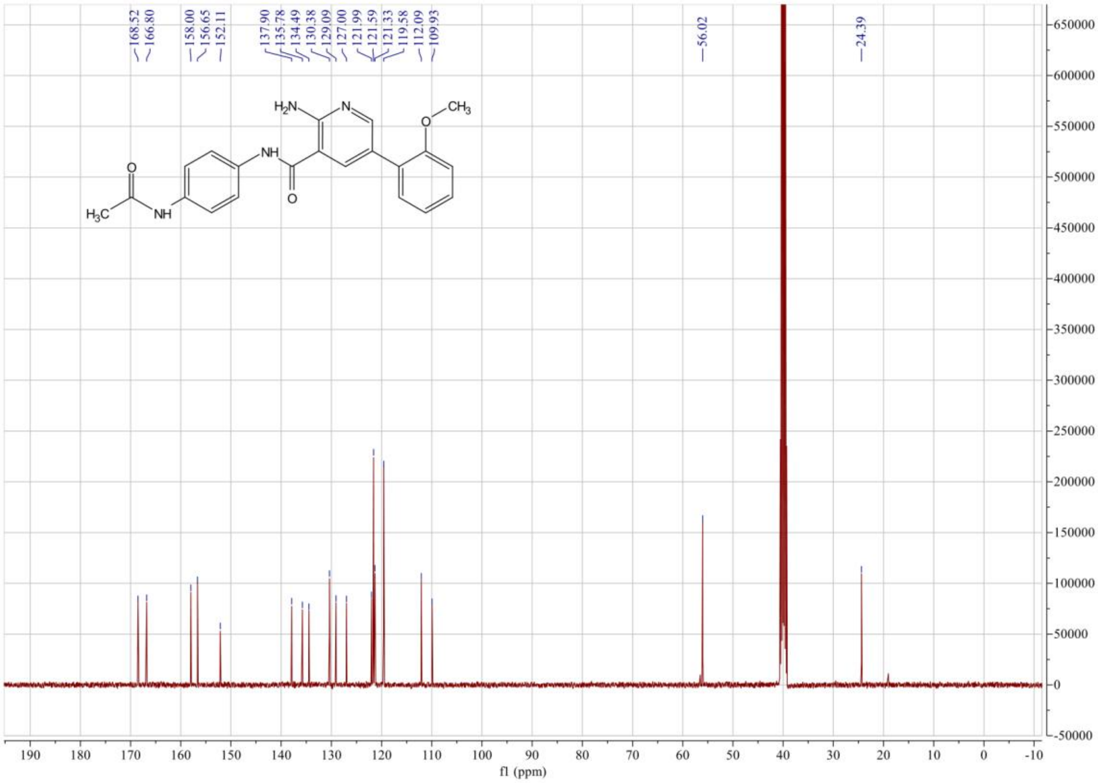

168.52 166.80 158.00 156.65 152.11 137.90 135.78 134.49 130.38 129.09 127.00 121.99 121.59 121.33 119.58 112.09 109.93
56.02
24.39
fl (ppm)

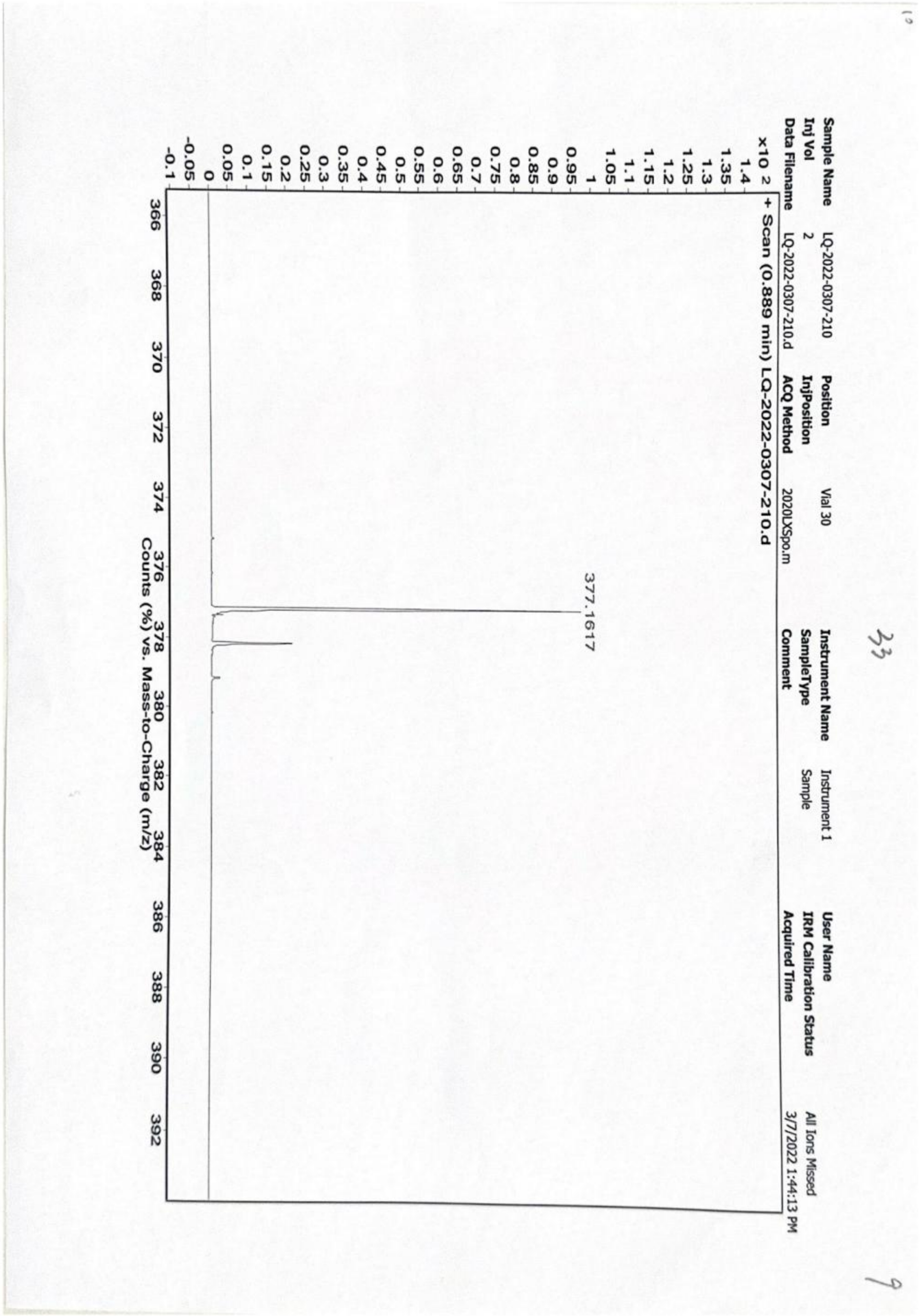

Figure S28. The ^{1}H NMR,^{13}C NMR and ESI-HRMS spectra of compound **33**

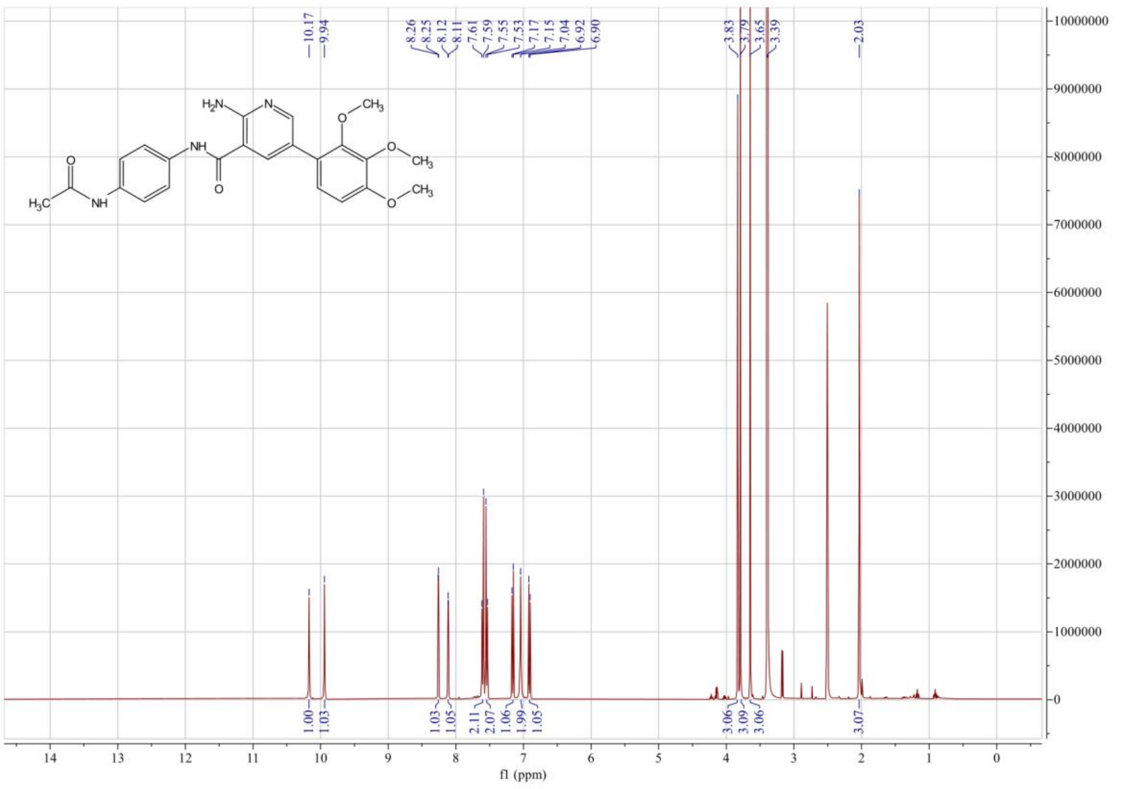

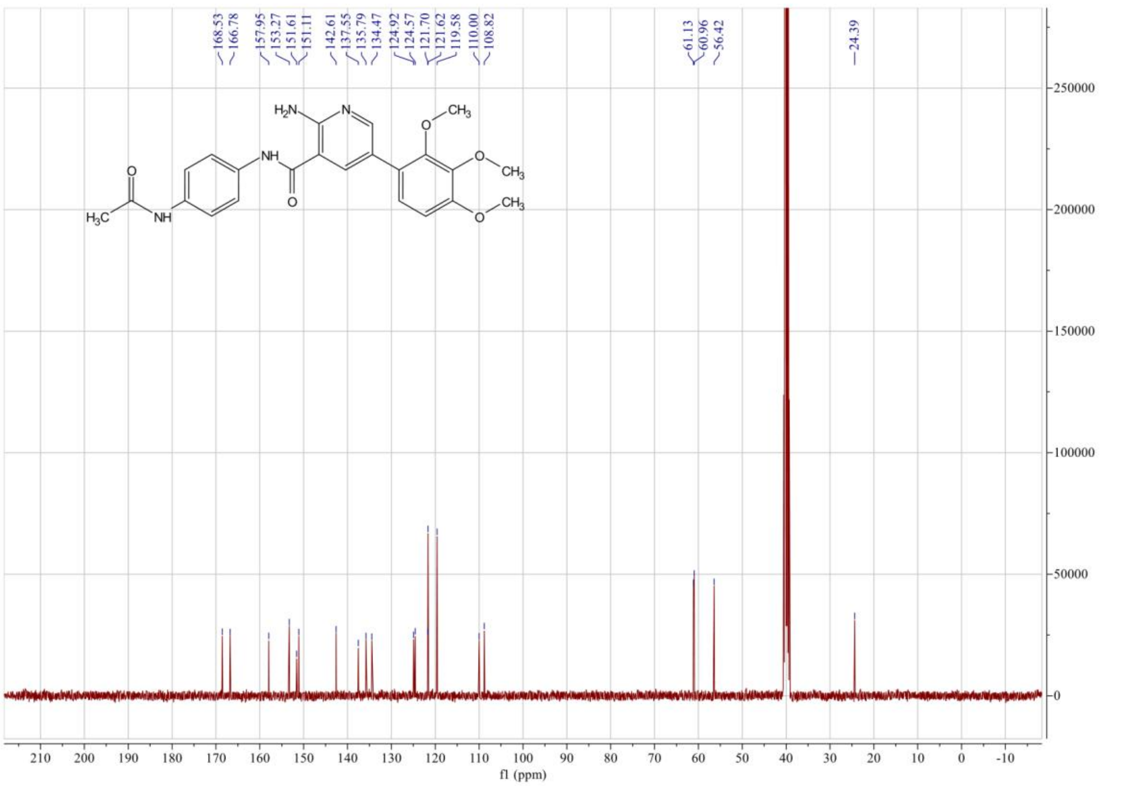

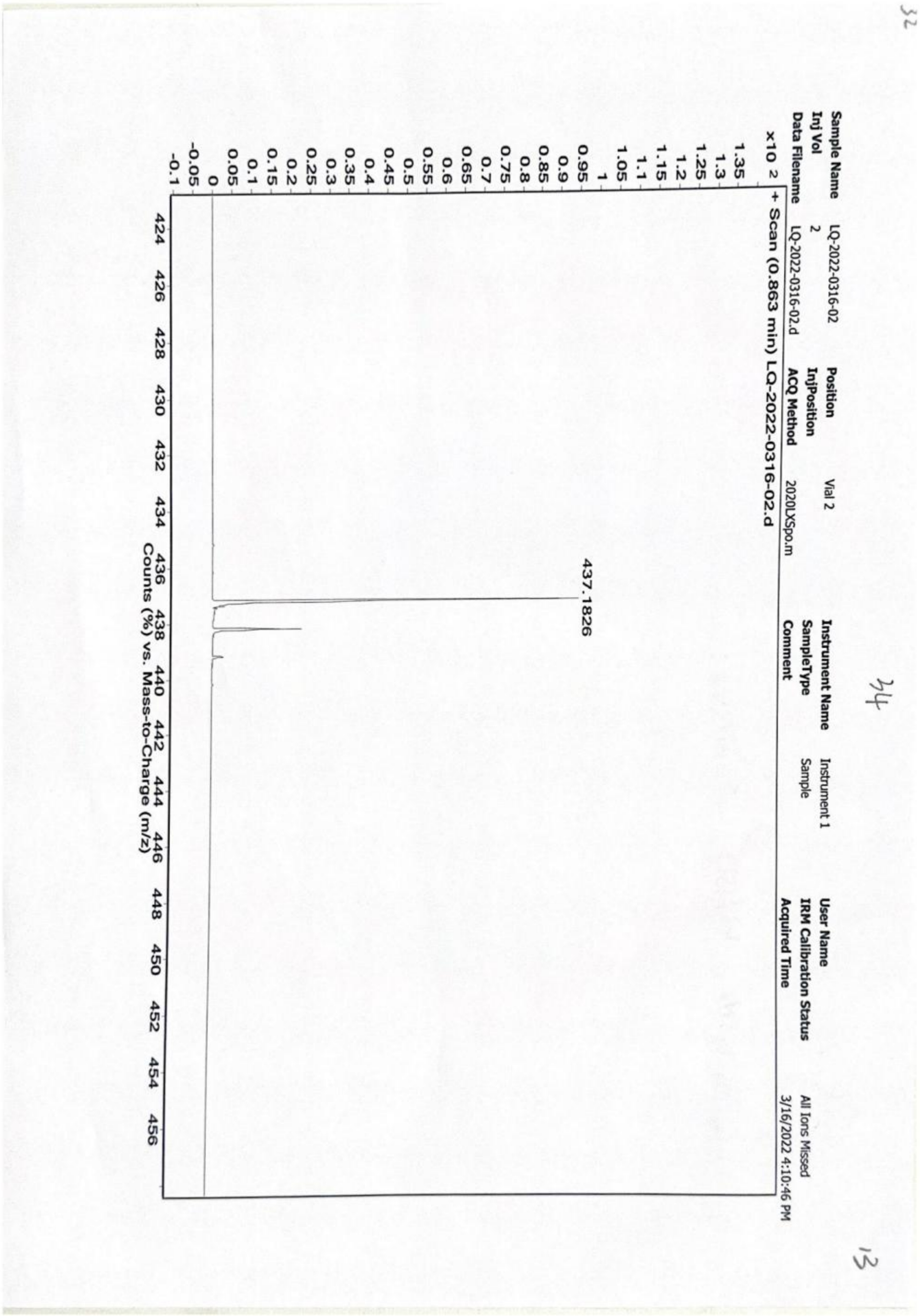

Figure S29. The ^{1}H NMR,^{13}C NMR and ESI-HRMS spectra of compound **34**

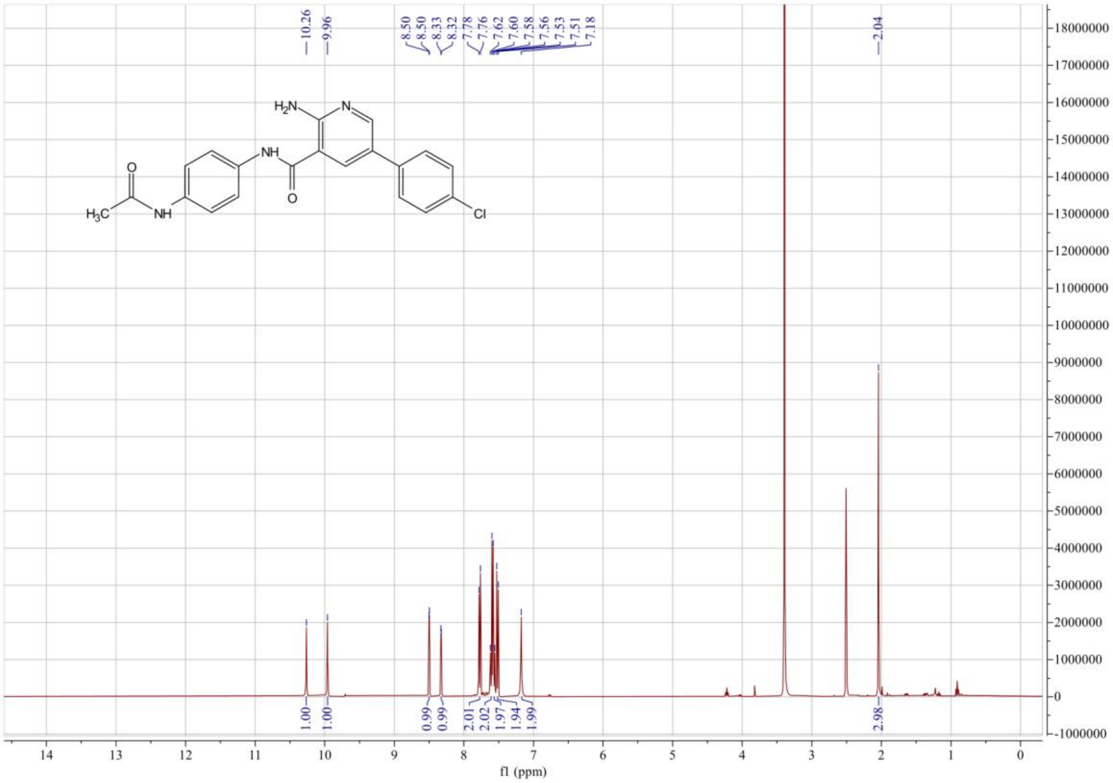
10.26
9.96
8.50
8.50
8.33
8.32
7.78
7.76
7.62
7.60
7.58
7.56
7.53
7.51
7.18
2.04
1.00
1.00
0.99
0.99
2.01
2.02
1.97
1.94
1.99
2.98
f1 (ppm)

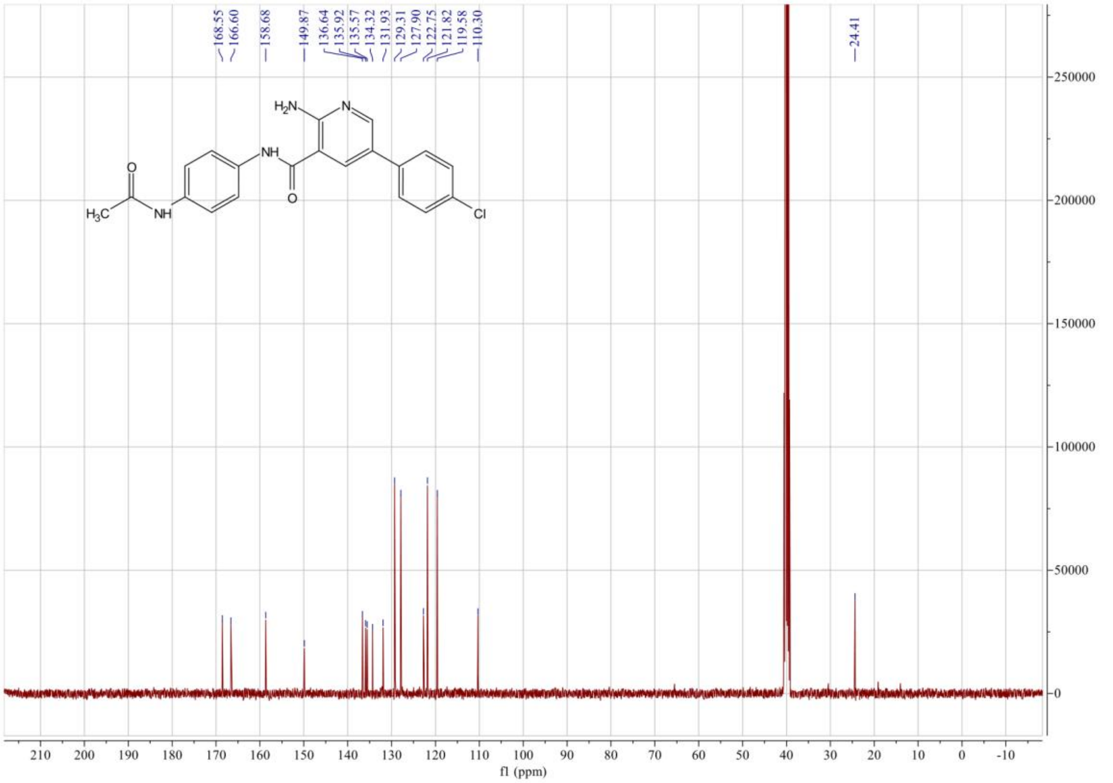
168.55
166.60
158.68
149.87
136.64
135.92
135.57
134.32
131.93
129.31
127.90
122.75
121.82
119.58
110.30
24.41
f1 (ppm)

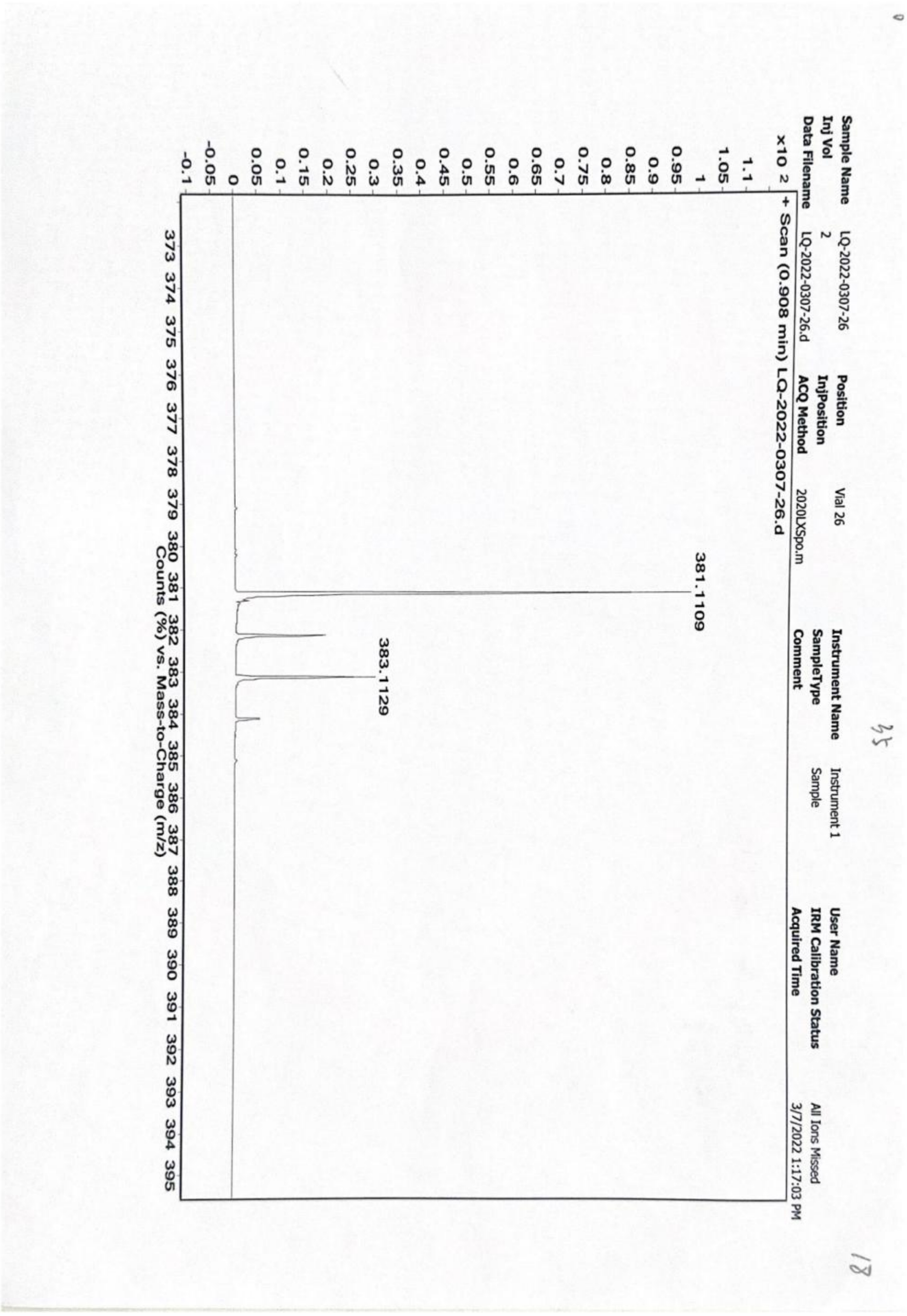

Figure S30. The ^{1}H NMR,^{13}C NMR and ESI-HRMS spectra of compound **35**

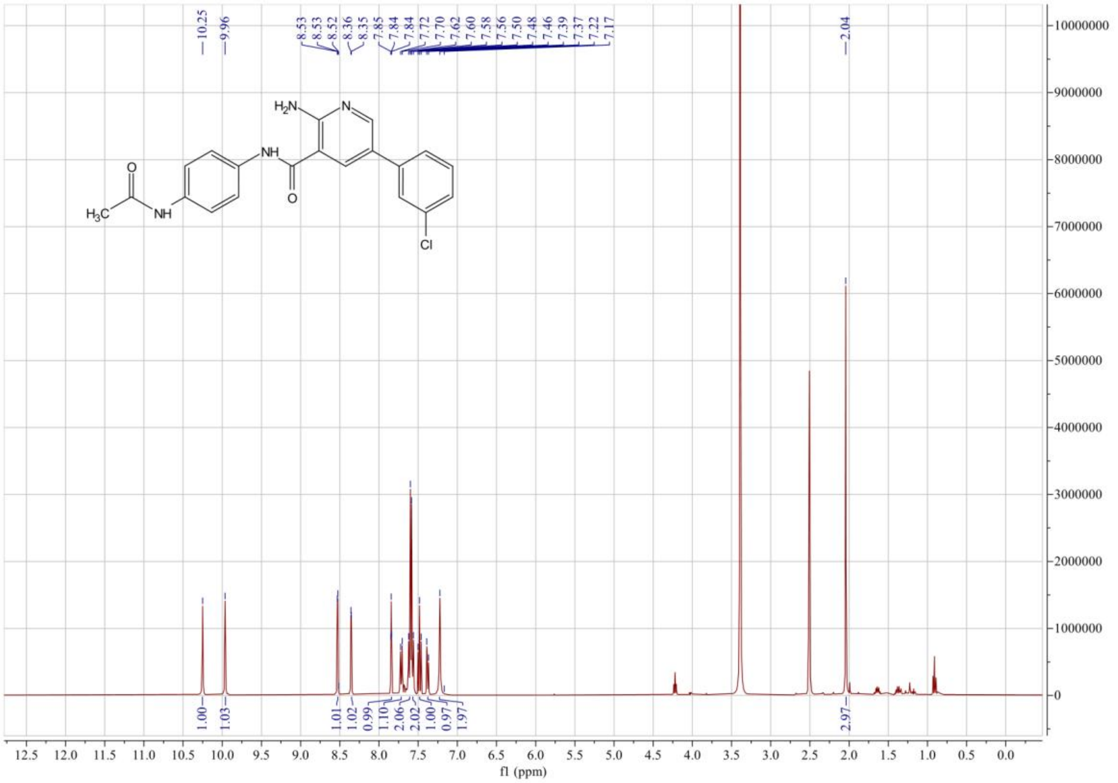

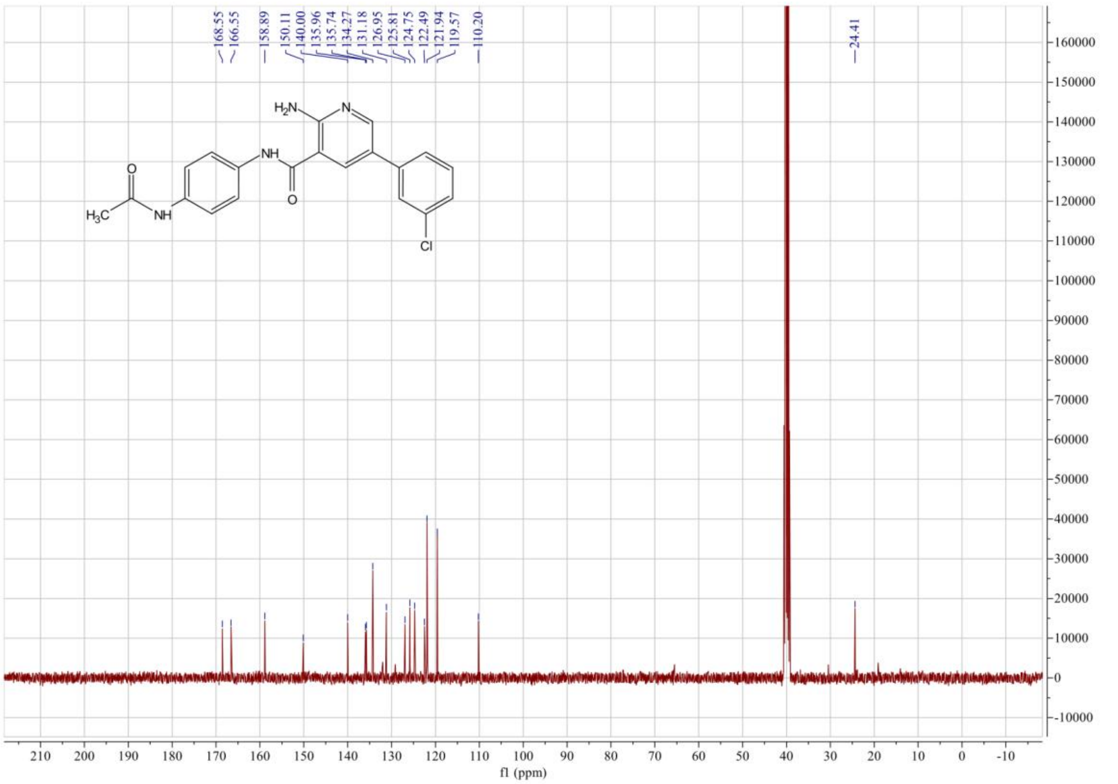

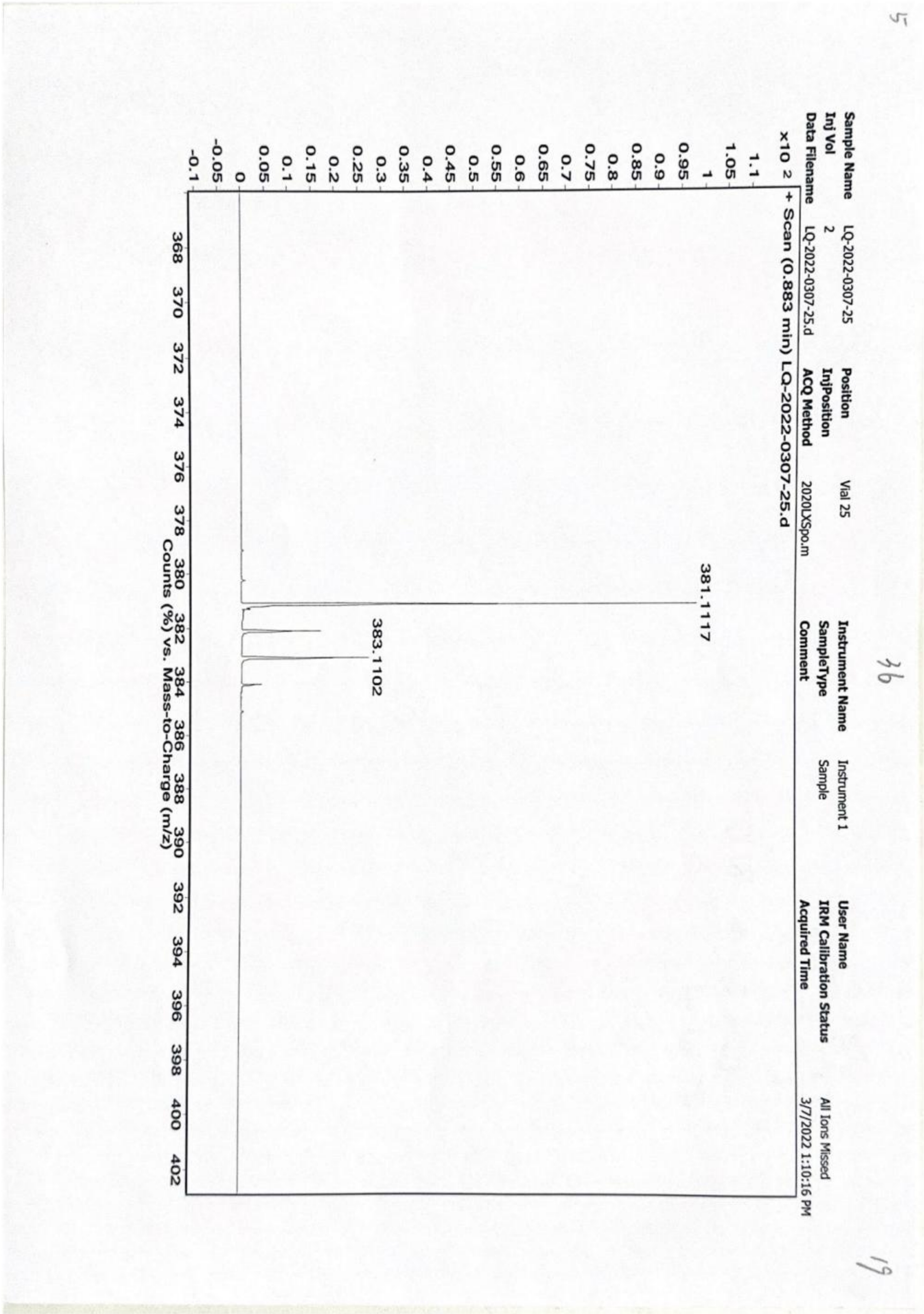

Figure S31. The ^{1}H NMR,^{13}C NMR and ESI-HRMS spectra of compound **36**

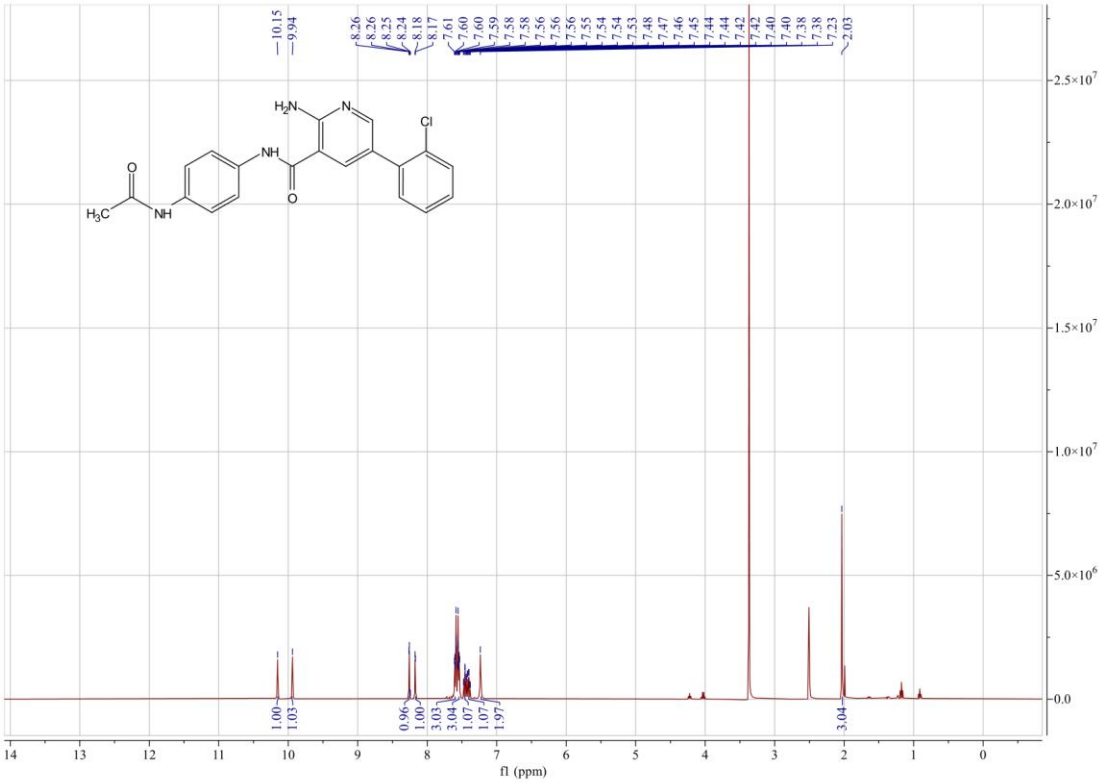
10.15
9.94
8.26
8.26
8.25
8.24
8.18
8.17
7.61
7.60
7.60
7.59
7.58
7.58
7.56
7.56
7.56
7.55
7.54
7.54
7.53
7.48
7.47
7.46
7.45
7.44
7.44
7.42
7.42
7.40
7.38
7.38
7.23
2.03
H2N
N
Cl
NH
O
O
H3C
NH
1.00
1.03
0.96
1.00
3.03
3.04
1.07
1.07
1.97
3.04
2.5×10^7
2.0×10^7
1.5×10^7
1.0×10^7
5.0×10^6
0.0
fl (ppm)

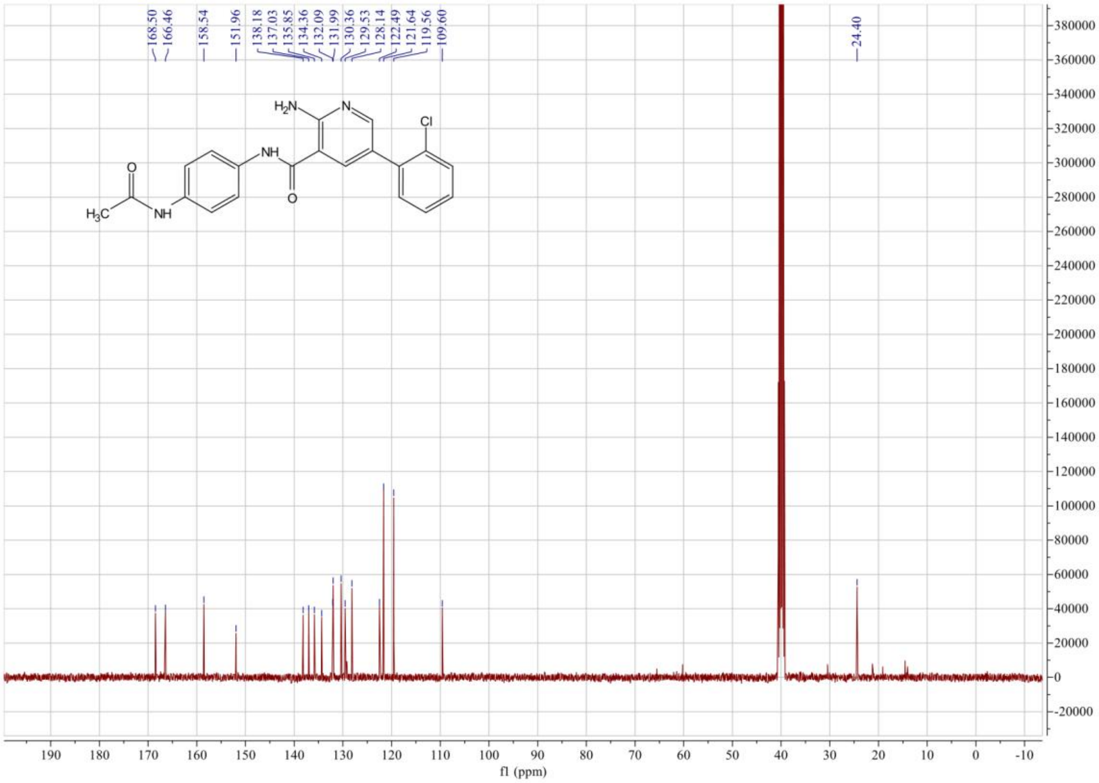
168.50
166.46
158.54
151.96
138.18
137.03
135.85
134.36
132.09
131.99
130.36
129.53
128.14
122.49
121.64
119.56
109.60
24.40
H2N
N
Cl
NH
O
O
H3C
NH
fl (ppm)

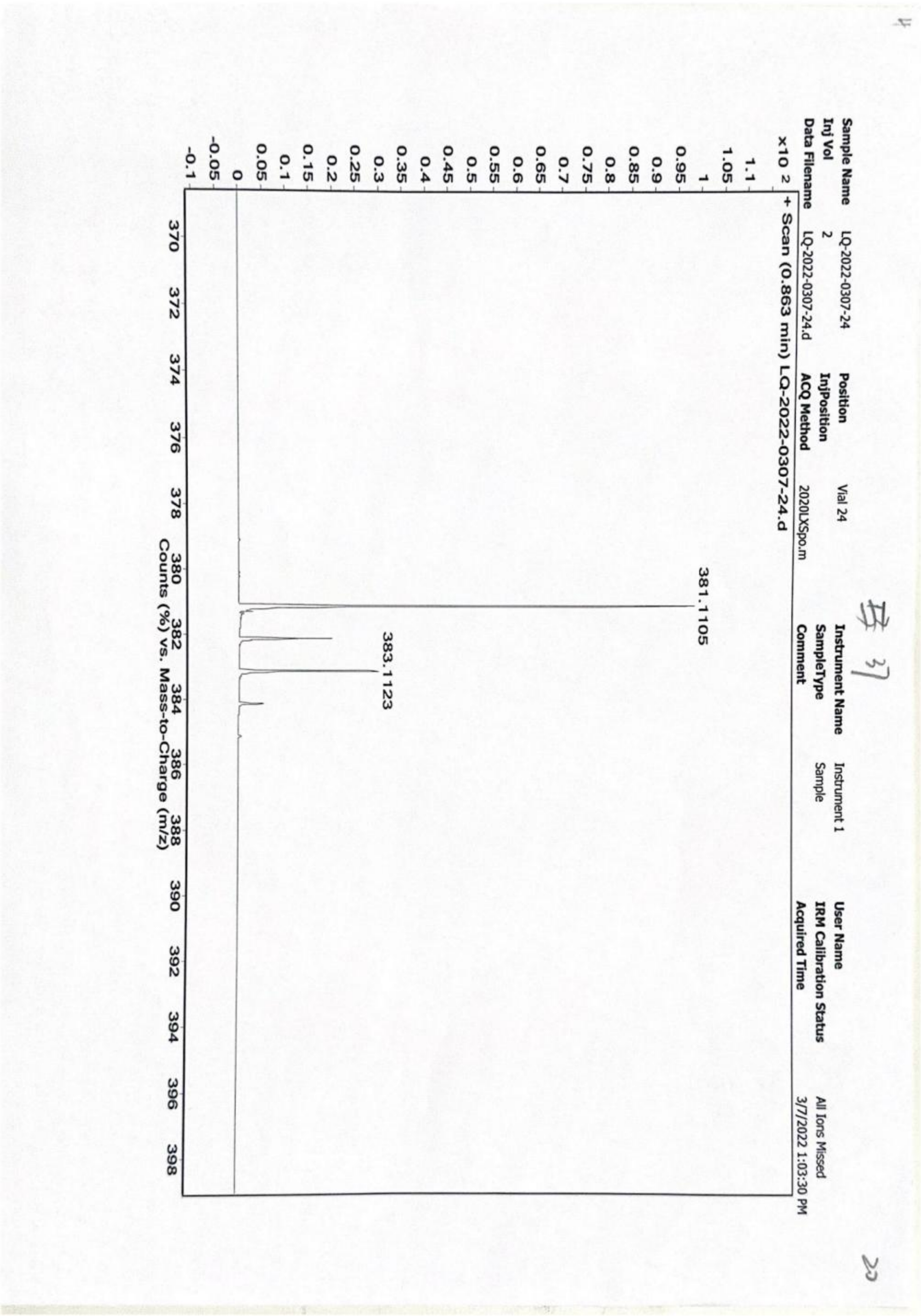

Figure S32. The ^{1}H NMR,^{13}C NMR and ESI-HRMS spectra of compound **37**

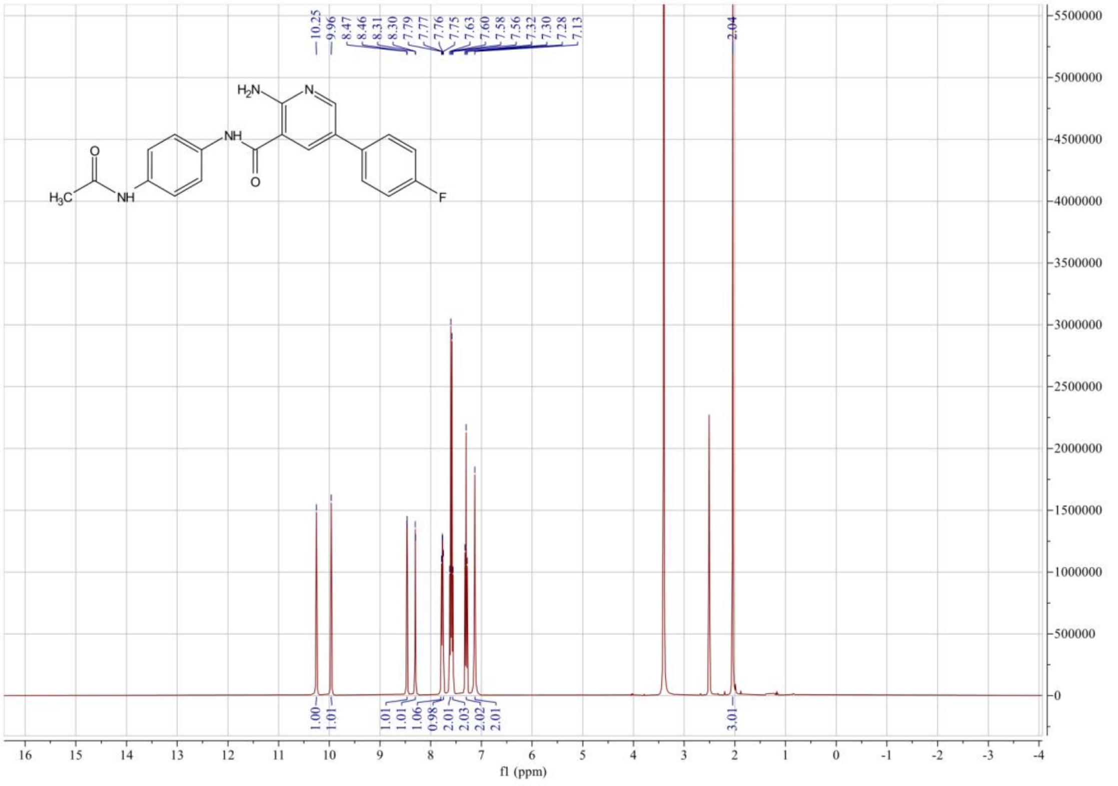
10.25
9.96
8.47
8.46
8.31
8.30
7.79
7.77
7.76
7.75
7.63
7.60
7.58
7.56
7.32
7.30
7.28
7.13
2.04
1.00
1.01
1.01
1.01
1.06
0.98
2.01
2.03
2.02
2.01
3.01
f1 (ppm)

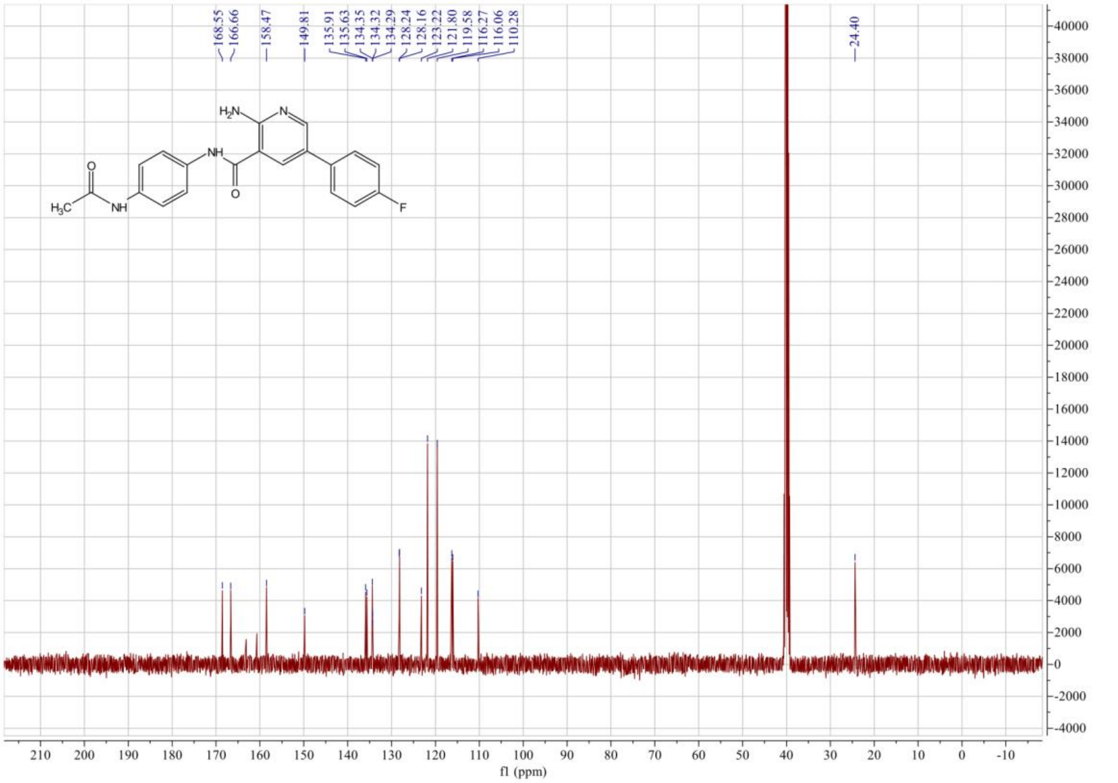
168.55
166.66
158.47
149.81
135.91
135.63
134.35
134.32
134.29
128.24
128.16
123.22
121.80
119.58
116.27
116.06
110.28
24.40
f1 (ppm)

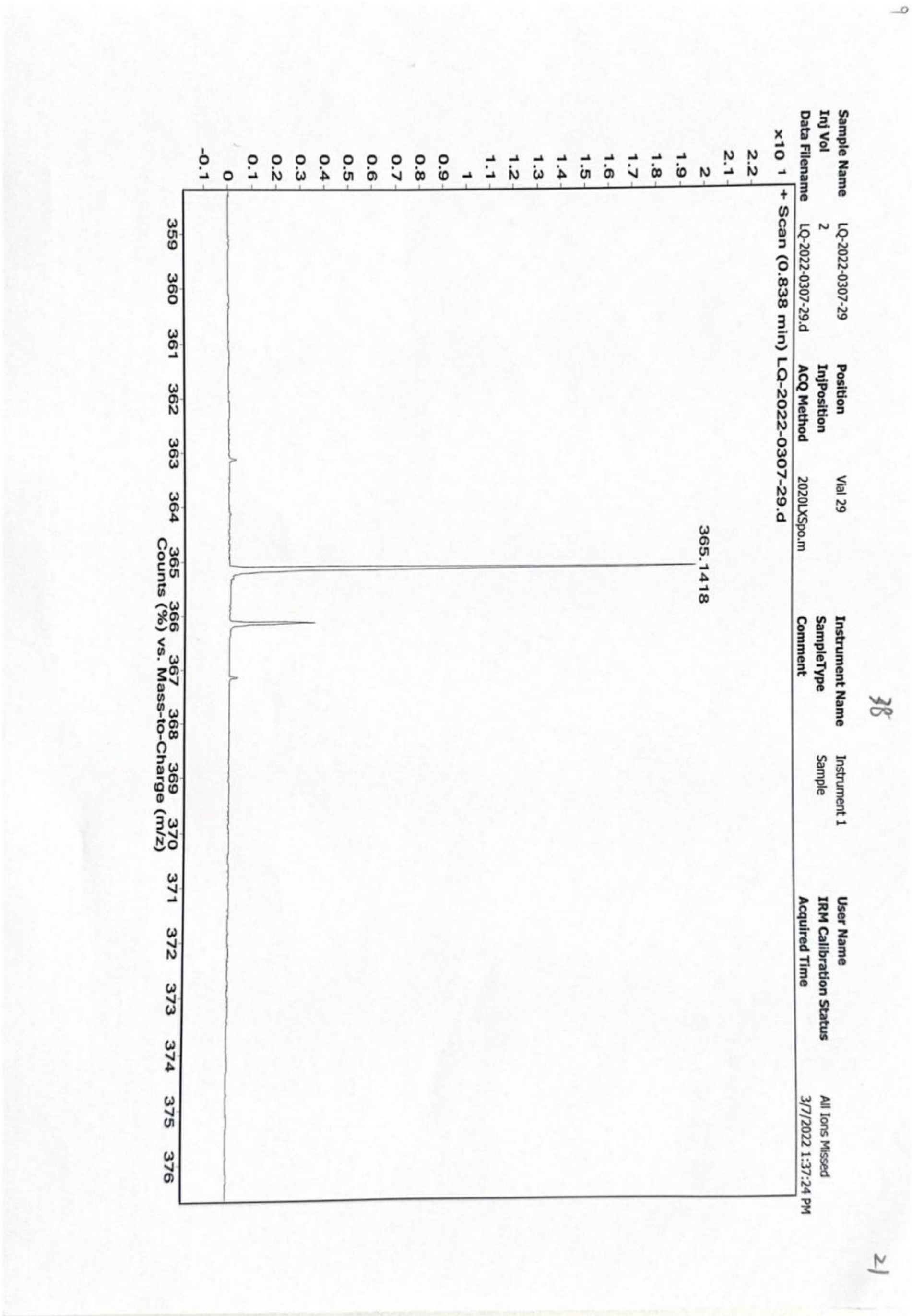

Figure S33. The ^{1}H NMR,^{13}C NMR and ESI-HRMS spectra of compound **38**

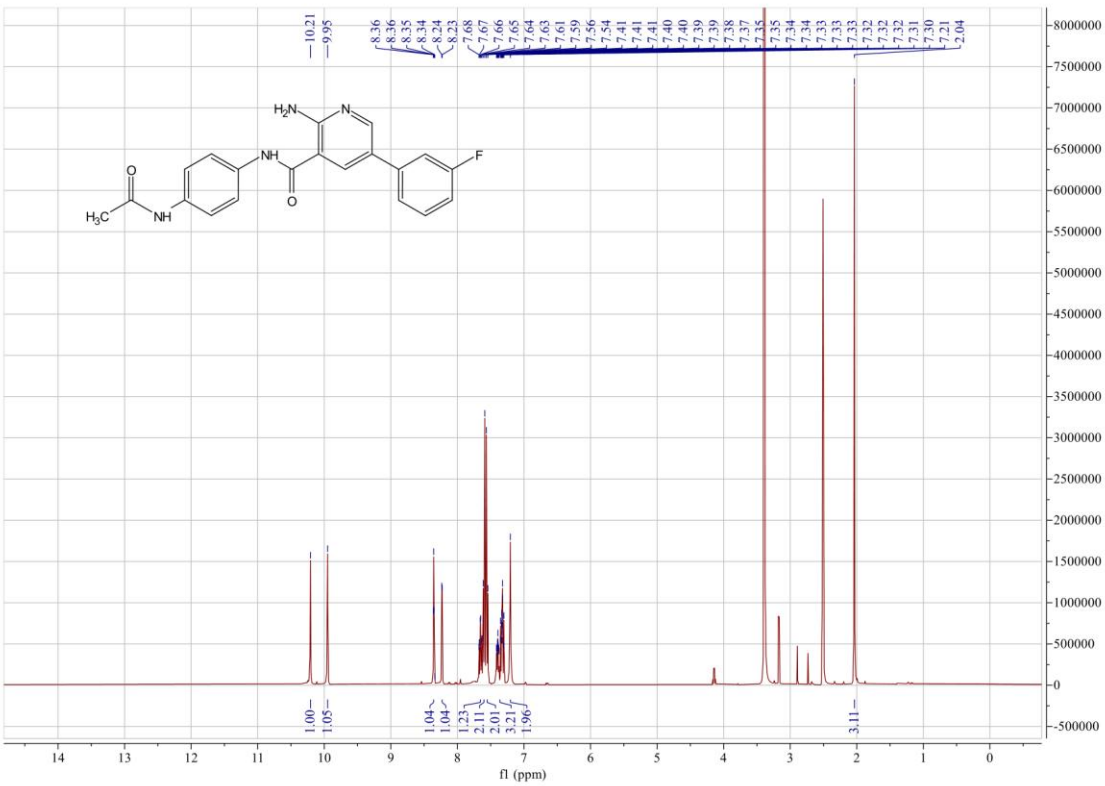
10.21
9.95
8.36 8.36 8.35 8.34 8.24 8.23 7.68 7.67 7.66 7.65 7.64 7.63 7.61 7.59 7.56 7.54 7.41 7.41 7.41 7.40 7.40 7.39 7.39 7.38 7.37 7.35 7.35 7.34 7.34 7.33 7.33 7.33 7.32 7.32 7.32 7.31 7.30 7.21 2.04
1.00 1.05 1.04 1.04 1.23 2.11 2.01 3.21 1.96 3.11
H2N
N
NH
O
F
H3C
NH
f1 (ppm)

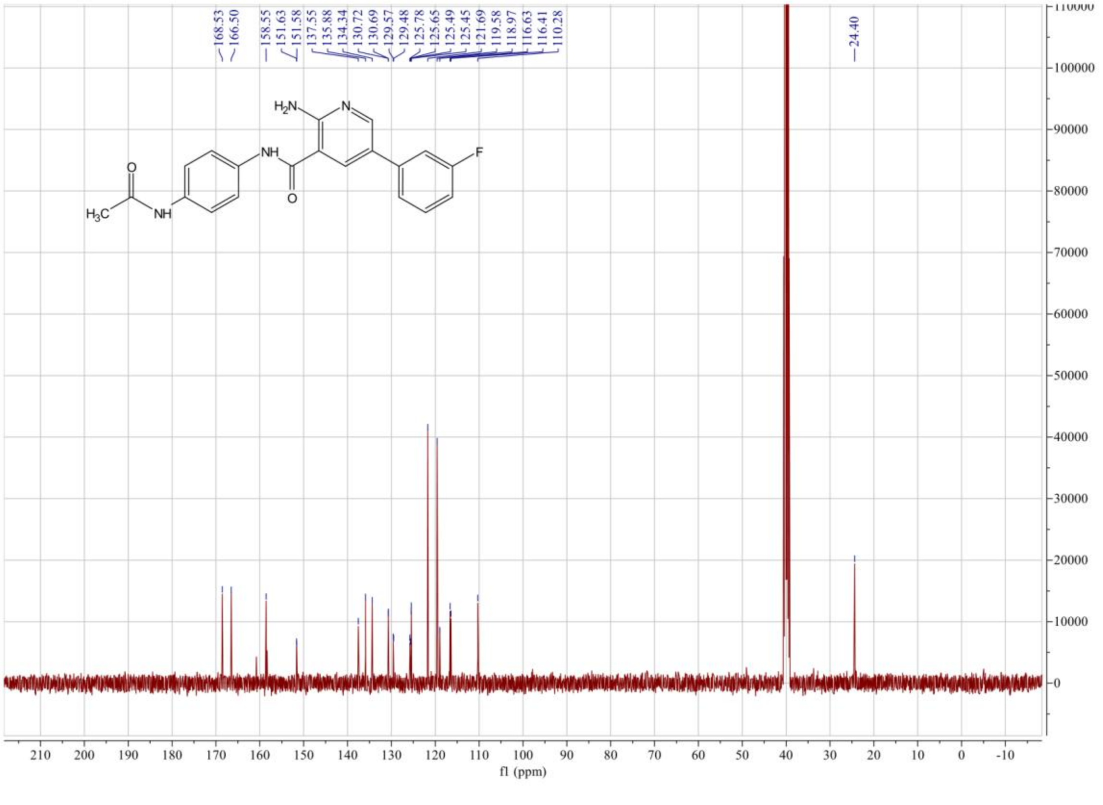
168.53 166.50 158.55 151.63 151.58 137.55 135.88 134.34 130.72 130.69 129.57 129.48 125.78 125.65 125.49 125.45 121.69 119.58 118.97 116.63 116.41 110.28
24.40
H2N
N
NH
O
F
H3C
NH
f1 (ppm)

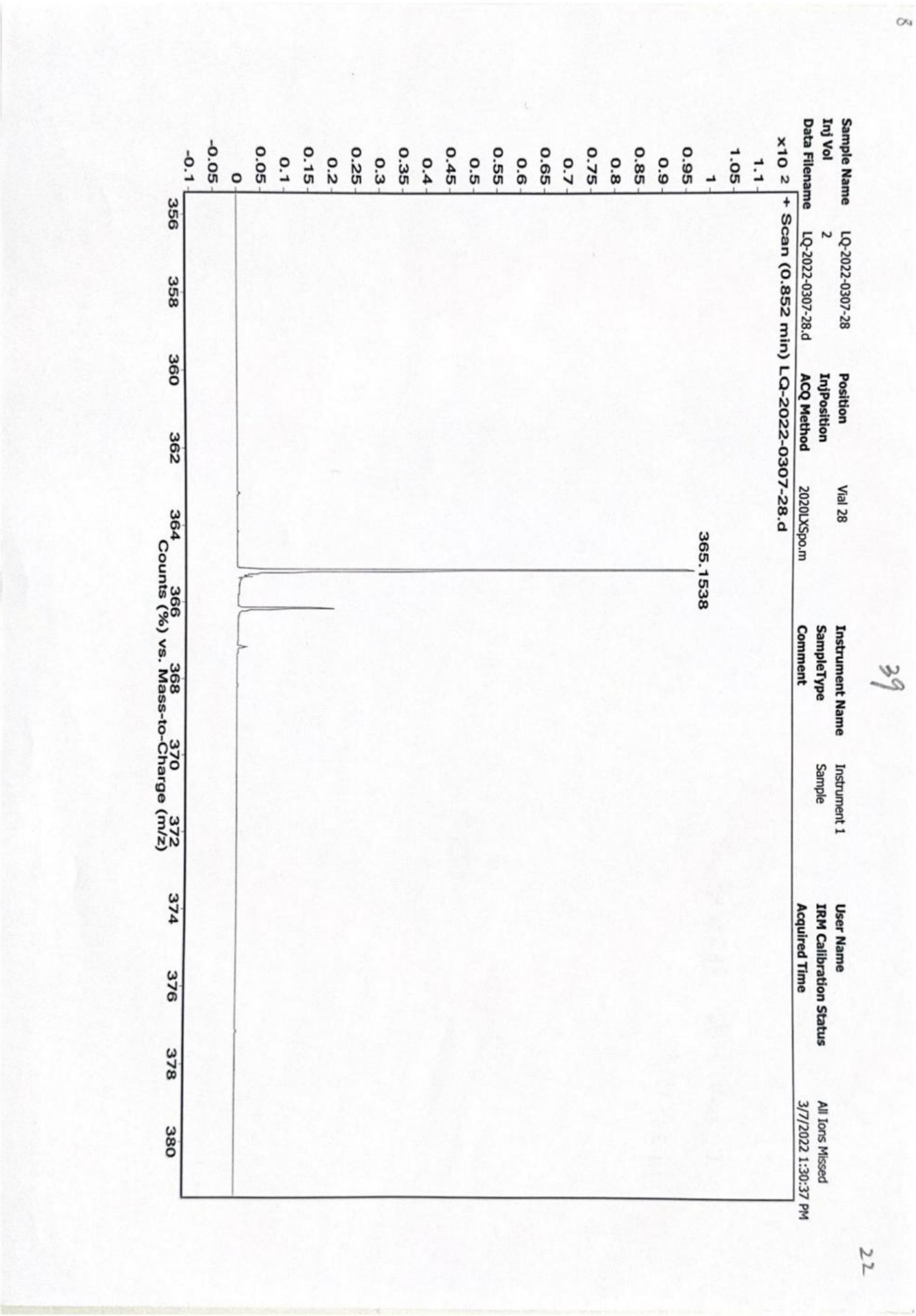

Figure S34. The ^{1}H NMR,^{13}C NMR and ESI-HRMS spectra of compound **39**

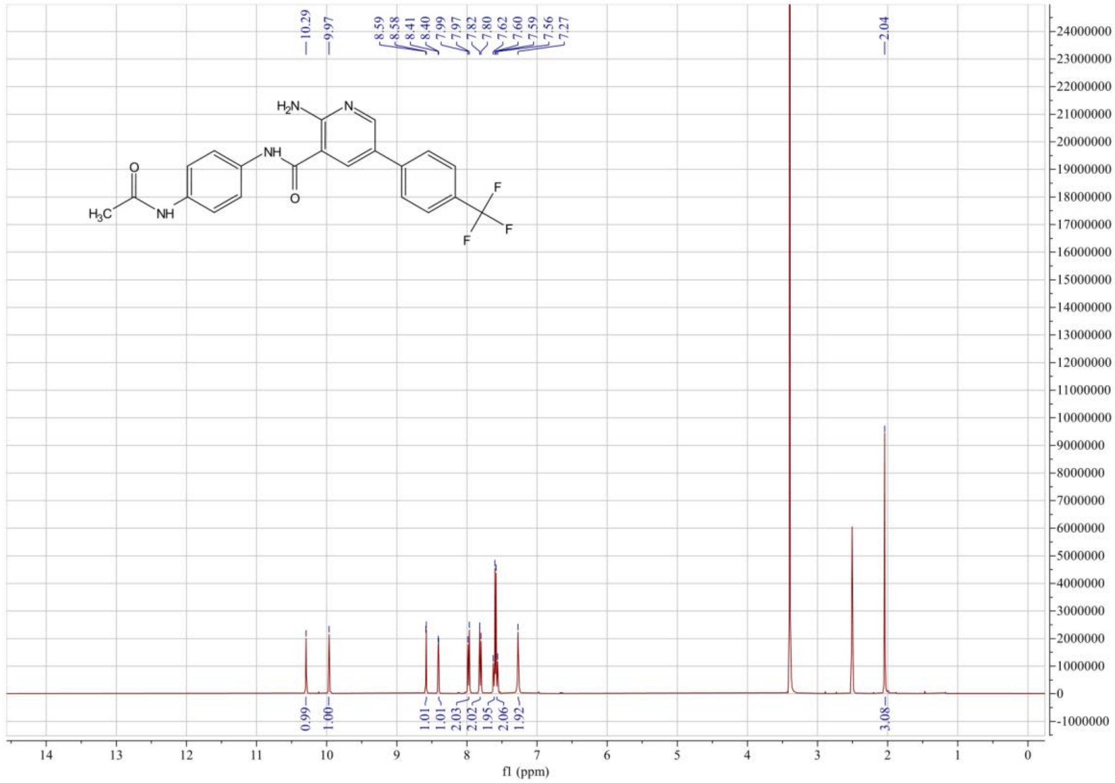

10.29
9.97
8.59
8.58
8.41
8.40
7.99
7.97
7.82
7.80
7.62
7.60
7.59
7.56
7.27
2.04
0.99
1.00
1.01
1.01
2.03
2.02
1.95
2.06
1.92
3.08
f1 (ppm)

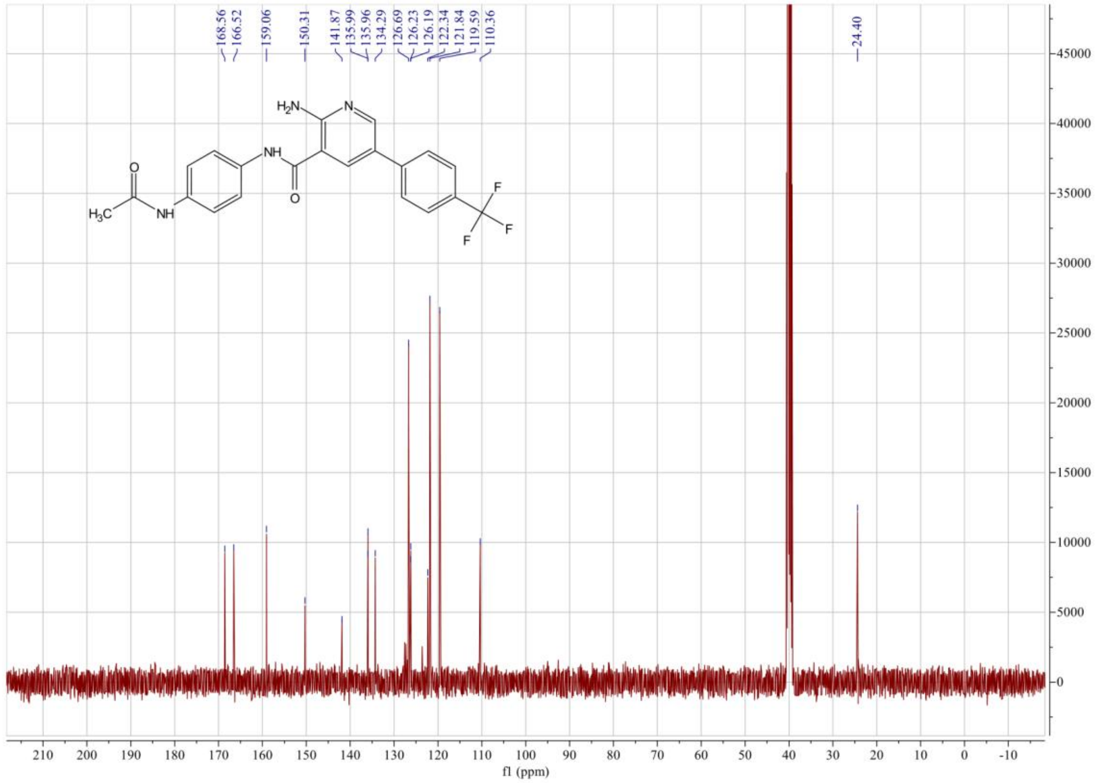

168.56
166.52
159.06
150.31
141.87
135.99
135.96
134.29
126.69
126.23
126.19
122.34
121.84
119.59
110.36
24.40
f1 (ppm)

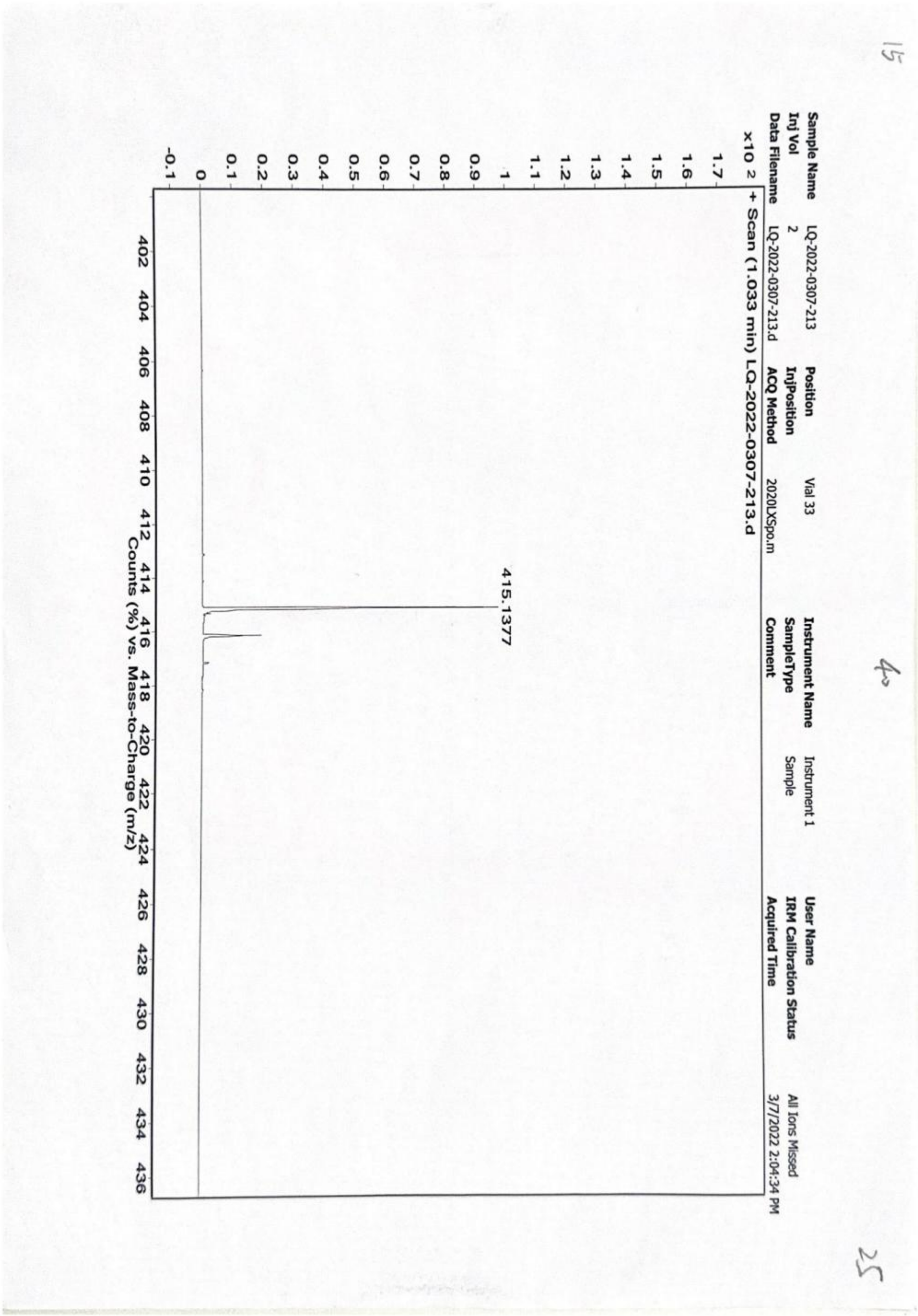

Figure S35. The ^{1}H NMR,^{13}C NMR and ESI-HRMS spectra of compound **40**

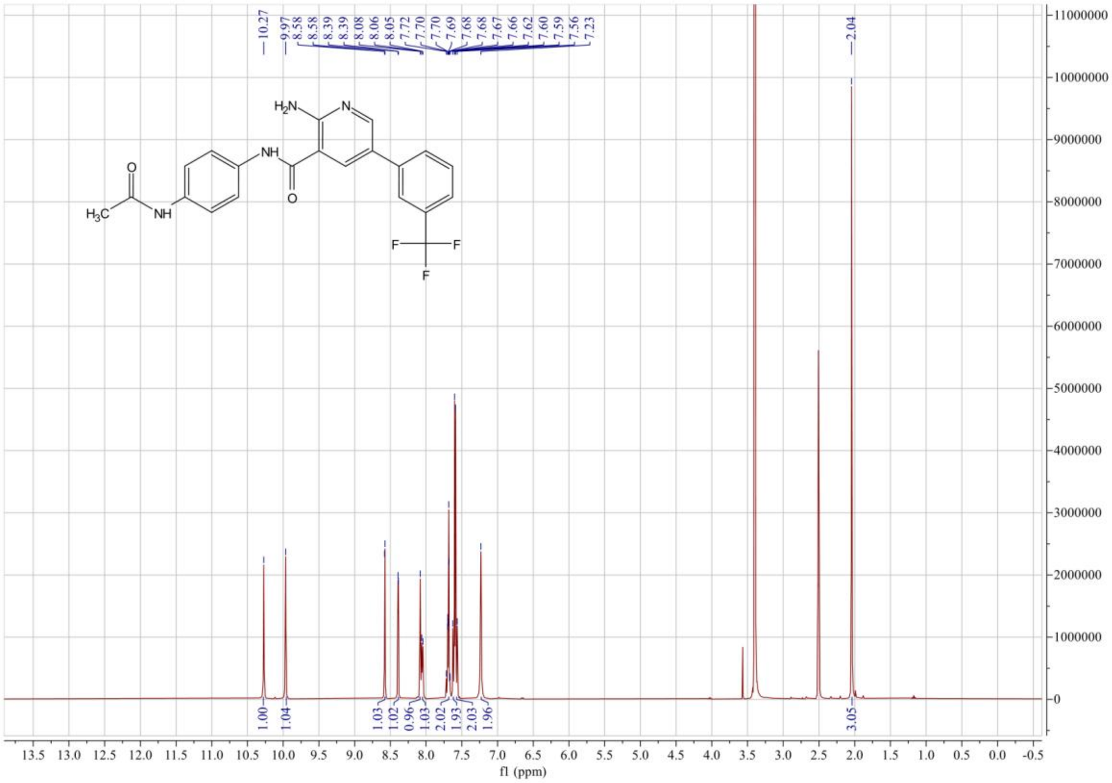
fl (ppm)

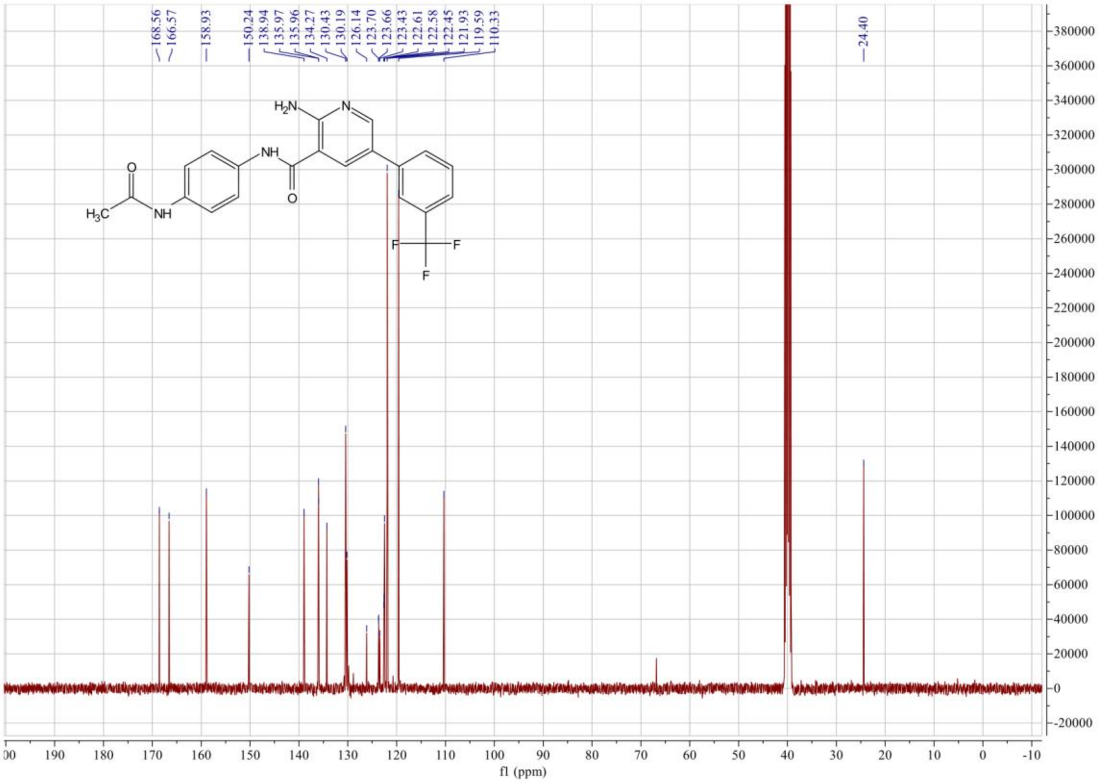
fl (ppm)

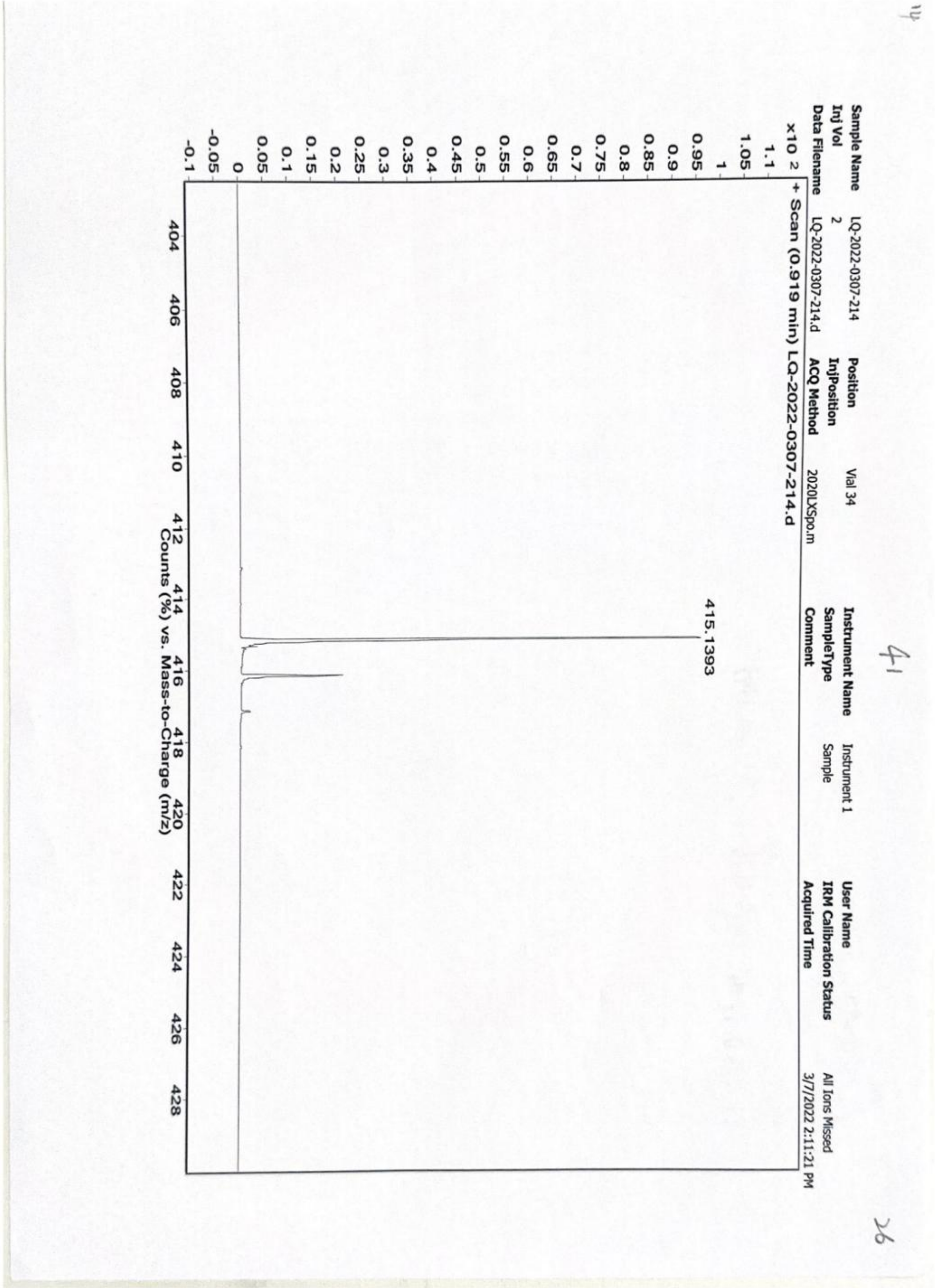

Figure S36. The ^{1}H NMR,^{13}C NMR and ESI-HRMS spectra of compound **41**

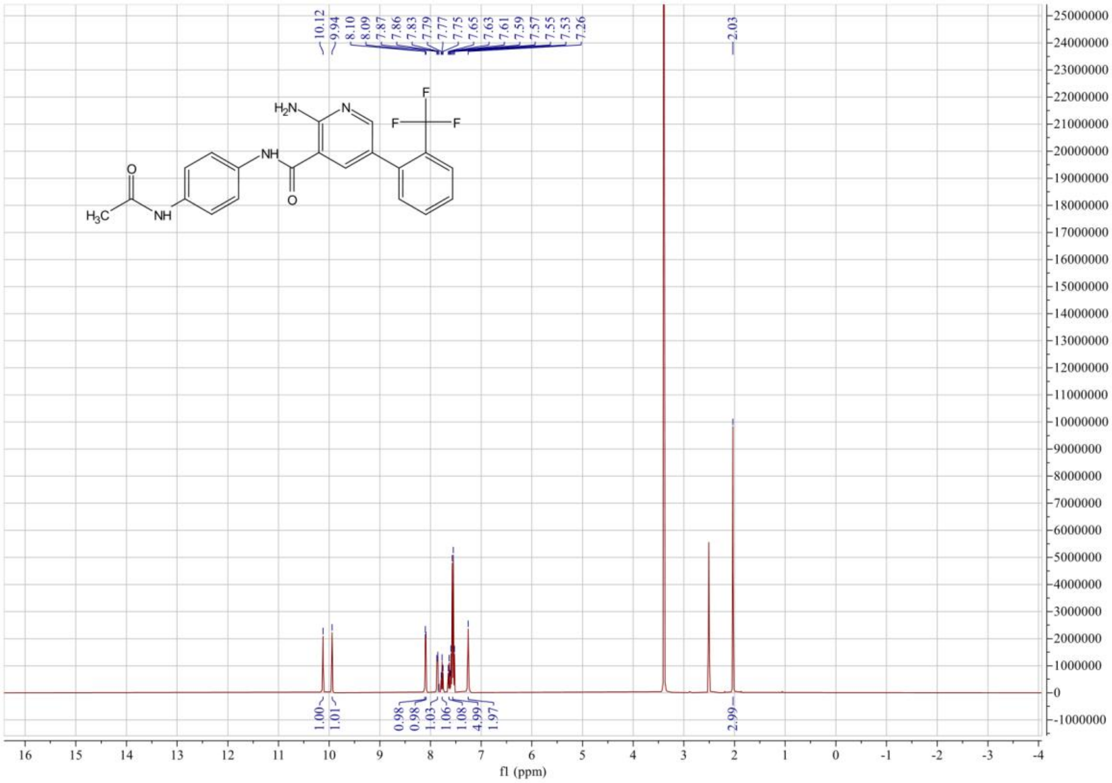
10.12
9.94
8.10
8.09
7.87
7.86
7.83
7.79
7.77
7.75
7.65
7.63
7.61
7.59
7.57
7.55
7.53
7.26
2.03
H2N
N
F
F
F
NH
O
O
H3C
NH
1.00
1.01
0.98
0.98
1.03
1.06
1.08
4.99
1.97
2.99
f1 (ppm)

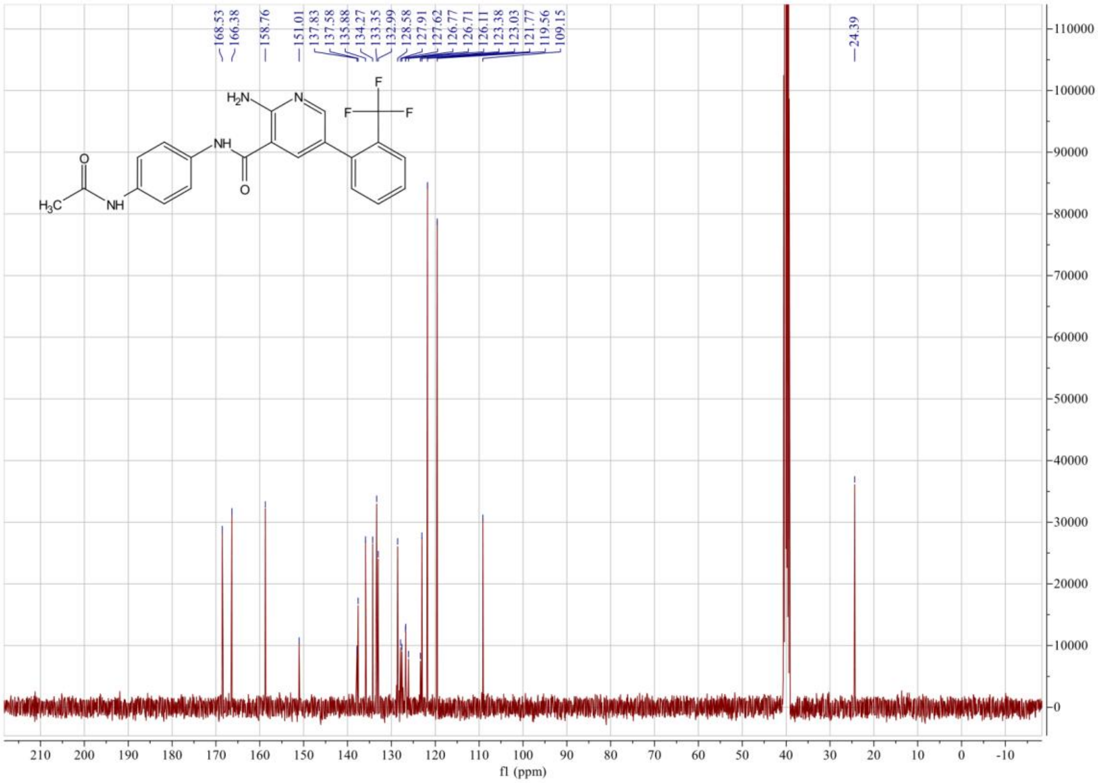
168.53
166.38
158.76
151.01
137.83
137.58
135.88
134.27
133.35
132.99
128.58
127.91
127.62
126.77
126.71
126.11
123.38
123.03
121.77
119.56
109.15
24.39
H2N
N
F
F
F
NH
O
O
H3C
NH
f1 (ppm)

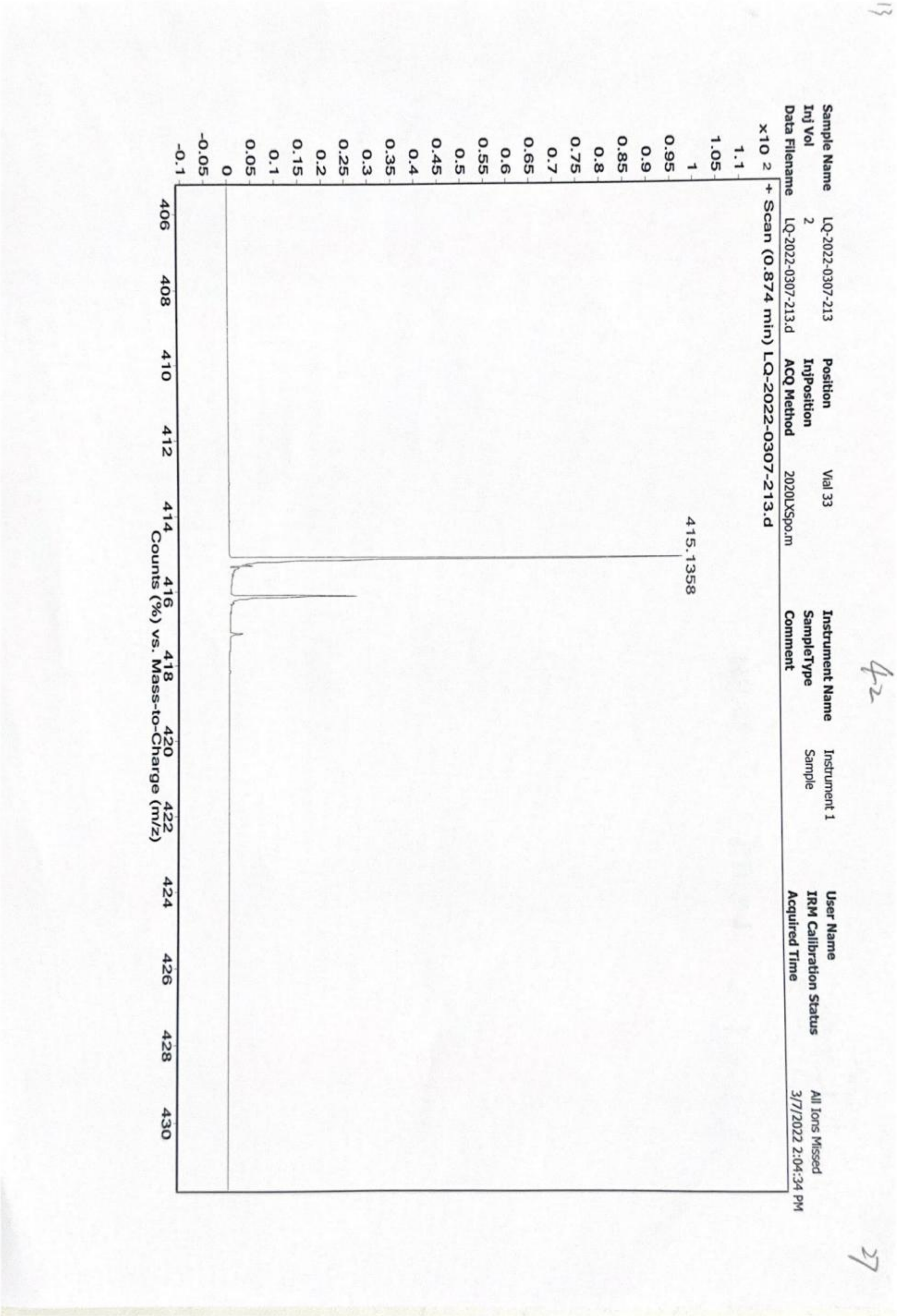

Figure S37. The ^{1}H NMR,^{13}C NMR and ESI-HRMS spectra of compound **42**

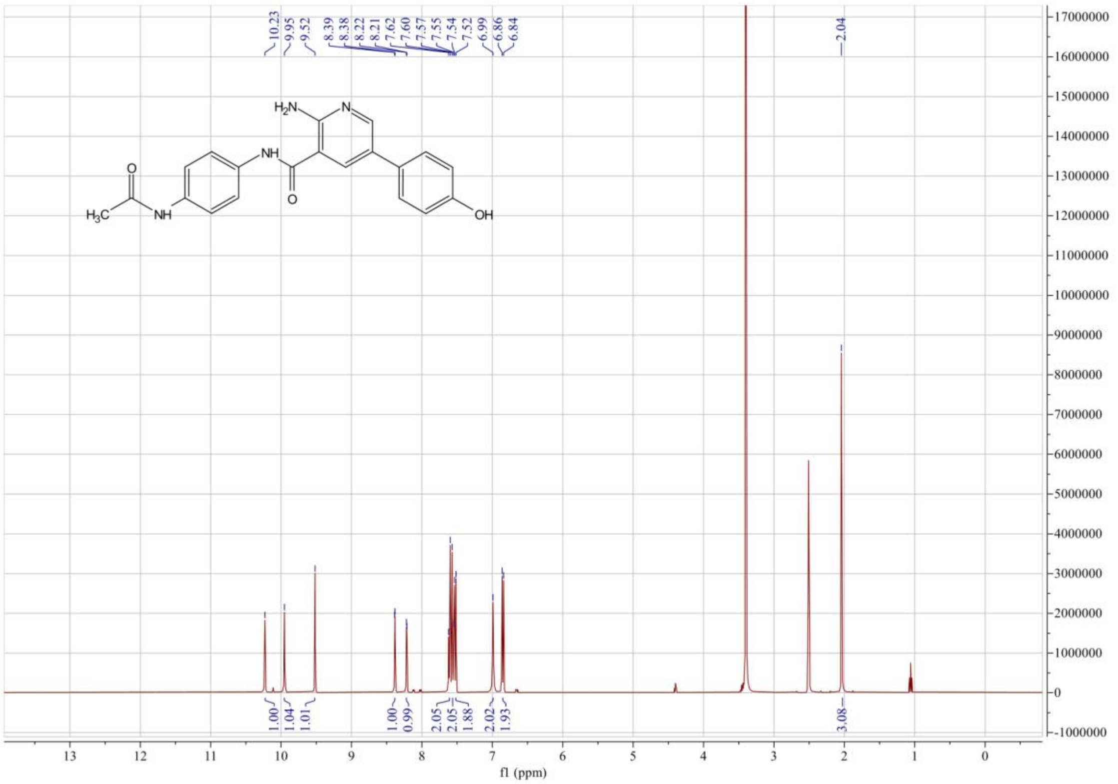

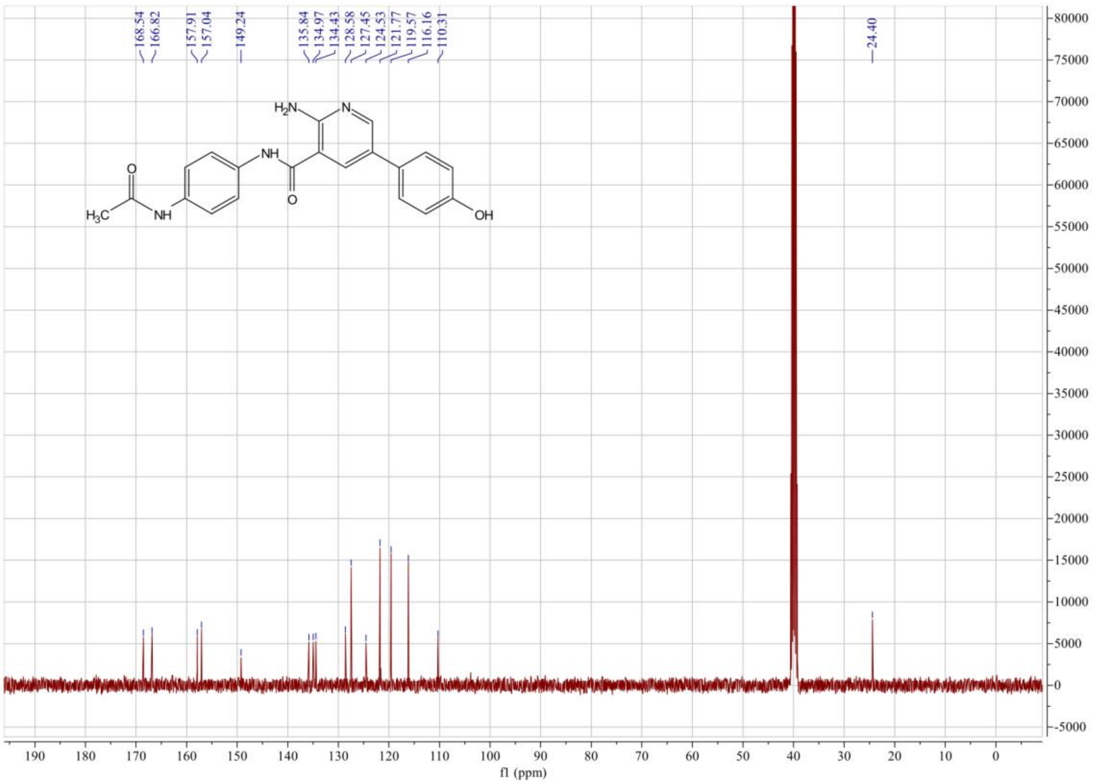

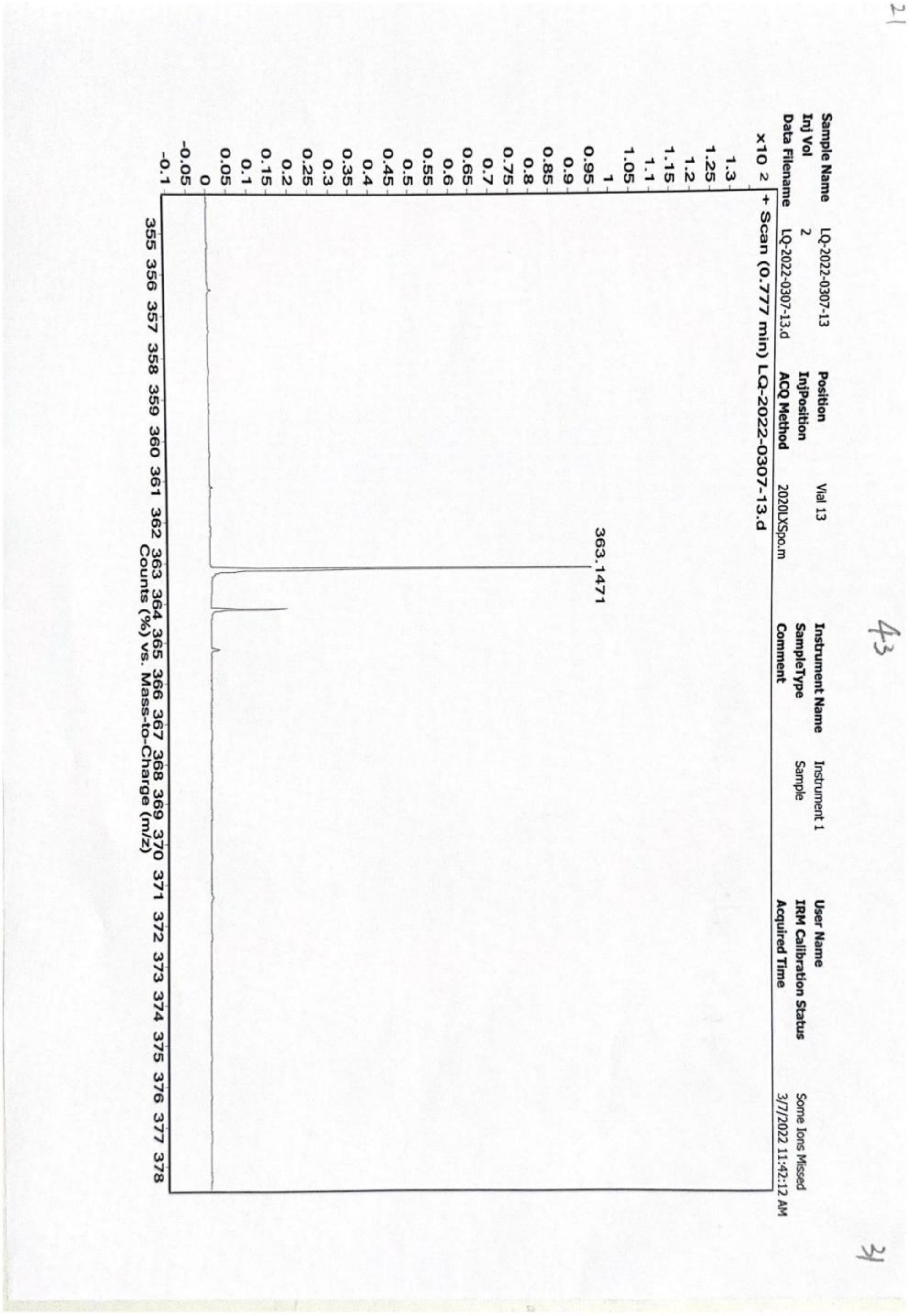

Figure S38. The ^{1}H NMR,^{13}C NMR and ESI-HRMS spectra of compound **43**

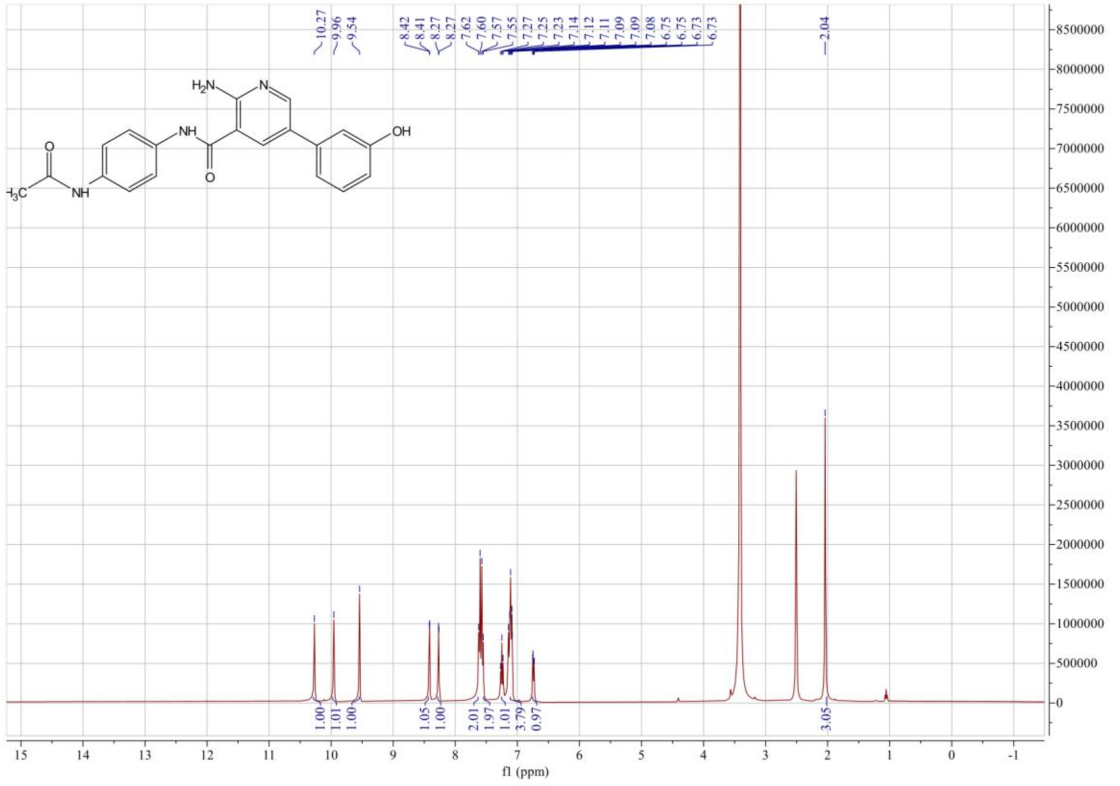

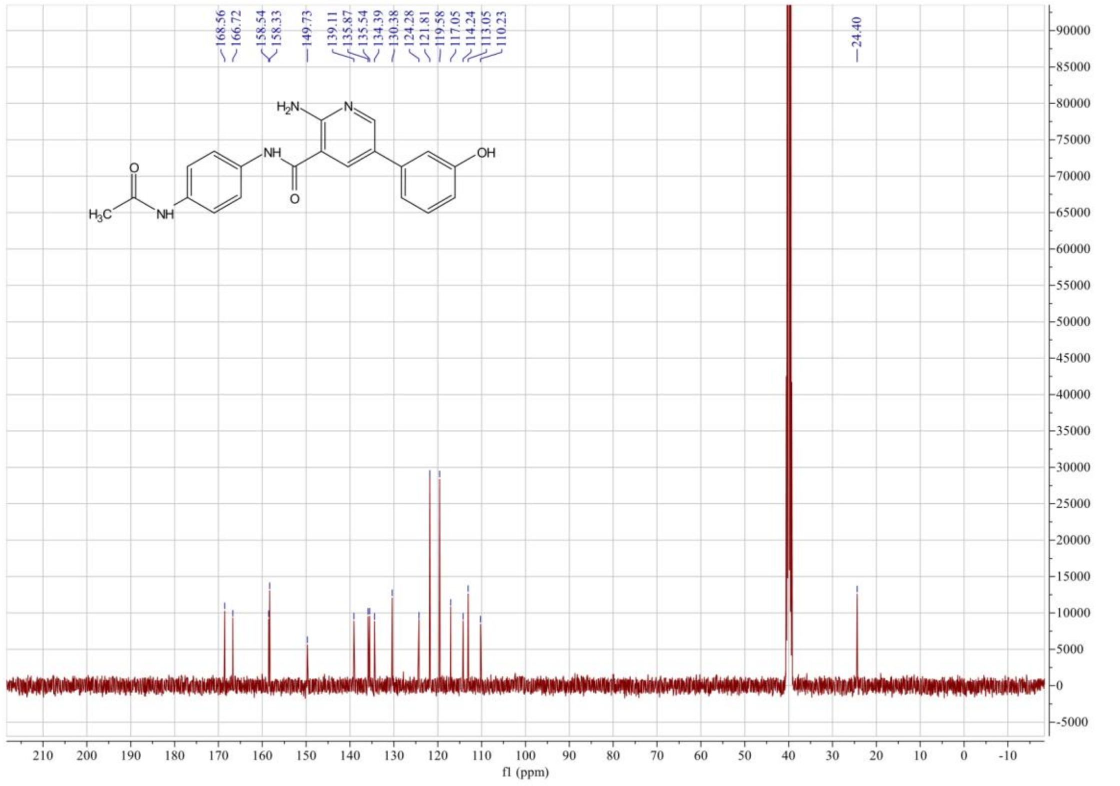

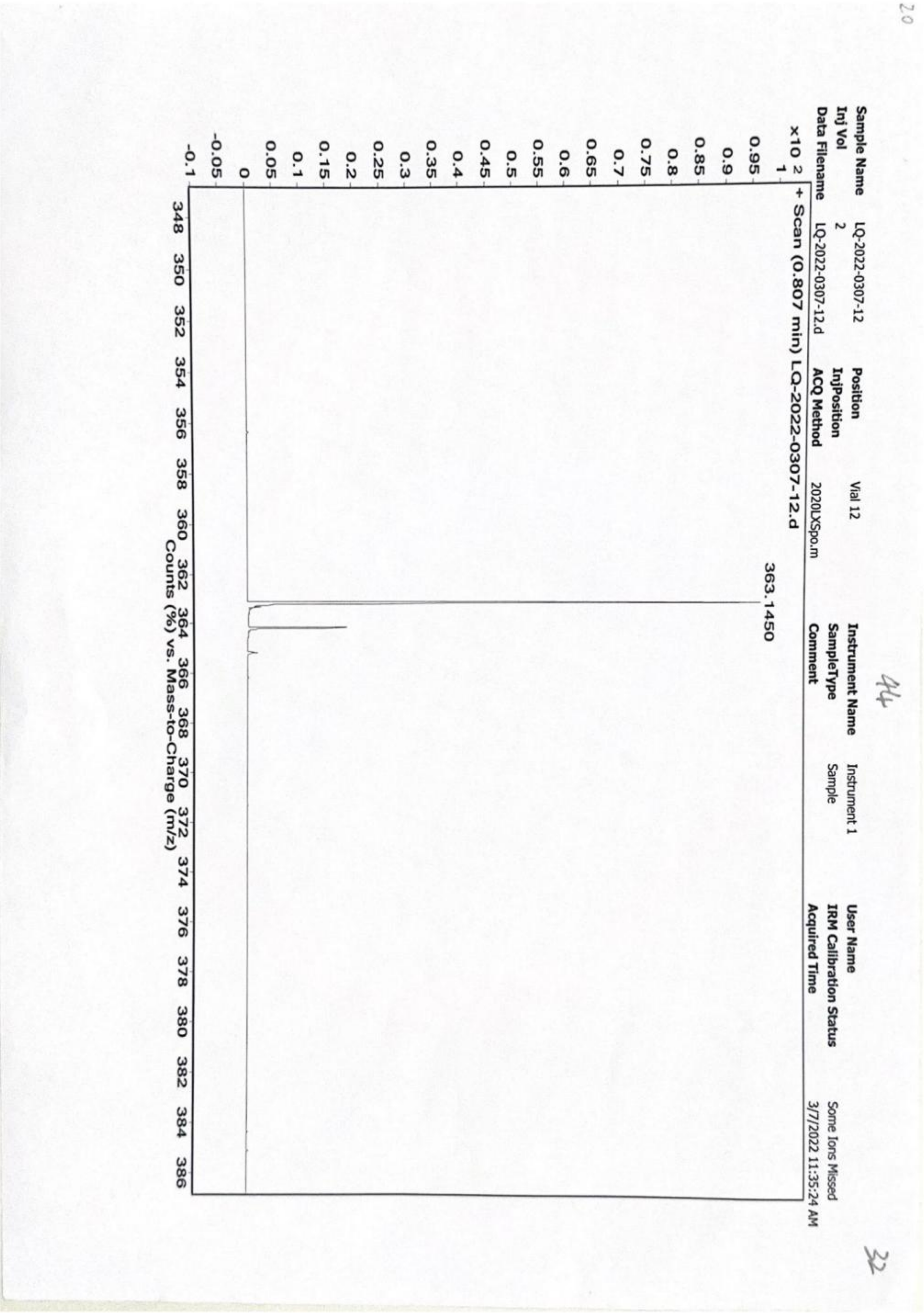

Figure S39. The ^{1}H NMR,^{13}C NMR and ESI-HRMS spectra of compound **44**

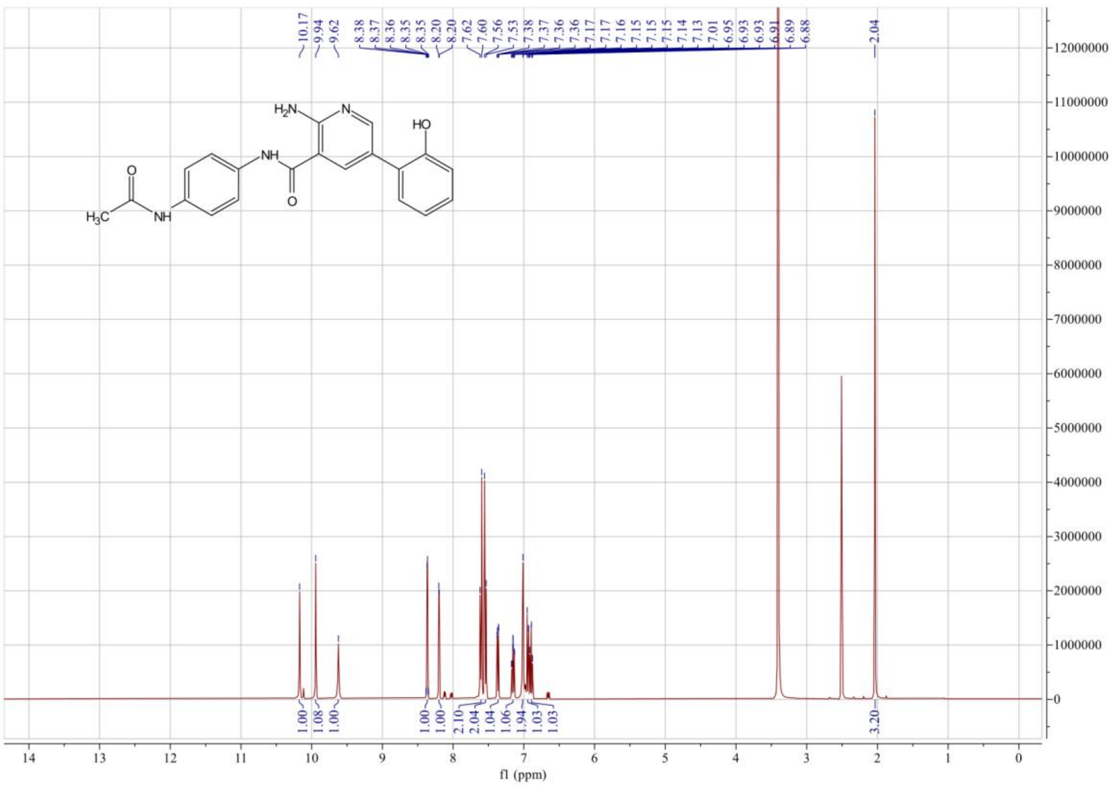
10.17
9.94
9.62
8.38
8.37
8.36
8.35
8.35
8.20
8.20
7.62
7.60
7.56
7.53
7.38
7.37
7.36
7.36
7.17
7.16
7.15
7.15
7.15
7.14
7.13
7.01
6.95
6.93
6.93
6.91
6.89
6.88
2.04
H2N
N
HO
NH
O
O
H3C
NH
1.00
1.08
1.00
1.00
1.00
2.10
2.04
1.04
1.06
1.94
1.03
1.03
3.20
f1 (ppm)

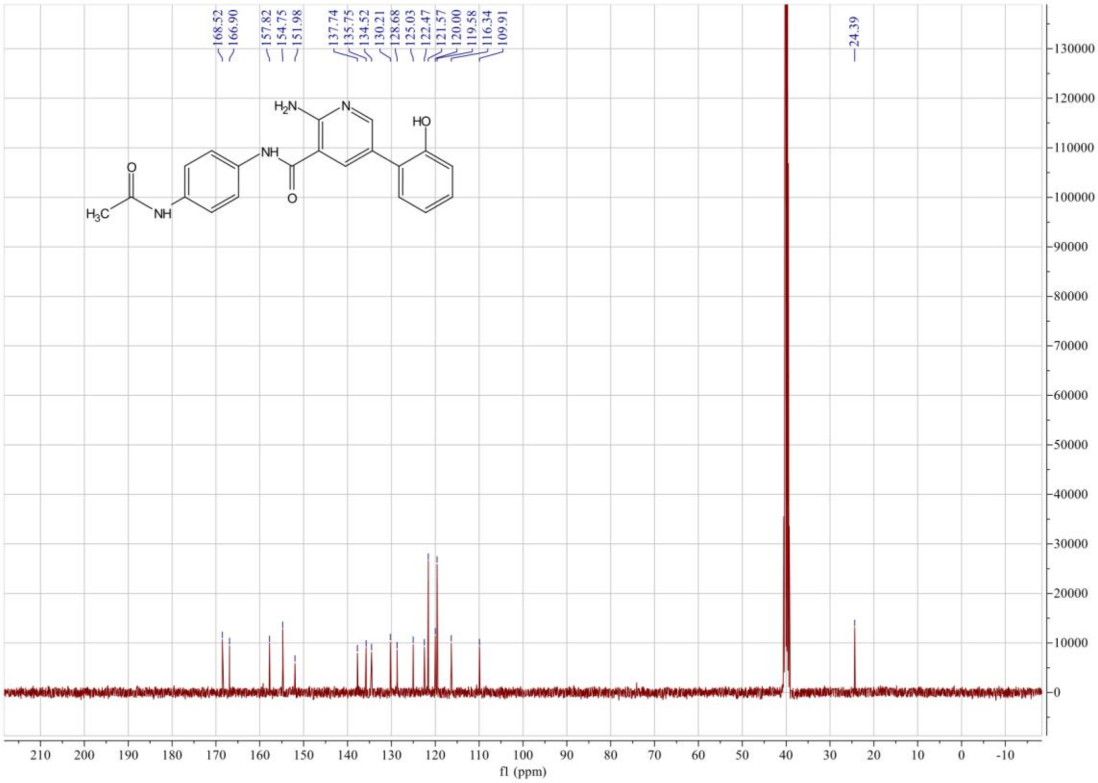
168.52
166.90
157.82
154.75
151.98
137.74
135.75
134.52
130.21
128.68
125.03
122.47
121.57
120.00
119.58
116.34
109.91
24.39
H2N
N
HO
NH
O
O
H3C
NH
f1 (ppm)

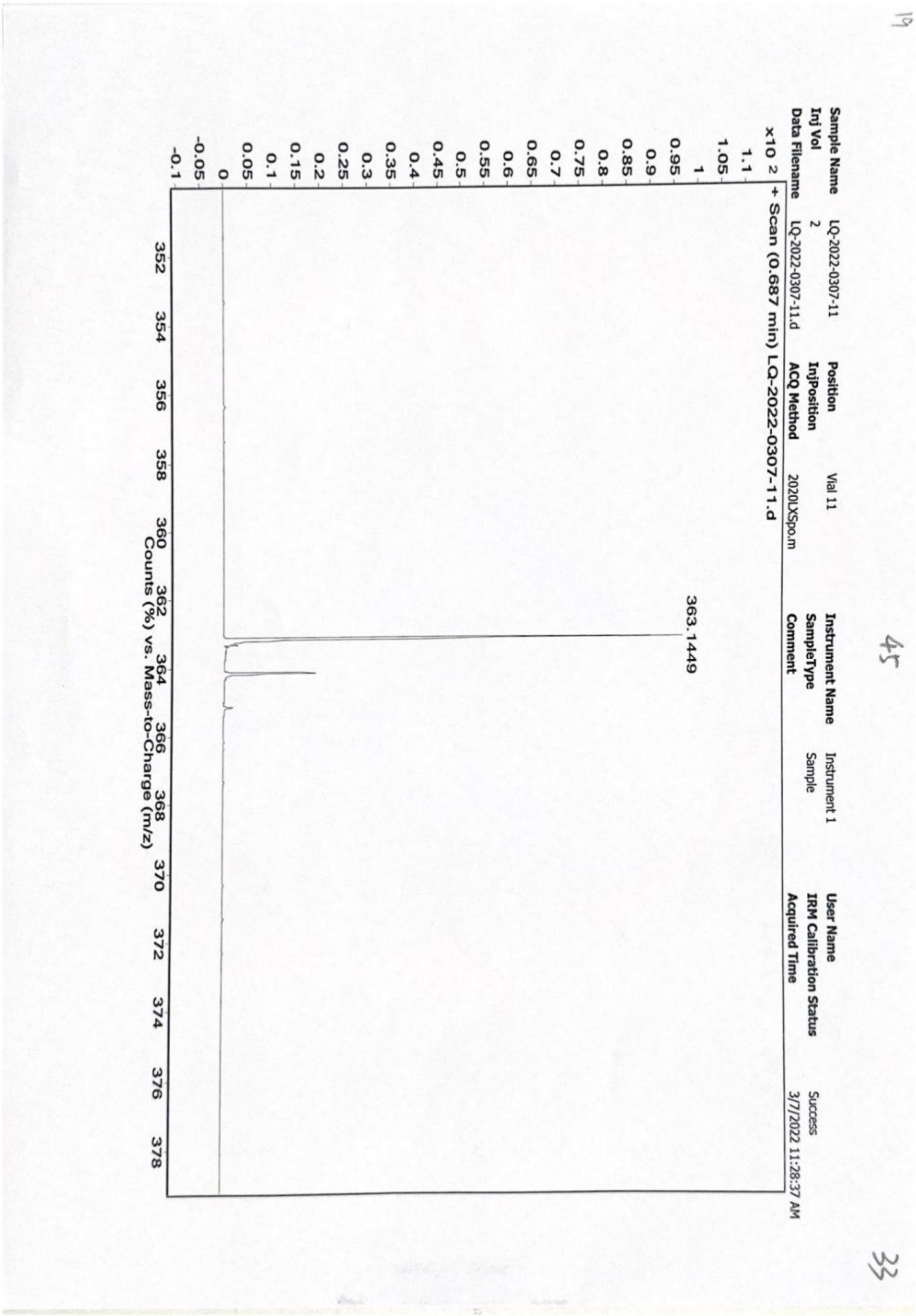

Figure S40. The ^{1}H NMR,^{13}C NMR and ESI-HRMS spectra of compound **45**

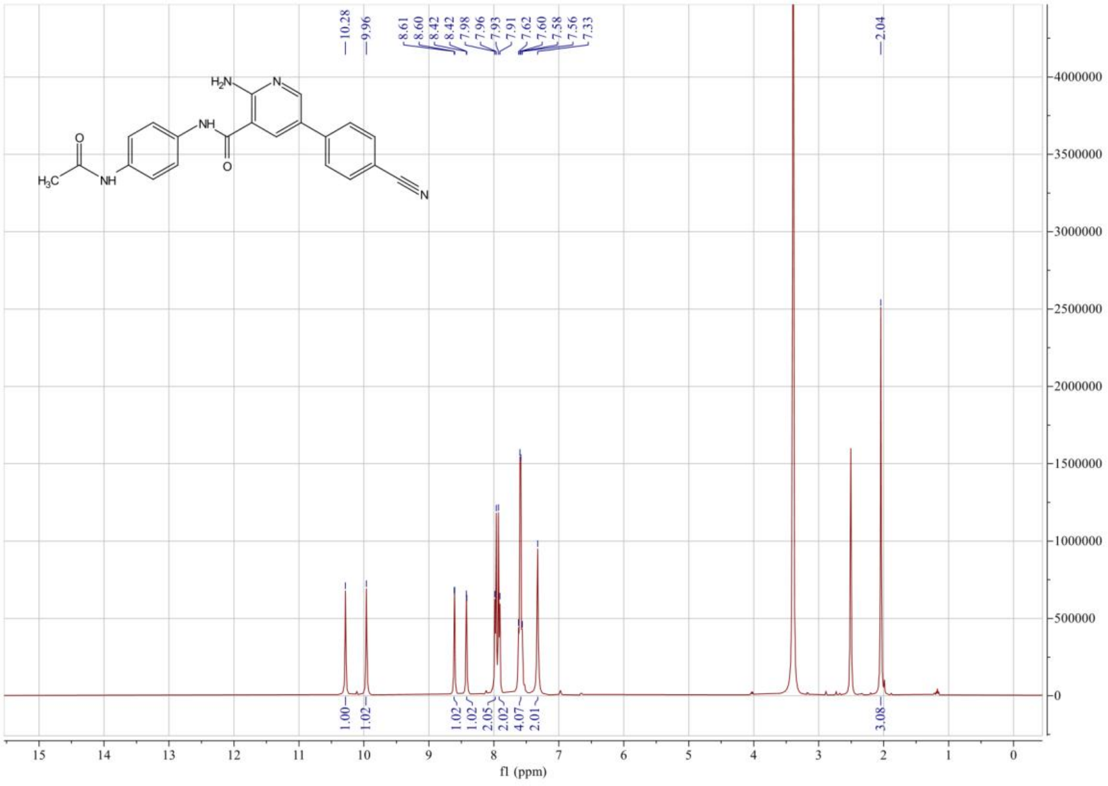
10.28
9.96
8.61
8.60
8.42
8.42
7.98
7.96
7.93
7.91
7.62
7.60
7.58
7.56
7.33
2.04
1.00
1.02
1.02
1.02
2.05
2.02
4.07
2.01
3.08
f1 (ppm)

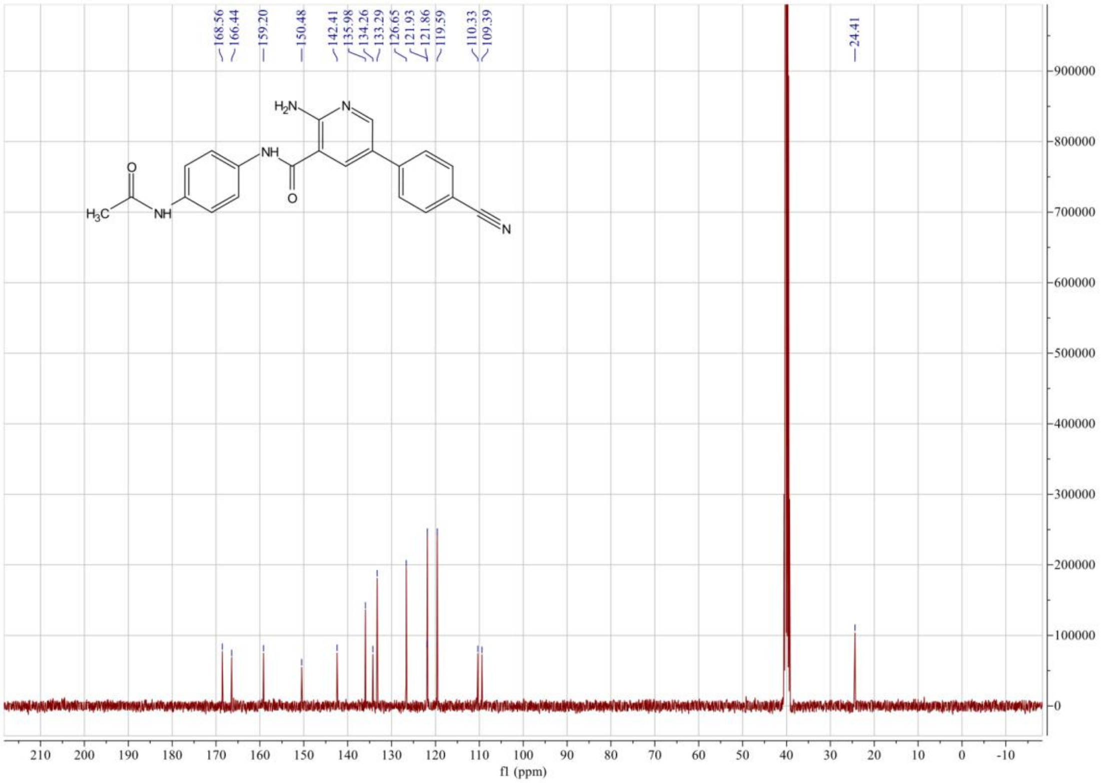
168.56
166.44
159.20
150.48
142.41
135.98
134.26
133.29
126.65
121.93
121.86
119.59
110.33
109.39
24.41
f1 (ppm)

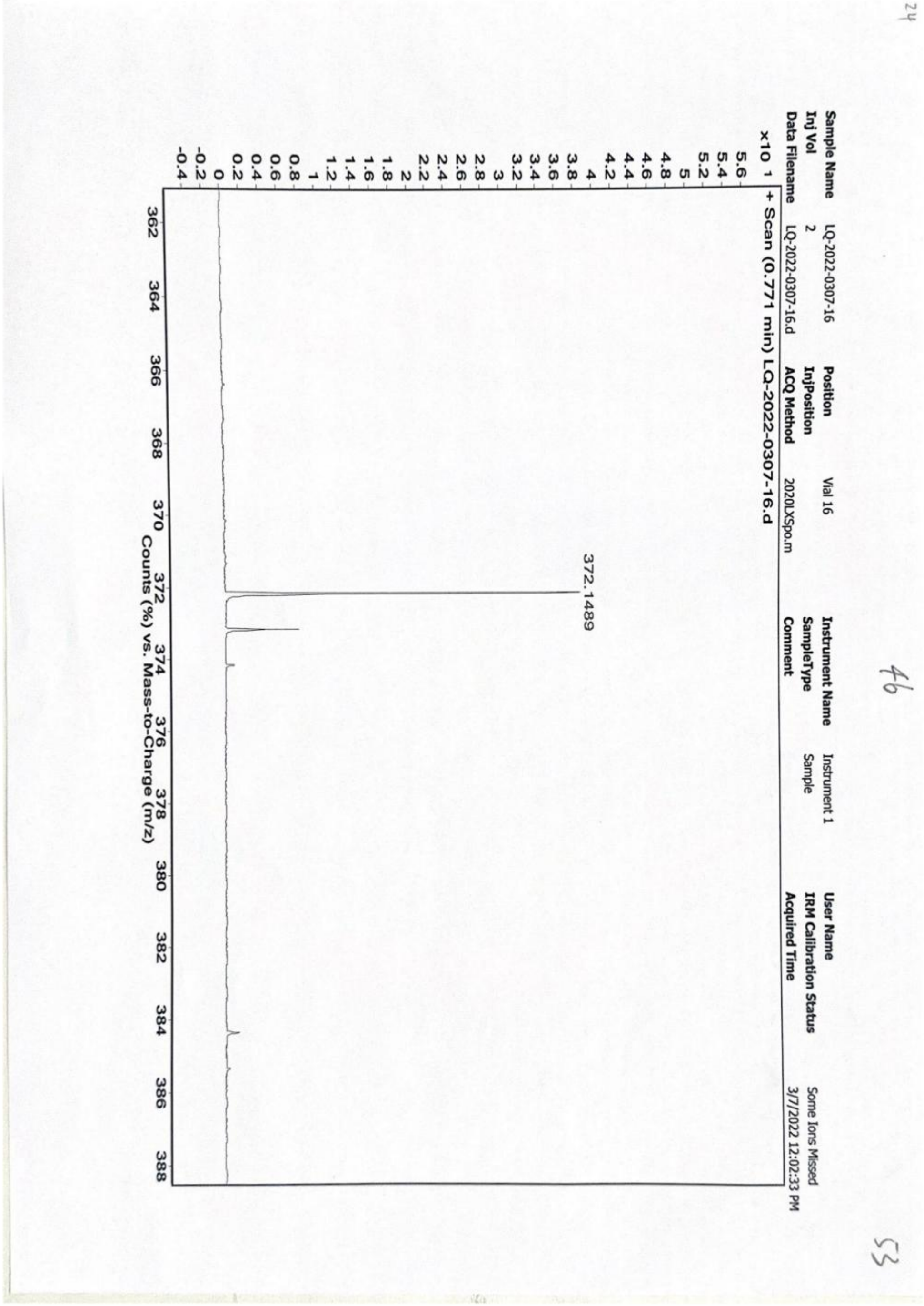

Figure S41. The ^{1}H NMR,^{13}C NMR and ESI-HRMS spectra of compound **46**

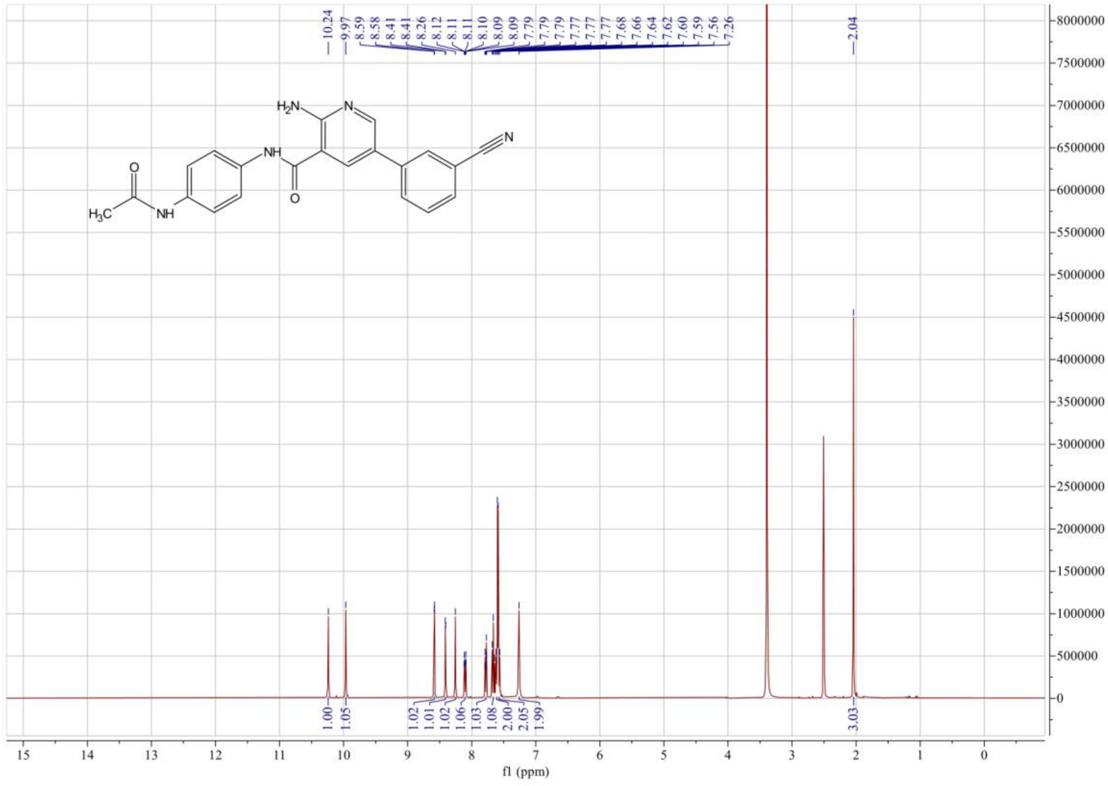
10.24
9.97
8.59
8.58
8.41
8.26
8.12
8.11
8.11
8.10
8.09
8.09
7.79
7.79
7.79
7.77
7.77
7.77
7.68
7.66
7.64
7.62
7.60
7.59
7.56
7.26
2.04
H2N
N
N
NH
O
O
H3C
NH
1.00
1.05
1.02
1.01
1.02
1.06
1.03
1.08
2.00
2.05
1.99
3.03
f1 (ppm)

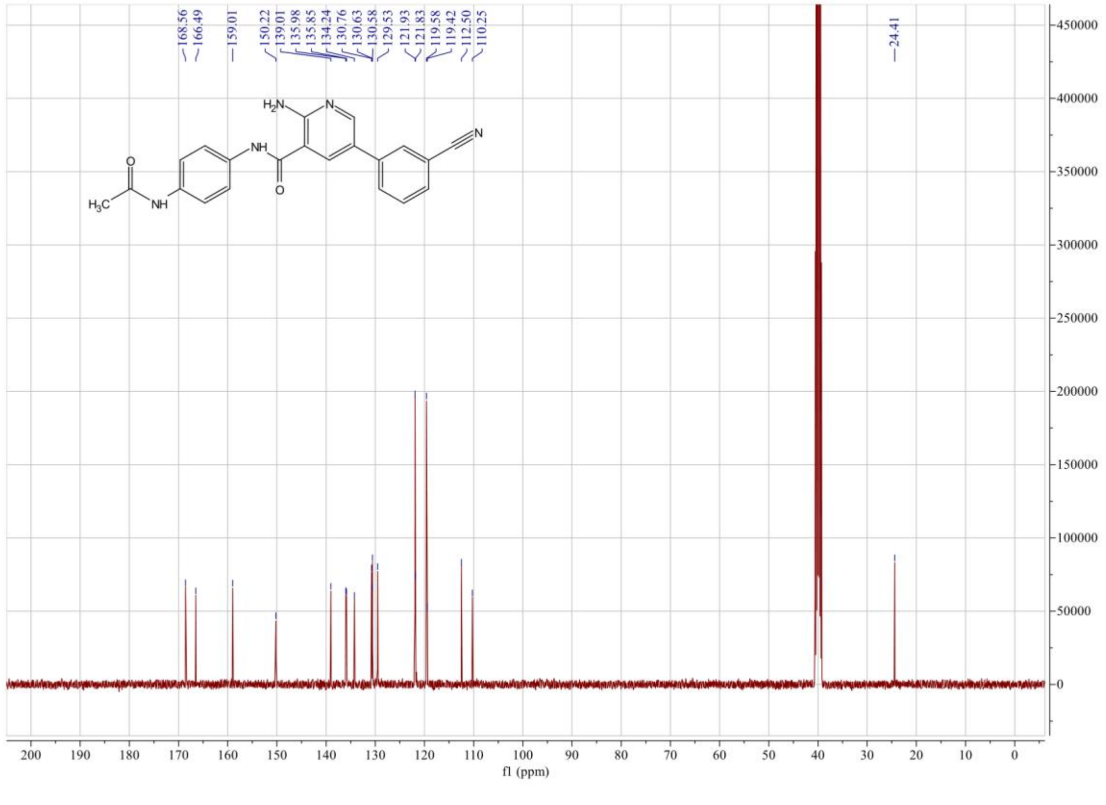
168.56
166.49
159.01
150.22
139.01
135.98
135.85
134.24
130.76
130.63
130.58
129.53
121.93
121.83
119.58
119.42
112.50
110.25
24.41
H2N
N
N
NH
O
O
H3C
NH
f1 (ppm)

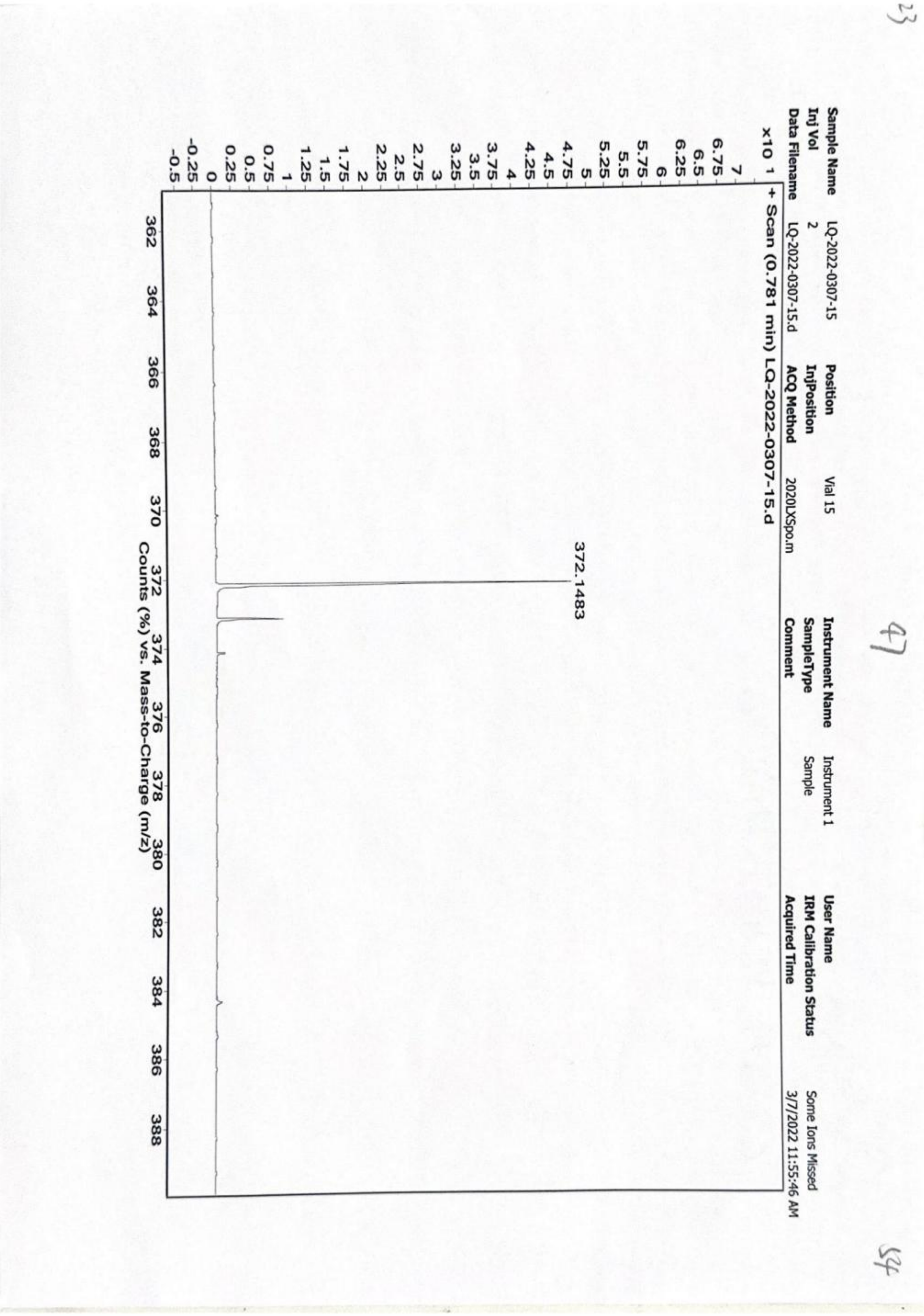

Figure S42. The ^{1}H NMR,^{13}C NMR and ESI-HRMS spectra of compound **47**

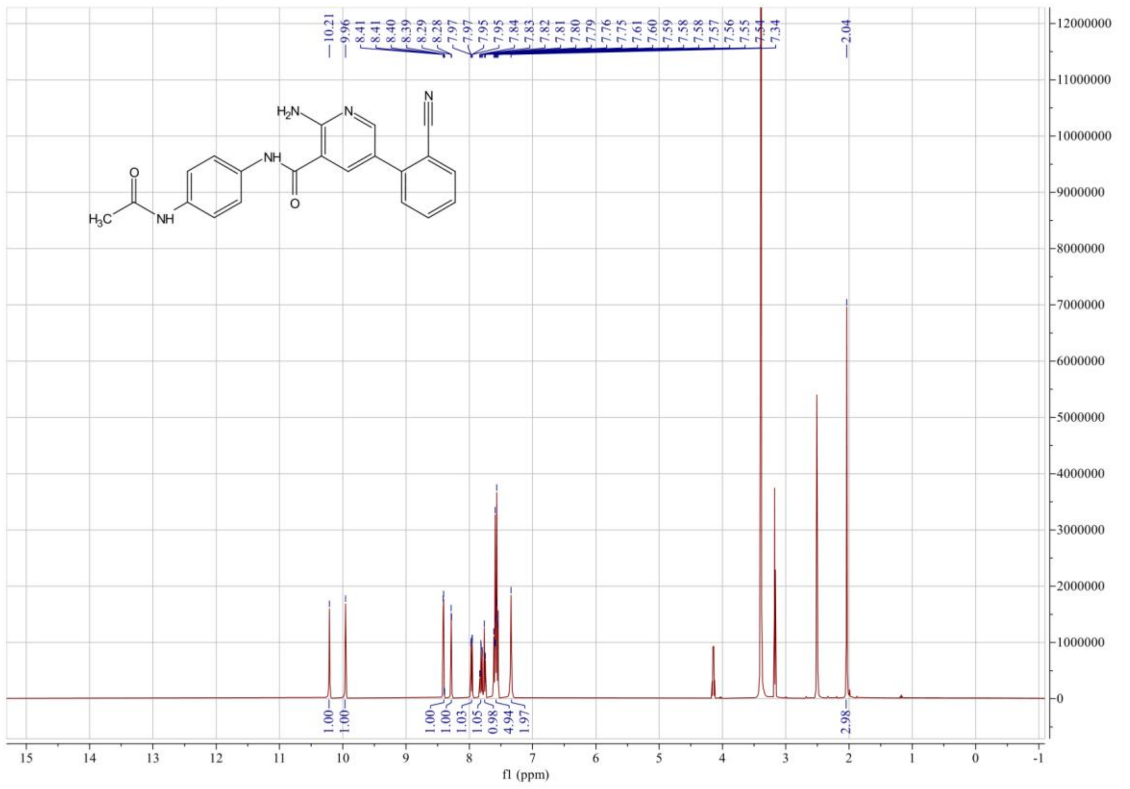

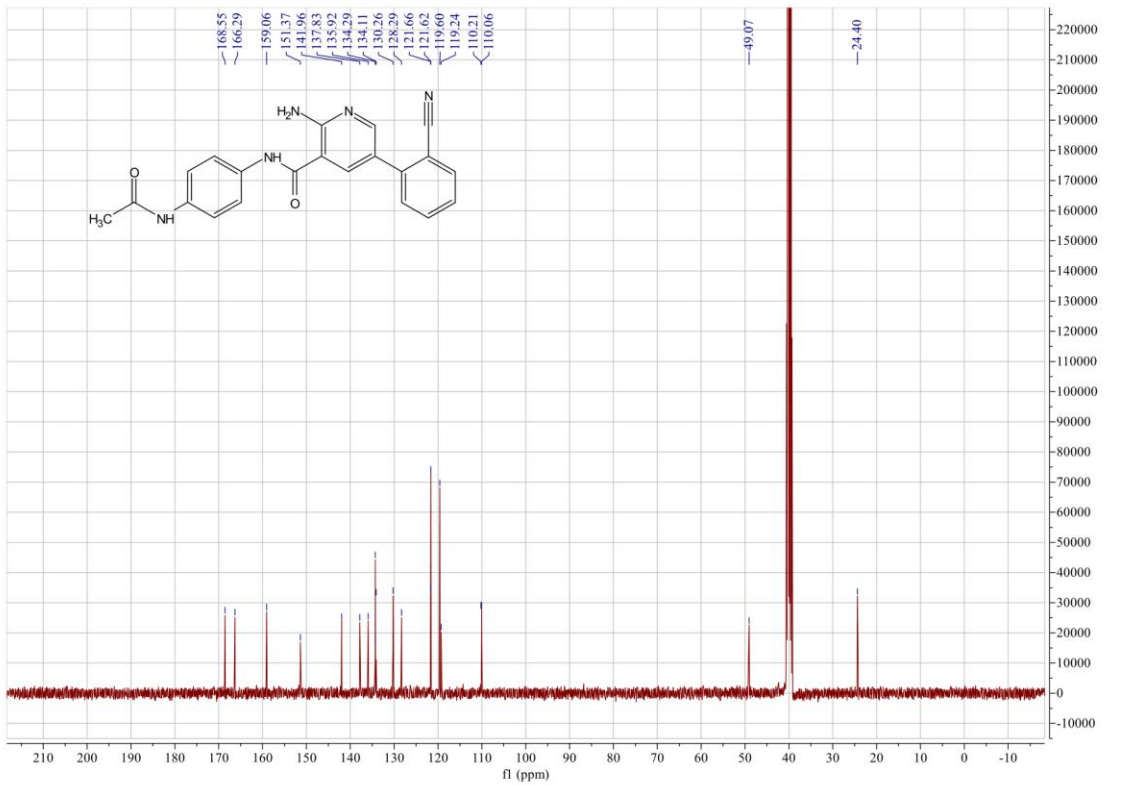

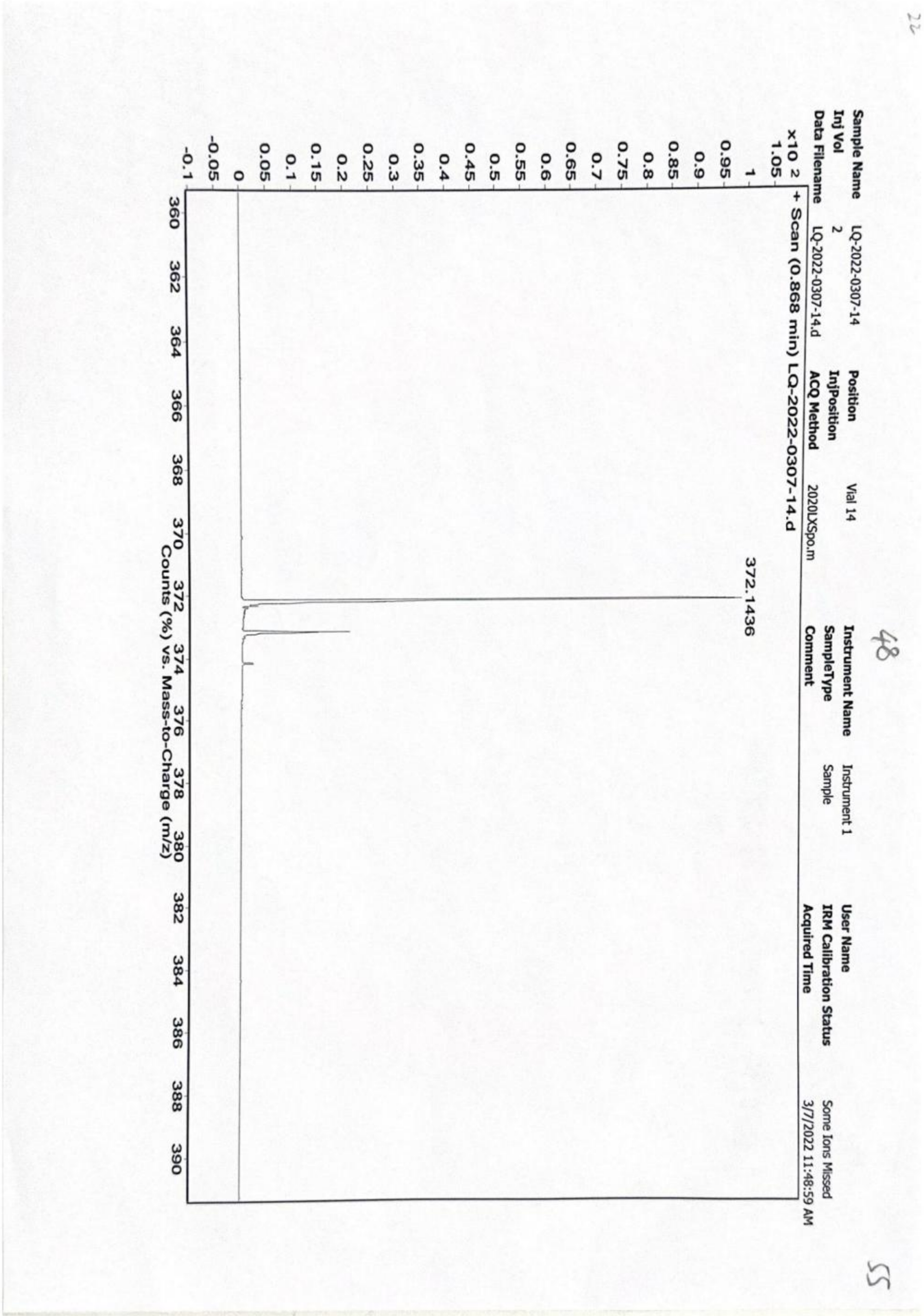

Figure S43. The ^{1}H NMR,^{13}C NMR and ESI-HRMS spectra of compound **48**

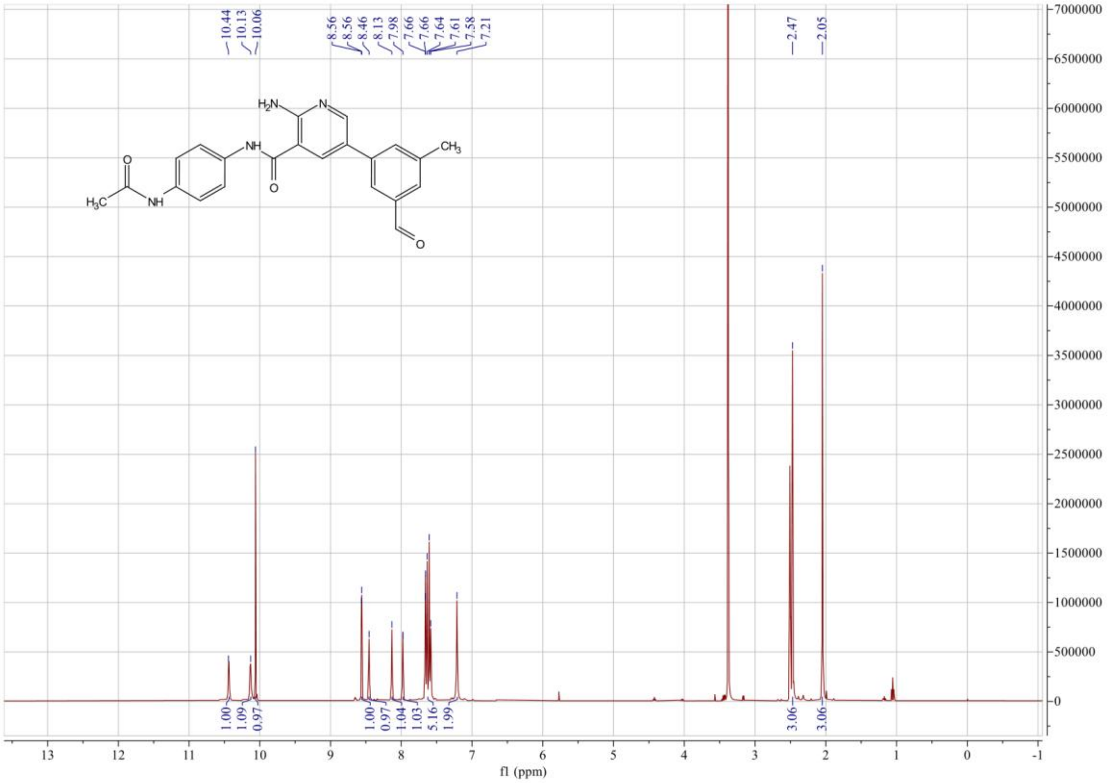
10.44
10.13
10.06
8.56
8.56
8.46
8.13
7.98
7.66
7.66
7.64
7.61
7.58
7.21
2.47
2.05
1.00
1.09
0.97
1.00
0.97
1.04
1.03
5.16
1.99
3.06
3.06
fl (ppm)

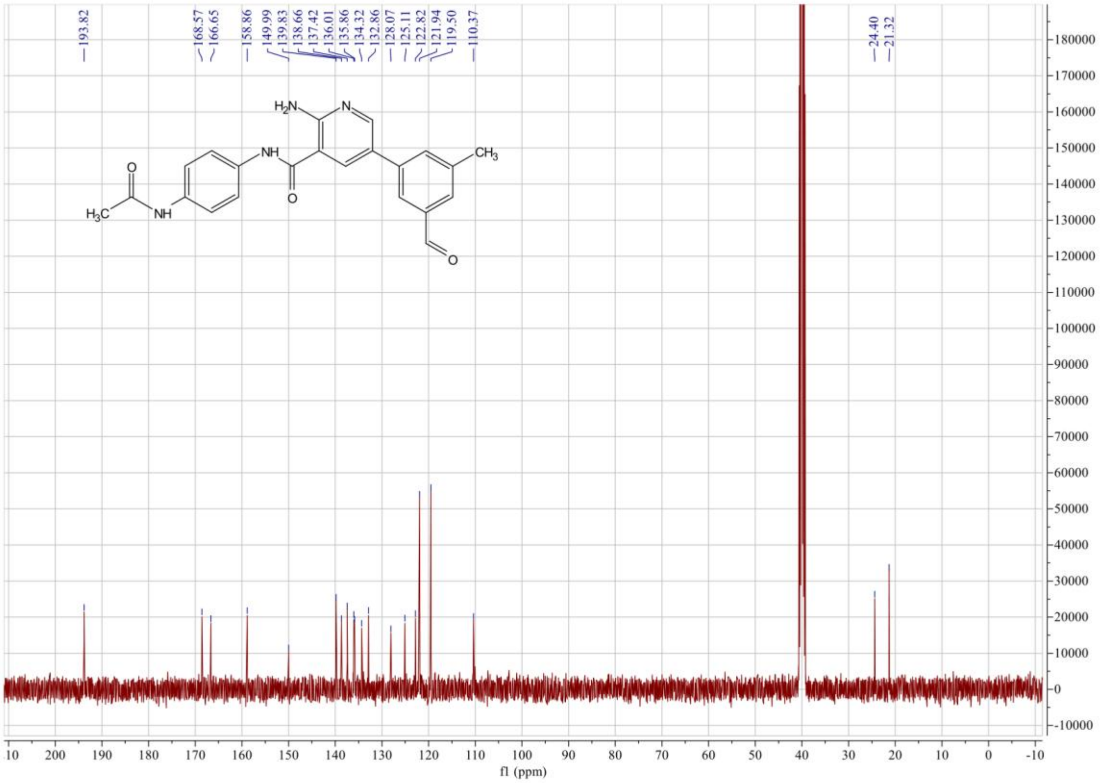
193.82
168.57
166.65
158.86
149.99
139.83
138.66
137.42
136.01
135.86
134.32
132.86
128.07
125.11
122.82
121.94
119.50
110.37
24.40
21.32
fl (ppm)

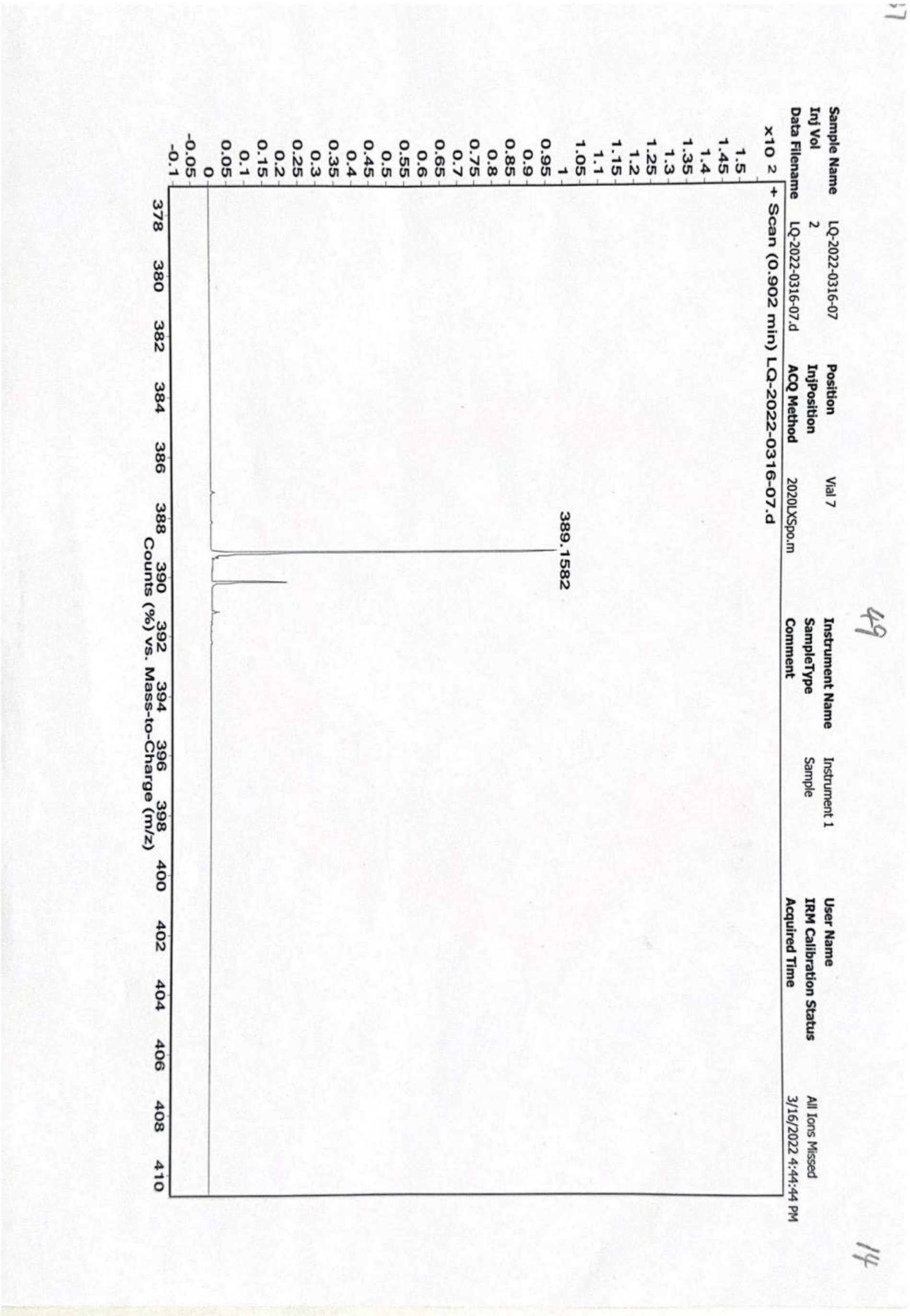

Figure S44. The ^{1}H NMR,^{13}C NMR and ESI-HRMS spectra of compound **49**

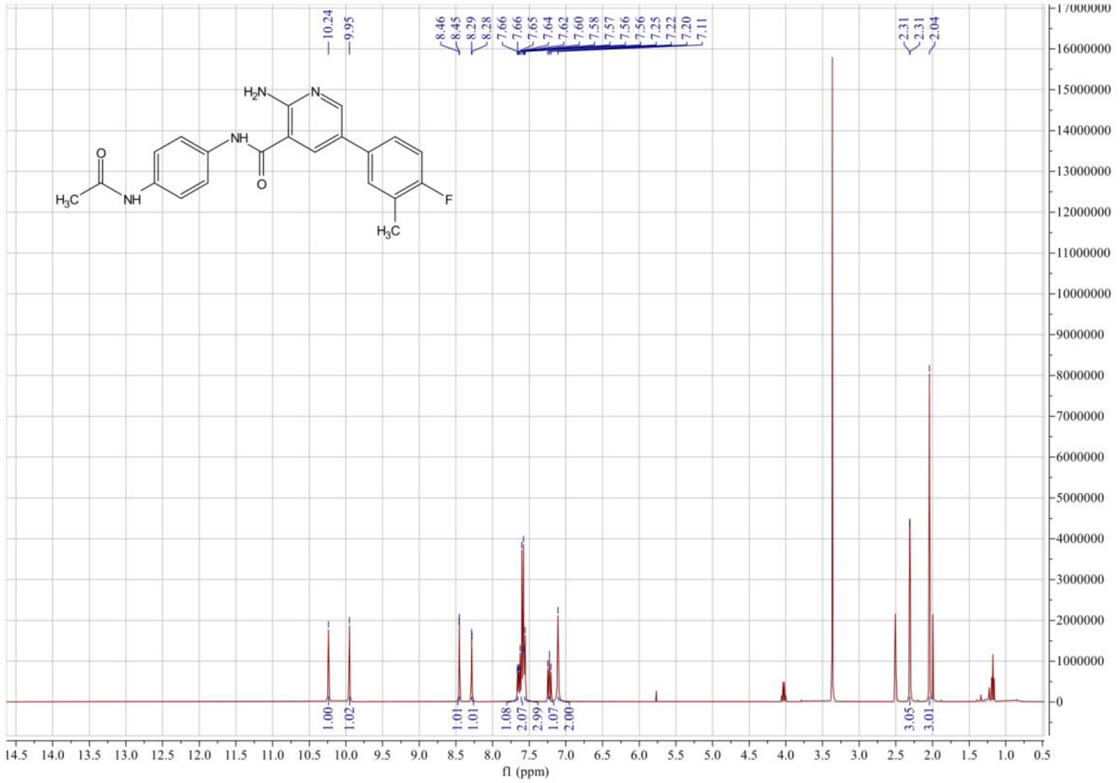

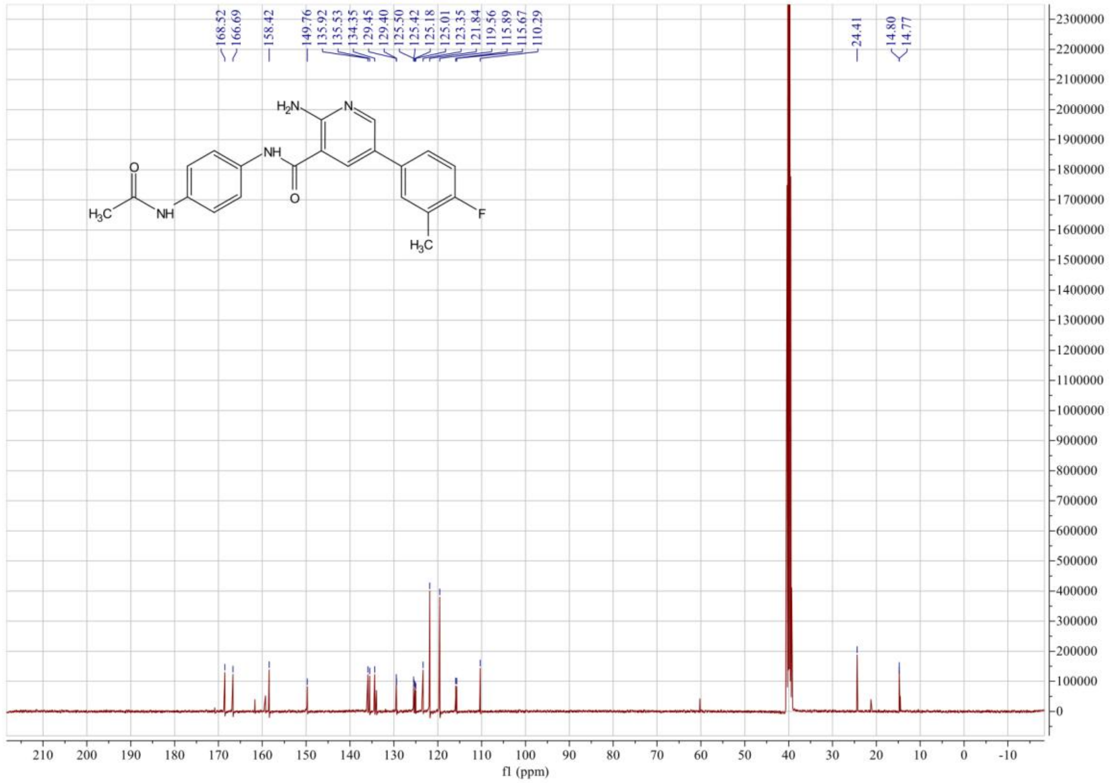

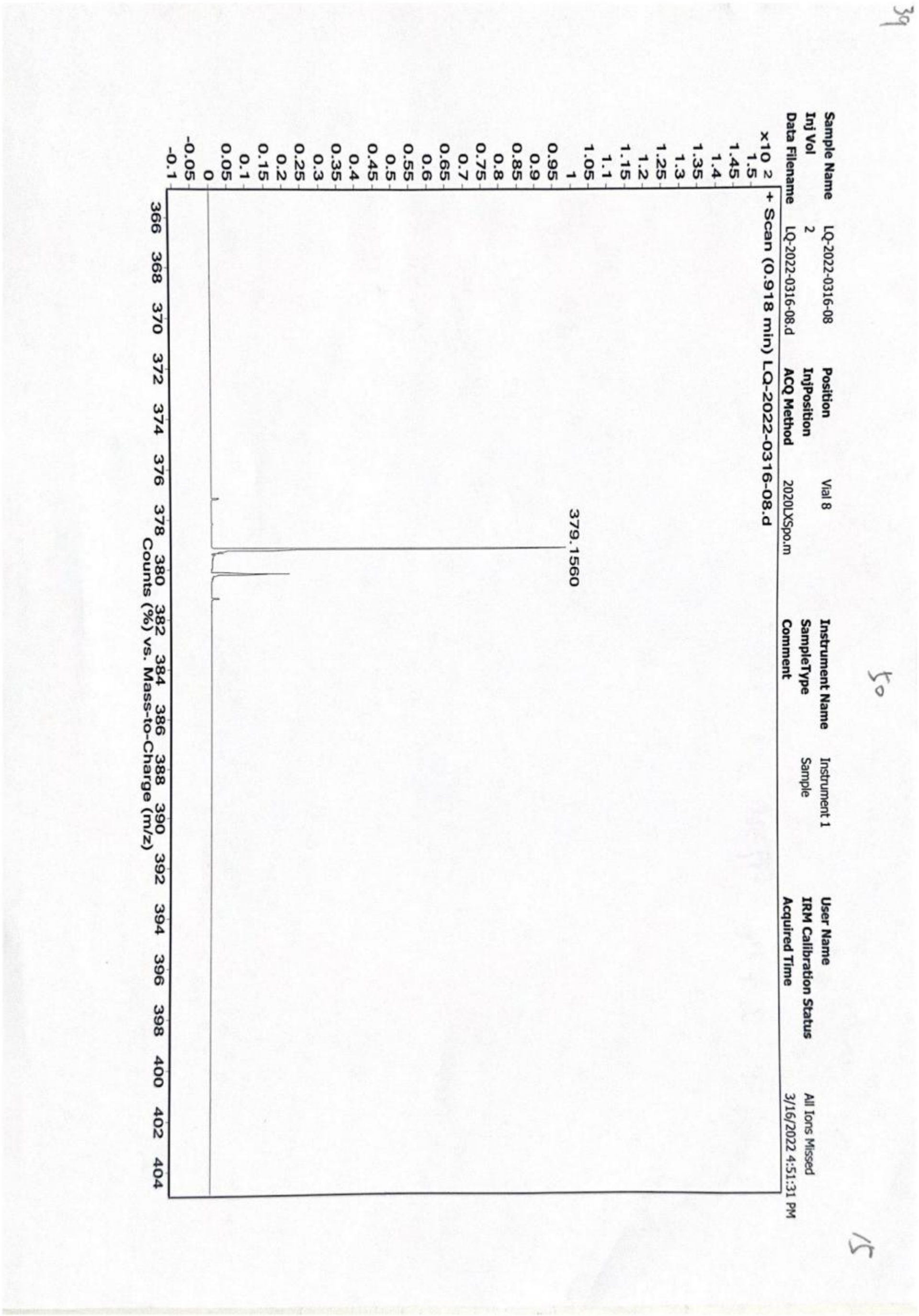

Figure S45. The ^{1}H NMR,^{13}C NMR and ESI-HRMS spectra of compound **50**

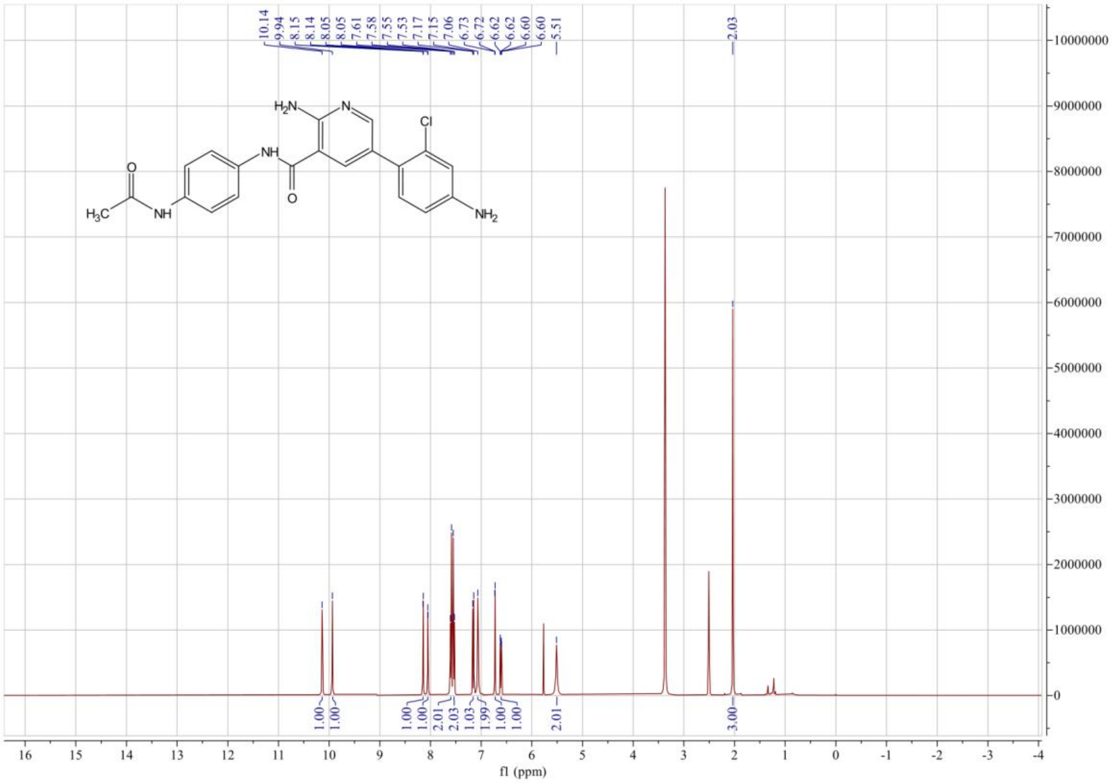

10.14 9.94 8.15 8.14 8.05 8.05 7.61 7.58 7.55 7.53 7.17 7.15 7.06 6.73 6.72 6.62 6.62 6.60 5.51
2.03
1.00 1.00 1.00 1.00 2.01 2.03 1.03 1.99 1.00 1.00 2.01 3.00
f1 (ppm)

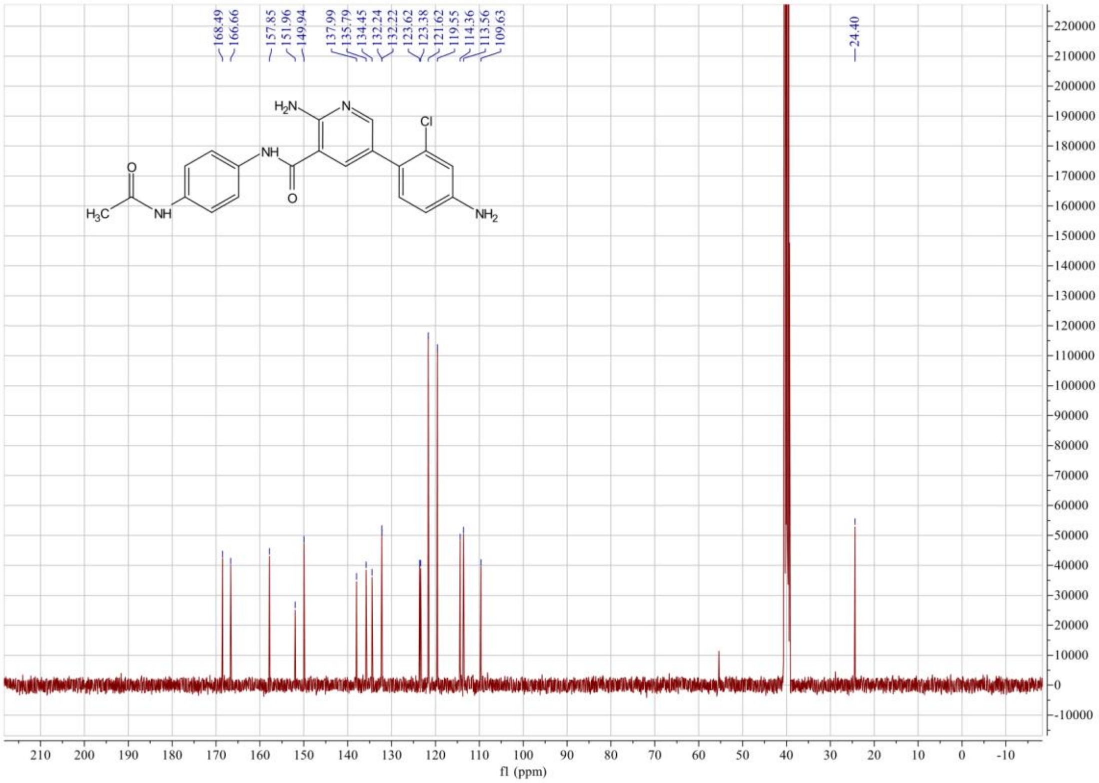

168.49 166.66 157.85 151.96 149.94 137.99 135.79 134.45 132.24 132.22 123.62 123.38 121.62 119.55 114.36 113.56 109.63
24.40
f1 (ppm)

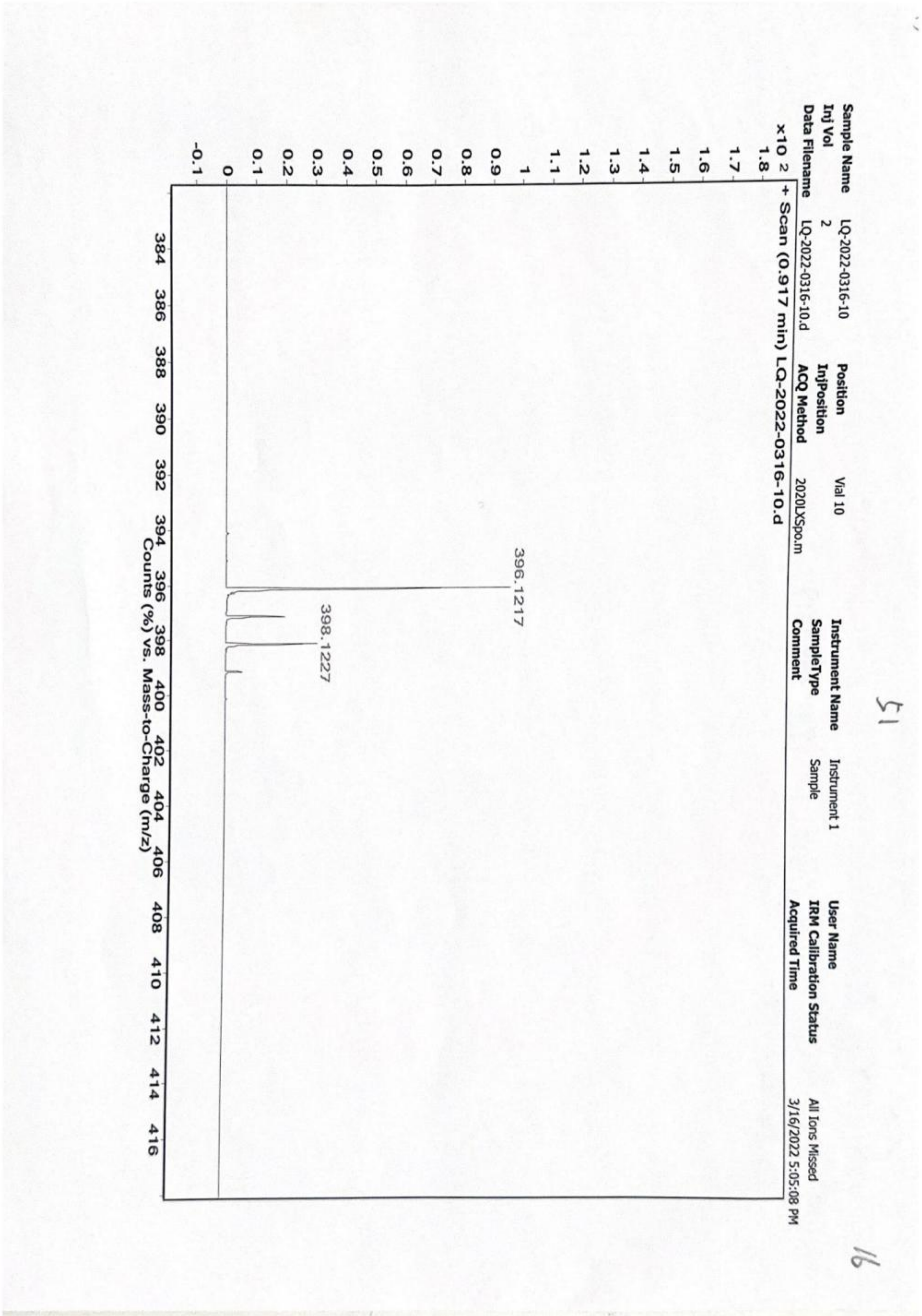

Figure S46. The ^{1}H NMR,^{13}C NMR and ESI-HRMS spectra of compound **51**

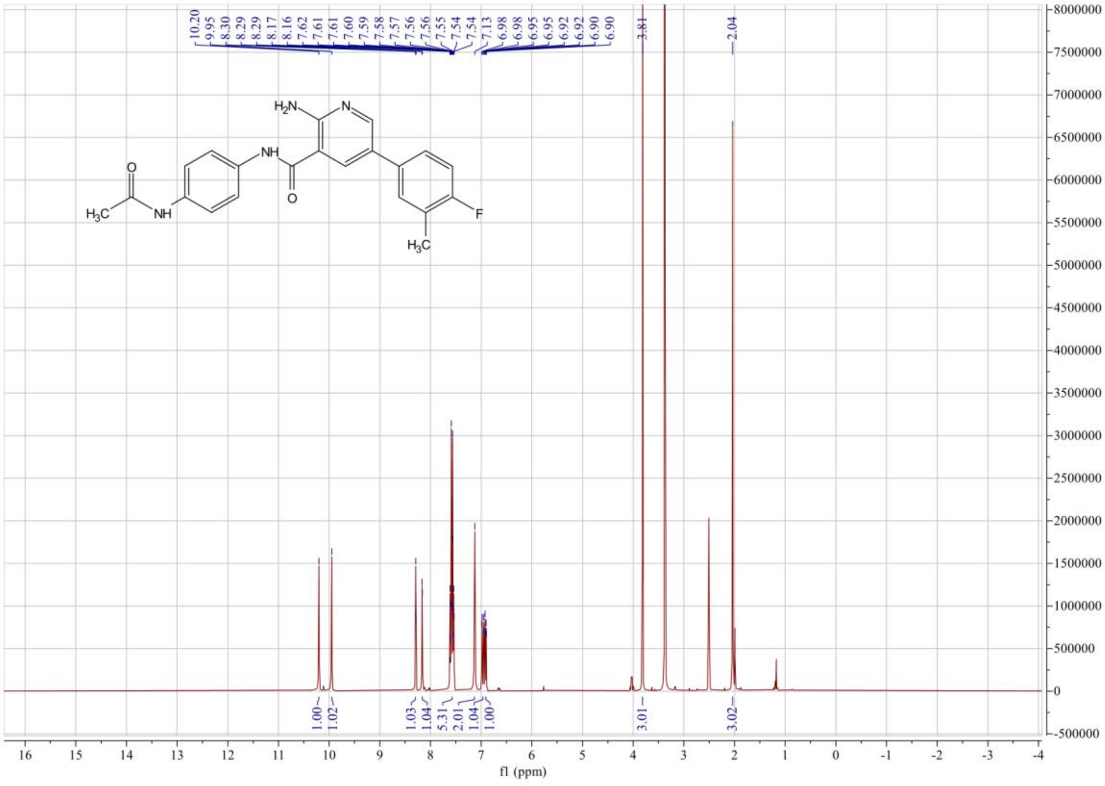

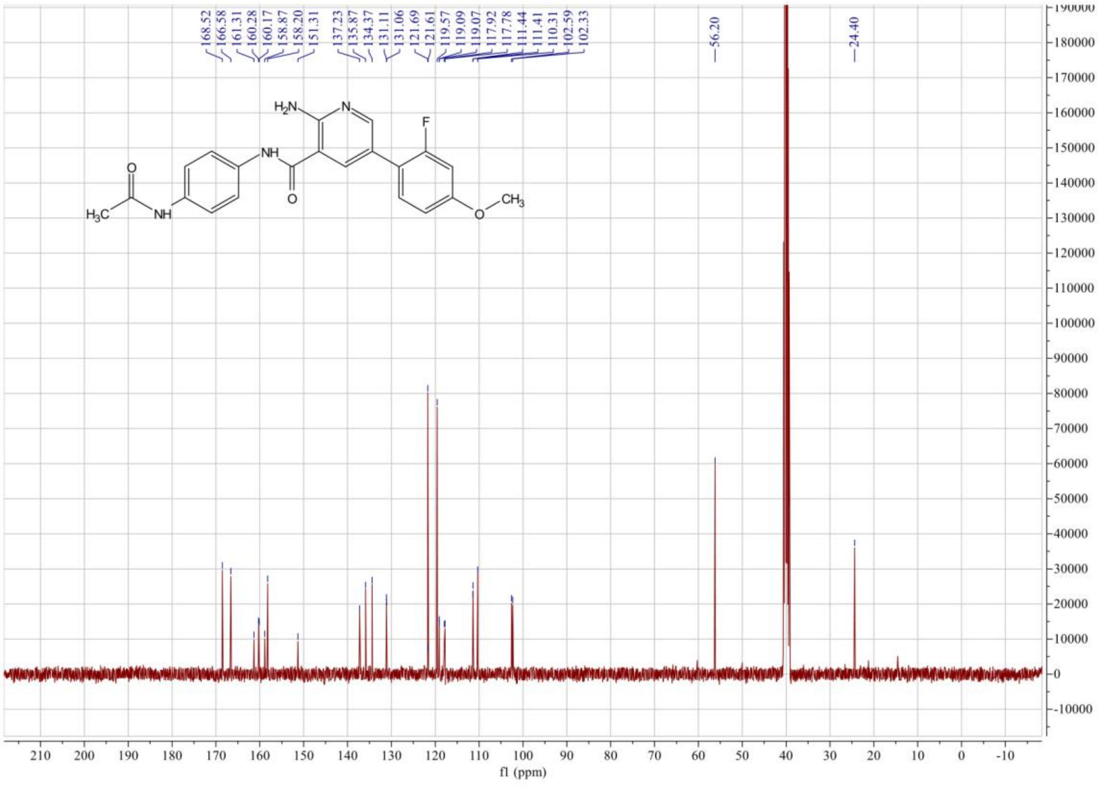

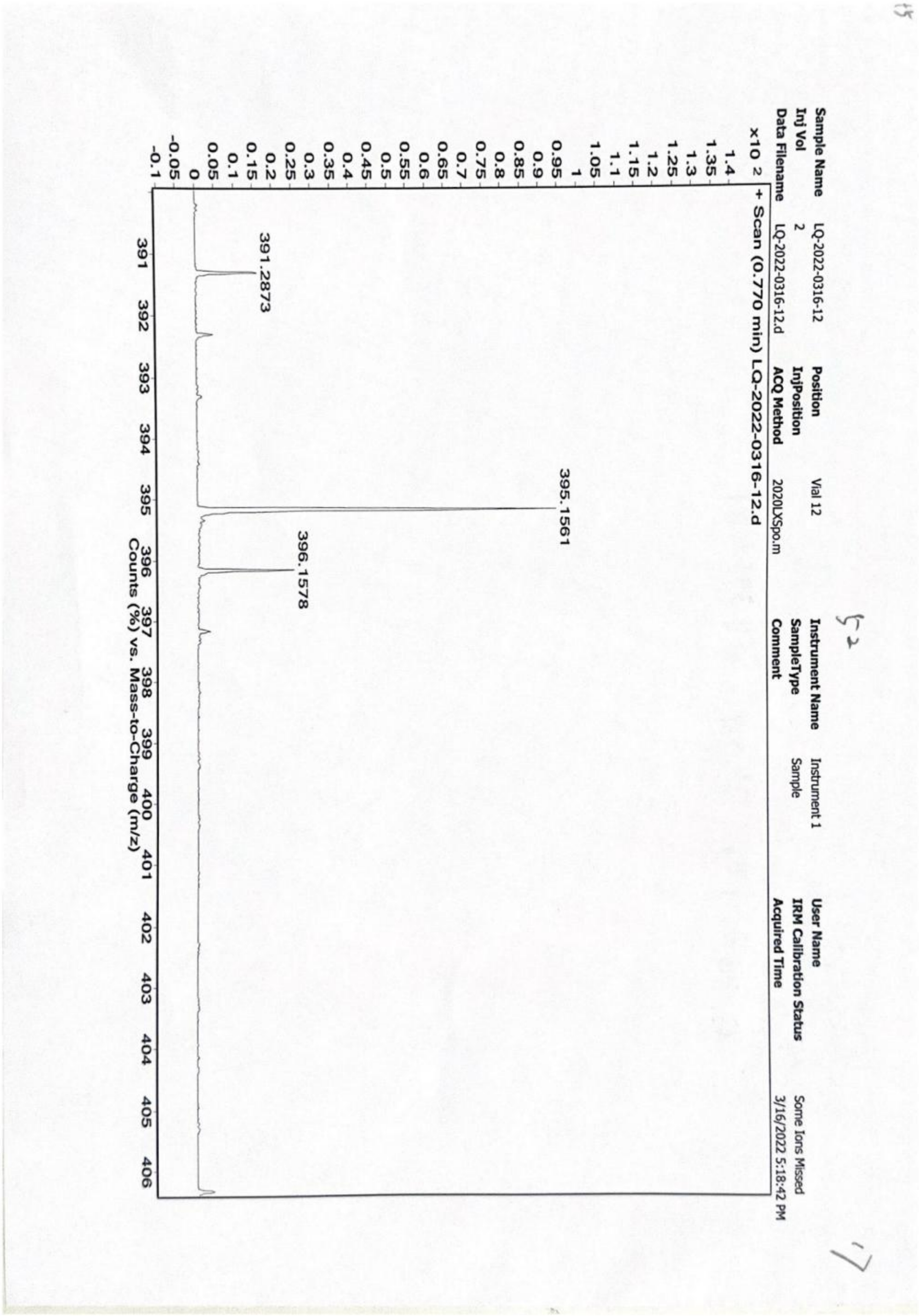

Figure S47. The ^{1}H NMR,^{13}C NMR and ESI-HRMS spectra of compound **52**

The purity of compound CX51

NO.	RT（min）	eluted condition $CH_3OH:H_2O$	Area%
51	5.439	1:1	97.944

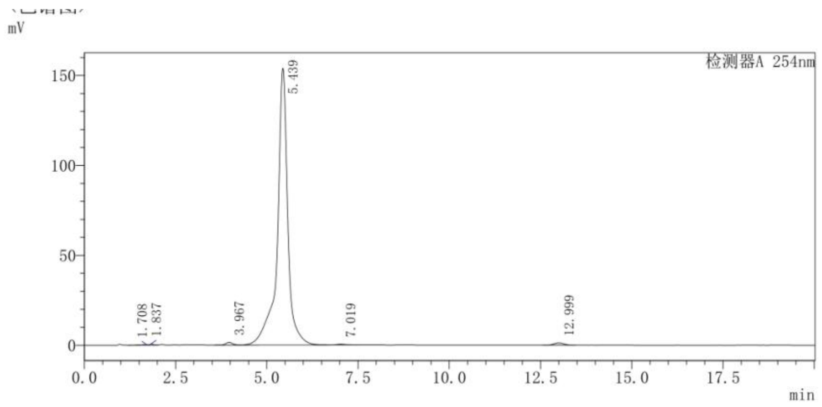

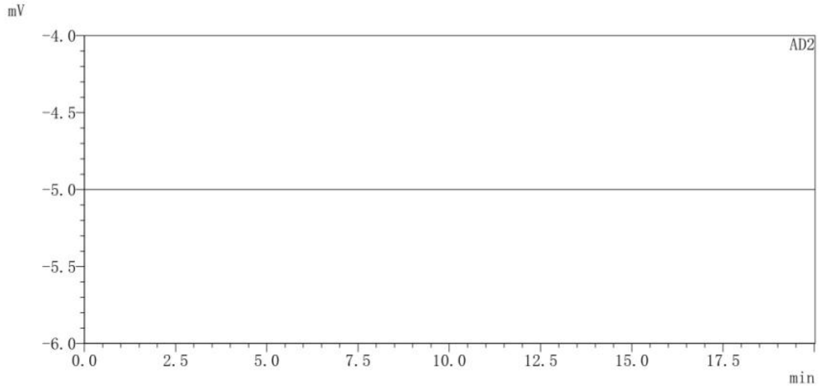

Supplement: Supplemental Material [file IENZ_A_2225135_SM2703.pdf]
